# Defining the Structure–Activity Relationships of Thiazolide Antivirals: Balancing Potency, Metabolic Stability, and Aqueous Solubility

**DOI:** 10.1002/cmdc.70570

**Published:** 2026-11-13

**Authors:** Joshua Taujanskas, Sophie Pate, Andrew V. Stachulski, Sara Piacentini, Simone La Frazia, M. Gabriella Santoro, Paul M. O’Neill

**Affiliations:** ^1^ Department of Chemistry University of Liverpool Liverpool UK; ^2^ Department of Biology University of Rome Tor Vergata Rome Italy; ^3^ Institute of Translational Pharmacology, CNR Rome Italy

**Keywords:** DMPK optimization, influenza A, medicinal chemistry, nitazoxanide, respiratory virus, thiazolide antivirals

## Abstract

Recent studies on thiazolide antivirals have identified compounds RM5038 and RM5021 as potent inhibitors of hepatitis B virus (HBV), hepatitis C virus (HCV), and influenza A, although their development has been limited by poor aqueous solubility and rapid metabolic clearance. In this work, a series of novel halogen‐substituted and 4′‐alkylsulfone thiazolide analogs were synthesized using updated convergent synthetic approaches and flexible protecting group strategies. DMPK evaluation demonstrated that 3‐X halogen substitution significantly improved aqueous solubility in parallel to enhancing metabolic stability. Within the chlorothiazolide series, compounds **10g**and **10l** displayed much improved metabolic stability profiles, excellent aqueous solubility representing a 10‐fold improvement over the parent drug, together with low single‐digit micromolar antiviral (influenza A) activity and acceptable in vitro safety indices. Evaluation of the 4′‐alkylsulfone thiazolides also revealed improved in vitro DMPK profiles while retaining micromolar antiviral potency. SAR analysis indicated that short alkyl sulfone substituents containing two to three carbons are optimal for antiviral activity, while branched alkyl groups may provide additional metabolic advantages. In summary, compounds **10g, 10l,** and **15d** represent promising early leads for progression to in vivo profiling and additional preclinical evaluation.

## Graphical abstract

Novel halogen‐substituted and 4′‐alkylsulfone thiazolide analogs were developed to overcome the poor solubility and metabolic instability of existing antiviral thiazolides. 3‐X halogen substitution significantly improved aqueous solubility and metabolic stability. Compounds 10g and 10l showed a 10‐fold increase in solubility, enhanced stability, potent influenza A antiviral activity, and acceptable safety profiles. Along with compound 15d, these candidates represent promising leads for further preclinical antiviral development.

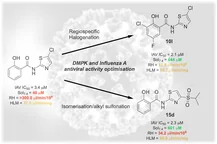

## 1 Introduction

Nitazoxanide (NTZ), the first reported thiazolide, is an FDA‐approved treatment for *Cryptosporidium parvum* and *Giardia intestinalis* [1, 2]. NTZ also exhibits broad‐spectrum activity against parasites, bacteria, and viruses with an excellent safety profile borne out across a range of in vitro, in vivo, and clinical trials [2, 3, 4].

In vivo, NTZ is rapidly hydrolyzed to the phenolic form, tizoxanide (TIZ), both in the gastrointestinal tract and in plasma (t_1/2_ = 6.5 min in plasma, Figure 1) [5]. Upon oral administration, 66.2% of an oral dose of NTZ is expelled in feces as the hydrolysis product, TIZ. In circulation, TIZ remains extensively plasma‐protein bound (>97.5%), reaching a maximum plasma concentration of 1.9 mg/L [6]. The major route of metabolism for TIZ is *O*‐glucuronidation with a half‐life of 1.0–1.6 h [6]. Despite the presence of a number of susceptible functional groups such as the thiazole ring or phenol, there is little evidence of oxidative metabolism of NTZ/TIZ in previous studies [5]. This is likely due to the low overall exposure of the compound in vivo through the combination of low absorption, high plasma‐protein binding, and subsequent rapid glucuronidation [7].

**FIGURE 1:**
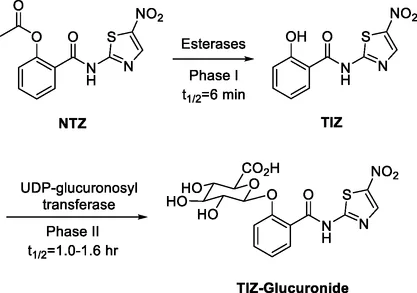
Major routes of phase I and II metabolism of NTZ in vivo.

With its unusually broad spectrum of activity, the mechanism of action of NTZ is of significant interest. A range of specific effects of NTZ against pathogens have been previously identified, including inhibition of pyruvate:ferredoxin/flavodoxin oxidoreductases (PFOR) in anaerobes [8, 9]. In the case of viruses, NTZ was found not to affect virus infectivity, binding, or entry into host cells but to act at the post‐entry level by interfering with nonstructural proteins NSP5 and NSP2 interaction (thus hindering virus morphogenesis) in the case of rotaviruses [10], and by impairing the maturation of several class‐I viral fusion glycoproteins, including influenza hemagglutinin (HA) [11, 12], paramyxovirus fusion F protein [13], and coronavirus spike *S*‐glycoprotein [14, 15]. This effect has been associated to the drug‐mediated inhibition of ERp57, an ER‐resident glycoprotein‐specific thiol‐oxidoreductase which is essential for correct disulfide‐bond architecture of selected viral proteins [13].

A number of host‐cell and immunomodulatory effects have also been reported, including, in addition to ERp57 inhibition, depletion of intracellular Ca^2+^ stores [16], upregulation of protein kinase R (PKR), and activation of interferon‐stimulated genes (ISGs) [17, 18]. Several studies have also shown NTZ to be a mild decoupler of mitochondrial respiration [19, 20, 21, 22, 23], which may play a role in the broad antiviral activity of NTZ and related thiazolide compounds.

Recent development of the thiazolide class has focussed on antiviral activity, with previous investigations identifying potent activity of key thiazolide analogs RM5038 and RM5021 (Figure 2) against hepatitis B virus (HBV), hepatitis C virus (HCV), and more recently influenza A virus (IAV) [24, 25, 26, 27]. Despite promising in vitro activity, NTZ and related thiazolide analogs still suffer from poor DMPK properties, especially low aqueous solubility and rapid metabolic clearance [28]. A number of strategies have been previously investigated for improving the DMPK of the thiazolides, including amino‐acid ester prodrugs (**1** and **2**, Figure 2) and amine salts (**3**, Figure 1) [28, 29]. Both strategies offered improvements in aqueous solubility and absorption; however, the resulting compounds still suffered from rapid glucuronidation in vivo.

**FIGURE 2:**
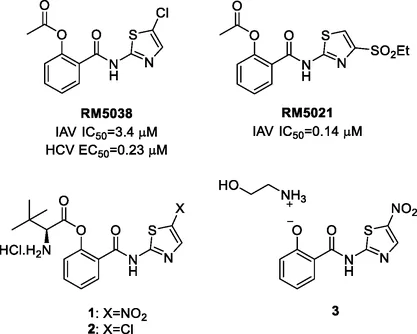
Structures of key thiazolide analogs RM5038 and RM5021 (which previously demonstrated potent antiviral activity against HCV ad IAV) [26, 27] and of thiazolide amino‐acid ester prodrugs **1** and **2** and the thiazolide ethanolamine salt, **3** [28, 29].

Therefore, it was clear that changes to the underlying scaffold would be necessary to overcome poor metabolic clearance. Previous investigations have demonstrated that substitution at the thiazole ring of the thiazolide scaffold has a marked effect on antiviral activity; however, substitutions on the phenyl ring often have minimal impact on antiviral activity [24, 25, 26]. For example, in a previous screen against HBV and HCV, a number of 3/4/5‐Me analogs of both TIZ and RM4848 were evaluated and shown to maintain activity compared the parent compound (Figure 3) [24, 26]. However, these compounds were significantly metabolized by human liver microsomes (HLM) and S9 fraction [25], implying significant oxidative metabolism and the possibility of generation of toxic *p*‐quinone methides [30, 31]. A further study evaluated 3/5‐Me analogs of TIZ for antitubercular activity and DMPK, finding that activity was maintained while plasma clearance was significantly reduced, with a corresponding increase in bioavailability (Figure 3) [32]. However, these analogs also showed significant toxicity in Vero cell lines, plausibly due to the generation of toxic metabolites, as previously implicated in the series.

**FIGURE 3:**
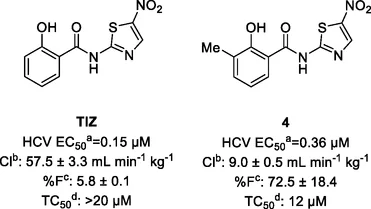
Key HCV activity and pharmacokinetic properties of TIZ and its 3‐Me analog, **4**. ^a^HCV genotype 1B EC_50_ reported by Stachulski et al. [26] ^b^Clearance (Cl), ^c^bioavailability (%F), and ^d^toxic concentration (TC_50_) in Vero cell lines reported by Odingo et al. [32].

The potent antiviral activity of the 4′‐ethylsulfone thiazolide, RM5021, also warranted further investigation. Previous screening elucidated elements of the SAR in this series, namely, that the ethylsulfone was superior to the methylsulfone (IC_50_ vs. IAV = 0.14 µM and 0.29 µM respectively) and the methylsulfone was superior to both the methylsulfoxide or methylsulfide [27]. The cyclohexylsulfone and phenylsulfone were inactive in this screen (A.V. Stachulski—personal communication), suggesting a window of activity between 2 and 5 carbons for the 4′‐alkylsulfone analogs. Furthermore, the DMPK profiles of the 4′‐alkylsulfone thiazolides have not previously been evaluated in detail, and it was proposed that they could offer advantages over TIZ and RM4848.

In this work, we report on a focused medicinal chemistry approach to identify new analogs of TIZ and lead antiviral analogs, RM4848 and RM5021 with (i) improved in vitro metabolic stability and aqueous solubility and (ii) a retention in the potent low micromolar antiviral activity against Influenza A. In addition, the 4′‐alkylsulfone series was expanded via an improved synthetic route to enable both antiviral evaluation of the SAR around the alkyl group and measurement of the in vitro DMPK properties of the 4′‐alkylsulfone thiazolide derivatives for the first time. It was hoped that by maintaining antiviral potency and improving the in vitro ADME, early leads could be identified for further in vivo evaluation.

## 2 Results and Discussion

### 2.1 Chemistry

The synthetic approach to the thiazolides is well established, consisting of protection of the salicylic acid phenol followed by amide coupling with the appropriate 2‐aminothiazole [25, 26, 27]. In the current study, the resulting thiazolide esters were then deprotected to the phenolic form, likely the true active agent in vivo. Furthermore, previous studies have occasionally shown significant differences in activity between the ester and phenol thiazolide derivatives [26], likely due to variable rates of hydrolysis of the phenol ester in different assay conditions. The evaluation of the phenolic thiazolide derivatives is likely to be most representative of the in vivo properties.

Initial synthesis focussed on the RM4848 series, **10a–m** (Figure 4), for which standard acetyl protection of the phenol was sufficient to enable amide coupling with 2‐amino‐5‐chorothiazole hydrochloride under Schotten–Baumann conditions. Deprotection was conveniently achieved with aqueous ammonia to afford the desired thiazolide phenols in good to moderate yields. Most of the required mono/disubstituted salicylic acids were commercially available, except the 3‐F, 5‐Cl, and 3‐Cl, 5‐F derivatives, **5k,** and **5l**. These were synthesized according to conditions previously reported by Silverman et al. [33].

**FIGURE 4:**
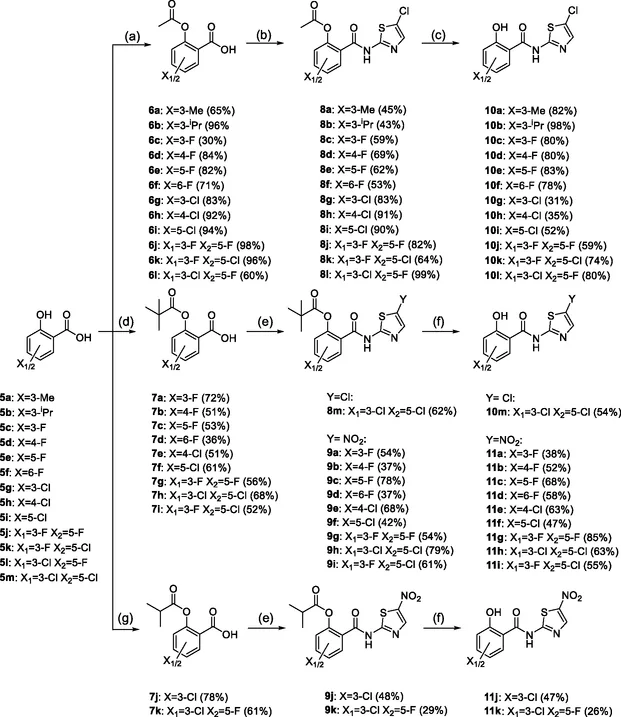
(a) Either (i) Ac_2_O, cat. H_2_SO_4_, reflux, 2 h (ii) distilled H_2_O, rt, 16 h (**6c, 6e–f**) or Ac_2_O, cat. pyridine, rt, 16 h; (**6a–b, 6d,** and **6g–l**); (b) either 5‐chlorothiazol‐2‐amine.HCl, HATU, DIPEA, anh. DCM, rt, 16 h (**8a–f**) or (i) (COCl)_2_, cat. DMF, anh. DCM, rt, 1 h (ii) 5‐chlorothiazol‐2‐amine.HCl, NaHCO_3_, 1:1 H_2_O to EtOAc, rt, 16 h (**8g–l**); (c) aq. NH_3_, THF or acetone, rt, 16 h; (d) (i) pivaloyl chloride, pyridine, anh. THF, rt, 16 h (ii) 12 M aq. HCl, 0 °C‐rt, 1–2 h; (e) (i) SOCl_2_, pyridine, anh. DCM, reflux, 1.5 h (ii) 5‐nitrothiazol‐2‐amine, NEt_3_, anh. THF, rt, 16 h; (f) either: aq. NH_3_, THF or acetone, rt, 16 h (**10m**) or NaOMe, anh. MeOH, rt, 1–7 h (**11a–k**); and (g) (i) isobutyryl chloride, pyridine, anh. THF, rt, 16 h (ii) 8 M aqueous HCl, rt, 0.25–1.0 h.

Synthesis of the TIZ analog series, **11a–k** (Figure 4), proved more challenging, as the poor nucleophilicity of 2‐amino‐5‐nitrothiazole resulted in low yields and significant byproduct formation during amide coupling under Schotten–Baumann conditions. Protection of the phenols as the hindered pivalate esters and subsequent amide coupling under anhydrous conditions allowed access to the desired thiazolide pivalate esters in good to moderate yields. Deprotection of the ester was subsequently achieved by treatment with NaOMe/MeOH.

Interestingly, some analogs possessing chlorine *ortho* to the phenol proved highly resistant to NaOMe/MeOH deprotection, affording incomplete reactions and significant degradation after stirring overnight (c.f. 1–2 h for complete conversion for other analogs under the same conditions). Here, use of the less‐hindered isobutyrate ester afforded the desired thiazolide isobutyrate esters in moderate yield. Deprotection with NaOMe/MeOH now proceeded at a reasonable rate, albeit still significantly slower than analogs bearing F/H *ortho*‐to‐the‐phenol.

The 4′‐alkylsulfone series was synthesized via an updated version of the previously described route to RM5021 (Figure 5) [27]. To enable convenient access to a greater range of analogs, the route was reconfigured around the common intermediate *N*‐(*O*‐pivaloyl)‐salicyloyl thiourea **17** (Figure 6), which was then reacted with the appropriate bromothioester to afford the 4′‐alkylsulfide thiazolide pivaloyl ester. Oxidation with *m*‐CPBA afforded the sulfone, and deprotection with NaOMe furnished the desired thiazolide phenol.

**FIGURE 5:**
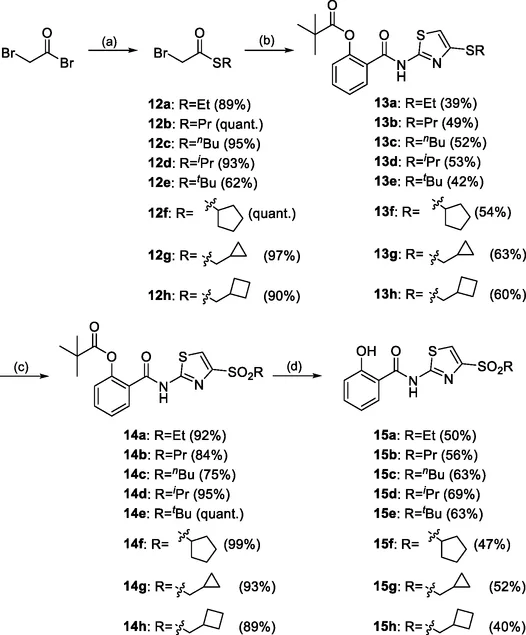
(a) R–SH, anh. Et_2_O, 1–2 h; (b) *N*‐(*O*‐pivaloyl)salicyloyl thiourea, **17**, EtOH, rt, 2–5 days; (c) *m*‐CPBA, DCM, 0 °C to rt, 1–4 h; and (d) NaOMe, MeOH, rt, 1–2 h.

**FIGURE 6:**
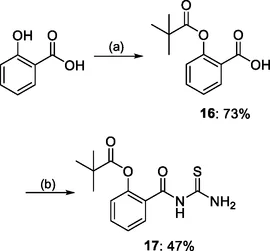
Synthesis of *N*‐(*O*‐pivaloyl)salicyloyl thiourea, **17**, from salicylic acid. (a) (i) pivaloyl chloride, pyridine, THF, 0 °C to rt, 16 h; (ii) HCl (12 M) and (b) (i) SOCl_2_, cat. pyridine, DCM, reflux, 3 h; (ii) NH_4_SCN, MeCN, reflux, 2 h; (iii) aq. NH_3_, 0 °C to rt, 1 h; and (iv) HCl (2 M).

### 2.2 DMPK Evaluation

Upon completion of the synthesis, all final compounds were submitted for DMPK evaluation (alongside comparators TIZ, RM4848, and the 4′‐ethylsulfone thiazolide, **15a**) using a high‐throughput platform provided by AstraZeneca U.K. Methods for the assays have been reported previously [34].

Results of the DMPK evaluation confirmed previous reports that both TIZ and RM4848 are poorly soluble and subject to rapid clearance (Table 1). Both compounds performed particularly poorly in rat hepatocytes (RH), with more moderate clearance observed in HLM. These results are consistent with the known susceptibility of the thiazolides to glucuronidation, compared to the more moderate values obtained in HLM, which generally do not contain phase II enzymes [35]. This suggests a degree of underlying oxidative metabolism which is likely overshadowed in vivo by rapid glucuronidation, hence not observed in previous studies. Interestingly, the 4′‐ethylsulfone analog, **15a**, showed a substantially improved DMPK profile over RM4848 and TIZ both in terms of aqueous solubility (7–10× increase) and metabolic clearance (4–5× reduction in both RH and HLM clearance), although RH clearance remained poor in absolute terms and HLM clearance was moderate. LogD_7.4_ remained within acceptable ranges for all three compounds, and *h*PPB remained high for both RM4848 and the 4′‐ethylsulfone analog (<1% free) (Table 1).

**TABLE 1:** In vitro DMPK evaluation of halogenated thiazolide analogs.

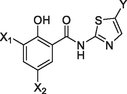								
Compound	**X**_**1**_	**X**_**2**_	Y	***h*PPB** a **,** % free	**LogD**_**7.4**_	**Sol**_**7.4**_ b, µM	**RH** c **,** µl/min/10^6^	**HLM** d **,** µl/min/mg
RM4848	H	H	Cl	<2.80	2.9	48	>300.0	37.5
TIZ	H	H	NO_2_	<0.05	1.8	33	244.0	50.2
**10a**	3‐Me	H	Cl	<2.80	2.8	26	>300	34
**10b**	3‐^i^Pr	H	Cl	<0.30	3.1	118	114	102
**10c**	3‐F	H	Cl	<0.28	1.8	360	75.9	11.9
**10d**	4‐F	H	Cl	0.14	2.8	49	>300.0	41.7
**10e**	5‐F	H	Cl	0.09	2.9	9	>300.0	14.5
**10f**	6‐F	H	Cl	0.22	2.3	211	>300.0	20.2
**10g**	3‐Cl	H	Cl	0.03	1.7	688	29.2	34.1
**10h**	4‐Cl	H	Cl	<0.03	2.8	31	275.0	12.8
**10i**	5‐Cl	H	Cl	<0.03	3.3	1	>300.0	34.0
**10j**	3‐F	5‐F	Cl	<0.26	1.8	62	58.6	18.1
**10k**	3‐F	5‐Cl	Cl	<0.22	2.2	39	19.6	13.1
**10l**	3‐Cl	5‐F	Cl	<0.29	2.1	644	10.6	15.7
**10m**	3‐Cl	5‐Cl	Cl	<0.26	2.7	231	3.0	10.3
**11a**	3‐F	H	NO_2_	0.07	1.1	383	18.5	24.4
**11b**	4‐F	H	NO_2_	0.08	1.5	450	75.9	5.5
**11c**	5‐F	H	NO_2_	0.06	1.7	116	177.0	6.7
**11d**	6‐F	H	NO_2_	0.15	1.1	187	>300.0	44.4
**11j**	3‐Cl	H	NO_2_	<0.06	1.6	581	13.8	40.1
**11e**	4‐Cl	H	NO_2_	<0.06	2.0	133	64.5	13.0
**11f**	5‐Cl	H	NO_2_	<0.03	2.3	292	118.0	17.2
**11g**	3‐F	5‐F	NO_2_	<0.06	1.5	>1000	21.4	<3.0
**11i**	3‐F	5‐Cl	NO_2_	<0.30	2.0	615	12.0	<3.0
**11k**	3‐Cl	5‐F	NO_2_	<3.20	1.9	>1000	6.8	<3.0
**11h**	3‐Cl	5‐Cl	NO_2_	<0.31	2.9	118	7.6	13.2

*Note*: Values determined by high‐throughput assay at AstraZeneca U.K. following previously reported methods [31]. Values color coded according to; green – good, orange – moderate, and red – poor. ND, Not determined.

a *h*PPB: human plasma protein binding based on equilibrium dialysis.

b Sol_7.4_: aqueous solubility in pH 7.4 PBS buffer.

c RH: rat hepatocyte intrinsic clearance.

d HLM: human liver microsomal intrinsic clearance.

Results for the initial alkyl analogs of RM4848, **10a** and **10b**, were mixed. 3‐Me substitution produced a substantial increase in aqueous solubility, and although RH clearance was reduced compared to RM4848, HLM clearance rose significantly. The ^i^Pr analog also showed increased HLM clearance and only a moderate increase in aqueous solubility. These results, in combination with the toxicity concerns raised by the earlier study from Odingo et al. led to further investigation of alkyl analogs being abandoned [32].

Results for the halogenated analogs of RM4848 and TIZ (**10c–m** and **11a–k**) showed that substitution on the phenyl ring did not have a significant effect on human plasma protein binding (*h*PPB). Only one analog, **11k**, had an appreciable fraction of unbound compound (<3.20% free), whilst all others remained highly bound (<1% free). The high plasma binding in this series is likely driven by acidity of the phenol group and, as discussed above, may prove to be a protective factor for the compounds in vivo. LogD_7.4_ values were moderately lowered by halogen substitution, but for all compounds they remained in the favorable range of 1–3 [36]. Only one compound, **10i**, strayed slightly above this range with a logD_7.4_ value of 3.3.

The effect of halogen substitution on aqueous solubility was more pronounced, with complex trends across the series. 3‐Cl or 3‐F monosubstitution consistently enhanced solubility by an order of magnitude or more across both the TIZ and RM4848 series (e.g., **10g**, Sol_7.4_ = 688 µM, c.f. RM4848, Sol_7.4_ = 48 µM). 3‐Cl substitution also offered greater enhancement of solubility over 3‐F (**10c** Sol_7.4_ = 360 µM). The 6‐F analogs in both the RM4848 and TIZ series (**10f** and **11d**) also produced reasonable increases in aqueous solubility (Sol_7.4_ = 211 and 187 µM respectively).

The effect of halogen monosubstitution also varied depending on the thiazole substituent. In the RM4848 series (**10c** **–i**), gains in solubility from both 3‐Cl and 3‐F substitution dropped off significantly as the halogen atom was moved away from the phenol, with the 5‐F and 5‐Cl analogs, **10e** and **10i**, showing extremely low solubility values (Sol_7.4_ = 9 and 1 µM respectively). The trend in the TIZ series (**11a** **–f**) was more complex, whereby monosubstitution did not produce a clear correlation between position and aqueous solubility. Solubility trends also varied between chloro‐ and fluoro‐substituted compounds; for example, solubility of the chloro‐substituted series followed the pattern 3‐Cl > 5‐Cl > 4‐Cl, whereas the fluoro‐substituted series followed 4‐F > 3‐F > 5‐F.

Clear trends in aqueous solubility across the disubstituted analogs were also not immediately apparent. Once again, 3‐Cl substitution provided a significant enhancement to aqueous solubility, but the extent varied depending on both the second phenyl substituent and the thiazole substituent. In the TIZ series, the 3‐Cl, 5‐F analog, **11k**, showed excellent aqueous solubility of >1000 μM. However, the 3,5‐Cl analog, **11h**, had a much lower solubility of 118 μM. In the RM4848 series, the picture was similar, with the 3‐Cl, 5‐F analog, **10l**, also showing excellent aqueous solubility (Sol_7.4_ = 644 μM), while the 3,5‐Cl analog, **10m**, was lower (Sol_7.4_ = 231 μM). 3‐F substitution improved solubility less reliably in the disubstituted RM4848 series, with both the 3,5‐F and 3‐F, 5‐Cl analog (**10j** and **10k**, Sol_7.4_ = 62 and 39 μM) performing far worse than the monosubstituted 3‐F analog, **10c** (Sol_7.4_ = 360 μM). However, this was reversed completely in the TIZ series, with both the 3,5‐F and 3‐F, 5‐Cl analogs (**11g** and **11i**) recording extremely high values for solubility (Sol_7.4_ = >1000 and 615 μM), far higher than the monosubstituted 3‐F analog, **11a** (Sol_7.4_ = 383 μM).

Several analogs also showed dramatic improvements in RH CLint. As discussed, both RM4848 and TIZ performed particularly poorly in this assay (RH CLint = >300.0 and 244.0 µl/min/10^6^ cells, respectively), and a majority of evaluated analogs showed at least a moderate decrease in RH CLint. In the RM4848 series, both 3‐F and 3‐Cl substitution (with and without a second phenyl substituent) produced at least a fourfold decrease in RH Clint, with several analogs performing far better. This effect appeared to be partially additive, with disubstituted compounds showing an additional reduction in CLint over the comparable 3‐substituted analogs. Notably, the 4/5‐Cl (**10h** and **10i**) and 4/5/6‐F (**11b** **–d**) analogs showed practically no improvement in this screen. Similar trends were observed in the TIZ series, with the disubstituted analogs also showing improvements over the corresponding monosubstituted analogs. The only exception was the 3,5‐F analog, **11g**, which showed marginally increased RH CLint compared to the 3‐F analog, **11a** (RH CLint = 21.4 and 18.5 µl/min/10^6^ cells, respectively). The monosubstituted TIZ analogs performed better than the equivalent RM4848 series in terms of RH Clint; however, the 6‐F analog, **11d**, showed no improvement in RH CLint, with a value higher than TIZ (Table 1).

In terms of HLM CLint, both RM4848 and TIZ showed moderate‐to‐poor initial values (HLM CLint = 37.5 and 50.2 µl/min/mg, respectively). Halogen substitution produced mostly moderate reductions in HLM CLint across the series, although several analogs did show substantial improvement. In the RM4848 series, 3‐Cl and 5‐Cl monosubstitution (**10g** and **10i**) had little effect on HLM CLint, although the 4‐Cl analog, **10h**, showed a reasonable reduction (HLM CLint = 12.8 µl/min/mg). Conversely, the 3‐F and 5‐F analogs, **11a** and **11c**, performed well, whereas the 4‐F analog, **11b**, showed a marginal increase (Table 1). The disubstituted analogs in the RM4848 series did not generally display any enhancement in HLM CLint over the comparable monsubstituted analogs, although all were moderately improved over the parent compound. The exception was the 3,5‐Cl analog (**10m**, HLM CLint = 10.3 µl/min/mg), which showed a substantial reduction in HLM CLint over both the 3‐Cl and 5‐Cl monosubstituted analogs, **10g** and **10i**. Trends in the TIZ series were subtly different again, with the 4‐F and 4‐Cl analogs, **11c** and **11e**, performing best in each monosubstituted series (HLM CLint = 13.0 and 5.5 µl/min/‍mg). The disubstituted TIZ analogs were the best performing across the entire series in terms of HLM CLint, with **11g**, **11i,** and **11k** returning HLM CLint values of <3 µl/min/mg.

As discussed above, the 4′‐ethylsulfone thiazolide, **15a**, displayed substantially improved DMPK properties over both TIZ and RM4848 (Table 2). The new 4′‐alkylsulfone analogs continued this trend, all displaying excellent aqueous solubility, with several returning extremely high values (e.g., Sol_7.4_ of **15d** = 601 µM and **15g** = 980 µM). LogD_7.4_ values were in the optimal window of 1–3 [36], although several analogs with shorter‐chain alkyl groups were toward the lower end of that range. Analogs with larger alkyl groups tended to display higher LogD_7.4_ values, reflecting the ability of large alkyl groups to mask the highly polar sulfone moiety. *h*PPB remained high for all analogs (<1% free) despite improved aqueous solubility, once again reflecting the acidity of the phenol group.

**TABLE 2:** In vitro DMPK data for 4′‐alkylsulfone thiazolide analogs.

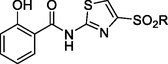						
Compound	**R**	***h*PPB** a **,** % free	**LogD**_**7.4**_	**Sol**_**7.4**_ b **,** µM	**RH** c **,** µl/min/10^6^	**HLM** d **,** µl/min/mg
**15a**	Et	<0.26	1.1	354	57.5	9.51
**15b**	Pr	<0.32	1.5	365	41.5	11.9
**15c**	^*n*^Bu	<0.25	2.1	204	67.1	261.0
**15d**	^*i*^Pr	<0.31	1.5	601	18.5	26.9
**15e**	^*t*^Bu	<0.33	1.8	176	14.6	50.0
**15f**	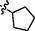	0.08	2.1	237	49.0	>300.0
**15g**	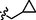	<0.34	1.6	980	34.2	21.3
**15h**	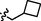	0.05	2.4	566	188.0	>300.0

*Note*: Values determined by high‐throughput assay at AstraZeneca U.K. following previously reported methods [31]. Values color coded according to; green – good, orange – moderate, and red – poor. ND, Not determined.

a *h*PPB: human plasma protein binding based on equilibrium dialysis.

b Sol_7.4_: aqueous solubility in pH 7.4 PBS buffer.

c RH: rat hepatocyte intrinsic clearance.

d HLM: human liver microsomal intrinsic clearance.

The metabolic clearance data for analogs **15a** **–15h** were also encouraging. Values for RH CLint were significantly improved over TIZ and RM4848; however, most analogs still performed poorly overall (defined as an RH CLint of >28 µl/min/10^6^), with the exception of **15d** and **15e** which returned moderate values of 18.5 and 14.6 µl/min/10^6^, respectively. These results are again indicative of significant phase II metabolism, likely glucuronidation, underscoring the need for mediation of reactivity at the phenol. Results for HLM CLint were more mixed. Most analogs displayed moderate clearance rates, with small decreases compared to TIZ and RM4848. However, a small number of analogs (**15c**, **15f**, and **15h**) returned very high values in the range of c.a. 300 µl/min/mg. These results are significantly higher than previous thiazolides and would likely be unsuitable for further development.

### 2.3 Antiviral Activity and Cytotoxicity Evaluation

Compounds were prioritized for antiviral evaluation based on their overall DMPK profiles, with particular emphasis on improvements in metabolic stability and aqueous solubility relative to the parent compounds. Consequently, analogs that failed to show a sufficiently balanced improvement across these parameters were deprioritised. For example, **10f** retained very high rat hepatocyte clearance despite improved solubility, while **11f** showed only a moderate improvement in cellular metabolic stability. Compound **11e** displayed a more favorable profile but was not progressed in preference to TIZ analogs, showing a stronger overall combination of solubility and metabolic stability.

Analogs from both the halogenated RM4848/TIZ and 4′‐alkylsulfone thiazolide series showing positive DMPK profiles were then evaluated for antiviral activity against influenza A virus [IAV strain A/PuertoRico/8/1934(H1N1)] in MDCK cells, Table 3. For the halogenated thiazolide series, the results showed a clear distinction between the RM4848 and TIZ analogs. In the RM4848 series, activity against IAV increased slightly for the halogenated analogs (IC_50_ = 1.6–3.4 μM) over the parent compound, RM4848 (IC_50_ = 3.9 μM), with the exception of **10c**, which showed a moderate decrease in activity (IC_50_ = 5.5 μM). In contrast, the halogenated analogs of TIZ were generally less active than the parent compound, except for **11j** and **11k** which showed only a slight decrease in activity (IC_50_ = 3.3 and 3.1 μM respectively). Of the compounds displaying micromolar activity, the majority showed acceptable selectivity indices. Only **11k** showed potential toxicity concerns with an SI value of 30.0 and an IC_90_ value equal to the LD_50_ at 94.4 μM. These results over the initial antiviral activity screen indicate that the RM4848 series tolerates halogen substitution far more readily than TIZ.

**TABLE 3:** In vitro antiviral activity of selected halogenated and 4′‐alkylsulfone thiazolide analogs.

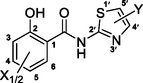							
Compound	**X**_**1**_	**X**_**2**_	Y	**IC**_**50**_ **,** **μM** a	**IC**_**90**_ **,** **μM** b	**LD**_**50**_ **,** **μM** c	**SI** d
RM4848	H	H	5′‐Cl	3.9	19.6	>196.3	>50.0
TIZ	H	H	5′‐NO_2_	2.3	26.4	>188.5	>83.3
**15a**	H	H	4′‐SO_2_Et	0.8	8.0	160	200
**10c**	3‐F	H	5′‐Cl	5.5	23.5	>183.4	>33.3
**10g**	3‐Cl	H	5′‐Cl	2.1	69.2	>172.9	>83.3
**10j**	3‐F	5‐F	5′‐Cl	3.4	61.9	>172.0	>50.6
**10k**	3‐F	5‐Cl	5′‐Cl	2.0	97.7	>162.8	>83.3
**10l**	3‐Cl	5‐F	5′‐Cl	1.6	97.7	>162.8	>100.0
**10m**	3‐Cl	5‐Cl	5′‐Cl	2.5	92.7	154.5	62.5
**11a**	3‐F	H	5′‐NO_2_	12.0	>177	>177	>14
**11b**	4‐F	H	5′‐NO_2_	56.5	123.6	>176.5	>3.1
**11g**	3‐F	5‐F	5′‐NO_2_	83.0	132.8	>166.0	>2.0
**11h**	3‐Cl	5‐Cl	5′‐NO_2_	23.9	89.8	89.8	3.8
**11i**	3‐F	5‐Cl	5′‐NO_2_	94.4	ND	94.4	1.0
**11j**	3‐Cl	H	5′‐NO_2_	3.3	100.1	>166.8	>50.0
**11k**	3‐Cl	5‐F	5′‐NO_2_	3.1	94.4	94.4	30.0
**15b**	H	H	4′‐SO_2_Pr	1.5	21.4	153	100
**15d**	H	H	4′‐SO_2_^*i*^Pr	2.3	35.2	153	67
**15g**	H	H	4′‐SO_2_CH_2_cPr^e^	8.9	103.4	148	17

a IC_50_: 50% inhibitory concentration.

b IC_90_: 90% inhibitory concentration.

c LD_50_: 50% cytotoxic concentration.

d SI: selectivity or safety index = LD_50_/IC_50_. IC_50_/IC_90_ values against IAV were determined in MDCK cells following infection with A/PR/8/1934(H1N1) (PR8) IAV by previously reported methods [27]. LD_50_ was determined using the previously reported MTT formazan dye assay [37].

e cPr: cyclopropyl.

Overall, these findings reveal a complex structure–activity relationship in which modifications designed to enhance metabolic stability also influence antiviral potency. This likely reflects the effects of structural changes on both access to the antiviral target and the molecular interactions required for productive target engagement. Encouragingly, several RM4848 analogs combined improved DMPK profiles with micromolar antiviral activity. In particular, the 3‐Cl, 5‐F analog **10l** was among the best‐performing compounds in the DMPK screen while retaining low micromolar activity against IAV (IC_50_ = 1.6 µM). Compounds **10c** and **10g** similarly showed strong antiviral activity together with marked improvements in DMPK properties relative to the parent compound. Taken together, these results define useful SAR for balancing antiviral potency with metabolic stability and other drug‐like properties, providing a strong basis for further optimization of the thiazolide series.

For the 4′‐alkylsulfone thiazolides, results of the antiviral screen were broadly in line with previous trends identified in the series. The ethylsulfone analog (**15a**, IC_50_ = 0.8 µM) showed comparable activity to the previous 4′‐alkylsulfone thiazolide screen (acetyl analog of **15a**, RM5021, gave IC_50_ = 0.14 µM) [27]. A clear correlation between the size of the alkyl group and the antiviral activity was also evident, with an 11‐fold reduction in activity between the ethyl analog and the cyclopropylmethyl. The branching of the alkyl group may also influence activity, with straight chains being preferred, as seen between the isomeric *n*‐propyl and isopropyl analogs (**15b** and **15d**, IC_50_ = 1.5 and 2.3 µM respectively). Overall, it appears that smaller, less‐hindered alkyl groups are optimal for high antiviral activity against IAV. Additionally, toxicity of all analogs was consistently low, with LD_50_ values falling between 148 and 160 µM. Hence, excellent SI values were observed for the most active analogs.

The antiviral screening results for the 4′‐alkylsulfone thiazolide analogs confirm the activity of these compounds is highly sensitive to the size and shape of the alkyl group. Previous screening suggested that the upper limit for antiviral activity was likely around the 5–6 carbon range. However, results from this investigation demonstrate a much tighter range of 2–3 carbons is necessary for potent antiviral activity. The ethylsulfone analog, **15a**, remains the lead compound in the series in terms of antiviral activity (IC_50_ = 0.8 µM), but this may be offset by the improved DMPK profile offered by, for example, the isopropyl analog, **15d**, which was only moderately less active in this screen.

## 3 Conclusions

In the current program, a range of novel halogen‐substituted and 4′‐alkylsulfone thiazolide analogs have been synthesized and evaluated for in vitro DMPK properties and antiviral activity versus IAV. New, flexible thiazolide protecting group chemistry has been developed as well as an updated, convergent synthesis of the 4′‐alkylsulfone thiazolides. DMPK evaluation has demonstrated that halogen substitution at the 3‐X position represents a powerful strategy for improving aqueous solubility and modulating metabolic clearance of the thiazolides. The leading candidates from the chlorothiazolide series are**10g** and **10l**, which display excellent aqueous solubility (10‐fold > parent drug), low single‐digit antiviral activity, and an acceptable in vitro safety window of around 80‐fold > 100; **10l** has the superior in vitro metabolic stability profile of this pair. The DMPK profile of the 4′‐alkylsulfone thiazolides has also been evaluated for the first time, showing significant improvements over previous thiazolides while maintaining micromolar antiviral activity. Elements of the SAR for the 4′‐alkylsulfone thiazolides have been refined, indicating that short alkyl groups of 2–3 carbons are likely to be optimal for potent antiviral activity, but that this should be weighed against potential DMPK improvements from branched alkyl groups. Additional progression of **15d** to in vivo studies is encouraged given its strong antiviral performance and enhanced metabolic stability profiles.

## Funding

This work was supported by the University of Liverpool (10.13039/501100000836).

## Conflicts of Interest

The authors declare no conflicts of interest.

## Supporting information

Supplementary Material

## Data Availability

The data that support the findings of this study are available in the Supporting Information of this article.

## References

[1] L. M. Fox and L. D. Saravolatz , “Nitazoxanide: A New Thiazolide Antiparasitic Agent,” Clinical Infectious Diseases 40, no.8 (2005): 1173–1180, 10.1086/428839. 15791519

[2] J.‐F. Rossignol , “Nitazoxanide: A First‐in‐Class Broad‐Spectrum Antiviral Agent,” Antiviral Research 110 (2014): 94–103, 10.1016/j.antiviral.2014.07.014. PMC7113776 25108173

[3] J.‐F. Rossignol , “Thiazolides: A New Class of Antiviral Drugs,” Expert Opinion on Drug Metabolism & Toxicology 5, no. 6 (2009): 667–674, 10.1517/17425250902988487. 19442032

[4] A. V. Stachulski , J. Taujanskas , S. L. Pate , et al., “Therapeutic Potential of Nitazoxanide: An Appropriate Choice for Repurposing versus SARS‐CoV‐2?,” ACS Infectious Diseases 7, no. 6 (2021): 1317–1331, 10.1021/acsinfecdis.0c00478. 33352056

[5] J. Broekhuysen , A. Stockis , R. L. Lins , J. G. De , and J. F. Rossignol , “Nitazoxanide: Pharmacokinetics and Metabolism in Man,” International Journal of Clinical Pharmacology and Therapeutics 38, no. 8 (2000): 387–394. 10.5414/cpp38387 10984012

[6] A. Stockis , X. Deroubaix , R. Lins , B. Jeanbaptiste , P. Calderon , and J. F. Rossignol , “Pharmacokinetics of Nitazoxanide After Single Oral Dose Administration in 6 Healthy Volunteers,” International Journal of Clinical Pharmacology and Therapeutics 34, no. 8 (1996): 349–351. 8864798

[7] T. Bohnert and Gan , “L.‐S. Plasma Protein Binding: From Discovery to Development,” Journal of Pharmaceutical Sciences 102, no. 9 (2013): 2953–2994, 10.1002/jps.23614. 23798314

[8] G. Sisson , A. Goodwin , A. Raudonikiene , et al., “Enzymes Associated with Reductive Activation and Action of Nitazoxanide, Nitrofurans, and Metronidazole in Helicobacter Pylori,” Antimicrobial Agents and Chemotherapy 46, no. 7 (2002): 2116–2123, 10.1128/AAC.46.7.2116-2123.2002. PMC127316 12069963

[9] P. S. Hoffman , G. Sisson , M. A. Croxen , et al., “Antiparasitic Drug Nitazoxanide Inhibits the Pyruvate Oxidoreductases of Helicobacter Pylori, Selected Anaerobic Bacteria and Parasites, and Campylobacter Jejuni,” Antimicrobial Agents and Chemothera 51, no. 3 (2007): 868–876, 10.1128/AAC.01159-06. PMC1803158 17158936

[10] S. La Frazia , A. Ciucci , F. Arnoldi , et al., “Thiazolides, a New Class of Antiviral Agents Effective Against Rotavirus Infection, Target Viral Morphogenesis, Inhibiting Viroplasm Formation,” Journal of Virology 87, no. 20 (2013): 11096–11106, 10.1128/JVI.01213-13. PMC3807293 23926336

[11] J. ‐F. Rossignol , S. La Frazia , L. Chiappa , A. Ciucci , and M. G. Santoro , “Thiazolides, a New Class of Anti‐influenza Molecules Targeting Viral Hemagglutinin at the Post‐translational Level,” Journal of Biological Chemistry 284, no. 43 (2009): 29798–29808, 10.1074/jbc.M109.029470. PMC2785610 19638339

[12] S. La Frazia , S. Piacentini , A. Riccio , J.‐F. Rossignol , and M. G. Santoro , “The Second‐Generation Thiazolide Haloxanide Is a Potent Inhibitor of Avian Influenza Virus Replication,” Antiviral Research 157 (2018): 159–168, 10.1016/j.antiviral.2018.06.008. 29908209

[13] S. Piacentini , S. La Frazia , A. Riccio , et al., “Nitazoxanide Inhibits Paramyxovirus Replication by Targeting the Fusion Protein Folding: Role of Glycoprotein‐Specific Thiol Oxidoreductase ERp57,” Scientific Reports 8 (2018): 10425, 10.1038/s41598-018-28172-9. PMC6041319 29992955

[14] A. Riccio , S. Santopolo , A. Rossi , S. Piacentini , J.‐F. Rossignol , and M. G. Santoro , “Impairment of SARS‐CoV‐2 Spike Glycoprotein Maturation and Fusion Activity by Nitazoxanide: an Effect Independent of Spike Variants Emergence,” Cellular and Molecular Life Sciences 79 (2022): 227, 10.1007/s00018-022-04246-w. PMC8989121 35391601

[15] S. Piacentini , A. Riccio , S. Santopolo , et al., “The FDA‐Approved Drug Nitazoxanide is a Potent Inhibitor of Human Seasonal Coronaviruses Acting at Postentry Level: Effect on the Viral Spike Glycoprotein,” Frontiers in Microbiology 14 (2023): 1–13, 10.3389/fmicb.2023.1206951. PMC10497118 37705731

[16] O. Ashiru , J. D. Howe , and T. D. Butters , “Nitazoxanide, an Antiviral Thiazolide, Depletes ATP‐Sensitive Intracellular Ca^2+^ Stores,” Virology 462‐463 (2014): 135–148, 10.1016/j.virol.2014.05.015. 24971706

[17] M. Elazar , M. Liu , S. A. McKenna , et al., “The Anti‐Hepatitis C Agent Nitazoxanide Induces Phosphorylation of Eukaryotic Initiation Factor 2α Via Protein Kinase Activated by Double‐Stranded RNA Activation,” Gastroenterology 137, no. 5 (2009): 1827–1835, 10.1053/j.gastro.2009.07.056. 19664635

[18] W. Dang , L. Xu , B. Ma , et al., “Nitazoxanide Inhibits Human Norovirus Replication and Synergizes with Ribavirin by Activation of Cellular Antiviral Response,” Antimicrobial Agents and Chemotherapy 62, no. 11 (2018): e00707–e00718, 10.1128/AAC.00707-18. PMC6201076 30104275

[19] J. T. Mesquita , E. G. Pinto , N. N. Taniwaki , A. J. Galisteo , and A. G. Tempone , “Lethal Action of the Nitrothiazolyl‐Salicylamide Derivative Nitazoxanide via Induction of Oxidative Stress in Leishmania (L.) Infantum,” Acta Tropica 128, no. 3 (2013): 666–673, 10.1016/j.actatropica.2013.09.018. 24071379

[20] W. Senkowski , X. Zhang , M. H. Olofsson , et al., “Three‐Dimensional Cell Culture‐Based Screening Identifies the Anthelmintic Drug Nitazoxanide as a Candidate for Treatment of Colorectal Cancer,” Molecular Cancer Therapeutics 14, no. 6 (2015): 1504–1516, 10.1158/1535-7163.MCT-14-0792. 25911689

[21] S. Sahdeo , A. Tomilov , K. Komachi , et al., “High‐Throughput Screening of FDA‐Approved Drugs Using Oxygen Biosensor Plates Reveals Secondary Mitofunctional Effects,” Mitochondrion 17 (2014): 116–125, 10.1016/j.mito.2014.07.002. PMC4142054 25034306

[22] N. Amireddy , S. N. Puttapaka , R. L. Vinnakota , H. G. Ravuri , S. Thonda , and S. V. Kalivendi , “The Unintended Mitochondrial Uncoupling Effects of the FDA‐Approved Anti‐Helminth Drug Nitazoxanide Mitigates Experimental Parkinsonism in Mice,” Journal of Biological Chemistry 292, no. 38 (2017): 15731–15743, 10.1074/jbc.M117.791863. PMC5612106 28798236

[23] F. Ek , K. Blom , T. Selvin , et al., “Sorafenib and Nitazoxanide Disrupt Mitochondrial Function and Inhibit Regrowth Capacity in Three‐Dimensional Models of Hepatocellular and Colorectal Carcinoma,” Scientific Reports 12, no. 1 (2022): 8943, 10.1038/s41598-022-12519-4. PMC9142582 35624293

[24] B. E. Korba , A. B. Montero , K. Farrar , et al., “Tizoxanide and Other Thiazolides Are Potent Inhibitors of Hepatitis B Virus and Hepatitis C Virus Replication,” Antiviral Research 77, no. 1 (2008): 56–63, 10.1016/j.antiviral.2007.08.005. 17888524

[25] A. V. Stachulski , C. Pidathala , E. C. Row , et al., “Thiazolides as Novel Antiviral Agents. 1. Inhibition of Hepatitis B Virus Replication,” Journal of Medicinal Chemistry 54, no. 12 (2011): 4119–4132, 10.1021/jm200153p. PMC3124649 21553812

[26] A. V. Stachulski , C. Pidathala , E. C. Row , et al., “J.‐F. Thiazolides as Novel Antiviral Agents. 2. Inhibition of Hepatitis C Virus Replication,” Journal of Medicinal Chemistry 54, no. 24 (2011): 8670–8680, 10.1021/jm201264t. 22059983

[27] A. V. Stachulski , M. G. Santoro , S. Piacentini , et al., “Second‐Generation Nitazoxanide Derivatives: Thiazolides are Effective Inhibitors of the Influenza A Virus,” Future Medicinal Chemistry 10, no. 8 (2018): 851–862, 10.4155/fmc-2017-0217. 29629834

[28] A. V. Stachulski , K. Swift , M. Cooper , et al., “Synthesis and Pre‐Clinical Studies of New Amino‐Acid Ester Thiazolide Prodrugs,” European Journal of Medicinal Chemistry 126 (2017): 154–159, 10.1016/j.ejmech.2016.09.080. PMC7125651 27750149

[29] A. V. Stachulski , J.‐F. Rossignol , S. Pate , et al., “Antiviral Activity, Preliminary Pharmacokinetics and Structural Parameters of Thiazolide Amine Salts,” Future Medicinal Chemistry 13, no. 20 (2021): 1731–1741, 10.4155/fmc-2021-0055. 34402654

[30] T. J. Monks and D. C. Jones , “The Metabolism and Toxicity of Quinones, Quinonimines, Quinone Methides, and Quinone‐Thioethers,” Current Drug Metabolism 3, no. 4 (2002): 425–438, 10.2174/1389200023337388. 12093358

[31] D. C. Thompson , K. Perera , and R. London , “Quinone Methide Formation from Para Isomers of Methylphenol (Cresol), Ethylphenol, and Isopropylphenol: Relationship to Toxicity,” Chemical Research in Toxicology 8, no. 1 (1995): 55–60, 10.1021/tx00043a007. 7703367

[32] J. Odingo , M. A. Bailey , M. Files , et al., “In Vitro Evaluation of Novel Nitazoxanide Derivatives Against Mycobacterium Tuberculosis,” ACS Omega 2, no. 9 (2017): 5873–5890, 10.1021/acsomega.7b00892. PMC6044914 30023755

[33] F. P. Silverman , P. D. Petracek , D. F. Heiman , C. M. Fledderman , and P. Warrior , “Salicylate Activity. 3. Structure Relationship to Systemic Acquired Resistance,” Journal of Agricultural and Food Chemistry 53, no. 25 (2005): 9775–9780, 10.1021/jf051383t. 16332130

[34] J. Wernevik , F. Bergström , A. Novén , et al., “A Fully Integrated Assay Panel for Early Drug Metabolism and Pharmacokinetics Profiling,” ASSAY and Drug Development Technologies 18, no. 4 (2020): 157–179, 10.1089/adt.2020.970. PMC7567642 32407132

[35] E. F. A. Brandon , C. D. Raap , I. Meijerman , J. H. Beijnen , and J. H. M. Schellens , “Update on in Vitro Test Methods in Human Hepatic Drug Biotransformation Research: Pros and Cons,” Toxicology and Applied Pharmacology 189, no. 3 (2003): 233–246, 10.1016/S0041-008X(03)00128-5. 12791308

[36] M. J. Waring , “Lipophilicity in Drug Discovery,” Expert Opinion on Drug Discovery 5, no. 3 (2010): 235–248, 10.1517/17460441003605098. 22823020

[37] M. G. Santoro , C. Amici , G. Elia , A. Benedetto , and E. Garaci , “Inhibition of Virus Protein Glycosylation as the Mechanism of the Antiviral Action of Prostaglandin A in Sendai Virus‐Infected Cells,” Journal of General Virology 70 (1989): 789–800, 10.1099/0022-1317-70-4-789. 2543761

