# Supplementary material for: Defining the Structure–Activity Relationships of Thiazolide Antivirals: Balancing Potency, Metabolic Stability, and Aqueous Solubility
Source: ChemMedChem. 2026 Nov 13;21(22):e70570. doi: 10.1002/cmdc.70570 (PMC13768565; doi:10.1002/cmdc.70570)
Supplement: Supplementary file 1 [file CMDC-21-e70570-s001.pdf]

Defining the Structure–Activity Relationships of Thiazolide Antivirals: Balancing Potency, Metabolic Stability and Aqueous Solubility

Joshua Taujanskas,[1‡] Sophie Pate,[1‡] Andrew V. Stachulski,[1] Sara Piacentini,[2] Simone La Frazia,[2] M. Gabriella Santoro[2,3] and Paul M. O'Neill[1]*

[1] *Department of Chemistry, University of Liverpool, L69 7ZD, Liverpool, UK*

[2] *Department of Biology, University of Rome Tor Vergata, Rome, Italy*

[3] *Institute of Translational Pharmacology, CNR, Rome, Italy*

‡These authors contributed equally to this study

Contents

1. Chemistry 3
   1.1 General Experimental Information 3
   1.2 Synthesis Of Chloro-fluoro Disubstituted Salicylic Acids 3
   1.3 General Procedures for Synthesis of *O*-Acylated Salicylic acids 6a-l, 7a-k 4
      1.3.1 Pyridine Catalysed Acetylation 4
      1.3.2 Sulfuric Acid Catalysed Acetylation 4
      1.3.3 Acylation With Pivaloyl Chloride or Isobutyryl Chloride 4
   1.4 General Procedures for Synthesis of *O*-Acyl Thiazolides, 8a-m, 9a-k 9
      1.4.1 HATU/DIPEA Amide Coupling 9
      1.4.2 Schotten-Baumann Amide Coupling 9
      1.4.3 Anhydrous Amide Coupling 9
   1.5 General Procedures for Synthesis of Thiazolides 10a-m, 11a-k 14
      1.5.1 Aminolysis of *O*-Acyl Thiazolides[8] 14
      1.5.2 Zemplén Deacylation of *O*-Acyl Thiazolides 14
   1.6 General Procedures for Synthesis of 4’-Alkylsulfone Thiazolides, 15a-h 19
      1.6.1 Bromothioester Synthesis[10] 19
      1.6.2 Hantzsch Thiazole Synthesis 20
      1.6.3 Oxidation of *O*-Pivaloyl-4’-alkylsulfide Thiazolides 20
      1.6.4 Zemplén Deacylation of *O*-Pivaloyl-4’-alkylsulfone Thiazolides. 20
2. Antiviral Assay Procedures 27
   2.2 Cell Culture and Treatments 27
   2.2 Virus Preparation, Infection and Titration 27
   2.3 DMPK Analysis

3. NMR Spectra of Compounds 10a-m, 11a-k, 15a-h 29
4. References 68

1. Chemistry

1.1 General Experimental Information

Chemicals were purchased from commercial sources (Fluorochem, Sigma-Aldrich, Acros Organics, Fisher Scientific or Enamine Ltd.) or obtained from Romark Laboratories and used without any further purification, unless otherwise stated. Column chromatography was performed on silica gel (Aldrich, 40−63 µm, 230-400 mesh), eluting with various solvent mixtures and using an airline to apply pressure. Thin layer chromatography was performed using aluminium backed silica plates (Merck, 0.25 mm, 60 F-254). Visualisation was achieved by UV fluorescence or using $KMnO_4$ or *para*-anisaldehyde followed by gentle heating.

Infra-red spectra were recorded in the range of 4000-600 cm^{-1} using a JASCO FT/IR 4200 spectrometer, or Bruker ALPHA FT-IR platinum ATR spectrometer. Elemental analyses were determined by the University of Liverpool Microanalysis Laboratory and the data are presented as (C%), (H%), (N%), (S%). Melting points (MP) were carried out using a Gallenkamp melting point machine and values were recorded in degrees Celsius with three concordant results.

Nuclear magnetic resonance spectra were recorded on either Bruker AMX 400 (^1H, 400 MHz; ^{13}C, 101 MHz; ^{19}F, 376 MHz) or Bruker 500 (^1H, 500 MHz; ^{13}C, 126 MHz) spectrometers. Chemical shifts (δ) are reported as ppm relative to residual solvent peak. Coupling constants (J) are reported in Hertz (Hz) and the splitting patterns were recorded as follows: s, singlet; d, doublet; t, triplet; q, quartet; sept, septet; dd, doublet of doublets; dt, doublet of triplets; m, multiplet.

Mass spectrometry was conducted by the analytical service within the Chemistry Department at the University of Liverpool. Mass spectrometry data using electron ionisation or chemical ionisation were recorded on an Agilent 7200 QTOF mass spectrometer. Where chemical ionisation was used either methane or ammonia were used as the carrier gas. Mass spectrometry data using electrospray ionisation were recorded on a Micromass TOF spectrometer.

HPLC analysis was performed using an Agilent 1200 HPLC system equipped with ZORBAX Eclipse Plus C18 (4.6 mm × 100 mm, 3.5 µm) column at 25 °C with 1.0 mL/min flow rate monitoring at 254 nm. Solvent – A) water containing 0.05% trifluoroacetic acid or 0.1% formic acid and B) acetonitrile containing 0.05% trifluoroacetic acid or 0.1% formic acid; Method (Acidic, 2-98%): Run time: 15 min, gradient: 2% B holds to 1 min, 2-98% B in 11 min, then holds at 98% B to 15 min.

1.2 Synthesis Of Chloro-fluoro Disubstituted Salicylic Acids

5-Chloro-3-fluoro-2-hydroxybenzoic acid, 5k[1]

A solution of 3-fluoro-2-hydroxybenzoic acid (3.02 g, 19.37 mmol, 1.0 eq) in dioxane (20 mL) was cooled to 0 °C before HCl (6 M, 12.8 mL, 76.80 mmol, 4.0 eq) was added dropwise, followed by dropwise aqueous H_2O_2 (35% wt/wt solution, 6.75 mL, 78.50 mmol, 4.1 eq). The reaction was protected from light then allowed to warm to room temperature and stirred vigorously at room temperature overnight. Upon completion the reaction was extracted with ethyl acetate (3 x 100 mL) and the combined organics were washed with brine (50 mL). The organics were then dried over $MgSO_4$, filtered and the solvent removed *in vacuo* to afford an off-white solid. Recrystallisation from 1,2-dichloroethane afforded the title compound as a white crystalline solid (2.84 g, 77%). ^1H NMR (400

MHz, $(CD_3)_2SO$) δ_H 7.68 (1H, dd, *J*=10.7, 2.4 Hz) and 7.55 (1H, m). ^{13}C NMR (101 MHz, $(CD_3)_2SO$) δ_C 170.38 (d, *J*=3.7 Hz), 151.28 (d, *J*=247.9 Hz), 148.77 (d, *J*=13.0 Hz), 125.19 (d, *J*=3.5 Hz), 121.97 (d, *J*=9.5 Hz), 121.84 (d, *J*=21.1 Hz) and 117.20 (d, *J*=3.8 Hz). $^{19}F\{^1H\}$ NMR (376 MHz, $(CD_3)_2SO$) δ_F −133.34 (s). HRMS (ES−) *m/z* calcd for $C_7H_3ClFO_3$: 188.9760. Found $[M-H]^-$ 188.9760 (diff 0.36 ppm).

3-Chloro-5-fluoro-2-hydroxybenzoic acid, 5l[1]

A solution of 5-fluoro-2-hydroxybenzoic acid (3.00 g, 19.25 mmol, 1.0 eq) in dioxane (20 mL) was cooled to 0°C before HCl (12 M, 6 mL, 192.0 mmol, 10.0 eq) was added dropwise, followed by dropwise aqueous H_2O_2 (35% wt/wt, 16.5 mL, 10.0 eq). The reaction was allowed to stir at 0 °C for 10 mins, then diluted with H_2O (16.0 mL) and allowed to warm to room temperature. The reaction was protected from light and stirred vigorously for four days. The reaction was extracted with ethyl acetate (3 x 100 mL), then the combined organics were washed with brine (50 mL), dried over $MgSO_4$ and filtered before the solvent was removed *in vacuo*. The crude product was recrystallised from 1,2-dichloroethane to afford a pale yellow crystalline solid (1.83 g, 50%).^1H NMR (400 MHz, $(CD_3)_2SO$) δ_H 11.70 (1H, br s), 7.76 (1H, dd, *J*=8.4, 3.0 Hz) and 7.54 (1H, dd, *J*=8.4, 3.0 Hz). ^{13}C NMR (101 MHz, $(CD_3)_2SO$) δ_C 171.13 (d, *J*=2.6 Hz), 154.07 (d, *J*=2.1 Hz), 153.86 (d, *J*=239.2 Hz), 123.47 (d, *J*=26.7 Hz), 121.86 (d, *J*=10.5 Hz), 115.05 (d, *J*=23.6 Hz) and 114.99. $^{19}F\{^1H\}$ NMR (376 MHz, $(CD_3)_2SO$) δ_F -122.61 (s). HRMS (ES−) *m/z* calcd for $C_7H_3ClFO_3$: 188.9760. Found $[M-H]^-$ 188.9759 (diff 0.76 ppm).

1.3 General Procedures for Synthesis of *O*-Acylated Salicylic acids 6a-l, 7a-k

1.3.1 Pyridine Catalysed Acetylation

To a suspension of the appropriate salicylic acid in acetic anhydride (1.7 mL $mmol^{-1}$) was added pyridine (2-3 drops). The reaction was allowed to stir overnight at room temperature, then the reaction was acidified with aqueous HCl (1 M, 4 volumes) and allowed to stir at 0 °C for 30 minutes. The resulting suspension was extracted with ethyl acetate or DCM (3 x 5 volumes) and the combined organics dried over $MgSO_4$ and filtered. The solvent was removed *in vacuo* and, if necessary, the crude product purified by recrystallisation from ethyl acetate and *n*-hexane to afford the desired product.

1.3.2 Sulfuric Acid Catalysed Acetylation

To a suspension of the appropriate salicylic acid in acetic anhydride (0.5 mL $mmol^{-1}$) was added conc. H_2SO_4 (2 drops). The reaction was allowed to stir for 3 h, then poured onto ice with stirring. Upon completion the resulting precipitate was filtered and dried *in vacuo* or, if necessary, extracted with ethyl acetate, dried over $MgSO_4$, filtered and evaporated to afford the crude product. Recrystallisation from ethyl acetate and *n*-hexane afforded the desired product.

1.3.3 Acylation With Pivaloyl Chloride or Isobutyryl Chloride

A solution of the relevant salicylic acid (1.0 eq) in anh. THF (2.1 mL $mmol^{-1}$) under N_2 was was cooled to 0°C, then the appropriate acyl chloride (3.0 eq) was added dropwise, followed by dropwise anh. pyridine (1.0 eq). The reaction was warmed to room temperature and allowed to stir vigorously overnight. Upon completion the reaction was cooled to 0°C, then acidified with aqueous HCl (8-12 M, 1 volume) and allowed to stir until a precipitate formed, adding extra HCl if necessary. The reaction was then extracted with ethyl acetate (3 x 4 volumes). The combined organics were washed with H_2O (2 x 4 volumes) and brine (4 volumes), dried over $MgSO_4$, and filtered. The solvent was removed *in vacuo* and the crude product purified by recrystallisation if necessary.

2-Acetoxy-3-methylbenzoic acid, 6a[2]

Prepared according to general procedure 1.3.1 from 2-hydroxy-3-methylbenzoic acid. Obtained a white crystalline solid (0.83 g, 65%). ^{1}H NMR (400 MHz, $CDCl_3$) δ_H 7.95 (d, *J*=7.6 Hz, 1H), 7.49 (d, *J*=7.6, 1H), 7.25 (app. t, *J*=7.6, 1H), 2.37 (s, 3H) and 2.25 (s, 3H). ^{13}C NMR (101 MHz, $CDCl_3$) δ_C 170.0, 169.3, 149.8, 136.4, 132.4, 130.2, 125.8, 122.1, 20.8 and 16.1. HRMS (ES−) *m/z* calcd for $C_{10}H_9O_4$: 193.0506. Found $[M-H]^-$: 193.0498 (diff 4.31 ppm).

2-Acetoxy-3-isopropylbenzoic acid, 6b[3]

Prepared according to general procedure 1.3.1 from 2-hydroxy-3-isopropylbenzoic acid. Obtained a white solid (1.2 g, 96%). ^{1}H NMR (400 MHz, $CDCl_3$) δ_H 7.96 (dd, *J*=7.6, 1.6 Hz, 1H), 7.57 (dd, *J*=7.6, 1.6 Hz, 1H), 7.31 (app. t, *J*=7.6 Hz, 1H), 3.13 (sept, *J*=7.0 Hz, 1H), 2.37 (s, 3H) and 1.23 (d, *J*=7.0 Hz, 6H). ^{13}C NMR (101 MHz, $CDCl_3$) δ_C 170.7, 169.9, 148.6, 142.4, 132.3, 130.1, 126.1, 122.1, 27.2, 23.0 and 21.0. HRMS (ES+) *m/z* calcd for $C_{12}H_{14}NaO_4$: 245.0784. Found $[M+Na]^+$: 245.0791 (diff −2.82 ppm).

2-Acetoxy-3-fluorobenzoic acid, 6c[4]

Prepared according to general procedure 1.3.2 from 3-fluoro-2-hydroxybenzoic acid. Obtained a white crystalline solid (1.54 g, 78%). ^{1}H NMR (400 MHz, $CDCl_3$) δ_H 7.87 (m, 1H), 7.42 (m, 1H), 7.32 (m, 1H) and 2.39 (s, 3H). ^{13}C NMR (101 MHz, $CDCl_3$) δ_C 169.0, 168.6, 155.1 (d, *J*=250 Hz), 139.4 (d), 127.6 (d), 126.6 (d), 124.3. 121.6 (d) and 20.5. ^{19}F{^{1}H} NMR (376 MHz, $CDCl_3$) δ_F -128.2. Elemental analysis calcd (%) for $C_9H_7FO_4$: C, 54.6; H, 3.6; found: C, 54.6; H, 3.5.

2-Acetoxy-4-fluorobenzoic acid, 6d[3]

Prepared according to general procedure 1.3.1 from 4-fluoro-2-hydroxybenzoic acid. Obtained a white solid (2.1 g, 84%).^{1}H NMR (400 MHz, $CDCl_3$) δ_H 8.15 (dd, *J*=8.8, 6.4 Hz, 1H), 7.06 (ddd, *J*=8.8, 7.7, 2.8 Hz, 1H), 6.88 (dd, *J*=8.8, 2.8 Hz, 1H) and 2.35 (s, 3H). ^{13}C NMR (101 MHz, $CDCl_3$) δ_C 169.27, 169.26, 166.2 (d, *J*=257.8 Hz), 153.1 (d, *J*=11.9 Hz), 134.6 (d, *J*=10.4 Hz), 118.6 (d, *J*=3.6 Hz), 113.6 (d, *J*=21.4 Hz), 112.1 (d, *J*=24.0 Hz) and 20.9. ^{19}F{^{1}H} NMR (376 MHz, $CDCl_3$) δ_F −101.45. HRMS (ES−) *m/z* calcd for $C_9H_6FO_4$: 197.0256. Found $[M-H]^-$: 197.0256 (diff −0.37 ppm).

2-Acetoxy-5-fluorobenzoic acid, 6e[5]

Prepared according to general procedure 1.3.2 from 5-fluoro-2-hydroxybenzoic acid. Obtained a white crystalline solid (1.49 g, 75%). ^{1}H NMR (400 MHz, $CDCl_3$) δ_H 7.80 (1H, dd, *J*=8.6, 3.2 Hz), 7.32 (1H, ddd, *J*=7.2, 5.6, 3.2 Hz), 7.12 (1H, dd, *J*=8.8, 4.6 Hz) and 2.34 (3H, s). ^{13}C NMR (101 MHz, $CDCl_3$) δ_C 169.0, 168.6, 155.1 (d, *J*=250 Hz), 139.4 (d), 127.6 (d), 126.6 (d), 124.3, 121.6 (d) and 20.5. ^{19}F{^{1}H} NMR δ_F −115.0.

2-Acetoxy-6-fluorobenzoic acid, 6f[4]

Prepared according to general procedure 1.3.2 from 6-fluoro-2-hydroxybenzoic acid. Obtained a white crystalline solid (1.48 g, 71%). ^{1}H NMR (400 MHz, $CDCl_3$) δ_H 7.53 (1H, m), 7.09 (1H, m), 6.95 (1H, m) and 2.32 (3H, s). ^{13}C NMR (101 MHz, $CDCl_3$) δ_C 169.1, 167.2, 161.5 (d, *J*=260 Hz), 150.6, 133.4 (d), 119.4

(d), 114.5 (d), 114.0 (d) and 20.8. $^{19}F\{^{1}H\}$ NMR δ_F −108.3. Elemental analysis calcd (%) for $C_9H_7FO_4$: C, 54.6; H, 3.6; found: C, 54.6; H, 3.6.

2-Acetoxy-3-chlorobenzoic acid, 6g[6]

Prepared according to general procedure 1.3.1 from 3-chloro-2-hydroxybenzoic acid. Obtained a white crystalline solid (1.03 g, 83%). ^{1}H NMR (400 MHz, $CDCl_3$) δ_H 9.45 (1H, br s), 8.01 (1H, dd, *J*=8.0, 1.6 Hz), 7.69 (1H, dd, *J*=8.0, 1.6 Hz), 7.30 (1H, app. t, *J*= 8.0 Hz) and 2.40 (3H, s). ^{13}C NMR (101 MHz, $CDCl_3$) δ_C 169.25, 168.44, 147.71, 135.31, 130.82, 129.35, 126.64, 124.26 and 20.60. HRMS (ES−) *m/z* calcd for $C_9H_6ClO_4$: 212.9960. Found $[M{-}H]^-$ 212.9963 (diff −1.47 ppm).

2-Acetoxy-4-chlorobenzoic acid, 6h[6]

Prepared according to general procedure 1.3.1 from 4-chloro-2-hydroxybenzoic acid. Obtained an off-white solid (1.16 g, 92%). ^{1}H NMR (500 MHz, $CDCl_3$) δ_H 8.76 (1H, br s), 8.05 (1H, d, *J*=8.5 Hz), 7.34 (1H, dd, *J*=8.5, 2.0 Hz), 7.17 (1H, d, *J*=2.0 Hz) and 2.34 (3H, s). ^{13}C NMR (101 MHz, $CDCl_3$) δ_C 169.28, 168.74, 151.84, 140.72, 133.43, 126.63, 124.65, 120.72 and 20.92. HRMS (ES−) *m/z* calcd for $C_9H_6ClO_4$: 212.9960. Found $[M{-}H]^-$ 212.9957 (diff 1.54 ppm).

2-Acetoxy-5-chlorobenzoic acid, 6i[6]

Prepared according to general procedure 1.3.1 from 5-chloro-2-hydroxybenzoic acid. Obtained a white crystalline solid (1.17 g, 94%). ^{1}H NMR (400 MHz, $CDCl_3$) δ_H 8.08 (1H, d, *J*=2.6 Hz), 7.58 (1H, dd, *J*=8.6, 2.6 Hz), 7.09 (1H, d, *J*=8.6 Hz) and 2.34 (3H, s). ^{13}C NMR (101 MHz, $CDCl_3$) δ_C 169.50, 168.19, 149.71, 134.74, 132.24, 131.77, 125.48, 123.49 and 20.93. HRMS (ES−) *m/z* calcd for $C_9H_6ClO_4$: 212.9960. Found $[M{-}H]^-$ 212.9959 (diff 0.39 ppm).

2-Acetoxy-3,5-difluorobenzoic acid, 6j

Prepared according to general procedure 1.3.1 from 3,5-difluoro-2-hydroxybenzoic acid. Obtained a white crystalline solid (0.62 g, 98%). ^{1}H NMR (400 MHz, $CDCl_3$) δ_H 8.45 (1H, br s), 7.62 (1H, ddd, *J*=8.4, 3.0, 2.0 Hz), 7.21 (1H, ddd, *J*=9.3, 7.7, 3.0 Hz) and 2.40 (3H, s). $^{19}F\{^{1}H\}$ NMR (376 MHz, $CDCl_3$) δ_F −110.95 (1F, d, *J*=6.3 Hz) and −122.84 (1F, d, *J*=6.3 Hz). ^{13}C NMR (101 MHz, $CDCl_3$) δ_C 168.49, 167.85, 159.10 (dd, *J*=249.2, 11.1 Hz), 155.18 (dd, *J*=252.7, 12.1 Hz), 135.91 (dd, *J*=14.6, 4.6 Hz), 124.97-124.88 (m), 114.09 (dd, *J*=25.0, 3.6 Hz), 110.18 (dd, *J*=26.6, 22.9 Hz) and 20.42. HRMS (CI+) *m/z* calcd for $C_9H_{10}F_2NO_4$: 234.0572. Found $[M+NH_4]^+$ 234.0567 (diff 2.12 ppm).

2-Acetoxy-5-chloro-3-fluorobenzoic acid, 6k

Prepared according to general procedure from 1.3.1 from 5-chloro-3-fluoro-2-hydroxybenzoic acid. Obtained a white crystalline solid (1.17 g, 96%). ^{1}H NMR (400 MHz, $(CD_3)_2SO$) δ_H 13.78 (1H, br s), 7.92 (1H, dd, *J*=9.6 Hz, 2.5 Hz), 7.74 (1H, m), and 2.32 (3H, s). $^{19}F\{^{1}H\}$ NMR (376 MHz, $(CD_3)_2SO$) δ_F −126.11 (s). ^{13}C NMR (400 MHz, $(CD_3)_2SO$) δ_C 168.64, 164.13 (d, *J*=3.2 Hz), 154.82 (d, *J*=250.9 Hz), 137.35 (d, *J*=14.4 Hz), 130.90 (d, *J*=10.1 Hz), 128.10, 126.83 (d, *J*=3.3 Hz), 121.38 (d, *J*=22.8 Hz) and 20.70. HRMS (ES−) *m/z* calcd for $C_9H_5ClFO_4$: 230.9856. Found $[M{-}H]^-$ 230.9866 (diff 4.22 ppm).

2-Acetoxy-3-chloro-5-fluorobenzoic acid, 6l

Prepared according to general procedure 1.3.1 from 3-chloro-5-fluoro-2-hydroxybenzoic acid. Obtained a white crystalline solid (1.04 g, 60%). ^{1}H NMR (400 MHz, $(CD_3)_2SO$) δ_H 7.90 (1H, dd, *J*=7.9, 3.1 Hz), 7.69 (1H, dd, *J*=8.6, 3.1 Hz) and 2.32 (3H, s). ^{19}F{^{1}H} NMR (376 MHz, $(CD_3)_2SO$) δ_F −113.29 (s). ^{13}C NMR (101 MHz, $(CD_3)_2SO$) δ_C 168.59, 164.30 (d, *J*=2.1 Hz), 158.95 (d, *J*=247.2 Hz), 143.32 (d, *J*=3.7 Hz), 129.33 (d, *J*=10.9), 127.80 (d, *J*=8.2 Hz), 121.63 (d, *J*=26.5 Hz), 117.26 (d, *J*=24.5 Hz) and 20.76. HRMS (ES−) *m/z* calcd for $C_9H_5ClFO_4$: 230.9866. Found $[M-H]^-$ 230.9865 (diff 0.18 ppm).

3-Fluoro-2-(pivaloyloxy)benzoic acid, 7a

Prepared according to general procedure 1.3.3 from 3-fluoro-2-hydroxybenzoic acid. Obtained a white crystalline solid (2.2 g, 72%). ^{1}H NMR (400 MHz, $CDCl_3$) δ_H 7.90 (d, *J*=8.0 Hz, 1H), 7.42 (app. t, *J*=8.6 Hz, 1H), 7.29 (m, 1H) and 1.42 (s, 9H). ^{13}C NMR (101 MHz, $CDCl_3$) δ_C 175.7, 169.5 (d, *J*=3.4 Hz), 155.0 (d, *J*=250.4 Hz), 139.9 (d, *J*=14.5 Hz), 127.4 (d, *J*=3.6 Hz), 126.2 (d, *J*=7.6 Hz), 124.6, 121.6 (d, *J*=19.3 Hz), 39.2 and 27.1. ^{19}F{^{1}H} NMR (376 MHz, $CDCl_3$) δ_F −128.30. HRMS (ES−) *m/z* calcd for $C_{12}H_{12}FO_4$: 239.0725. Found $[M-H]^-$: 239.0723 (diff 0.82 ppm). MP 143-144 °C. Elemental analysis calcd (%) for $C_{12}H_{13}FO_4$: C, 60.00; H, 5.45 Found: C, 59.96; H, 5.47.

4-Fluoro-2-(pivaloyloxy)benzoic acid, 7b

Prepared according to general procedure 1.3.3 from 4-fluoro-2-hydroxybenzoic acid. Obtained a white crystalline solid (1.57 g, 51%). ^{1}H NMR (400 MHz, $CDCl_3$) δ_H 8.15 (dd, *J*=8.8, 6.4 Hz, 1H), 7.04 (m, 1H), 6.83 (dd, *J*=8.8, 2.4 Hz, 1H), 1.40 (s, 9H). ^{13}C NMR (101 MHz, $CDCl_3$) δ_C 176.4, 169.5, 166.1 (d, *J*=258.3 Hz), 153.5 (d, *J*=11.8 Hz), 134.5 (d, *J*=10.3 Hz), 118.9 (d, *J*=3.5 Hz), 113.3 (d, *J*=21.3 Hz), 111.9 (d, *J*=23.7 Hz), 39.1 and 27.1. ^{19}F{^{1}H} NMR (376 MHz, $CDCl_3$) δ_F −102.06. HRMS (ES−) *m/z* calcd for $C_{12}H_{12}FO_4$: 239.0725. Found $[M-H]^-$: 239.0729 (diff −1.47 ppm). MP 99-100 °C. Elemental analysis calcd (%) for $C_{12}H_{13}FO_4$: C, 60.00; H, 5.45 Found: C, 59.96; H, 5.41.

5-Fluoro-2-(pivaloyloxy)benzoic acid, 7c

Prepared according to general procedure 1.3.3 from 5-fluoro-2-hydroxybenzoic acid. Obtained a white crystalline solid (1.64 g, 53%). ^{1}H NMR (400 MHz, $CDCl_3$) δ_H 7.79 (dd, *J*=8.8, 3.2 Hz, 1H), 7.31 (m, 1H), 7.05 (dd, *J*=8.8, 4.4 Hz, 1H) and 1.37 (s, 9H). ^{13}C NMR (101 MHz, $CDCl_3$) δ_C 176.8, 168.9 (d, *J*=2.0 Hz), 159.6 (d, *J*=247.1 Hz), 147.5 (d, *J*=3.0 Hz), 125.5 (d, *J*=8.0 Hz), 123.8 (d, *J*=7.7 Hz), 121.6 (d, *J*=23.3 Hz), 118.8 (d, *J*=25.3 Hz), 39.1 and 27.1. ^{19}F{^{1}H} NMR (376 MHz, $CDCl_3$) δ_F −115.52. HRMS (ES−) *m/z* calcd for $C_{12}H_{12}FO_4$: 239.0725. Found $[M-H]^-$: 239.0725 (diff −0.08 ppm). MP 138-139 °C. Elemental analysis calcd (%) for $C_{12}H_{13}FO_4$: C, 60.00; H, 5.45 Found: C, 59.98; H, 5.37.

6-Fluoro-2-(pivaloyloxy)benzoic acid, 7d

Prepared according to general procedure 1.3.3 from 6-fluoro-2-hydroxybenzoic acid. Obtained a white crystalline solid (1.12 g, 36%). ^{1}H NMR (400 MHz, $CDCl_3$) δ_H 7.49 (app. Td, *J*=8.4, 6.0 Hz, 1H), 7.07 (app. T, *J*=8.4 Hz, 1H), 6.93 (d, *J*=8.4 Hz, 1H) and 1.35 (s, 9H). ^{13}C NMR (101 MHz, $CDCl_3$) δ_C 176.4, 167.4, 161.2 (d, *J*=258.8 Hz), 150.7 (d, *J*=4.4 Hz), 133.1 (d, *J*=10.4 Hz), 119.1 (d, *J*=3.5 Hz), 114.3 (d, *J*=15.5 Hz), 113.9 (d, *J*=22.3 Hz), 39.1 and 27.0. ^{19}F{^{1}H} NMR (376 MHz, $CDCl_3$) δ_F −109.22. HRMS (ES−) *m/z* calcd for

$C_{12}H_{12}FO_4$: 239.0725. Found [M−H]$^-$: 239.0715 (diff 4.15ppm). MP 88-89 °C. Elemental analysis calcd (%) for $C_{12}H_{13}FO_4$: C, 60.00; H, 5.45 Found: C, 59.96; H, 5.43.

4-Chloro-2-(pivaloyloxy)benzoic acid, 7e

Prepared according to general procedure 1.3.3 from 4-chloro-2-hydroxybenzoic acid. Obtained a white crystalline solid (1.53 g, 51%). ^{1}H NMR (400 MHz, $CDCl_3$) δ_H 8.02 (1H, d, *J*=8.5 Hz), 7.32 (1H, dd, *J*=8.5, 2.0 Hz), 7.11 (1H, d, *J*=2.0 Hz) and 1.37 (9H, s). ^{13}C NMR (101 MHz, $CDCl_3$) δ_C 177.16, 170.08, 152.97, 141.26, 134.15, 127.09, 125.22, 121.85, 39.84 and 27.86. HRMS (ES−) calcd for $C_{12}H_{12}ClO_4$: 255.0430. Found [M−H]$^-$ 255.0427 (diff 0.91 ppm).

5-Chloro-2-(pivaloyloxy)benzoic acid, 7f

Prepared according to general procedure 1.3.3 from 5-chloro-2-hydroxybenzoic acid. Obtained a white crystalline solid (1.83 g, 61%). ^{1}H NMR (400 MHz, $CDCl_3$) δ_H 8.07 (1H, d, *J*=2.6 Hz), 7.56 (1H, dd, *J*=8.6, 2.6 Hz), 7.03 (1H, d, *J*=8.6 Hz) and 1.37 (9H, s). ^{13}C NMR (101 MHz, $CDCl_3$) δ_C 176.63, 168.65, 150.08, 134.57, 132.14, 131.46, 125.38, 123.84, 39.10 and 27.13. HRMS (ES−) calcd for $C_{12}H_{12}ClO_4$: 255.0430. Found [M−H]$^-$ 255.0428 (diff 0.68 ppm).

3,5-Difluoro-2-(pivaloyloxy)benzoic acid, 7g

Prepared according to general procedure 1.3.3 from 3,5-difluoro-2-hydroxybenzoic acid. Obtained a white crystalline solid (1.69 g, 56% yield). ^{1}H NMR (500 MHz, $(CD_3)_2SO$) δ_H 13.70 (1H, br s), 7.74 (1H, ddd, *J*=10.1, 8.4, 3.0 Hz), 7.53 (1H, ddd, *J*=8.7, 3.0, 1.8 Hz) and 1.30 (9H, s). ^{13}C NMR (126 MHz, $(CD_3)_2SO$) δ_C 175.18, 163.96-163.92 (m), 158.49 (dd, *J*=246.0, 11.9 Hz), 154.3 (dd, *J*=249.2, 12.8 Hz), 134.63 (dd, *J*=14.3, 4.1 Hz), 127.71 (app. D, *J*=8.1 Hz), 113.09 (dd, *J*=24.6, 3.4 Hz), 108.95 (dd, *J*=27.4, 23.6 Hz), 38.58 and 26.71. ^{19}F{^{1}H} NMR (376 MHz, $(CD_3)_2SO$) δ_F −111.93 (1F, d, *J*=5.8 Hz) and -124.92 (1F, d, *J*=5.8 Hz). HRMS (ES−) calcd for $C_{12}H_{11}F_2O_4$: 257.0631. Found [M−H]$^-$ 257.0636 (diff −1.91 ppm).

3,5-Dichloro-2-(pivaloyloxy)benzoic acid, 7h

Prepared according to general procedure 1.3.3 from 3,5-dichloro-2-hydroxybenzoic acid. Obtained a white crystalline solid (0.95 g, 68%).^{1}H NMR (400 MHz, $CDCl_3$) δ_H 7.98 (m, 1H), 7.68 (m, 1H) and 1.40 (s, 9H). ^{13}C NMR (101 MHz, $CDCl_3$) δ_C 175.4, 168.1, 146.8, 134.8, 131.6, 130.7, 130.3, 125.3, 39.4 and 27.2. HRMS (ES−) *m/z* calcd for $C_{12}H_{11}Cl_2O_4$: 289.0040. Found [M−H]$^-$: 289.0040 (diff 0.1 ppm). MP 164-165 °C. Elemental analysis calcd (%) for $C_{12}H_{12}Cl_2O_4$: C, 49.51; H, 4.15 Found: C, 49.84; H, 4.08.

5-Chloro-3-fluoro-2-(pivaloyloxy)benzoic acid, 7i

Prepared according to general procedure 1.3.3 from **5l**. Obtained a white crystalline solid (1.51 g, 52% yield). ^{1}H NMR (500 MHz, $(CD_3)_2SO$) δ_H 13.76 (1H, br s), 7.90 (1H, dd, *J*=9.6, 2.4 Hz), 7.72 (1H, dd, *J*=2.4, 1.8 Hz) and 1.30 (9H, s). ^{13}C NMR (126 MHz, $(CD_3)_2SO$) δ_C 175.01, 163.78 (d, *J*=3.0 Hz), 154.23 (d, *J*=250.7 Hz), 137.05 (d, *J*=14.4 Hz), 130.35 (d, *J*=10.0 Hz), 128.01, 126.28 (d, *J*=3.3 Hz), 120.83 (d, *J*=22.8 Hz), 38.62 and 26.67. ^{19}F{^{1}H} NMR (376 MHz, $(CD_3)_2SO$) δ_F −126.33 (s). HRMS (ES−) calcd for $C_{12}H_{11}ClFO_4$: 273.0335. Found [M−H]$^-$ 273.0343 (diff −2.90 ppm).

3-Chloro-2-(isobutyryloxy)benzoic acid, 7j

Prepared according to general procedure 1.3.3 from 3-chloro-2-hydroxybenzoic acid. Obtained a white crystalline solid (1.02 g, 78% yield). ^{1}H NMR (400 MHz, $(CD_3)_2SO$) δ_H 13.42 (1H, br s), 7.88 (1H, app. d, *J*=7.9 Hz), 7.82 (1H, app. d, *J*=7.9 Hz), 7.41 (1H, app. t, *J*=7.9 Hz), 2.86 (1H, sept, *J*=7.0 Hz) and 1.26 (6H, d, *J*=7.0 Hz). ^{13}C NMR (101 MHz, $(CD_3)_2SO$) δ_C 174.11, 165.44, 146.64, 134.32, 130.65, 128.26, 127.61, 127.03, 33.85, and 19.00. HRMS (ES−) calcd for $C_{11}H_{10}ClO_4$: 241.0273. Found $[M-H]^-$ 241.0271 (diff 0.70 ppm).

3-chloro-5-fluoro-2-(isobutyryloxy)benzoic acid, 7k

Prepared according to general procedure 1.3.3 from **5l**. Recrystallisation from hexane afforded **7k** as a white crystalline solid (1.26 g, 61% yield). ^{1}H NMR (400 MHz, $(CD_3)_2SO$) δ_H 13.75 (1H, br s), 7.90 (1H, dd, *J*=7.9, 3.0 Hz), 7.68 (1H, dd, *J*=8.6, 3.0 Hz), 2.86 (1H, sept, *J*=7.0 Hz) and 1.26 (1H, d, *J*=7.0 Hz). ^{13}C NMR (126 MHz, $(CD_3)_2SO$) δ_C 173.67, 163.94 (d, *J*=2.3 Hz), 158.49 (d, *J*=247.1 Hz), 142.76 (d, *J*=3.5 Hz), 128.86 (d, *J*=10.9 Hz), 127.58 (d, *J*=8.1 Hz), 121.17 (d, *J*=26.7 Hz), 116.78 (d, *J*=24.5 Hz), 33.36 and 18.52. ^{19}F{^{1}H} NMR (376 MHz, $(CD_3)_2SO$) δ_F −113.41 (s). HRMS (ES−) calcd for $C_{11}H_9ClFO_4$: 259.0179. Found $[M-H]^-$ 259.0180 (diff −0.46 ppm).

1.4 General Procedures for Synthesis of *O*-Acyl Thiazolides, 8a-m, 9a-k

1.4.1 HATU/DIPEA Amide Coupling

To a solution of the appropriate *O*-acetyl salicylic acid (1.0 eq.) in anh. DCM (1.1 mL $mmol^{-1}$) was added HATU (1.5 eq.) and DIPEA (1.0 eq.) under N_2 at room temperature. The reaction was allowed to stir for 30 minutes, then 2-amino-5-chlorothiazole hydrochloride (1.0 eq.) and DIPEA (1.0 eq.) were added and stirring continued overnight. The reaction mixture was partitioned between DCM and water, and the aqueous phase was extracted with DCM (3 x 5 volumes) before the combined organic phases were dried over $MgSO_4$, filtered, and concentrated *in vacuo* to afford the crude product. Purification *via* flash column chromatography eluting with a mixture of ethyl acetate and *n*-hexane afforded the desired product.

1.4.2 Schotten-Baumann Amide Coupling

To a suspension of the appropriate *O*-acetyl salicylic acid (1.0 eq) in anh. DCM (4.2 mL $mmol^{-1}$) under N_2 was added oxalyl chloride (1.2 eq) and anh. DMF (3 drops). The reaction was allowed to stir for 1 hr at room temperature before the volatiles were removed *in vacuo*. The residue was dissolved in ethyl acetate (0.5 volumes) and added dropwise to a vigorously stirred mixture of 2-amino-5-chlorothiazole hydrochloride (1.0 eq), $NaHCO_3$ (4.0 eq), water (1 volume) and ethyl acetate (0.5 volumes). The resulting biphasic mixture was then allowed to stir overnight at room temperature. The organic and aqueous phases were then separated, and the aqueous phase extracted with ethyl acetate (2 x 5 volumes). The combined organics were then washed with HCl (0.5 M, 2 x 5 volumes) and brine (5 volumes), then dried over $MgSO_4$ and filtered. The solvent was then removed *in vacuo* to afford the desired product.

1.4.3 Anhydrous Amide Coupling

To a solution of the appropriate *O*-pivaloyl or *O*-isobutyryl salicylic acid (1.0 eq) in anh. DCM (3.8 mL $mmol^{-1}$) under N_2 was added thionyl chloride (1.1 eq) and anh. pyridine (2-3 drops). The reaction was heated to reflux and allowed to stir for 1.5 hours. After cooling to room temperature, the volatiles were removed *in vacuo* and the residue was dissolved in anh. THF (0.3 volumes). The resulting mixture was

added dropwise to a stirred solution of the relevant 2-aminothiazole (1.0 eq) and anh. triethylamine (1.0 eq) in anh. THF (0.6 volumes) under N_2. The reaction was allowed to stir overnight at room temperature, then diluted with ethyl acetate (6 volumes) and washed successively with HCl (2 M, 2 x 3 volumes), saturated $NaHCO_3$ (3 volumes) and brine (3 volumes). The organics were dried over $MgSO_4$, filtered and then the solvent was removed *in vacuo*. Purification *via* flash chromatography, eluting with a mixture of ethyl acetate and *n*-hexane, afforded the desired product.

2-((5-Chlorothiazol-2-yl)carbamoyl)-6-methylphenyl acetate, 8a[7]

Prepared according to general procedure 1.4.1 from **6a**. Obtained a yellow solid (0.31 g, 45%). ^{1}H NMR (400 MHz, $CDCl_3$) δ_H 11.53 (br s, 1H), 7.62 (d, *J*=7.6 Hz, 1H), 7.49 (d, *J*=7.6 Hz, 1H), 7.29 (app. t, *J*=7.6 Hz, 1H), 6.78 (s, 1H), 2.35 (s, 3H) and 2.27 (s, 3H). ^{13}C NMR (101 MHz, $CDCl_3$) δ_C 168.7, 163.8, 156.8, 147.4, 135.1, 134.5, 132.5, 127.2, 126.3, 126.2, 120.9, 20.7 and 16.3. HRMS (ES+) m/z calcd for $C_{13}H_{11}ClN_2NaO_3S$: 333.0071. Found $[M+Na]^+$: 333.0073 (diff −0.58 ppm). MP 134–136 °C. Elemental analysis calcd (%) for $C_{13}H_{11}ClN_2O_3S$: C, 50.52; H, 3.57; N, 9.01; S, 10.32 Found: C, 50.13; H, 3.74; N, 9.12; S, 10.37.

2-((5-Chlorothiazol-2-yl)carbamoyl)-6-isopropylphenyl acetate, 8b

Prepared according to general procedure 1.4.1 from **6b**. Obtained a yellow solid (0.56 g, 43%). ^{1}H NMR (400 MHz, $CDCl_3$) δ_H 11.59 (br s, 1H), 7.63–7.54 (m, 2H), 7.36 (t, *J*=7.8 Hz, 1H), 6.67 (s, 1H), 3.10 (sept, *J*=7.0 Hz, 1H), 2.34 (s, 3H) and 1.26 (d, *J*=7.0 Hz, 6H). ^{13}C NMR (101 MHz, CDCl3) δ_C 169.3, 164.2, 156.8, 146.1, 142.6, 134.5, 130.8, 126.8, 126.58, 126.55, 120.8, 27.4, 23.0 and 20.8. HRMS (ES+) m/z calcd for $C_{15}H_{15}ClN_2NaO_3S$: 361.0384. Found $[M+Na]^+$: 361.0386 (diff −0.62 ppm). MP 117–119 °C. Elemental analysis calcd (%) for $C_{15}H_{15}ClN_2O_3S$: C, 53.18; H, 4.46; N, 8.27; S, 9.46 Found: C, 53.04; H, 4.54; N, 8.36; S, 9.42.O.

2-((5-Chlorothiazol-2-yl)carbamoyl)-6-fluorophenyl acetate, 8c

Prepared according to general procedure 1.4.2 from **6c**. Obtained an off-white solid (0.185 g, 59%). ^{1}H NMR (400 MHz, $CDCl_3$) δ_H 11.58 (br s, 1H), 7.63 (d, 1H), 7.39 (m, 2H), 6.80 (s, 1H) and 2.39 (s, 3H). ^{13}C NMR (101 MHz, $CDCl_3$) δ_C 167.5, 162.2 (d), 156.4, 154.8 (d, *J*=250 Hz), 136.9 (d), 134.5, 127.9, 127.2 (d), 124.8 (d), 121.4, 120.6 (d) and 20.4. ^{19}F{^{1}H} NMR (376 MHz, CDCl3) δ_F −125.5. HRMS (ES+) *m/z* Calcd for $C_{12}H_9ClFN_2O_3S$: 315.0001. Found $[M+H]^+$: 314.9998. Elemental analysis calcd (%) for $C_{12}H_8ClFN_2O_3S$: C, 45.8; H, 2.6; N, 8.9. Found: C, 45.8; H, 2.5; N, 8.7.

2-((5-Chlorothiazol-2-yl)carbamoyl)-5-fluorophenyl acetate, 8d

Prepared according to general procedure 1.4.2 from **6d**. Obtained a white solid (1.09 g, 69%). Unable to be isolated, used crude without purification.

2-((5-Chlorothiazol-2-yl)carbamoyl)-4-fluorophenyl acetate, 8e

Prepared according to general procedure 1.4.2 from **6e**. Obtained an off-white solid (0.146 g, 62%). ^{1}H NMR (400 MHz, $CDCl_3$) δ_H 11.12 (br m, 1H), 7.63 (m, 1H), 7.33 (m, 1H), 7.25 (m, 1H), 6.96 (s, 1H) and 2.38 (s, 3H). ^{13}C NMR (101 MHz, $CDCl_3$) δ_C 168.5, 161.7, 160.0 (d, *J*=254 Hz), 156.0, 144.4 (d, *J*=3 Hz), 134.7, 126.6 (d, *J*=8 Hz), 125.5 (d, *J*=8 Hz), 121.6, 120.4 (d, *J*=23 Hz), 117.0 (d, *J*=25 Hz) and 21.0. ^{19}F{^{1}H} NMR (376 MHz, $CDCl_3$) δ_F −114.0. HRMS (ES+) *m/z* Calcd for $C_{12}H_9ClFN_2O_3S$: 315.0001. Found $[M+H]^+$:

315.0015. Elemental analysis calcd (%) for $C_{12}H_8ClFN_2O_3S$: C, 45.8; H, 2.6; N, 8.9. Found: C, 45.7; H, 2.55; N, 8.85.

2-((5-Chlorothiazol-2-yl)carbamoyl)-3-fluorophenyl acetate, 8f

Prepared according to general procedure 1.4.2 from **6f**. Obtained an off-white solid (0.168 g, 53%). ^{1}H NMR (400 MHz, $CDCl_3$) δ_H 12.25 (br s, 1H), 7.58 (m, 1H), 7.10 (m, 2H), 6.48 (s, 1H) and 2.26 (s, 3H). ^{13}C NMR (101 MHz, $CDCl_3$) δ_C 168.8, 159.8, 159.7 (d, *J*=268 Hz), 156.7, 149.9, 134.0, 132.8, 121.2 (d), 119.6 (d), 116.7 (d), 113.7 (d) and 20.9. ^{19}F NMR (376 MHz, $CDCl_3$) δ_F −111.9. HRMS (ES+) *m/z* Calcd for $C_{12}H_9ClFN_2O_3S$: 315.0001. Found $[M+H]^+$: 315.0011. Elemental analysis calcd (%) for $C_{12}H_8ClFN_2O_3S$: C, 45.8; H, 2.6; N, 8.9. Found: C, 45.7; H, 2.6; N, 8.7.

2-Chloro-6-((5-chlorothiazol-2-yl)carbamoyl)phenyl acetate, 8g

Prepared according to general procedure 1.4.2 from **6g**. Obtained a brown solid (0.32 g, 83% yield). ^{1}H NMR (400 MHz, $CDCl_3$) δ_H 12.05 (1H, br s), 7.71-7.66 (2H, m), 7.34 (1H, app. t, *J*=7.9 Hz), 6.71 (1H, s) and 2.37 (3H, s). ^{13}C NMR (101 MHz, $CDCl_3$) δ_C 167.88, 162.78, 156.95, 145.48, 134.26, 133.90, 129.29, 128.34, 127.79, 127.06, 121.33 and 20.45. HRMS (ES+) calcd for $C_{12}H_9Cl_2N_2O_3S$: 330.9705. Found $[M+H]^+$ 330.9708 (diff −0.73 ppm).

5-Chloro-2-((5-chlorothiazol-2-yl)carbamoyl)phenyl acetate, 8h

Prepared according to general procedure 1.4.2 from **6h**. Obtained as **8h** an off-white solid (0.35 g, 91%). ^{1}H NMR (400 MHz, $CDCl_3$) δ_H 11.23 (br s, 1H), 7.86 (d, *J*=8.4 Hz, 1H), 7.38 (dd, *J*=8.4, 2.0 Hz, 1H), 7.30 (d, *J*=2.0 Hz, 1H), 7.03 (s, 1H) and 2.39 (s, 3H). ^{13}C NMR (101 MHz, $CDCl_3$) δ_C 168.07, 162.10, 156.17, 149.11, 139.30, 134.67, 131.17, 126.86, 124.29, 123.60, 121.47 and 21.02. HRMS (ES+) *m/z* calcd for $C_{12}H_9Cl_2N_2O_3S$: 330.9705. Found $[M+H]^+$: 330.9708 (diff −0.91 ppm).

4-Chloro-2-((5-chlorothiazol-2-yl)carbamoyl)phenyl acetate, 8i

Prepared according to general procedure 1.4.2 from **6i**. Obtained a pale orange solid (0.35 g, 90%). ^{1}H NMR (400 MHz, $CDCl_3$) δ_H 11.32 (br s, 1H), 7.86 (d, *J*=2.6 Hz, 1H), 7.57 (dd, *J*=8.7, 2.6 Hz, 1H), 7.21 (d, *J*=8.7 Hz, 1H), 6.98 (s, 1H) and 2.37 (s, 3H). ^{13}C NMR (101 MHz, $CDCl_3$) δ_C 168.36, 161.82, 156.22, 147.06, 134.67, 133.30, 132.13, 129.96, 126.64, 125.20, 121.58 and 21.00. HRMS (ES+) *m/z* calcd for $C_{12}H_9Cl_2N_2O_3S$: 330.9705. Found $[M+H]^+$: 330.9707 (diff −0.49 ppm).

2-((5-Chlorothiazol-2-yl)carbamoyl)-4,6-difluorophenyl acetate, 8j

Prepared according to general procedure 1.4.2 from **6j**. Obtained an off white solid (0.63 g, 82%). ^{1}H NMR (400 MHz, $(CD_3)_2SO$) δ_H 13.08 (br s, 1H), 7.76 (ddd, *J*=10.3, 8.6, 2.9 Hz, 1H), 7.63–7.59 (m, 2H) and 2.30 (s, 3H). ^{13}C NMR (101 MHz, $(CD_3)_2SO$) δ_C 168.43, 162.68, 158.95 (dd, *J*=246.4, 11.6 Hz), 156.41, 154.49 (dd, *J*=250.1, 13.1 Hz), 136.13, 133.42 (dd, *J*=14.5, 4.2 Hz), 129.59 (app. d, *J*=8.7 Hz), 119.37, 112.79 (dd, *J*=25.2, 3.5 Hz), 108.67 (dd, *J*=27.2, 23.4 Hz) and 20.47. $^{19}F\{^{1}H\}$ NMR (376 MHz, $(CD_3)_2SO$) δ_F −111.50 (d, *J*=5.8 Hz, 1F) and −122.84 (app. s, 1F). HRMS (ES+) *m/z* calcd for $C_{12}H_8ClF_2N_2O_3S$: 332.9907. Found $[M+H]^+$: 332.9907 (diff −0.13 ppm).

4-Chloro-2-((5-chlorothiazol-2-yl)carbamoyl)-6-fluorophenyl acetate, 8k

Prepared according to general procedure 1.4.2 from **6k**. Obtained an off-white solid (0.24 g, 64%).^{1}H NMR (400 MHz, $(CD_3)_2SO$) δ_H 13.09 (br s, 1H), 7.92 (dd, *J*=9.8, 2.1 Hz, 1H), 7.79 (app. s, 1H), 7.62 (s, 1H) and 2.31 (s, 3H). ^{13}C NMR (101 MHz, $(CD_3)_2SO$) δ_C 168.27, 162.55, 154.38 (d, *J*=251.42 Hz), 136.10, 135.84 (d, *J*=14.59 Hz), 130.94 (d, *J*=9.9 Hz), 125.76 (d, *J*=3.3 Hz), 124.89 (d, *J*=3.5 Hz), 120.61 (d, *J*=22.6 Hz), 120.52 (d, *J*=22.1 Hz), 119.35 and 20.49. ^{19}F{^{1}H} NMR (376 MHz, $(CD_3)_2SO$) δ_F −125.17 (s). HRMS (ES+) *m/z* calcd for $C_{12}H_7ClF_2N_2NaO_3S$: 370.9431. Found $[M+Na]^+$: 370.9430 (diff 0.28 ppm).

2-Chloro-6-((5-chlorothiazol-2-yl)carbamoyl)-4-fluorophenyl acetate, 8l

Prepared according to general procedure 1.4.2 from **6l**. Obtained an off-white solid (0.38 g, 99%).^{1}H NMR (400 MHz, $(CD_3)_2SO$) δ_H 13.07 (s, 1H), 7.90 (dd, *J*=8.1, 2.8 Hz, 1H), 7.75 (dd, *J*=8.4, 2.8 Hz, 1H), 7.63 (s, 1H) and 2.30 (s, 3H). ^{13}C NMR (101 MHz, $(CD_3)_2SO$) δ_C 168.29, 162.84, 158.87 (d, *J*=247.5 Hz), 156.28, 141.90 (d, *J*=3.6 Hz), 136.16, 129.72 (d, *J*=8.4 Hz), 128.87 (d, *J*=11.3 Hz), 120.67 (d, *J*=26.5 Hz), 119.40, 116.31 (d, *J*=25.3 Hz) and 20.54. ^{19}F{^{1}H} NMR (376 MHz, $(CD_3)_2SO$) δ_F −113.12 (s). HRMS (ES+) *m/z* calcd for $C_{12}H_7ClF_2N_2NaO_3S$: 370.9431. Found $[M+Na]^+$: 370.9430 (diff 0.28 ppm).

2,4-Dichloro-6-((5-chlorothiazol-2-yl)carbamoyl)phenyl pivalate, 8m

Prepared according to general procedure 1.4.3 from **7h**. Obtained a white solid (0.69 g, 62%).^{1}H NMR (400 MHz, $CDCl_3$) δ_H 7.67 (s, 2H), 6.89 (s, 1H) and 1.37 (s, 9H). ^{13}C NMR (101 MHz, $CDCl_3$) δ_C 175.3, 161.3, 156.2, 144.2, 134.4, 133.4, 132.3, 130.1, 129.4, 128.0, 121.8, 39.5 and 27.1. HRMS (ES+) *m/z* calcd for $C_{15}H_{13}Cl_3N_2NaO_3S$: 428.9605. Found $[M+Na]^+$: 428.9602 (diff 0.73 ppm). MP 152–153 °C. Elemental analysis calcd (%) for $C_{15}H_{13}Cl_3N_2O_2S$: C, 44.19; H, 3.21; N, 6.87; S, 7.86. Found: C, 44.11; H, 3.33; N, 7.06; S, 7.72.

2-Fluoro-6-((5-nitrothiazol-2-yl)carbamoyl)phenyl pivalate, 9a

Prepared according to general procedure 1.4.3 from **7a**. Obtained a beige solid (0.98 g, 54%).^{1}H NMR (400 MHz, $CDCl_3$) δ_H 10.47 (br s, 1H), 8.20 (s, 1H), 7.83 (d, *J*=7.6 Hz, 1H), 7.43 (m, 2H) and 1.45 (s, 9H). ^{13}C NMR (101 MHz, $CDCl_3$) δ_C 175.0, 161.9 (d, *J*=3.1 Hz), 160.4, 154.5 (d, *J*=252.9 Hz), 144.0, 140.6, 137.5 (d, *J*=14.7 Hz), 127.4 (d, *J*=7.7 Hz), 126.1, 125.9 (d, *J*=3.6 Hz), 121.5 (d, *J*=19.3 Hz), 39.6 and 27.1. ^{19}F{^{1}H} NMR (376 MHz, $CDCl_3$) δ_F −125.27. HRMS (ES−) *m/z* calcd for $C_{15}H_{13}FN_3O_5S$: 366.0565. Found $[M-H]^-$: 366.0578 (diff −3.51 ppm). MP 191–192 °C. Elemental analysis calcd (%) for $C_{15}H_{14}FN_3O_5S$: C, 49.04; H, 3.84; N, 11.44. Found: C, 49.05; H, 3.87; N, 11.19.

5-Fluoro-2-((5-nitrothiazol-2-yl)carbamoyl)phenyl pivalate, 9b

Prepared according to general procedure 1.4.3 from **7b**. Obtained a yellow solid (0.68 g, 37%).^{1}H NMR (400 MHz, $(CD_3)_2SO$) δ_H 13.61 (br s, 1H), 8.70 (s, 1H), 7.91 (m, 1H), 7.34 (m, 2H) and 1.26 (s, 9H). ^{13}C NMR (101 MHz, $(CD_3)_2SO$) δ_C 175.9, 165.0, 164.5 (d, *J*=252.6 Hz), 162.2, 150.8 (d, *J*=12.1 Hz), 143.0, 142.6, 132.2 (d, *J*=10.4 Hz), 123.2 (d, *J*=3.4 Hz), 113.6 (d, *J*=21.8 Hz), 111.8 (d, *J*=25.0 Hz), 39.1 and 27.1. ^{19}F{^{1}H} NMR (376 MHz, $(CD_3)_2SO$) δ_F −105.34. HRMS (ES+) *m/z* calcd for $C_{15}H_{14}FN_3NaO_5S$: 390.0530. Found $[M+Na]^+$: 390.0526 (diff 1.25 ppm). MP 147–148 °C. Elemental analysis calcd (%) for $C_{15}H_{14}FN_3O_5S$: C, 49.04; H, 3.84; N, 11.44. Found: C, 49.16; H, 3.83; N, 11.34.

4-Fluoro-2-((5-nitrothiazol-2-yl)carbamoyl)phenyl pivalate, 9c

Prepared according to general procedure 1.4.3 from **7c**. Obtained an orange solid (1.43 g, 78%).^{1}H NMR (400 MHz, $(CD_3)_2SO$) δ_H 13.68 (br s, 1H), 8.71 (s, 1H), 7.73 (dd, *J*=8.8, 3.0 Hz, 1H), 7.55 (app. td, *J*=8.8, 3.0 Hz, 1H), 7.37 (dd, *J*=8.8, 4.6 Hz, 1H) and 1.25 (s, 9H). ^{13}C NMR (101 MHz, $(CD_3)_2SO$) δ_C 176.3, 164.6, 162.0, 159.2 (d, *J*=244.7 Hz), 145.1 (d, *J*=2.8 Hz), 143.0, 142.7, 127.9 (d, *J*=7.8 Hz), 125.7 (d, *J*=8.5 Hz), 120.3 (d, *J*=23.3 Hz), 116.9 (d, *J*=25.9 Hz), 39.0 and 27.1. ^{19}F{^{1}H} NMR (376 MHz, $(CD_3)_2SO$) δ_F −115.88. HRMS (ES+) *m/z* calcd for $C_{15}H_{14}FN_3NaO_5S$: 390.0530. Found $[M+Na]^+$: 390.0530 (diff 0.15 ppm). MP 158–159 °C. Elemental analysis calcd (%) for $C_{15}H_{14}FN_3O_5S$: C, 49.04; H, 3.84; N, 11.44. Found: C, 48.99; H, 3.78; N, 11.52.

3-Fluoro-2-((5-nitrothiazol-2-yl)carbamoyl)phenyl pivalate, 9d

Prepared according to general procedure 1.4.3 from **7d**. Obtained a brown solid (0.56 g, 37%).^{1}H NMR (400 MHz, $(CD_3)_2SO$) δ_H 13.92 (br s, 1H), 8.70 (s, 1H), 7.69 (m, 1H), 7.37 (app. t, *J*=8.3 Hz, 1H), 7.22 (d, *J*=8.3 Hz, 1H) and 1.20 (s, 9H). ^{13}C NMR (101 MHz, $(CD_3)_2SO$) δ_C 175.8, 161.8, 161.3, 159.3 (d, *J*=250.8 Hz), 149.2 (d, *J*=5.6 Hz), 143.0, 142.9, 133.6 (d, *J*=9.8 Hz), 119.8 (d, *J*=3.2 Hz), 116.9 (d, *J*=19.3 Hz), 114.1 (d, *J*=21.1 Hz), 39.0 and 27.0. ^{19}F{^{1}H} NMR (376 MHz, $(CD_3)_2SO$) δ_F −113.17. HRMS (ES+) *m/z* calcd for $C_{15}H_{14}FN_3NaO_5S$: 390.0530. Found $[M+Na]^+$: 390.0528 (diff 0.57 ppm). MP 154–155 °C. Elemental analysis calcd (%) for $C_{15}H_{14}FN_3O_5S$: C, 49.04; H, 3.84; N, 11.44. Found: C, 48.97; H, 3.81; N, 11.34.

5-Chloro-2-((5-nitrothiazol-2-yl)carbamoyl)phenyl pivalate, 9e

Prepared according to general procedure 1.4.3 from **7e**. Obtained a yellow solid (0.75 g, 68%).^{1}H NMR (500 MHz, $(CD_3)_2SO$) δ_H 13.67 (br s, 1H), 8.70 (s, 1H), 7.85 (d, *J*=8.8 Hz, 1H), 7.57–7.55 (m, 2H) and 1.26 (s, 9H). ^{13}C NMR (126 MHz, $(CD_3)_2SO$) δ_C 176.01, 164.96, 162.09, 149.89, 142.98, 142.67, 137.60, 131.53, 126.55, 125.50, 124.12, 39.05 and 27.09. HRMS (ES+) *m/z* calcd for $C_{15}H_{14}ClN_3NaO_5S$: 406.0235. Found $[M+Na]^+$: 406.0232 (diff 0.71 ppm).

4-Chloro-2-((5-nitrothiazol-2-yl)carbamoyl)phenyl pivalate, 9f

Prepared according to general procedure 1.4.3 from **7f**. Obtained an orange solid (0.63 g, 42%).^{1}H NMR (500 MHz, $(CD_3)_2SO$) δ_H 13.96 (br s, 1H), 8.71 (s, 1H), 7.93 (d, *J*=2.6 Hz, 1H), 7.75 (dd, *J*=8.7, 2.6 Hz, 1H), 7.36 (d, *J*=8.7 Hz, 1H) and 1.25 (s, 9H). ^{13}C NMR (126 MHz, $(CD_3)_2SO$) δ_C 176.15, 164.50, 162.00, 147.81, 142.99, 142.72, 133.31, 130.39, 129.84, 128.08, 125.68, 39.03 and 27.11. HRMS (ES+) *m/z* calcd for $C_{15}H_{14}ClN_3NaO_5S$: 406.0235. Found $[M+Na]^+$: 406.0238 (diff −0.86 ppm).

2,4-Difluoro-6-((5-nitrothiazol-2-yl)carbamoyl)phenyl pivalate, 9g

Prepared according to general procedure 1.4.3 from **7g**. Obtained a pale orange solid (0.81 g, 54%).^{1}H NMR (500 MHz, $(CD_3)_2SO$) δ_H 13.79 (s, 1H), 8.71 (s, 1H), 7.80 (ddd, *J*=10.3, 8.6, 2.8 Hz, 1H), 7.65 (ddd, *J*=8.4, 2.8, 1.7 Hz, 1H) and 1.26 (s, 9H). ^{13}C NMR (126 MHz, $(CD_3)_2SO$) δ_C 174.89, 163.11, 161.37, 158.52 (dd, *J*=246.50, 11.58 Hz), 153.89 (dd, *J*=250.41, 12.9 Hz), 142.41, 142.38, 133.11 (dd, *J*=14.7, 4.2 Hz), 128.80 (app. d, *J*=9.5 Hz), 112.37 (dd, *J*=25.5, 3.3 Hz), 108.65 (dd, *J*=27.4, 23.3 Hz), 38.65 and 26.57. ^{19}F{^{1}H} NMR (376 MHz, $(CD_3)_2SO$) δ_F −111.31 (d, *J*=6.1 Hz, 1F) and 123.83 (d, *J*=6.1 Hz, 1F). HRMS (ES+) *m/z* calcd for $C_{15}H_{12}F_2N_3O_5S$: 384.0471. Found $[M-H]^-$: 384.0482 (diff −2.76 ppm).

2,4-Dichloro-6-((5-nitrothiazol-2-yl)carbamoyl)phenyl pivalate, 9h

Prepared according to general procedure 1.4.3 from **7h**. Obtained a pale yellow solid (1.0 g, 79%).^{1}H NMR (500 MHz, $(CD_3)_2SO$) δ_H 13.79 (br s, 1H), 8.71 (s, 1H), 8.09 (d, *J*=2.5 Hz, 1H), 7.96 (d, *J*=2.5 Hz, 1H) and 1.28 (s, 9H). ^{13}C NMR (126 MHz, $(CD_3)_2SO$) δ_C 175.0, 163.7, 161.9, 144.3, 142.9, 142.8, 133.2, 131.2, 129.7, 129.04, 129.00, 39.2 and 27.1. HRMS (ES+) *m/z* calcd for $C_{15}H_{13}Cl_2N_3NaO_5S$: 439.9845. Found $[M+Na]^+$: 439.9836 (diff 2.11 ppm). MP 198–199 °C. Elemental analysis calcd (%) for $C_{15}H_{13}Cl_2N_3O_5S$: C, 43.08; H, 3.13; N, 10.05; S, 7.67. Found: C, 43.00; H, 3.30; N, 9.80; S, 7.32.

4-Chloro-2-fluoro-6-((5-nitrothiazol-2-yl)carbamoyl)phenyl pivalate, 9i

Prepared according to general procedure 1.4.3 from **7i**. Obtained an off-white solid (0.91 g, 61%).^{1}H NMR (500 MHz, $(CD_3)_2SO$) δ_H 13.79 (br s, 1H), 8.71 (s, 1H), 7.95 (dd, *J*=9.8, 2.4 Hz, 1H), 7.83–7.82 (m, 1H) and 1.27 (s, 9H). ^{13}C NMR (126 MHz, $(CD_3)_2SO$) δ_C 174.74, 162.95, 161.39, 153.77 (d, *J*=251.7 Hz), 142.39, 142.36, 135.57 (d, *J*=14.6 Hz), 130.55 (d, *J*=9.8 Hz), 129.14, 125.33 (d, *J*=3.1 Hz), 120.55 (d, *J*=22.6 Hz), 38.70 and 26.55. ^{19}F{^{1}H} NMR (376 MHz, $(CD_3)_2SO$) δ_F −125.42 (s). HRMS (ES+) *m/z* calcd for $C_{15}H_{12}ClFN_3O_5S$: 400.0176. Found $[M-H]^-$: 400.0174 (diff 0.51 ppm).

2-Chloro-6-((5-nitrothiazol-2-yl)carbamoyl)phenyl isobutyrate, 9j

Prepared according to general procedure 1.4.3 from **7j**. Purified by recrystallisation from ethyl acetate instead of flash chromatography. Obtained an orange, crystalline solid (0.74 g, 48%).^{1}H NMR (500 MHz, $(CD_3)_2SO$) δ_H 13.72 (br s, 1H), 8.70 (s, 1H), 7.86–7.81 (m, 2H), 7.49 (app. t, *J*=7.9 Hz, 1H), 2.88–2.82 (m, 1H) and 1.23 (d, *J*=6.9 Hz, 6H). ^{13}C NMR (126 MHz, $(CD_3)_2SO$) δ_C 173.40, 164.44, 161.72, 144.69, 142.48, 142.12, 133.54, 128.64, 128.07, 127.36, 127.31, 33.35 and 18.52. HRMS (ES−) *m/z* calcd for $C_{14}H_{11}ClN_3O_5S$: 368.0113. Found $[M-H]^-$: 368.0110 (diff −0.43 ppm).

2-Chloro-4-fluoro-6-((5-nitrothiazol-2-yl)carbamoyl)phenyl isobutyrate, 9k

Prepared according to general procedure 1.4.3 from **7k**. Obtained an off-white solid (0.43 g, 29%).^{1}H NMR (500 MHz, $(CD_3)_2SO$) δ_H 13.79 (br s, 1H), 8.71 (s, 1H), 7.94 (dd, *J*=8.1, 3.0 Hz, 1H), 7.79 (dd, *J*=8.3, 3.0 Hz, 1H), 2.84 (sept, *J*=7.0 Hz, 1H) and 1.22 (d, *J*=7.0 Hz, 6H). ^{13}C NMR (126 MHz, $(CD_3)_2SO$) δ_C 173.47, 163.36, 161.47, 158.40 (d, *J*=247.4 Hz), 142.39, 142.36, 141.37 (d, *J*=3.5 Hz), 128.81 (d, *J*=8.4 Hz), 128.46 (d, *J*=11.0 Hz), 120.74 (d, *J*=26.5 Hz), 115.96 (d, *J*=25.5 Hz), 33.29 and 18.51. ^{19}F{^{1}H} NMR (376 MHz, $(CD_3)_2SO$) δ_F −112.98 (s). HRMS (ES−) *m/z* calcd for $C_{14}H_{10}ClFN_3O_5S$: 386.0019. Found $[M-H]^-$: 386.0000 (diff 3.51 ppm).

1.5 General Procedures for Synthesis of Thiazolides 10a-m, 11a-k

1.5.1 Aminolysis of *O*-Acyl Thiazolides[8]

To a solution of the appropriate *O*-acetyl or *O*-pivaloyl thiazolide in THF or acetone (0.25 mL mmol^{-1}) was added ammonium hydroxide (35%, 1 volume). The reaction was allowed to stir overnight at room temperature, then the volatiles were removed *in vacuo* and the resulting crude mixture extracted with ethyl acetate (5 volumes). The organics were washed with HCl (1.0 M, 2 x 5 volumes) and brine (5 volumes) then dried over $MgSO_4$ and filtered. The solvent was removed *in vacuo* and the crude product was purified by recrystallisation from ethyl acetate or DCM-methanol if required.

1.5.2 Zemplén Deacylation of *O*-Acyl Thiazolides

The appropriate *O*-pivaloyl or *O*-isobutyryl thiazolide (1.0 eq) was suspended in anh. methanol (3.2 mL mmol^{-1}) under N_2. A solution of sodium methoxide (2.1 eq) in anh. methanol (1 volume) was then

added dropwise and the reaction was allowed to stir at room temperature for 1-3 hrs. Upon completion the reaction was quenched with dropwise addition of HCl (0.1 M, 4 volumes) and extracted with ethyl acetate (3 x 10 volumes). The combined organics were then washed with brine (10 volumes), dried over $MgSO_4$ and filtered before the solvent was removed *in vacuo*. The final product was purified by flash chromatography (eluting with ethyl acetate-hexane then acetone-ethyl acetate) or recrystallisation (from ethyl acetate or DCM-methanol) if necessary.

***N*-(5-Chlorothiazol-2-yl)-2-hydroxy-3-methylbenzamide, 10a**[8]

Prepared according to general procedure 1.5.1 from **8a**. Obtained an off-white solid (0.18 g, 82%).^{1}H NMR (400 MHz, $CDCl_3$) δ_H 11.37 (br s, 1H), 10.83 (br s, 1H), 7.48 (d, *J*=7.6 Hz, 1H), 7.39 (d, *J*=7.6 Hz, 1H), 7.22 (s, 1H), 6.86 (app. t, *J*=7.6 Hz, 1H) and 2.31 (s, 3H). ^{13}C NMR (101 MHz, $CDCl_3$) δ_C 168.1, 160.4, 156.2, 136.7, 134.8, 128.3, 123.9, 121.8, 118.8, 112.0 and 15.8. HRMS (ES+) *m/z* calcd for $C_{11}H_{10}ClN_2O_2S$: 269.0146. Found $[M+H]^+$: 269.0149 (diff −0.97 ppm). IR (ν_{max}, cm^{-1}) 3119 (O–H stretch), 3031 (N–H stretch), 1633 (C=O stretch), 1541 (N–H bend), 1300 (O–H bend), 1247 (C–N stretch), 745 (C–Cl stretch). Purity (HPLC) 95.1%, Rt=11.29 min. MP 171–172 °C.

***N*-(5-Chlorothiazol-2-yl)-2-hydroxy-3-isopropylbenzamide, 10b**

Prepared according to general procedure 1.5.1 from **8b**. Obtained an off-white solid (0.22 g, 98%).^{1}H NMR (400 MHz, $CDCl_3$) δ_H 11.54 (br s, 1H), 11.16 (br s, 1H), 7.50 (dd, *J*=7.8, 1.2 Hz, 1H), 7.47 (unresolved dd, *J*=7.8, 1.2 Hz, 1H), 7.19 (s, 1H), 6.91 (app. t, *J*=7.8 Hz, 1H), 3.43 (sept, *J*=7.0 Hz, 1H) and 1.27 (d, *J*=7.0 Hz, 6H). ^{13}C NMR (101 MHz, $CDCl_3$) δ_C 168.4, 159.7, 156.3, 138.4, 134.9, 132.5, 123.7, 121.7, 119.0, 112.1, 26.6 and 22.3. HRMS (ES+) *m/z* calcd for $C_{13}H_{14}ClN_2O_2S$: 297.0459. Found $[M+H]^+$: 297.0463 (diff −1.18 ppm). Purity (HPLC) 99.1%, Rt=12.65 min. MP 134–135 °C.

***N*-(5-Chlorothiazol-2-yl)-3-fluoro-2-hydroxybenzamide, 10c**

Prepared according to general procedure 1.5.1 from **8c**. Obtained the title compound as an off-white solid (0.073 g, 80%). ^{1}H NMR (400 MHz, $(CD_3)_2SO$) δ_H 12.39 (br s, 2H), 7.72 (d, 1H), 7.67 (s, 1H), 7.48–7.43 (m, 1H) and 6.98–6.93 (m, 1H). ^{13}C NMR (101 MHz, $(CD_3)_2SO$) δ_C 153.1, 150.7, 125.6, 120.5, 120.4, 119.6 and 119.4. ^{19}F{^{1}H} NMR (376 MHz, $(CD_3)_2SO$) δ_F −135.2. HRMS (ES+) *m/z* Calcd for $C_{10}H_7ClFN_2O_3S$: 272.9895. Found $[M+H]^+$: 272.9897. Elemental analysis calcd (%) for $C_{10}H_6ClFN_2O_3S$: C, 44.0; H, 2.2; N, 10.3. Found: C, 44.2; H, 2.25; N, 10.2.

***N*-(5-Chlorothiazol-2-yl)-4-fluoro-2-hydroxybenzamide, 10d**

Prepared according to general procedure 1.5.1 from **8d**. Obtained a white solid (0.70 g, 80%).^{1}H NMR (400 MHz, $(CD_3)_2SO$) δ_H 12.19 (br s, 2H), 8.00 (dd, *J*=8.6, 7.0 Hz, 1H), 7.61 (s, 1H) and 6.82 (m, 2H). ^{13}C NMR (101 MHz, $(CD_3)_2SO$) δ_C 165.9 (d, *J*=251.2 Hz), 164.9, 160.0 (d, *J*=12.2 Hz), 157.0, 134.9, 133.2 (d, *J*=11.5 Hz), 118.8, 114.2 (d, *J*=2.3 Hz), 107.7 (d, *J*=22.2 Hz) and 104.3 (d, *J*=24.2 Hz). ^{19}F{^{1}H} NMR (376 MHz, $(CD_3)_2SO$) δ_F −104.41. HRMS (ES+) *m/z* calcd for $C_{10}H_7ClFN_2O_2S$: 272.9895. Found $[M+H]^+$: 272.9896 (diff −0.1 ppm). IR (ν_{max}, cm^{-1}) 3647 (O–H stretch), 3277 (N–H stretch), 1677 (C=O stretch), 1548 (N–H in-plane bend), 1434 (O–H bend), 1279 (C–N stretch), 830 (C–Cl stretch) and 637 (N–H out-of-plane bend). Purity (HPLC) 98.4%, Rt=10.26 min. MP 231–233 °C.

***N*-(5-Chlorothiazol-2-yl)-5-fluoro-2-hydroxybenzamide, 10e**

Prepared according to general procedure 1.5.1 from **8e**. Obtained an off-white solid (0.085 g, 62%). ^{1}H NMR (400 MHz, $(CD_3)_2SO$) δ_H 12.02 (br s, 2H), 7.67 (dd, 1H), 7.63 (s, 1H), 7.35 (m, 1H) and 7.05 (dd, 1H). ^{13}C NMR (101 MHz, $(CD_3)_2SO$) δ_C 155.6 (d, *J*=234 Hz), 154.2 (br), 122.0, 121.8, 119.2, 119.1, 117.9, 116.1 and 115.8. ^{19}F NMR (376 MHz, $(CD_3)_2SO$) δ_F −124.1. HRMS (ES+) *m/z* Calcd for $C_{10}H_7ClFN_2O_2S$: 272.9895. Found $[M+H]^+$: 272.9904. Elemental analysis calcd (%) for $C_{10}H_6ClFN_2O_2S$: C, 44.05; H, 2.2; N, 10.2. Found: C, 44.1; H, 2.1; N, 10.3.

_N_-(5-Chlorothiazol-2-yl)-6-fluoro-2-hydroxybenzamide, 10f

Prepared according to general procedure 1.5.1 from **8f**. Obtained an off-white solid (0.116 g, 83%). ^{1}H NMR (400 MHz, $(CD_3)_2SO$) δ_H 12.78 (br s, 1H), 10.57 (br s, 1H), 7.57 (s, 1H), 7.32 (dd, 1H) and 6.79–6.72 (m, 2H). ^{13}C NMR (101 MHz, $(CD_3)_2SO$) δ_C 162.3, 160.1 (d, *J*=245 Hz), 156.8 (d), 156.1, 136.2, 132.5 (d), 118.8, 112.5, 111.9 (d) and 106.2 (d). ^{19}F{^{1}H} NMR (376 MHz, $(CD_3)_2SO$) δ_F −115.7. HRMS (ES−) *m/z* Calcd for $C_{10}H_5ClFN_2O_2S$: 270.9750. Found $[M-H]^-$: 270.9749. Elemental analysis calcd (%) for $C_{10}H_6ClFN_2O_2S$: C, 44.05; H, 2.2; N, 10.3. Found: C, 44.1; H, 2.1; N, 10.3.

3-Chloro-_N_-(5-chlorothiazol-2-yl)-2-hydroxybenzamide, 10g

Prepared according to general procedure 1.5.1 from **8g**. Obtained a pale orange solid (0.074 g, 31%).^{1}H NMR (400 MHz, $(CD_3)_2SO$) δ_H 7.94 (dd, *J*=7.9, 1.3 Hz, 1H), 7.71 (s, 1H), 7.61 (dd, *J*=7.9, 1.3 Hz, 1H) and 6.94 (app. t, *J*=7.9 Hz, 1H). ^{13}C NMR (101 MHz, $(CD_3)_2SO$) δ_C 168.79, 160.47, 155.51, 134.71, 130.90, 128.78, 121.86, 119.97, 118.97 and 118.13. HRMS (ES+) *m/z* calcd for $C_{10}H_7Cl_2N_2O_2S$: 288.9600. Found $[M+H]^+$: 288.9600 (diff 0.01 ppm). Elemental analysis calcd (%) for $C_{10}H_6Cl_2N_2O_2S$: C, 41.54; H, 2.09; N, 9.69; S, 11.09. Found: C, 41.44; H, 2.28; N, 9.64; S, 10.66. Purity (HPLC) 95.7%, Rt=10.78 min. MP 205 °C (decomp.).

4-Chloro-_N_-(5-chlorothiazol-2-yl)-2-hydroxybenzamide, 10h

Prepared according to general procedure 1.5.1 from **8h**. Obtained an off-white solid (0.10 g, 35%).^{1}H NMR (400 MHz, $(CD_3)_2SO$) δ_H 7.90 (d, *J*=8.1 Hz, 1H), 7.59 (s, 1H) and 7.04–7.01 (m, 2H). ^{13}C NMR (101 MHz, $(CD_3)_2SO$) δ_C 164.75, 158.56, 157.04, 138.81, 134.84, 132.37, 120.25, 118.89, 117.19 and 116.55. HRMS (ES+) *m/z* calcd for $C_{10}H_7Cl_2N_2O_2S$: 288.9600. Found $[M+H]^+$: 288.9600 (diff −0.13 ppm). Elemental analysis calcd (%) for $C_{10}H_6Cl_2N_2O_2S$: C, 41.54; H, 2.09; N, 9.69; S, 11.09. Found: C, 41.51; H, 2.19; N, 9.68; S, 10.98. Purity (HPLC) 97.1%, Rt=10.88 min. MP 223 °C (decomp.).

5-Chloro-_N_-(5-chlorothiazol-2-yl)-2-hydroxybenzamide, 10i

Prepared according to general procedure 1.5.1 from **8i**. Obtained an off-white solid (0.13 g, 52%).^{1}H NMR (400 MHz, $(CD_3)_2SO$) δ_H 7.86 (d, *J*=2.3 Hz, 1H), 7.59 (s, 1H), 7.48 (dd, *J*=8.7, 2.3 Hz, 1H) and 7.03 (d, *J*=8.7 Hz, 1H). ^{13}C NMR (101 MHz, $(CD_3)_2SO$) δ_C 164.79, 156.70, 135.23, 134.33, 129.69, 123.62, 119.58 and 119.04. HRMS (ES+) *m/z* calcd for $C_{10}H_7Cl_2N_2O_2S$: 288.9600. Found $[M+H]^+$: 288.9601 (diff −0.4 ppm). Elemental analysis calcd (%) for $C_{10}H_6Cl_2N_2O_2S$: C, 41.54; H, 2.09; N, 9.69; S, 11.09. Found: C, 41.60; H, 2.20; N, 9.73; S, 10.93. MP 234 °C (decomp.).

_N_-(5-Chlorothiazol-2-yl)-3,5-difluoro-2-hydroxybenzamide, 10j

Prepared according to general procedure 1.5.1 from **8j**. Obtained an off-white solid (0.15 g, 59%). ^{1}H NMR (400 MHz, $(CD_3)_2SO$) δ_H 12.54 (br s, 1H), 7.71 (s, 1H) and 7.57–7.50 (m, 2H). ^{13}C NMR (101 MHz,

$(CD_3)_2SO$) δ_C 166.07, 159.40, 153.84 (dd, *J*=237.8, 11.2 Hz), 151.69 (dd, *J*=245.3, 12.1 Hz), 143.68 (app. d, *J*=16.3 Hz), 132.27, 120.52 (dd, *J*=8.4, 3.4 Hz), 118.33, 111.01 (dd, *J*=24.2, 3.3 Hz) and 109.23 (dd, *J*=27.6, 22.7 Hz). $^{19}F\{^1H\}$ NMR (376 MHz, $(CD_3)_2SO$) δ_F −121.79 (app. s, 1F) and −130.23 (app. s, 1F). HRMS (ES+) *m/z* calcd for $C_{10}H_6ClF_2N_2O_2S$: 290.9801. Found $[M+H]^+$: 290.9802 (diff −0.27 ppm). Elemental analysis calcd (%) for $C_{10}H_5ClF_2N_2O_2S$: C, 41.32; H, 1.73; N, 9.64. Found: C, 41.55; H, 1.82; N, 9.42. MP 234 °C (decomp.).

5-Chloro-*N*-(5-chlorothiazol-2-yl)-3-fluoro-2-hydroxybenzamide, 10k

Prepared according to general procedure 1.5.1 from **8k**. Obtained an off-white solid (0.14 g, 74%).^1H NMR (400 MHz, $(CD_3)_2SO$) δ_H 7.77–7.69 (m, 2H) and 7.61 (dd, *J*=10.5, 2.4 Hz, 1H). ^{13}C NMR (101 MHz, $(CD_3)_2SO$) δ_C 166.11, 159.64, 152.08 (d, *J*=246.0 Hz), 146.63 (d, *J*=13.5 Hz), 132.05, 124.85 (d, *J*=3.3 Hz), 121.84 (d, *J*=9.5 Hz), 121.68 (d, *J*=3.2 Hz), 120.43 (d, *J*=22.1 Hz) and 118.18. $^{19}F\{^1H\}$ NMR (376 MHz, $(CD_3)_2SO$) δ_F −131.62 (s). HRMS (CI+) *m/z* calcd for $C_{10}H_6Cl_2FN_2O_2S$: 306.9506. Found $[M+H]^+$: 306.9505 (diff 0.09 ppm). Elemental analysis calcd (%) for $C_{10}H_5Cl_2FN_2O_2S$: C, 39.11; H, 1.64; N, 9.12. Found: C, 39.04; H, 1.58; N, 9.10. MP 242 °C (decomp.).

3-Chloro-*N*-(5-chlorothiazol-2-yl)-5-fluoro-2-hydroxybenzamide, 10l

Prepared according to general procedure 1.5.1 from **8l**. Obtained **10l** as a beige solid (0.19 g, 80%).^1H NMR (500 MHz, $(CD_3)_2SO$) δ_H 13.08 (br s, 1H), 7.79 (s, 1H), 7.72 (dd, *J*=9.1, 3.1 Hz, 1H) and 7.67 (dd, *J*=8.0, 3.1 Hz, 1H). ^{13}C NMR (126 MHz, $(CD_3)_2SO$) δ_C 168.17, 161.56, 154.14 (d, *J*=238.3 Hz), 152.41, 129.69, 122.42 (d, *J*=10.4 Hz), 121.92 (d, *J*=26.6 Hz), 119.29 (d, *J*=7.4 Hz), 117.87 and 114.50 (d, *J*=24.0 Hz). $^{19}F\{^1H\}$ NMR (376 MHz, $(CD_3)_2SO$) δ_F −122.96 (s). HRMS (ES+) *m/z* calcd for $C_{10}H_6Cl_2FN_2O_2S$: 306.9506. Found $[M+H]^+$: 306.9507 (diff −0.36 ppm). Elemental analysis calcd (%) for $C_{10}H_5Cl_2FN_2O_2S$: C, 39.11; H, 1.64; N, 9.12. Found: C, 39.62; H, 1.67; N, 8.93. Purity (HPLC) 97.0%, Rt=10.93 min. MP 244 °C (decomp.).

3,5-Dichloro-*N*-(5-chlorothiazol-2-yl)-2-hydroxybenzamide, 10m

Prepared according to general procedure 1.5.1 from **8m**. Obtained a pale yellow solid (0.21 g, 54%).^1H NMR (400 MHz, $(CD_3)_2SO$) δ_H 7.91 (app. d, *J*=2.4 Hz, 1H), 7.79 (s, 1H) and 7.75 (app. d, *J*=2.4 Hz, 1H). ^{13}C NMR (126 MHz, $(CD_3)_2SO$) δ_C 168.3, 162.0, 154.9, 133.5, 129.1, 128.1, 123.0, 122.7, 120.2 and 117.8. HRMS (ES+) *m/z* calcd for $C_{10}H_6Cl_3N_2O_2S$: 322.9210. Found $[M+H]^+$: 322.9200 (diff 1.42 ppm). Purity (HPLC) 99.7%, Rt=9.53 min. MP 224–225 °C.

3-Fluoro-2-hydroxy-*N*-(5-nitrothiazol-2-yl)benzamide, 11a

Prepared according to general procedure 1.5.2 from **9a**. Obtained a pale yellow crystalline solid (0.22 g, 38%).^1H NMR (400 MHz, $(CD_3)_2SO$) δ_H 8.73 (s, 1H), 7.67 (d, *J*=8.0 Hz, 1H), 7.47 (app. ddd, *J*=10.8, 8.0, 1.4 Hz, 1H) and 6.97 (app. td, *J*=8.0, 4.8 Hz, 1H). ^{13}C NMR (126 MHz, $(CD_3)_2SO$) δ_C 166.7, 162.8, 152.0 (d, *J*=241.7 Hz), 146.2 (d, *J*=15.2 Hz), 142.1, 141.9, 125.8 (d, *J*=3.3 Hz), 120.8 (d, *J*=14.0 Hz), 120.7 (d, *J*=1.3 Hz) and 119.7 (d, *J*=7.1 Hz). $^{19}F\{^1H\}$ NMR (376 MHz, $(CD_3)_2SO$) δ_F −134.73. HRMS (ES−) *m/z* calcd for $C_{10}H_5FN_3O_4S$: 281.9990. Found $[M-H]^-$: 282.0000 (diff −4.44 ppm). Purity (HPLC) 99.2%, Rt=9.51 min. MP 248–249 °C (decomp.).

4-Fluoro-2-hydroxy-*N*-(5-nitrothiazol-2-yl)benzamide, 11b

Prepared according to general procedure 1.5.2 from **9b**. Obtained an orange solid (0.14 g, 52%). ^{1}H NMR (400 MHz, $(CD_3)_2SO$) δ_H 8.50 (s, 1H), 7.93 (m, 1H) and 6.64 (m, 2H). ^{13}C NMR (126 MHz, $(CD_3)_2SO$) δ_C 171.0, 170.9, 165.5 (d, *J*=248.2 Hz), 163.5 (d, *J*=13.8 Hz), 145.7, 138.2, 132.1 (d, *J*=11.4 Hz), 116.6 (d, *J*=2.2 Hz), 105.6 (d, *J*=22.1 Hz) and 103.9 (d, *J*=23.1 Hz). ^{19}F{^{1}H} NMR (376 MHz, $(CD_3)_2SO$) δ_F −107.14. HRMS (ES−) *m/z* calcd for $C_{10}H_5FN_3O_4S$: 281.9990. Found $[M-H]^-$: 281.9995 (diff −1.65 ppm). Purity (HPLC) 99.9%, Rt=9.77 min. Rf 0.51 (1:9, MeOH:DCM). MP >250 °C (decomp.).

5-Fluoro-2-hydroxy-*N*-(5-nitrothiazol-2-yl)benzamide, 11c

Prepared according to general procedure 1.5.2 from **9c**. Obtained a yellow solid (0.21 g, 68%). ^{1}H NMR (400 MHz, $(CD_3)_2SO$) δ_H 8.68 (s, 1H), 7.64 (dd, *J*=9.3, 3.3 Hz, 1H), 7.35 (app. td, *J*=8.8, 3.3 Hz, 1H) and 7.02 (dd, *J*=8.8, 4.5 Hz, 1H). ^{13}C NMR (126 MHz, $(CD_3)_2SO$) δ_C 166.4, 163.9, 155.3 (d, *J*=235.9 Hz), 154.8, 143.2, 141.6, 122.0 (d, *J*=23.5 Hz), 119.1 (d, *J*=7.6 Hz), 118.4 (d, *J*=7.1 Hz) and 115.9 (d, *J*=24.4 Hz). ^{19}F{^{1}H} NMR (376 MHz, $(CD_3)_2SO$) δ_F −124.54. HRMS (ES−) *m/z* calcd for $C_{10}H_5FN_3O_4S$: 281.9990. Found $[M-H]^-$: 281.9995 (diff −1.68 ppm). Purity (HPLC) 99.3%, Rt=9.74 min. MP >250 °C (decomp.).

2-Fluoro-6-hydroxy-*N*-(5-nitrothiazol-2-yl)benzamide, 11d

Prepared according to general procedure 1.5.2 from **9d**. Obtained a yellow solid (0.12 g, 58%). ^{1}H NMR (400 MHz, $(CD_3)_2SO$) δ_H 8.68 (s, 1H), 7.64 (dd, *J*=8.9, 3.9 Hz, 1H), 7.35 (app. td, *J*=8.9, 3.2 Hz, 1H) and 7.02 (dd, *J*=8.9, 3.9 Hz, 1H. ^{13}C NMR (126 MHz, $(CD_3)_2SO$) δ_C 170.3, 169.6 (d, *J*=2.0 Hz), 163.5 (d, *J*=257.0 Hz), 163.2 (d, *J*=3.6 Hz), 145.7, 138.1, 133.0 (d, *J*=12.5 Hz), 113.7 (d, *J*=3.2 Hz), 109.0 (d, *J*=6.7 Hz) and 105.7 (d, *J*=22.7 Hz). ^{19}F{^{1}H} NMR (376 MHz, $(CD_3)_2SO$) δ_F −111.81. HRMS (ES−) *m/z* calcd for $C_{10}H_5FN_3O_4S$: 281.9990. Found $[M-H]^-$: 281.9983 (diff 2.56 ppm). Purity (HPLC) 99.7%, Rt=9.11 min. MP >250 °C (decomp.).

4-Chloro-2-hydroxy-*N*-(5-nitrothiazol-2-yl)benzamide, 11e[9]

Prepared according to general procedure 1.5.2 from **9e**. Obtained a beige powder (0.15 g, 63%). ^{1}H NMR (500 MHz, $(CD_3)_2SO$) δ_H 8.68 (s, 1H), 7.85 (d, *J*=8.4 Hz, 1H) and 7.05–7.03 (m, 2H). ^{13}C NMR (126 MHz, $(CD_3)_2SO$) δ_C 165.14, 161.76, 158.11, 142.09, 141.78, 138.76, 132.09, 119.86, 116.78 and 116.23. HRMS (ES−) *m/z* calcd for $C_{10}H_5ClN_3O_4S$: 297.9695. Found $[M-H]^-$: 297.9704 (diff −2.96 ppm). Purity (HPLC) 97.6%, Rt=10.34 min. MP 217 °C (decomp.).

5-Chloro-2-hydroxy-*N*-(5-nitrothiazol-2-yl)benzamide, 11f[9]

Prepared according to general procedure 1.5.2 from **9f**. Obtained an orange solid (0.11 g, 47%). ^{1}H NMR (500 MHz, $(CD_3)_2SO$) δ_H 8.65 (s, 1H), 7.82 (d, *J*=2.7 Hz, 1H), 7.48 (dd, *J*=8.8, 2.7 Hz, 1H) and 7.01 (dd, *J*=8.8 Hz, 1H). ^{13}C NMR (126 MHz, $(CD_3)_2SO$) δ_C 165.87, 163.37, 156.82, 142.63, 141.15, 133.91, 129.19, 122.86, 119.13, 118.96. HRMS (ES−) *m/z* calcd for $C_{10}H_5ClN_3O_4S$: 297.9695. Found $[M-H]^-$: 297.9701 (diff −2.25 ppm). Purity (HPLC) 95.3%, Rt=10.32 min. MP 171 °C (decomp.).

3,5-Difluoro-2-hydroxy-*N*-(5-nitrothiazol-2-yl)benzamide, 11g

Prepared according to general procedure 1.5.2 from **9g**. Obtained an off-white solid (0.20 g, 85%). ^{1}H NMR (500 MHz, $(CD_3)_2SO$) δ_H 8.69 (s, 1H) and 7.51–7.45 (m, 2H). ^{13}C NMR (126 MHz, $(CD_3)_2SO$) δ_C 166.39, 164.00, 153.29 (dd, *J*=237.8, 11.2 Hz), 151.26 (dd, *J*=245.0, 12.2 Hz), 143.42 (dd, *J*=14.4, 1.8 Hz), 141.81, 140.74, 120.43 (dd, *J*=8.4, 3.5 Hz), 110.60 (dd, *J*=24.0, 3.4 Hz) and 108.79 (dd, *J*=27.5, 22.8

Hz). $^{19}F\{^{1}H\}$ NMR (376 MHz, $(CD_3)_2SO$) δ_F −122.04 (app. s, 1F) and −130.29 (app. s, 1F).HRMS (ES−) *m/z* calcd for $C_{10}H_4F_2N_3O_4S$: 299.9896. Found $[M-H]^-$: 299.9902 (diff −2.11 ppm). Purity (HPLC) 97.5%, Rt=9.78 min. MP 192 °C (decomp.).

3,5-Dichloro-2-hydroxy-*N*-(5-nitrothiazol-2-yl)benzamide, 11h

Prepared according to general procedure 1.5.2 from **9h**. Obtained an orange solid (0.17 g, 63%).^{1}H NMR (400 MHz, $(CD_3)_2SO$) δ_H 8.55 (s, 1H), 7.78 (d, *J*=2.8 Hz, 1H) and 7.58 (d, *J*=2.8 Hz, 1H). ^{13}C NMR (126 MHz, $(CD_3)_2SO$) δ_C 169.5, 169.2, 157.9, 145.2, 139.3, 132.4, 127.7, 123.1, 121.0 and 120.1. HRMS (ES−) *m/z* calcd for $C_{10}H_4Cl_2N_3O_4S$: 331.9305. Found $[M-H]^-$: 331.9315 (diff −2.93 ppm). Purity (HPLC) 97.4%, Rt=10.93 min. MP >250 °C (decomp.).

5-Chloro-3-fluoro-2-hydroxy-*N*-(5-nitrothiazol-2-yl)benzamide, 11i

Prepared according to general procedure 1.5.2 from **9i**. Obtained an orange solid (0.13 g, 55%).^{1}H NMR (500 MHz, $(CD_3)_2SO$) δ_H 8.74 (s, 1H) and 7.66–7.65 (m, 2H). ^{13}C NMR (126 MHz, $(CD_3)_2SO$) δ_C 165.82, 162.99, 151.56 (d, *J*=245.9 Hz), 145.36 (d, *J*=14.9 Hz), 141.20, 141.10, 124.56 (d, *J*=3.3 Hz), 121.87 (d, *J*=9.5 Hz), 121.54 (d, *J*=3.3 Hz) and 120.26 (d, *J*=22.0 Hz). $^{19}F\{^{1}H\}$ NMR (376 MHz, $(CD_3)_2SO$) δ_F −131.57 (s). HRMS (CI−) *m/z* calcd for $C_{10}H_4ClFN_3O_4$: 315.9601. Found $[M-H]^-$: 315.9600 (diff −0.86 ppm). Purity (HPLC) 98.8%, Rt=10.34 min. MP 219 °C (decomp.).

3-Chloro-2-hydroxy-*N*-(5-nitrothiazol-2-yl)benzamide, 11j

Prepared according to general procedure 1.5.2 from **9j**. Obtained an orange solid (0.041 g, 47%).^{1}H NMR (500 MHz, $(CD_3)_2SO$) δ_H 8.65 (s, 1H), 7.87 (dd, *J*=7.9, 1.7 Hz, 1H), 7.55 (dd, *J*=7.8, 1.7 Hz, 1H) and 6.87 (app. t, *J*=7.8 Hz, 1H). ^{13}C NMR (126 MHz, $(CD_3)_2SO$) δ_C169.47, 166.96, 156.09, 142.80, 139.33, 133.59, 128.37, 121.32, 119.84 and 118.48. HRMS (ES−) *m/z* calcd for $C_{10}H_5ClN_3O_4S$: 297.9695. Found $[M-H]^-$: 297.9702 (diff −2.31 ppm). Purity (HPLC) 96.7%, Rt=10.15 min. MP 220 °C (decomp.).

3-Chloro-5-fluoro-2-hydroxy-*N*-(5-nitrothiazol-2-yl)benzamide, 11k

Prepared according to general procedure 1.5.2 from **9k**. Obtained a yellow crystalline solid (0.064 g, 26%).^{1}H NMR (500 MHz, $(CD_3)_2SO$) δ_H 8.80 (s, 1H) and 7.69–7.67 (m, 2H). ^{13}C NMR (126 MHz, $(CD_3)_2SO$) δ_C167.91, 164.38, 153.76 (d, *J*=238.4 Hz), 151.51, 140.23, 139.96, 122.27 (d, *J*=10.3 Hz), 121.58 (d, *J*=26.5 Hz), 119.52 (d, *J*=7.5 Hz) and 114.25 (d, *J*=24.3 Hz). $^{19}F\{^{1}H\}$ NMR (376 MHz, $(CD_3)_2SO$) δ_F −123.06 (s). HRMS (ESI−) *m/z* calcd for $C_{10}H_4ClFN_3O_4$: 315.9601. Found $[M-H]^-$: 315.9600 (diff −0.63 ppm). Purity (HPLC) 99.5%, Rt=10.32 min. MP 200 °C (decomp.).

1.6 General Procedures for Synthesis of 4′-Alkylsulfone Thiazolides, 15a-h

1.6.1 Bromothioester Synthesis[10]

To a solution of bromoacetyl bromide (1.0 eq) in anh. diethyl ether (3.6 mL $mmol^{-1}$), under N_2, was added the appropriate alkylthiol (1.0 eq) dropwise. The reaction was allowed to stir at room temperature for 1-2 hrs, then quenched with sat. $NaHCO_3$ (1 volume). The organics were separated, dried over $MgSO_4$ and filtered. The solvent was then removed *in vacuo* to afford the desired product.

*Note: Bromothioesters **12a-h** were found to be intensely malodourous and were analysed by ^{1}H and ^{13}C NMR only to minimise handling.*

1.6.2 Hantzsch Thiazole Synthesis

A solution of the appropriate bromothioester (1.4 eq) in ethanol (1 mL $mmol^{-1}$) was added dropwise to a stirred suspension of *N*-(*O*-pivaloyl) salicyloyl thiourea, **17**, (1.0 eq) in ethanol (1 volume). The reaction was stirred at room temperature for 2-5 days and monitored by TLC. Upon completion, the reaction was quenched with ½ sat. $NaHCO_3$ (4 volumes) and extracted with ethyl acetate (3 x 4 volumes). The combined organics were washed with H_2O (4 volumes) and brine (4 volumes), before drying over $MgSO_4$. The organics were filtered, and the solvent removed *in vacuo*. If necessary, purification was performed by column chromatography, eluting in mixtures of ethyl acetate and *n*-hexane.

1.6.3 Oxidation of *O*-Pivaloyl-4′-alkylsulfide Thiazolides

The appropriate *O*-pivaloyl-4′-alkylsulfide thiazolide (1.0 eq) was taken up in DCM (2.5 mL $mmol^{-1}$) and cooled to 0°C. A solution of *m*-CPBA (≤77%, 2.1 eq) in DCM (1 volume) was added dropwise, then the reaction was warmed to room temperature and stirred for 1-4 hrs. Upon completion, the reaction was quenched with aqueous $Na_2S_2O_3$ (10% wt/wt, 2 volumes) and extracted with ethyl acetate (3 x 4 volumes). The combined organics were washed with ½ sat. $NaHCO_3$ (2 x 4 volumes) and brine (4 volumes), then dried over MgSO4 and filtered. The solvent was removed *in vacuo* to afford the desired product.

1.6.4 Zemplén Deacylation of *O*-Pivaloyl-4′-alkylsulfone Thiazolides.

The appropriate *O*-pivaloyl-4′-alkylsulfone thiazolide (1.0 eq) was taken up in anh. methanol (5 mL $mmol^{-1}$) under N_2. A solution of sodium methoxide (2.1 eq) in anh. methanol (1 volume) was then added dropwise and the resulting solution stirred at room temperature for 1-2 hrs. Upon completion, the reaction was quenched with HCl (0.1 M, 4 volumes) and extracted with ethyl acetate (3 x 10 volumes). The combined organics were washed with brine (10 volumes), dried over $MgSO_4$ and filtered before the solvent was removed *in vacuo*. Purification by recrystallisation from ethanol afforded the desired product.

***S*-Ethyl 2-bromoethanethioate, 12a[10]**

Prepared according to general procedure 1.6.1 from ethanethiol. Obtained a clear, colourless oil (0.23 g, 89%).^{1}H NMR (400 MHz, $CDCl_3$) δ_H 3.99 (s, 2H), 2.92 (q, *J*=7.4 Hz, 2H) and 1.25 (t, *J*=7.4 Hz, 3H). ^{13}C NMR (101 MHz, $CDCl_3$) δ_C 192.64, 33.5, 24.56 and 14.33.

***S*-*n*-Propyl 2-bromoethanethioate, 12b[10]**

Prepared according to general procedure 1.6.1 from 1-propanethiol. Obtained a clear, colourless oil (1.66 g, quant. yield). ^{1}H NMR (400 MHz, $CDCl_3$) δ_H 4.04 (s, 2H), 2.93 (t, *J*=7.2 Hz, 2H), 1.69–1.60 (m, 2H) and 0.99 (t, *J*=7.4 Hz, 3H). ^{13}C NMR (101 MHz, $CDCl_3$) δ_C 192.77, 33.65, 32.01, 22.60 and 13.31.

***S*-*n*-Butyl 2-bromoethanethioate, 12c**

Prepared according to general procedure 1.6.1 from 1-butanethiol. Obtained a clear, colourless oil (1.61 g, 95%).^{1}H NMR (400 MHz, $CDCl_3$) δ_H 4.02 (s, 2H), 2.95 (t, *J*=7.3 Hz, 2H), 1.63–1.56 (m, 2H), 1.45–

1.36 (m, 2H) and 0.93 (t, *J*=7.3 Hz, 3H). ^{13}C NMR (101 MHz, $CDCl_3$) δ_C 192.74, 33.59, 31.16, 29.85, 21.90 and 13.53.

_S_-Isopropyl 2-bromoethanethioate, 12d[10]

Prepared according to general procedure 1.6.1 from 2-propanethiol. Obtained a clear, colourless oil (1.47 g, 93%).^{1}H NMR (400 MHz, $CDCl_3$) δ_H 4.00 (s, 2H), 3.69 (sept, *J*=6.9 Hz, 1H) and 1.34 (d, *J*=6.9 Hz, 6H). ^{13}C NMR (101 MHz, $CDCl_3$) δ_C 192.49, 36.23, 33.72 and 22.70.

_S_-(_tert_-Butyl) 2-bromoethanethioate, 12e[11,12]

Prepared according to general procedure 1.6.1 from 2-methylpropane-2-thiol. Obtained a clear, colourless oil (0.72 g, 62%).^{1}H NMR (400 MHz, $CDCl_3$) δ_H 3.95 (s, 2H) and 1.50 (s, 9H). ^{13}C NMR (101 MHz, $CDCl_3$) δ_C 192.38, 49.53, 34.15 and 29.60.

_S_-Cyclopentyl 2-bromoethanethioate, 12f

Prepared according to general procedure 1.6.1 from cyclopentanethiol. Obtained a clear, colourless oil (1.10 g, quant. yield). ^{1}H NMR (400 MHz, $CDCl_3$) δ_H 4.00 (2H, s), 3.79-3.72 (1H, m), 2.15-2.09 (2H, m) and 1.74-1.54 (6H, m). ^{13}C NMR (101 MHz, $CDCl_3$) δ_C 193.10, 43.69, 33.64, 33.00 and 24.72.

_S_-(Cyclopropylmethyl) 2-bromoethanethioate, 12g

Prepared according to general procedure 1.6.1 from cyclopropylmethanethiol. Obtained a yellow oil (1.08 g, 97% yield). ^{1}H NMR (400 MHz, $CDCl_3$) δ_H 4.04 (2H, s), 2.91 (2H, d, *J*=7.3 Hz), 1.06-0.97 (1H, m), 0.62-0.57 (2H, m) and 0.29-0.25 (2H, m). ^{13}C NMR (101 MHz, $CDCl_3$) δ_C 192.86, 35.92, 33.61, 10.46 and 5.82.

_S_-(Cyclobutylmethyl) 2-bromoethanethioate, 12h

Prepared according to general procedure 1.6.1 from cyclobutylmethanethiol. Obtained a clear, colourless oil (0.94 g, 90%).^{1}H NMR (400 MHz, $CDCl_3$) δ_H 4.02 (s, 2H), 3.03 (d, *J*=7.5 Hz, 2H), 2.57–2.46 (m, 1H), 2.13–2.05 (m, 2H), 1.90–1.80 (m, 2H) and 1.74–1.64 (m, 2H). ^{13}C NMR (101 MHz, $CDCl_3$) δ_C 192.75, 36.09, 34.71, 33.60, 27.55 and 17.81.

2-((4-(Ethylthio)thiazol-2-yl)carbamoyl)phenyl pivalate, 13a

Prepared according to general procedure 1.6.2 from **12a**. Obtained a yellow oil (0.23 g, 39%).^{1}H NMR (500 MHz, $(CD_3)_2SO$) δ_H 12.64 (s, 1H), 7.62–7.59 (m, 1H), 7.39 (app. dt, *J*=7.6, 1.0 Hz, 1H), 7.27 (dd, *J*=7.7, 1.6 Hz, 1H), 7.24 (dd, *J*=8.1, 1.0 Hz, 1H), 7.00 (s, 1H), 2.97 (q, *J*=7.3 Hz, 2H), 1.26 (t, *J*=7.3 Hz, 3H) and 1.23 (s, 9H). ^{13}C NMR (126 MHz, $(CD_3)_2SO$) δ_C 175.80, 163.93, 158.14, 148.35, 143.00, 132.37, 129.23, 127.34, 125.84, 123.08, 108.60, 38.50, 26.71, 26.42 and 14.54. HRMS (ES−) *m/z* calcd for $C_{17}H_{19}N_2O_3S_2$: 363.0843. Found $[M-H]^-$: 363.0830 (diff 1.96 ppm).

2-((4-(_n_-Propylthio)thiazol-2-yl)carbamoyl)phenyl pivalate, 13b

Prepared according to general procedure 1.6.2 from **12b**. Obtained an off-white solid (0.33 g, 49%).^{1}H NMR (500 MHz, $(CD_3)_2SO$) δ_H 12.63 (s, 1H), 7.71 (dd, *J*=7.7, 1.6 Hz, 1H), 7.60 (app. dt, *J*=7.9, 1.6 Hz, 1H), 7.39 (app. dt, *J*=7.7, 0.9 Hz, 1H), 7.24 (app. dt, *J*=7.9, 0.9 Hz, 1H), 6.99 (s, 1H), 2.94 (t, *J*=7.1 Hz, 2H), 1.64–1.57 (m, 2H), 1.23 (s, 9H) and 0.96 (t, *J*=7.3 Hz, 3H). ^{13}C NMR (126 MHz, $(CD_3)_2SO$) δ_C 175.79,

163.93, 158.09, 148.33, 143.16, 132.37, 129.24, 127.35, 125.84, 123.07, 108.55, 38.50, 34.21, 26.70, 22.24 and 13.15. HRMS (ES−) *m/z* calcd for $C_{18}H_{21}N_2O_3S_2$: 377.0999. Found $[M-H]^-$: 377.0990 (diff 2.36 ppm).

2-((4-(*n*-Butylthio)thiazol-2-yl)carbamoyl)phenyl pivalate, 13c

Prepared according to general procedure 1.6.2 from **12c**. Obtained a yellow solid (0.37 g, 52%).^{1}H NMR (500 MHz, $(CD_3)_2SO$) δ_H 12.63 (s, 1H), 7.72 (dd, *J*=7.6, 1.5 Hz, 1H), 7.60 (app. dt, *J*=7.9, 1.5 Hz, 1H), 7.39 (app. dt, *J*=7.6, 0.8 Hz, 1H), 7.24 (dd, *J*=7.9, 0.8 Hz, 1H), 6.99 (s, 1H), 2.96 (t, *J*=7.3 Hz, 2H), 1.60–1.54 (m, 2H), 1.43–1.36 (m, 2H), 1.23 (s, 9H) and 0.88 (t, *J*=7.4 Hz, 3H). ^{13}C NMR (126 MHz, $(CD_3)_2SO$) δ_C 175.80, 163.94, 158.10, 148.33, 143.23, 132.37, 129.24, 127.37, 125.85, 123.07, 108.51, 38.51, 31.95, 30.97, 26.72, 21.29 and 13.51. HRMS (ES−) *m/z* calcd for $C_{19}H_{23}N_2O_3S_2$: 391.1156. Found $[M-H]^-$: 391.1140 (diff 1.61 ppm).

2-((4-(Isopropylthio)thiazol-2-yl)carbamoyl)phenyl pivalate, 13d

Prepared according to general procedure 1.6.2 from **12d**. Obtained an off-white solid (0.36 g, 53%).^{1}H NMR (500 MHz, $(CD_3)_2SO$) δ_H 12.64 (s, 1H), 7.73 (dd, *J*=7.6, 1.4 Hz, 1H), 7.62–7.60 (m, 1H), 7.40 (app. t, *J*=7.6 Hz, 1H), 7.25 (app. d, *J*=8.1 Hz, 1H), 7.13 (s, 1H), 3.49 (sept, *J*=6.7 Hz, 1H), 1.27 (d, *J*=6.7 Hz, 6H) and 1.23 (s, 9H). ^{13}C NMR (126 MHz, $(CD_3)_2SO$) δ_C 176.26, 164.43, 158.44, 148.79, 142.59, 132.83, 129.72, 127.83, 126.31, 123.53, 112.73, 38.97, 37.74, 27.17 and 23.47. HRMS (ES−) *m/z* calcd for $C_{18}H_{21}N_2O_3S_2$: 377.0999. Found $[M-H]^-$: 377.0990 (diff 0.20 ppm).

2-((4-(*tert*-Butylthio)thiazol-2-yl)carbamoyl)phenyl pivalate, 13e

Prepared according to general procedure 1.6.2 from **12e**. Obtained an orange solid (0.30 g, 42%).^{1}H NMR (500 MHz, $(CD_3)_2SO$) δ_H 12.66 (s, 1H), 7.73 (dd, *J*=7.7, 1.6 Hz, 1H), 7.61 (app. dt, *J*=8.0, 1.6 Hz, 1H), 7.42–7.38 (m, 2H), 7.25 (dd, *J*=8.0, 0.8 Hz, 1H), 1.32 (s, 9H) and 1.23 (s, 9H). ^{13}C NMR (126 MHz, $(CD_3)_2SO$) δ_C 175.78, 164.09, 157.55, 148.23, 140.60, 132.33, 129.33, 127.45, 125.85, 123.02, 119.80, 46.40, 38.52, 30.87 and 26.71. HRMS (ES−) *m/z* calcd for $C_{19}H_{23}N_2O_3S_2$: 391.1156. Found $[M-H]^-$: 391.1150 (diff −0.05 ppm).

2-((4-(Cyclopentylthio)thiazol-2-yl)carbamoyl)phenyl pivalate, 13f

Prepared according to general procedure 1.6.2 from **12f**. Obtained as an orange solid (0.39 g, 54%).^{1}H NMR (500 MHz, $(CD_3)_2SO$) δ_H 12.63 (s, 1H), 7.73 (dd, *J*=7.7, 1.4 Hz, 1H), 7.61 (app. dt, *J*=7.9, 1.4 Hz, 1H), 7.40 (app. t, *J*=7.6 Hz, 1H), 7.25 (app. d, *J*=7.9 Hz, 1H), 7.04 (s, 1H), 3.70–3.65 (m, 1H), 2.07–2.01 (m, 2H), 1.71–1.66 (m, 2H), 1.62–1.51 (m, 4H) and 1.24 (s, 9H). ^{13}C NMR (126 MHz, $(CD_3)_2SO$) δ_C 175.80, 163.95, 157.95, 148.35, 143.37, 132.37, 129.24, 127.36, 125.84, 123.07, 110.21, 45.02, 38.50, 32.99, 26.71 and 24.37. HRMS (ES−) *m/z* calcd for $C_{20}H_{23}N_2O_3S_2$: 403.1156. Found $[M-H]^-$: 403.1140 (diff 1.55 ppm).

2-((4-((Cyclopropylmethyl)thio)thiazol-2-yl)carbamoyl)phenyl pivalate, 13g

Prepared according to general procedure 1.6.2 from **12g**. Obtained an off-white solid (0.44 g, 63%). ^{1}H NMR (500 MHz, $(CD_3)_2SO$) δ_H 12.63 (s, 1H), 7.72 (dd, *J*=7.7, 1.6 Hz, 1H), 7.60 (app. dt, *J*=8.0, 1.6 Hz, 1H), 7.39 (app. dt, *J*=7.7, 0.9 Hz, 1H), 7.24 (dd, *J*=8.0, 0.9 Hz, 1H), 6.99 (s, 1H), 2.93 (d, *J*=7.1 Hz, 2H), 1.23 (s, 9H), 1.08–1.00 (m, 1H), 0.56–0.47 (m, 2H) and 0.28–0.19 (m, 2H). ^{13}C NMR (126 MHz, $(CD_3)_2SO$) δ_C 175.79, 163.93, 158.02, 148.34, 143.41, 132.35, 129.21, 127.35, 125.83, 123.07, 108.38, 38.50, 37.75, 26.71, 10.71 and 5.42. HRMS (ES−) *m/z* calcd for $C_{19}H_{21}N_2O_3S_2$: 389.0999. Found $[M-H]^-$: 389.0990 (diff 1.33 ppm).

2-((4-((Cyclobutylmethyl)thio)thiazol-2-yl)carbamoyl)phenyl pivalate, 13h

Prepared according to general procedure 1.6.2 from **12h**. Obtained a yellow solid (0.43 g, 60%). ^{1}H NMR (500 MHz, $(CD_3)_2SO$) δ_H 12.62 (s, 1H), 7.72 (dd, *J*=7.7, 1.6 Hz, 1H), 7.62–7.59 (m, 1H), 7.39 (app. dt, *J*=7.6, 1.1 Hz, 1H), 7.24 (dd, *J*=8.1, 0.9 Hz, 1H), 6.98 (s, 1H), 3.04 (d, *J*=7.5 Hz, 2H), 2.53–2.44 (m, 1H), 2.06–1.99 (m, 2H), 1.86–1.75 (m, 2H), 1.73–1.65 (m, 2H) and 1.23 (s, 9H). ^{13}C NMR (126 MHz, $(CD_3)_2SO$) δ_C 175.78, 163.92, 158.02, 148.32, 143.07, 132.35, 129.23, 127.35, 125.83, 123.06, 108.72, 38.54, 38.50, 34.55, 27.21, 26.70 and 17.48. HRMS (ES−) *m/z* calcd for $C_{20}H_{23}N_2O_3S_2$: 403.1156. Found $[M-H]^-$: 403.1150 (diff 1.30 ppm).

2-((4-(Ethylsulfonyl)thiazol-2-yl)carbamoyl)phenyl pivalate, 14a

Prepared according to general procedure 1.6.3 from **13a**. Obtained a white semi-solid (0.16 g, 92%). ^{1}H NMR (500 MHz, $(CD_3)_2SO$) δ_H 13.12 (s, 1H), 8.11 (s, 1H), 7.77 (dd, *J*=7.5, 1.4 Hz, 1H), 7.63 (app. dt, *J*=7.9, 1.4 Hz, 1H), 7.41 (app. t, *J*=7.5 Hz, 1H), 7.26 (app. d, *J*=7.9 Hz, 1H), 3.30 (q, *J*=7.4 Hz, 2H), 1.24 (s, 9H) and 1.15 (t, *J*=7.4 Hz, 3H).

2-((4-(*n*-Propylsulfonyl)thiazol-2-yl)carbamoyl)phenyl pivalate, 14b

Prepared according to general procedure 1.6.3 from **13b**. Obtained a pale-yellow oil (0.25 g, 84%). ^{1}H NMR (500 MHz, $(CD_3)_2SO$) δ_H 13.12 (s, 1H), 8.11 (s, 1H), 7.77 (app. d, *J*=7.7 Hz, 1H), 7.64 (app. t, *J*=8.0 Hz, 1H), 7.41 (app. t, *J*=7.7 Hz, 1H), 7.26 (app. d, *J*=8.0 Hz, 1H), 3.28 (t, *J*=7.7 Hz, 2H), 1.65–1.57 (m, 2H), 1.24 (s, 9H) and 0.94 (t, *J*=7.4 Hz, 3H). ^{13}C NMR (126 MHz, $(CD_3)_2SO$) δ_C 175.79, 164.74, 160.02, 148.47, 146.89, 132.81, 129.35, 126.68, 125.91, 123.15, 121.67, 54.83, 38.51, 26.68, 16.08 and 12.57. HRMS (ES−) *m/z* calcd for $C_{18}H_{21}N_2O_3S_2$: 409.0897. Found $[M-H]^-$: 409.0890 (diff 1.19 ppm).

2-((4-(*n*-Butylsulfonyl)thiazol-2-yl)carbamoyl)phenyl pivalate, 14c

Prepared according to general procedure 1.6.3 from **13c**. Obtained a yellow oil (0.29 g, 82%). ^{1}H NMR (500 MHz, $(CD_3)_2SO$) δ_H 13.11 (s, 1H), 8.11 (s, 1H), 7.77 (dd, *J*=7.6, 1.6 Hz, 1H), 7.63 (app. dt, *J*=7.9, 1.6 Hz, 1H), 7.41 (app. dt, *J*=7.6, 1.0 Hz, 1H), 7.26 (dd, *J*=7.9, 1.0 Hz, 1H), 3.31–3.28 (m, 2H), 1.59–1.53 (m, 2H), 1.39–1.32 (m, 2H), 1.24 (s, 9H) and 0.85 (t, *J*=7.4 Hz, 3H). ^{13}C NMR (126 MHz, $(CD_3)_2SO$) δ_C 175.79, 164.76, 160.03, 148.46, 146.87, 132.80, 129.35, 126.70, 125.92, 123.15, 121.67, 52.95, 38.51, 26.69, 24.23, 20.79 and 13.41. HRMS (ES−) *m/z* calcd for $C_{19}H_{23}N_2O_5S_2$: 423.1054. Found $[M-H]^-$: 423.1050 (diff 0.77 ppm).

2-((4-(Isopropylsulfonyl)thiazol-2-yl)carbamoyl)phenyl pivalate, 14d

Prepared according to general procedure 1.6.3 from **13d**. Obtained an off-white solid (0.30 g, 95%). ^{1}H NMR (500 MHz, $(CD_3)_2SO$) δ_H 13.11 (s, 1H), 8.12 (s, 1H), 7.77 (dd, *J*=7.6, 1.5 Hz, 1H), 7.63 (app. dt, *J*=7.9,

1.5 Hz, 1H), 7.41 (app. t, *J*=7.6 Hz, 1H), 7.26 (app. d, *J*=7.9 Hz, 1H), 3.42 (sept, *J*=6.8 Hz, 1H), 1.24 (s, 9H) and 1.22 (d, *J*=6.8 Hz, 6H). ^{13}C NMR (126 MHz, $(CD_3)_2SO$) δ_C 175.79, 164.73, 159.90, 148.44, 145.26, 132.78, 129.37, 126.72, 125.91, 123.15, 122.91, 53.23, 38.52, 26.69 and 14.92. HRMS (ES−) *m/z* calcd for $C_{18}H_{21}N_2O_5S_2$: 409.0897. Found $[M-H]^-$: 409.0900 (diff −1.01 ppm).

2-((4-(*tert*-Butylsulfonyl)thiazol-2-yl)carbamoyl)phenyl pivalate, 14e

Prepared according to general procedure 1.6.3 from **13e**. Obtained a white solid (0.24 g, quant.). ^{1}H NMR (500 MHz, $(CD_3)_2SO$) δ_H 13.42 (s, 1H), 8.13 (s, 1H), 7.78 (dd, *J*=7.6, 1.5 Hz, 1H), 7.64 (app. dt, *J*=7.9, 1.5 Hz, 1H), 7.41 (app. dt, *J*=7.6, 0.8 Hz, 1H), 7.27 (app. d, *J*=7.9 Hz, 1H), 1.32 (s, 9H) and 1.24 (s, 9H). ^{13}C NMR (126 MHz, $(CD_3)_2SO$) δ_C 175.78, 164.75, 159.40, 148.40, 144.25, 132.74, 129.43, 126.78, 125.89, 124.58, 123.13, 59.12, 38.53, 26.71 and 22.99. HRMS (ES−) *m/z* calcd for $C_{19}H_{23}N_2O_5S$: 423.1054. Found $[M-H]^-$: 423.1050 (diff 0.70 ppm).

2-((4-(Cyclopentylsulfonyl)thiazol-2-yl)carbamoyl)phenyl pivalate, 14f

Prepared according to general procedure 1.6.3 from **13f**. Obtained a white solid (0.29 g, 89%).^{1}H NMR (500 MHz, $(CD_3)_2SO$) δ_H 13.10 (s, 1H), 8.12 (s, 1H), 7.77 (dd, *J*=7.6, 1.6 Hz, 1H), 7.63 (app. dt, *J*=7.9, 1.6 Hz, 1H), 7.41 (app. dt, *J*=7.6, 0.9 Hz, 1H), 7.26 (dd, *J*=7.9, 0.9 Hz, 1H), 3.78–3.72 (m, 1H), 1.97–1.83 (m, 4H), 1.62–1.55 (m, 4H) and 1.24 (s, 9H). ^{13}C NMR (126 MHz, $(CD_3)_2SO$) δ_C 175.80, 164.75, 159.87, 148.44, 146.48, 132.77, 129.37, 126.74, 125.90, 123.14, 122.28, 61.68, 38.52, 26.69, 26.50 and 25.54. HRMS (ES−) *m/z* calcd for $C_{20}H_{23}N_2O_5S_2$: 435.1054. Found $[M-H]^-$: 435.1050 (diff 0.06 ppm).

2-((4-((Cyclopropylmethyl)sulfonyl)thiazol-2-yl)carbamoyl)phenyl pivalate, 14g

Prepared according to general procedure 1.6.3 from **13g**. Obtained a white solid (0.30 g, 94%).^{1}H NMR (500 MHz, $(CD_3)_2SO$) δ_H 13.11 (s, 1H), 8.12 (s, 1H), 7.77 (dd, *J*=7.7, 1.5 Hz, 1H), 7.63 (app. dt, *J*=8.0, 1.5 Hz, 1H), 7.41 (app. dt, *J*=7.7, 0.8 Hz, 1H), 7.26 (app. d, *J*=8.0 Hz, 1H), 3.25 (d, *J*=7.2 Hz, 2H), 1.24 (s, 9H), 0.94–0.86 (m, 1H), 0.53–0.43 (m, 2H) and 0.20–0.14 (m, 2H). ^{13}C NMR (126 MHz, $(CD_3)_2SO$) δ_C 175.81, 164.77, 159.90, 148.44, 147.01, 132.76, 129.31, 126.75, 125.91, 123.16, 121.99, 57.96, 38.52, 26.69, 4.36 and 3.65. HRMS (ES−) *m/z* calcd for $C_{19}H_{21}N_2O_5S_2$: 421.0897. Found $[M-H]^-$: 421.0890 (diff 1.08 ppm).

2-((4-((Cyclobutylmethyl)sulfonyl)thiazol-2-yl)carbamoyl)phenyl pivalate, 14h

Prepared according to general procedure 1.6.3 from **13h**. Obtained a white solid (0.30 g, 89%).^{1}H NMR (500 MHz, $(CD_3)_2SO$) δ_H 13.11 (s, 1H), 8.08 (s, 1H), 7.77 (dd, *J*=7.7, 1.4 Hz, 1H), 7.63 (app. dt, *J*=8.0, 1.4 Hz, 1H), 7.43–7.40 (m, 1H), 7.26 (app. d, *J*=8.0 Hz, 1H), 3.42 (d, *J*=7.2 Hz, 2H), 2.63–2.54 (m, 1H), 1.99–1.91 (m, 2H), 1.86–1.68 (m, 4H) and 1.24 (s, 9H). ^{13}C NMR (126 MHz, $(CD_3)_2SO$) δ_C 175.80, 164.77, 159.95, 148.42, 147.19, 132.77, 129.32, 126.74, 125.91, 123.14, 121.72, 58.87, 38.51, 29.32, 27.48, 26.68 and 18.69. HRMS (ES−) *m/z* calcd for $C_{20}H_{23}N_2O_5S_2$: 435.1054. Found $[M-H]^-$: 435.1050 (diff −0.19 ppm).

***N*-(4-(Ethylsulfonyl)thiazol-2-yl)-2-hydroxybenzamide, 15a[13]**

Prepared according to general procedure 1.6.4 from **14a**. Obtained an off-white crystalline solid (0.053 g, 53%).^{1}H NMR (500 MHz, $(CD_3)_2SO$) δ_H 12.28 (br s, 1H), 11.64 (br s, 1H), 8.14 (s, 1H), 7.97 (dd, *J*=7.9, 1.7 Hz, 1H), 7.52–7.48 (m, 1H), 7.06 (app. d, *J*=8.2 Hz, 1H), 7.02–6.99 (m, 1H), 3.33 (q, *J*=7.4 Hz, 2H) and 1.16 (t, *J*=7.4 Hz, 3H). ^{13}C NMR (126 MHz, $(CD_3)_2SO$) δ_C165.07, 159.55, 157.34, 146.35, 134.79, 130.28, 122.20, 119.79, 117.20, 116.41, 47.72 and 6.99. HRMS (ES−) *m/z* calcd for $C_{12}H_{11}N_2O_4S_2$: 311.0166. Found $[M-H]^-$: 311.0160 (diff −0.12 ppm). Purity (HPLC) 97.4%, Rt=8.51 min. MP 224 °C (decomp.).

2-Hydroxy-*N*-(4-(*n*-propylsulfonyl)thiazol-2-yl)benzamide, 15b

Prepared according to general procedure 1.6.4 from **14b**. Obtained an off-white crystalline solid (0.13 g, 56%).^{1}H NMR (500 MHz, $(CD_3)_2SO$) δ_H 12.27 (br s, 1H), 11.63 (br s, 1H), 8.13 (s, 1H), 7.97 (dd, *J*=7.9, 1.7 Hz, 1H), 7.52–7.49 (m, 1H), 7.07 (dd, *J*=8.3, 0.6 Hz, 1H), 7.03–6.99 (m, 1H), 3.31 (t, *J*=7.7 Hz, 2H), 1.67–1.59 (m, 2H) and 0.95 (t, *J*=7.4 Hz, 3H). ^{13}C NMR (126 MHz, $(CD_3)_2SO$) δ_C 165.53, 159.98, 157.79, 147.44, 135.26, 130.76, 122.41, 120.27, 117.67, 116.88, 55.21, 16.42 and 13.02. HRMS (ES−) *m/z* calcd for $C_{13}H_{13}N_2O_4S_2$: 325.0322. Found $[M-H]^-$: 325.0320 (diff −0.27 ppm). Purity (HPLC) 95.4%, Rt=9.07 min. MP 202–204 °C (decomp.).

***N*-(4-(*n*-Butylsulfonyl)thiazol-2-yl)-2-hydroxybenzamide, 15c**

Prepared according to general procedure 1.6.4 from **14c**. Obtained as a white crystalline solid (0.15 g, 63%).^{1}H NMR (500 MHz, $(CD_3)_2SO$) δ_H 12.28 (br s, 1H), 11.63 (br s, 1H), 8.13 (s, 1H), 7.97 (dd, *J*=7.9, 1.7 Hz, 1H), 7.52–7.48 (m, 1H), 7.06 (dd, *J*=8.3, 0.7 Hz, 1H), 7.02–6.99 (m, 1H), 3.34–3.31 (m, 2H), 1.61–1.55 (m, 2H), 1.40–1.33 (m, 2H) and 0.85 (t, *J*=7.4 Hz, 3H). ^{13}C NMR (126 MHz, $(CD_3)_2SO$) δ_C 165.09, 159.52, 157.34, 146.95, 134.80, 130.29, 121.94, 119.80, 117.20, 116.43, 52.86, 24.09, 20.75 and 13.43. HRMS (ES−) *m/z* calcd for $C_{14}H_{15}N_2O_4S_2$: 339.0479. Found $[M-H]^-$: 339.0480 (diff −0.35 ppm). Purity (HPLC) 97.2%, Rt=9.62 min. MP 204–206 °C (decomp.).

2-Hydroxy-*N*-(4-(isopropylsulfonyl)thiazol-2-yl)benzamide, 15d[13]

Prepared according to general procedure 1.6.4 from **14d**. Obtained a white crystalline solid (0.16 g, 69%).^{1}H NMR (500 MHz, $(CD_3)_2SO$) δ_H 12.28 (br s, 1H), 11.62 (br s, 1H), 8.14 (s, 1H), 7.97 (dd, *J*=8.0, 1.5 Hz, 1H), 7.52–7.48 (m, 1H), 7.06 (app. d, *J*=8.0 Hz, 1H), 7.01 (app. t, *J*=7.5 Hz, 1H), 3.47 (sept, *J*=6.8 Hz, 1H) and 1.22 (d, *J*=6.8 Hz, 6H). ^{13}C NMR (126 MHz, $(CD_3)_2SO$) δ_C 165.56, 159.90, 157.80, 145.76, 135.25, 130.75, 123.62, 120.25, 117.66, 116.90, 53.49 and 15.35. HRMS (ES−) *m/z* calcd for $C_{13}H_{13}N_2O_4S_2$: 325.0322. Found $[M-H]^-$: 325.0310 (diff 0.87 ppm). Purity (HPLC) 97.7%, Rt=9.81 min. MP 202–203 °C (decomp.).

***N*-(4-(*tert*-Butylsulfonyl)thiazol-2-yl)-2-hydroxybenzamide, 15e**

Prepared according to general procedure 1.6.4 from **14e**. Obtained a white solid (0.11 g, 63%). ^{1}H NMR (500 MHz, $(CD_3)_2SO$) δ_H 12.31 (br s, 1H), 11.58 (br s, 1H), 8.14 (s, 1H), 7.97 (dd, *J*=7.9, 1.6 Hz, 1H), 7.50 (app. dt, *J*=7.8, 1.7 Hz, 1H), 7.05 (app. d, *J*=8.2 Hz, 1H), 7.06–6.99 (m, 1H) and 1.32 (s, 9H). ^{13}C NMR (126 MHz, $(CD_3)_2SO$) δ_C 165.71, 159.39, 157.82, 144.69, 135.20, 130.70, 125.34, 120.21, 117.65, 116.98, 59.62 and 23.47. HRMS (ES−) *m/z* calcd for $C_{14}H_{16}N_2O_4S_2$: 339.0479. Found $[M-H]^-$: 339.0470 (diff 1.90 ppm). Purity (HPLC) 99.7%, Rt=9.17 min. MP 228 °C (decomp.).

***N*-(4-(Cyclopentylsulfonyl)thiazol-2-yl)-2-hydroxybenzamide, 15f**

Prepared according to general procedure 1.6.4 from **14f**. Obtained a white solid (0.10 g, 47%).^{1}H NMR (500 MHz, $(CD_3)_2SO$) δ_H 12.27 (br s, 1H), 11.63 (br s, 1H), 8.14 (s, 1H), 7.97 (dd, *J*=7.9, 1.7 Hz, 1H), 7.52–7.48 (m, 1H), 7.06 (dd, *J*=8.3, 0.6 Hz, 1H), 7.03–6.99 (m, 1H), 3.84–3.78 (m, 1H), 1.97–1.83 (m, 4H) and 1.64–1.55 (m, 4H). ^{13}C NMR (126 MHz, $(CD_3)_2SO$) δ_C 165.50, 159.87, 157.77, 147.01, 135.25, 130.77, 122.93, 120.27, 117.66, 116.92, 61.93, 26.94 and 26.04. HRMS (ES−) *m/z* calcd for $C_{15}H_{15}N_2O_4S_2$: 351.0479. Found $[M-H]^-$: 351.0470 (diff 1.57 ppm). Purity (HPLC) 99.7%, Rt=9.52 min. MP 208–209 °C.

***N*-(4-((Cyclopropylmethyl)sulfonyl)thiazol-2-yl)-2-hydroxybenzamide, 15g**

Prepared according to general procedure 1.6.4 from **14g**. Obtained an off-white crystalline solid (0.10 g, 52%).^{1}H NMR (500 MHz, $(CD_3)_2SO$) δ_H 12.26 (br s, 1H), 11.64 (br s, 1H), 8.14 (s, 1H), 7.97 (dd, *J*=7.9, 1.0 Hz, 1H), 7.52–7.49 (m, 1H), 7.07 (app. d, *J*=7.9 Hz, 1H), 7.01 (app. t, *J*=7.5 Hz, 1H), 3.29 (d, *J*=7.1 Hz, 2H), 0.94–0.89 (m, 1H), 0.50–0.46 (m, 2H) and 0.19–0.16 (m, 2H). ^{13}C NMR (126 MHz, $(CD_3)_2SO$) δ_C 165.04, 159.37, 157.34, 147.08, 134.82, 130.31, 122.24, 119.82, 117.21, 116.39, 57.84, 4.29 and 3.64. HRMS (ES−) *m/z* calcd for $C_{14}H_{13}N_2O_4S_2$: 337.0322. Found $[M-H]^-$: 337.0310 (diff 1.67 ppm). Purity (HPLC) 98.3%, Rt=9.08 min. MP 179–182 °C (decomp.).

***N*-(4-((Cyclobutylmethyl)sulfonyl)thiazol-2-yl)-2-hydroxybenzamide, 15h**

Prepared according to general procedure 1.6.4 from **14h**. Obtained a white crystalline solid (0.088 g, 40%).^{1}H NMR (500 MHz, $(CD_3)_2SO$) δ_H 12.27 (br s, 1H), 11.64 (br s, 1H), 8.10 (s, 1H), 7.97 (app. d, *J*=8.0 Hz, 1H), 7.50 (app. t, *J*=7.5 Hz, 1H), 7.07 (app. d, *J*=8.0 Hz, 1H), 7.01 (app. t, *J*=7.5 Hz, 1H), 3.45 (d, *J*=7.2 Hz, 2H), 2.65–2.58 (m, 1H), 1.97–1.93 (m, 2H) and 1.86–1.71 (m, 4H). ^{13}C NMR (126 MHz, $(CD_3)_2SO$) δ_C 165.07, 159.44, 157.34, 147.28, 134.82, 130.30, 121.98, 119.82, 117.21, 116.41, 58.77, 29.26, 27.50 and 18.73. HRMS (ES−) *m/z* calcd for $C_{15}H_{15}N_2O_4S_2$: 351.0479. Found $[M-H]^-$: 351.0470 (diff 0.73 ppm). Purity (HPLC) 97.5%, Rt=9.77 min. MP 191–192 °C (decomp.).

2-(Pivaloyloxy)benzoic acid, 16[14]

2-Hydroxybenzoic acid (5.02 g, 36.4 mmol, 1.0 eq) was dissolved in anh. THF (75 mL) in N_2. The reaction was cooled to 0℃ before pivaloyl chloride (9.75 mL, 79.2 mmol, 2.2 eq) and anh. pyridine (3.0 mL, 37.2 mmol, 1.0 eq) were added dropwise. The reaction was warmed to room temperature and allowed to stir overnight, after which the reaction was acidified with HCl (12 M, 50 mL) and stirred for 30 mins. The reaction was extracted with ethyl acetate (3 x 100 mL) and the combined organics were washed with H_2O (2 x 100 mL) and brine (100 mL). The organics were dried over $MgSO_4$, filtered and the solvent removed *in vacuo*. The crude product was dissolved in hot diethyl ether and triturated with hexane to afford the title compound as a white, crystalline solid (5.92 g, 73% yield). ^{1}H NMR (500 MHz, $(CD_3)_2SO$) δ_H 13.07 (1H, br s), 7.90 (1H, dd, *J*=7.8, 1.7 Hz), 7.62 (1H, app. dt, *J*=7.7, 1.7 Hz), 7.37 (1H, app. dt, *J*=7.7, 0.9 Hz), 7.15 (1H, dd, *J*=8.1, 0.9 Hz) and 1.29 (9H, s). ^{13}C NMR (126 MHz, $(CD_3)_2SO$) δ_C 175.98, 165.77, 150.20, 133.61, 131.25, 126.00, 124.52, 123.62, 38.40 and 26.84. HRMS (ES−) calcd for $C_{12}H_{13}O_4$: 221.0819. Found $[M-H]^-$ 221.0810 (diff 3.86 ppm).

2-(Carbamothioylcarbamoyl)phenyl pivalate, 17

2-(Pivaloyloxy)benzoic acid, **16**, (5.00 g, 22.51 mmol, 1.0 eq) was dissolved in anh. DCM (90 mL) under N_2. To the resulting solution was added thionyl chloride (1.80 mL, 24.8 mmol, 1.1 eq) and anh. pyridine (12 drops). The reaction was then heated to reflux and stirred for 3 hrs. After cooling to room

temperature, the volatiles were removed *in vacuo*. The residue was dissolved in anh. acetonitrile (70 mL) before ammonium thiocyanate (freshly recrystallised from ethanol, 1.88 g, 24.7 mmol, 1.1 eq) was added portion-wise. The reaction was then heated to reflux and stirred for 2 hrs. After cooling to room temperature, the reaction was filtered through a celite pad. Ammonium hydroxide (30%, 1.60 mL, 24.75 mmol, 1.1 eq) was added dropwise at 0°C, then the reaction was warmed to room temperature and stirred for 1.0 hr. Extra ammonium hydroxide (30%, 0.80 mL, 12.40 mmol, 0.55 eq) was added and the reaction stirred for a further 30 mins. The reaction was then acidified with HCl (2 M, 3 mL), diluted with H_2O (100 mL) and extracted with ethyl acetate (3 x 100 mL). The combined organics were washed with H_2O (100 mL) and brine (100 mL), then dried over $MgSO_4$, filtered, and the solvent was removed *in vacuo*. Recrystallisation from 50% ethyl acetate-*n*-hexane) afforded the title compound as a yellow crystalline solid (2.99 g, 47% yield). ^{1}H NMR (500 MHz, $(CD_3)_2SO$) δ_H 11.34 (1H, br s), 9.64 (1H, br s), 9.51 (1H, br s), 7.61-7.56 (2H, m), 7.35 (1H, app. dt, *J*=7.5, 0.6 Hz), 7.21 (1H, app. d, *J*=8.0 Hz) and 1.29 (9H, s). ^{13}C NMR (126 MHz, $(CD_3)_2SO$) δ_C 181.65, 175.86, 166.48, 147.89, 132.44, 129.39, 127.86, 125.74, 122.88, 38.66 and 26.75. HRMS (ES−) calcd for $C_{13}H_{15}N_2O_3S$: 279.0809. Found $[M-H]^-$ 279.0800 (diff 3.03 ppm).

2. Antiviral Assay Procedures

2.1 Cell Culture and Treatments

Madin-Darby canine kidney (MDCK) cells (American Type Culture Collection, ATCC CCL-34) were grown at 37°C in a 5% CO_2 atmosphere in RPMI-1640 medium (Euroclone), supplemented with 10% fetal calf serum (FCS), 2 mM glutamine and antibiotics. Tizoxanide and thiazolide analogues were dissolved in DMSO stock solution and diluted in culture medium before testing. Compounds were added to infected cells after a one-hour virus adsorption period and maintained in the medium for the duration of the experiment. Controls received equal amounts of DMSO vehicle, which did not affect cell viability or virus replication. Cell viability was determined by the 3-(4,5-dimethylthiazol-2-yl)-2,5-diphenyltetrazolium bromide (MTT) to MTT formazan conversion assay (Sigma-Aldrich), as described.[15] The 50% Lethal Dose (LD_{50}) was calculated using Prism 5.0 software (Graph-Pad Software Inc.).[15]

2.2 Virus Preparation, Infection and Titration

The influenza A virus (IAV) strain A/PuertoRico/8/1934(H1N1) (PR8), a prototype strain of the H1N1 IAV subtype, was utilized for this study. PR8 virus was grown in the allantoic cavity of 10-day-old embryonated eggs; virus titer was determined by plaque-assay, as described previously.[16]

Confluent cell monolayers were mock-infected or infected with PR8 virus for 1h at 37°C at a multiplicity of infection (MOI) of 5 HAU (hemagglutinating units)/10^5 cells, as described.[16] After the adsorption period, the viral inoculum was removed and, after repeated washings, cells were treated with different concentrations (0.1, 1, 5, 10 and 50 µg/ml) of each compound or vehicle and maintained at 37°C in RPMI-1640 medium containing 2% FCS for 24 h; in parallel, cell viability was determined in mock-infected cells by MTT-assay, as described above. Each compound was tested in duplicate, triplicate or quadruplicate. Virus yield was determined 24 h post infection (p.i.) by HA-titration, as described.[17] Statistical analysis of virus yield in HAU was performed using Student's *t* test for unpaired data. Compounds IC_{50} (50% inhibitory concentration) and IC_{90} (90% inhibitory concentration) were calculated using Prism 5.0 software.

2.3 DMPK Analysis

DMPK properties were assessed using a high-throughput platform at AstraZeneca (United Kingdom). Methodologies for the determination of $logD_{7.4}$, aqueous solubility, plasma protein binding, microsomal clearance, and hepatocyte clearance have been reported previously.[18]

3. NMR Spectra of Compounds 10a-m, 11a-k, 15a-h

***N*-(5-Chlorothiazol-2-yl)-2-hydroxy-3-methylbenzamide (10a)**

^{1}H NMR (400 MHz, $CDCl_3$)

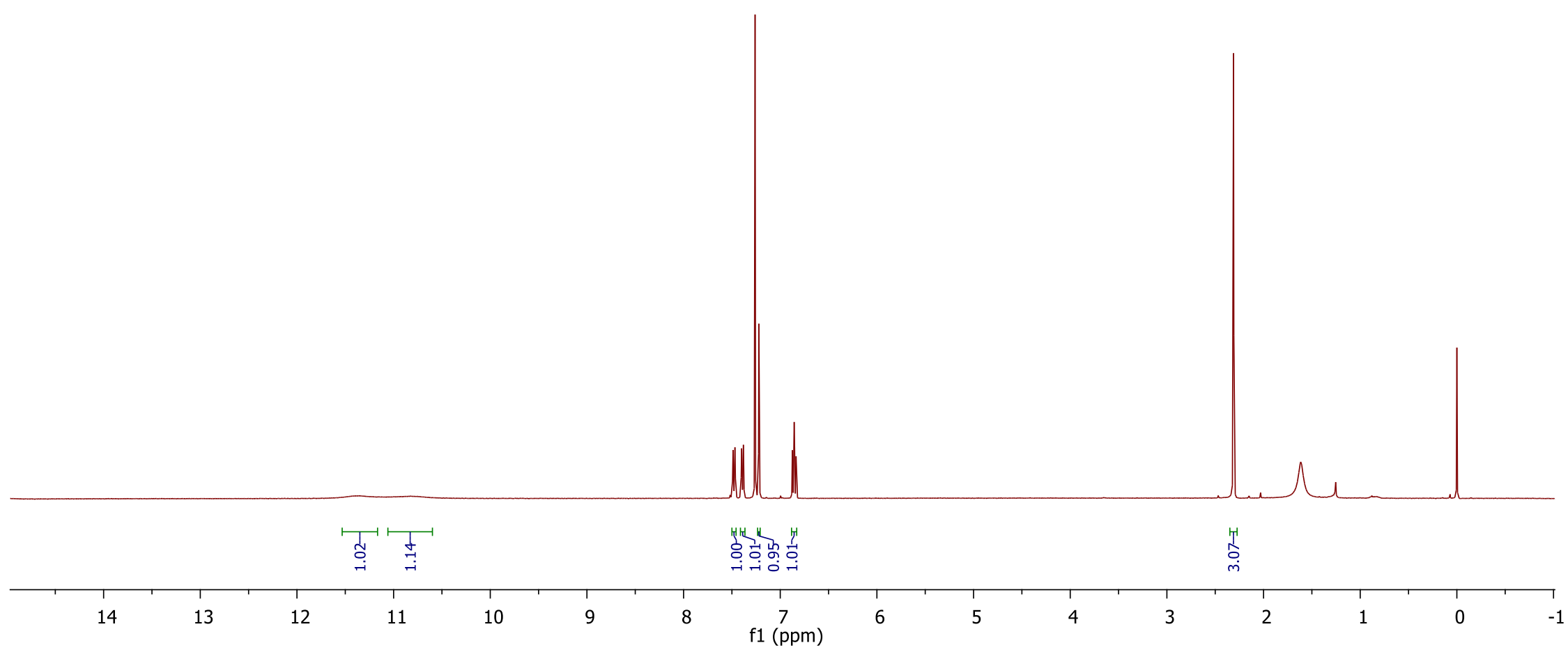

^{13}C NMR (101 MHz, $CDCl_3$)

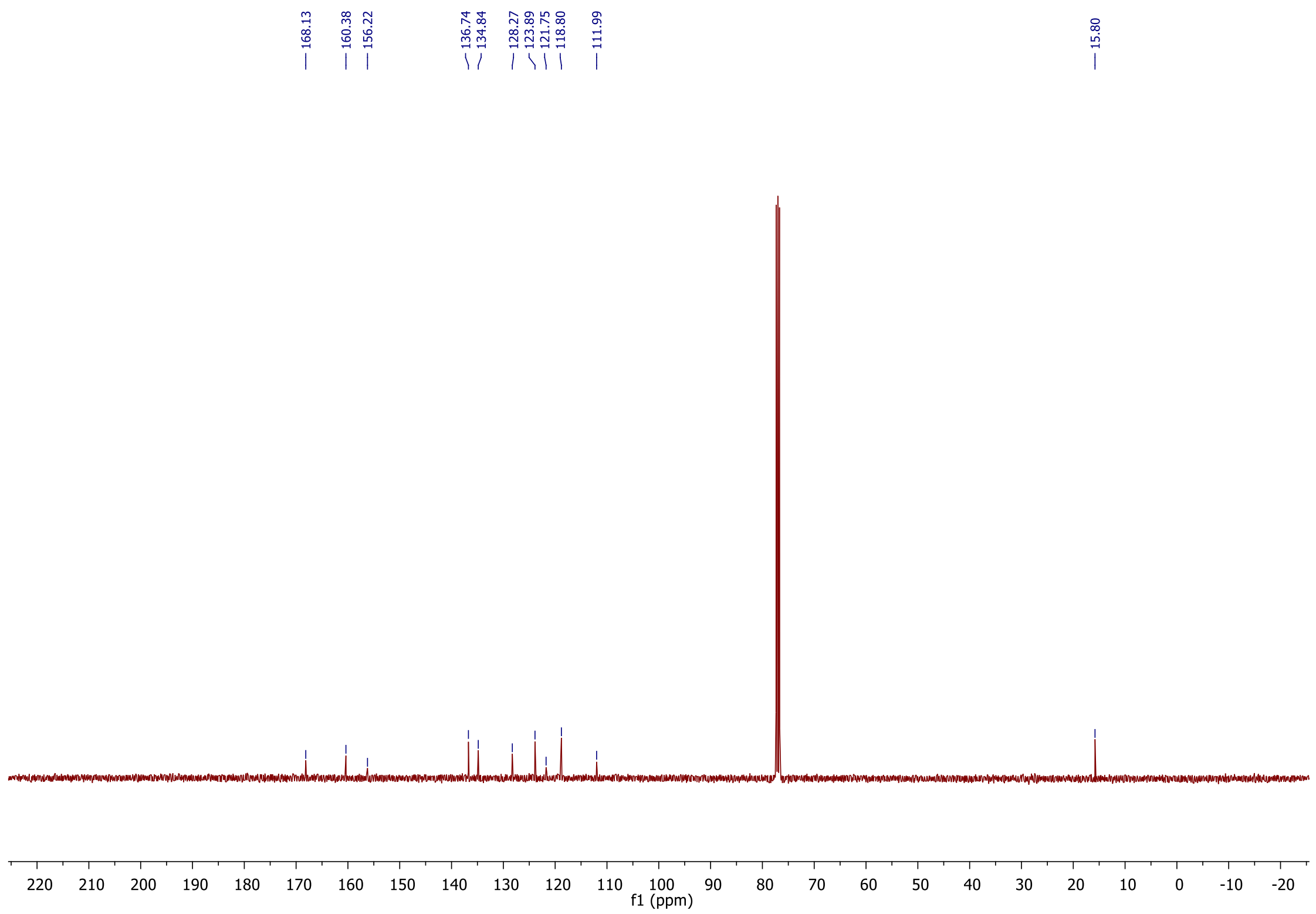

***N*-(5-Chlorothiazol-2-yl)-2-hydroxy-3-isopropylbenzamide (10b)**

^{1}H NMR (400 MHz, $CDCl_3$)

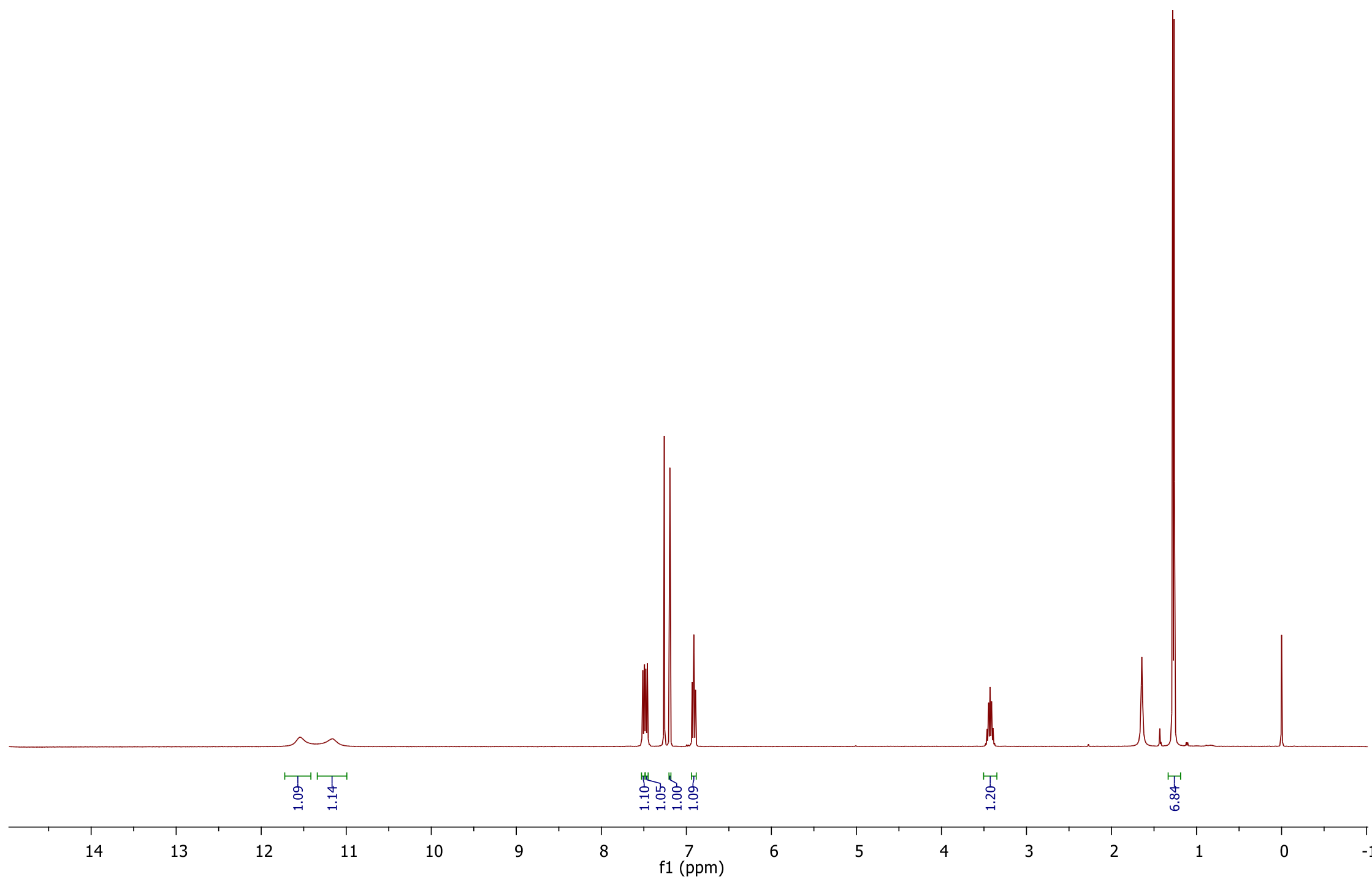

^{13}C NMR (101 MHz, $CDCl_3$)

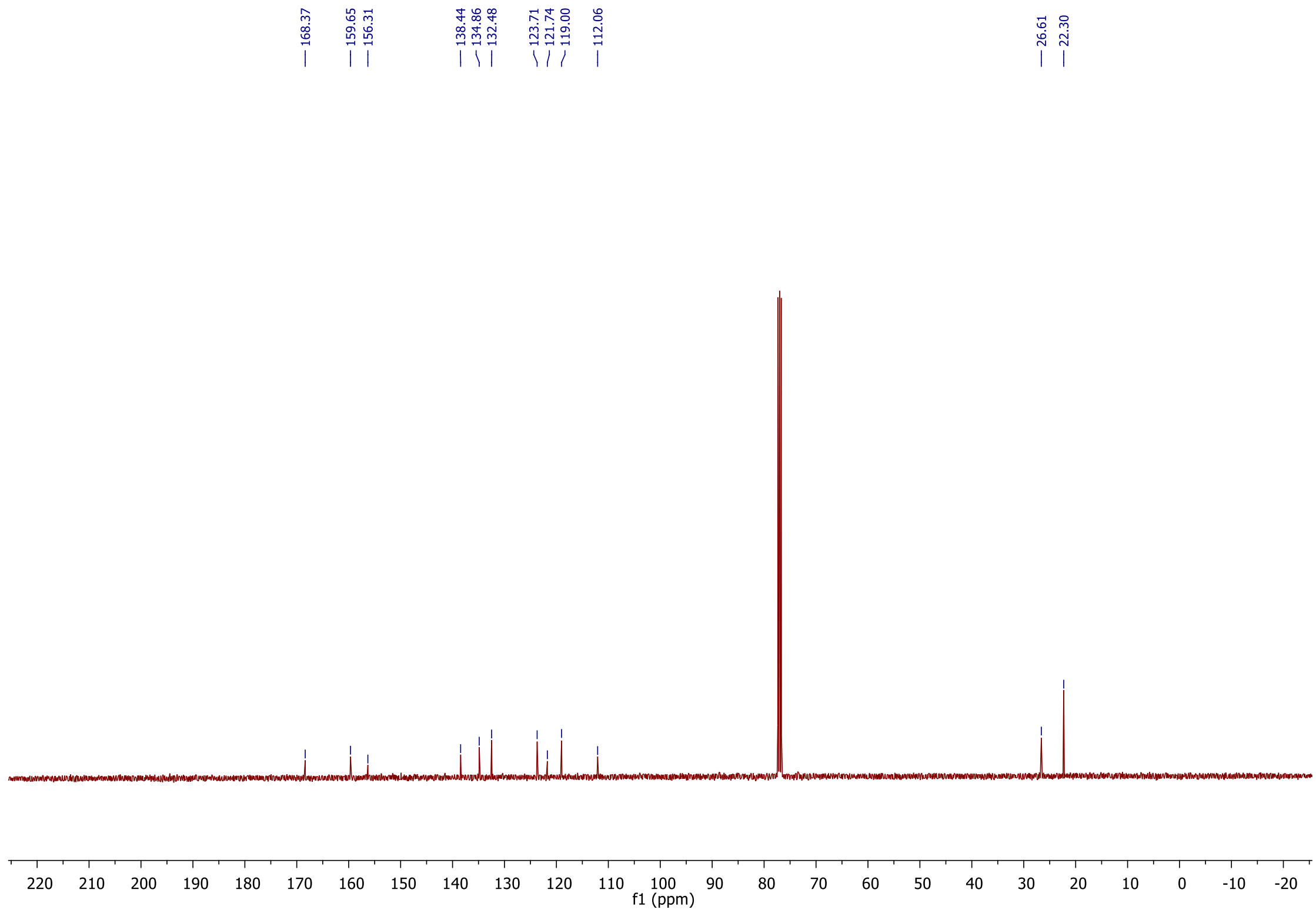

***N*-(5-Chlorothiazol-2-yl)-3-fluoro-2-hydroxybenzamide (10c)**

^{1}H NMR (400 MHz, $(CD_3)_2SO$)

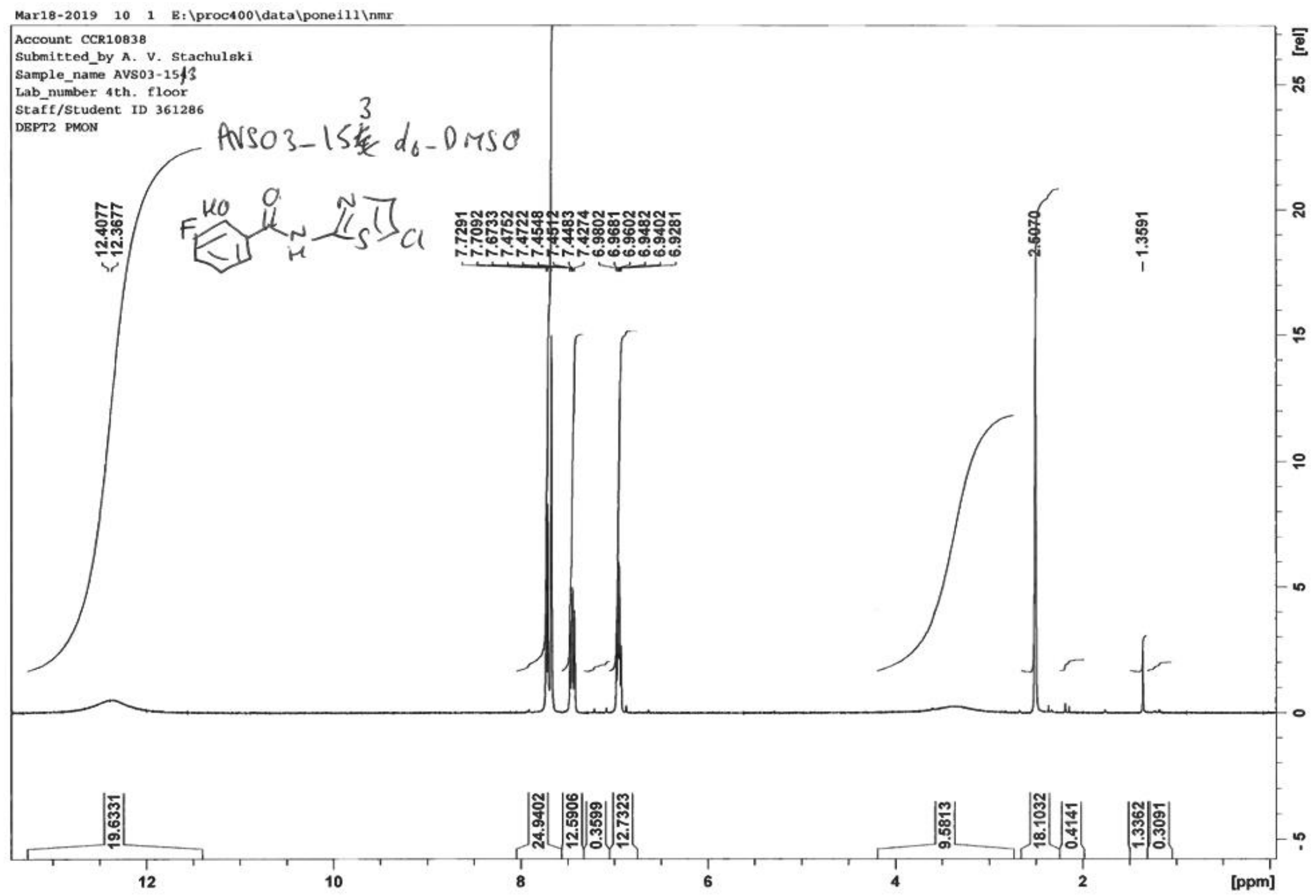

^{13}C NMR (101 MHz, $(CD_3)_2SO$)

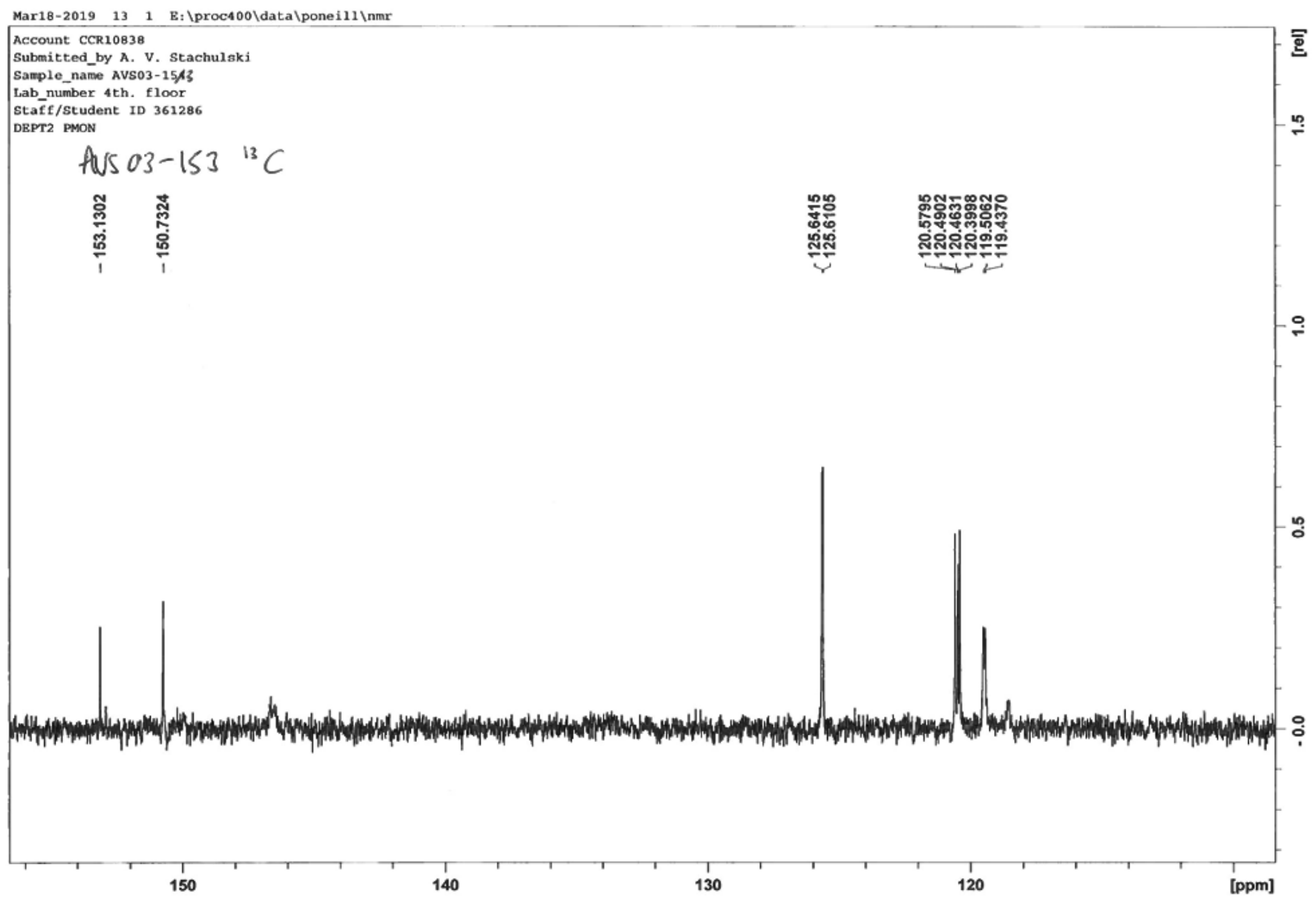

$^{19}F\{^1H\}$ NMR (376 MHz, $(CD_3)_2SO$)

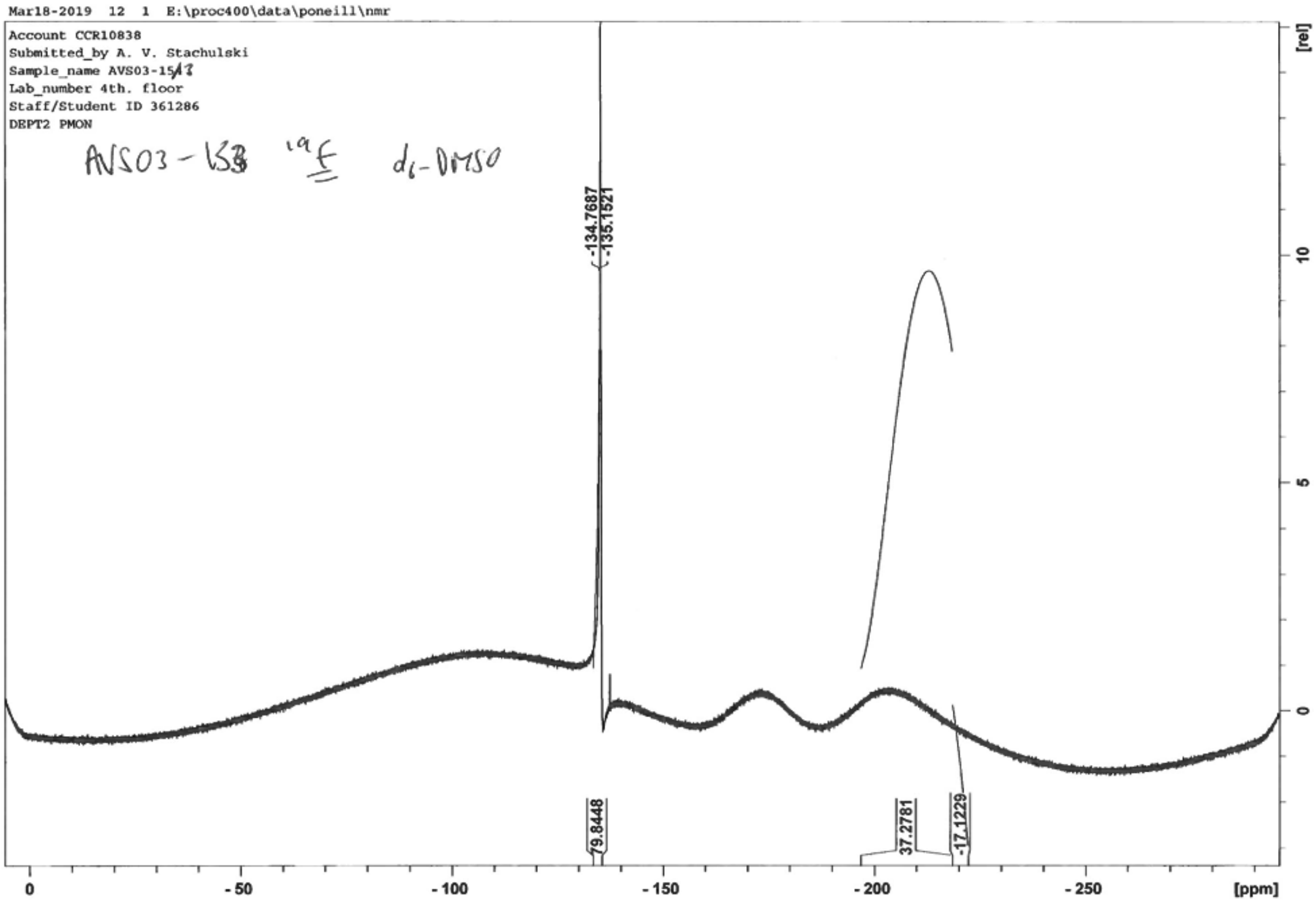

N-(5-Chlorothiazol-2-yl)-4-fluoro-2-hydroxybenzamide (10d)

^1H NMR (400 MHz, $(CD_3)_2SO$)

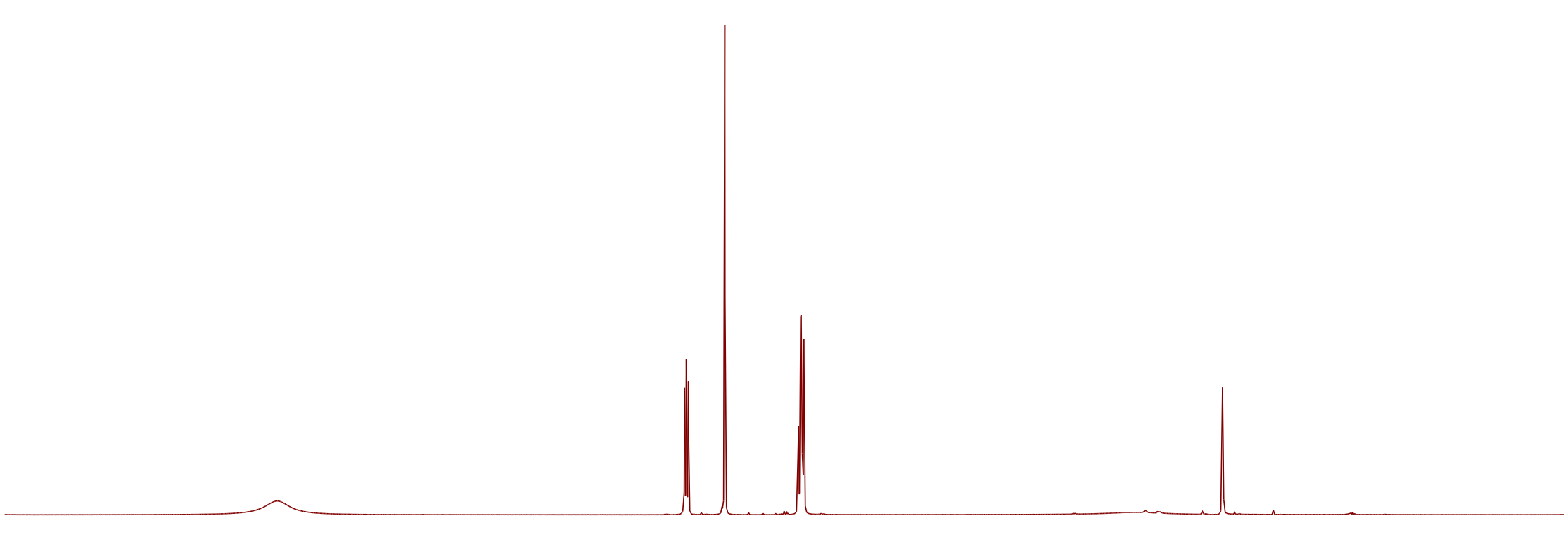

^{13}C NMR (101 MHz, $(CD_3)_2SO$)

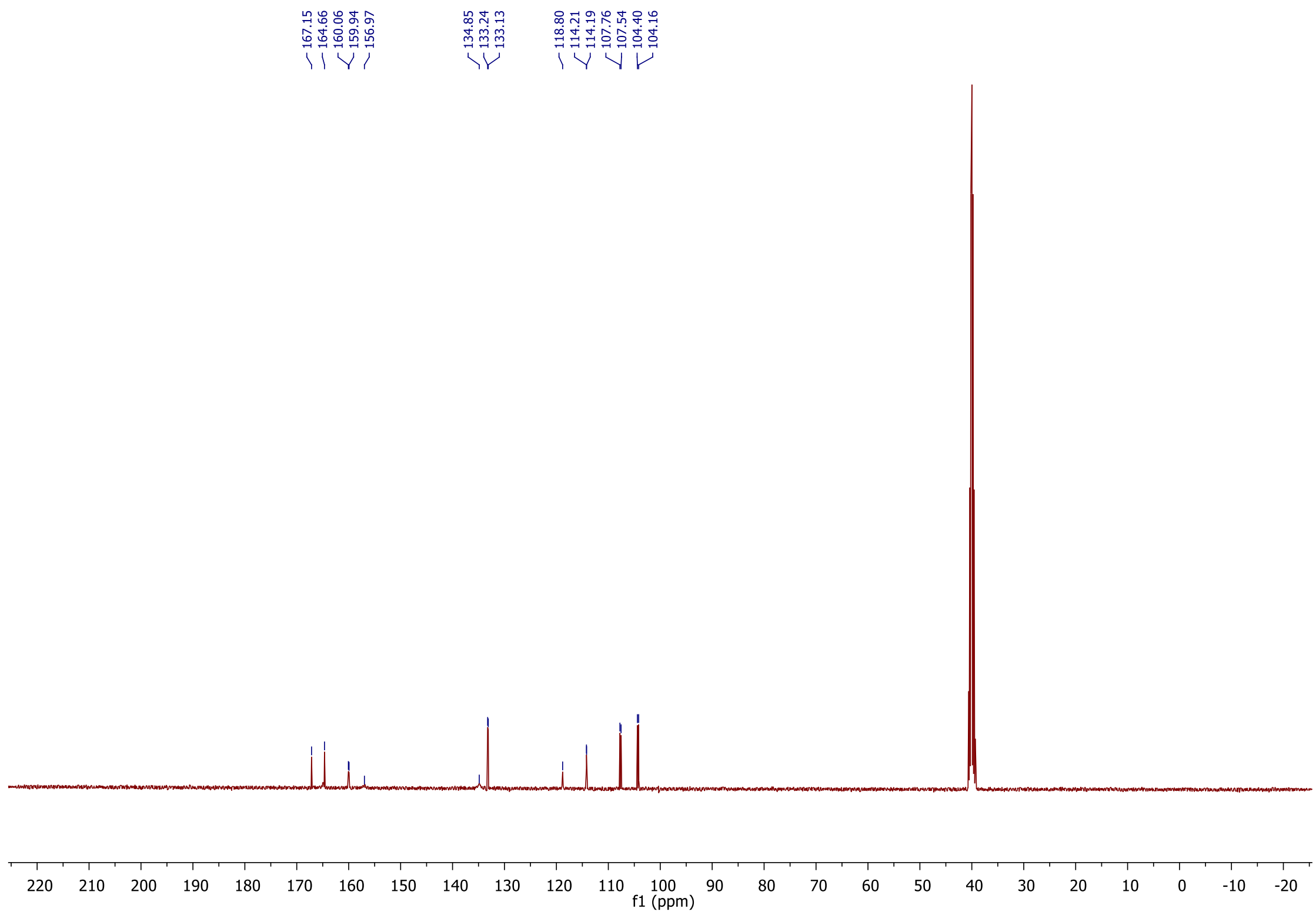

$^{19}F\{^1H\}$ NMR (376 MHz, $(CD_3)_2SO$)

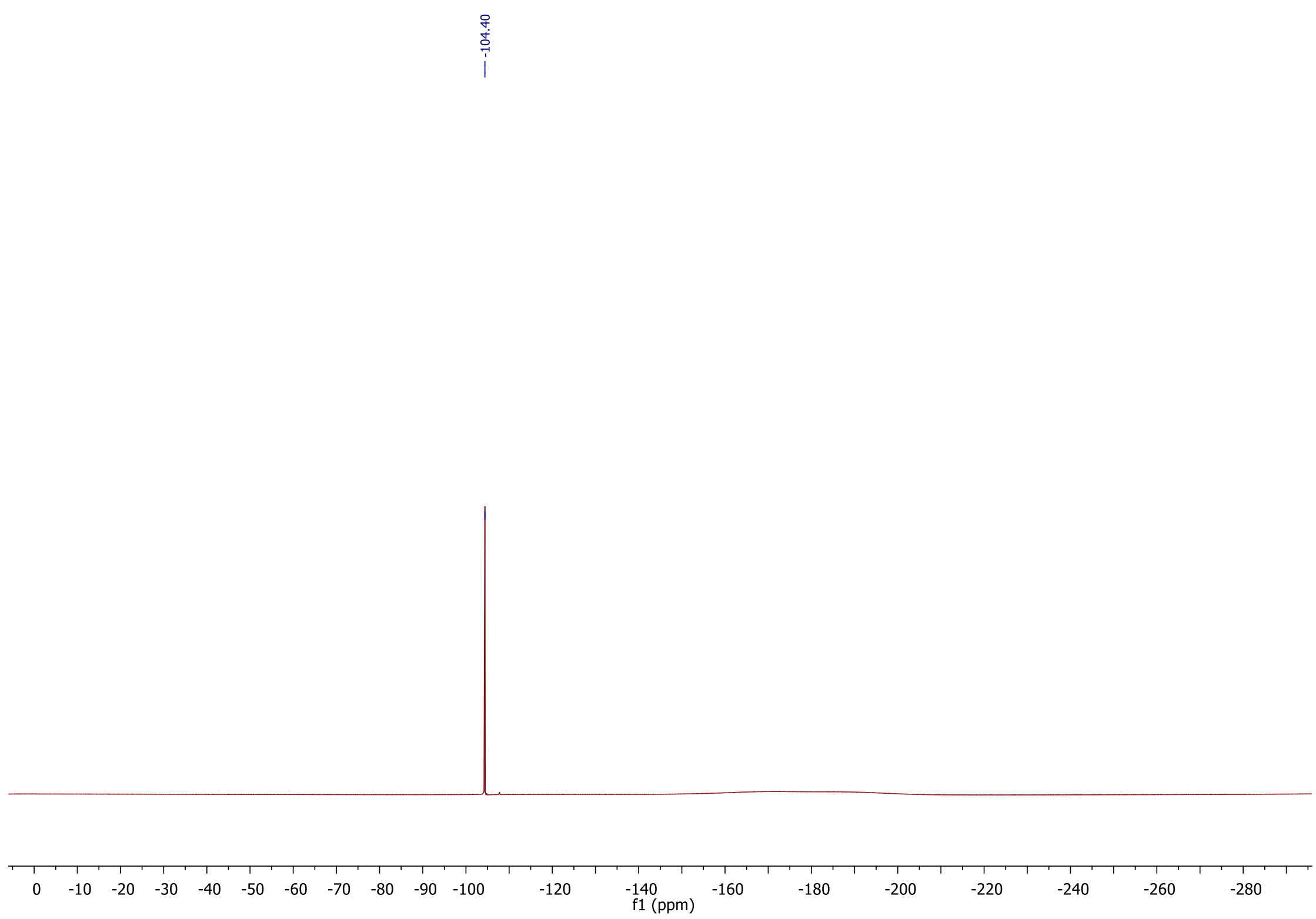

***N*-(5-Chlorothiazol-2-yl)-5-fluoro-2-hydroxybenzamide (10e)**

^1H NMR (400 MHz, $(CD_3)_2SO$)

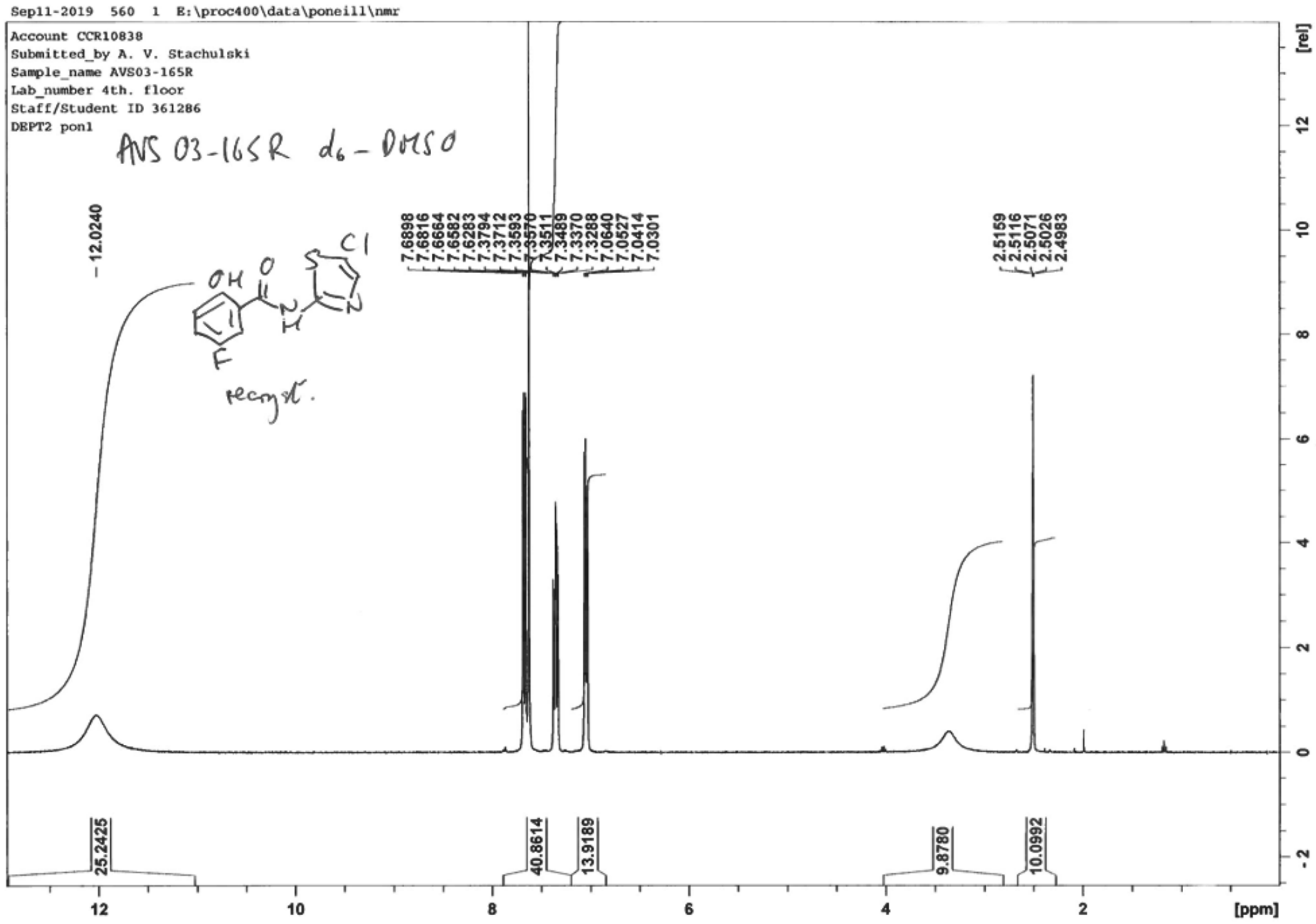

^{13}C NMR (101 MHz, $(CD_3)_2SO$)

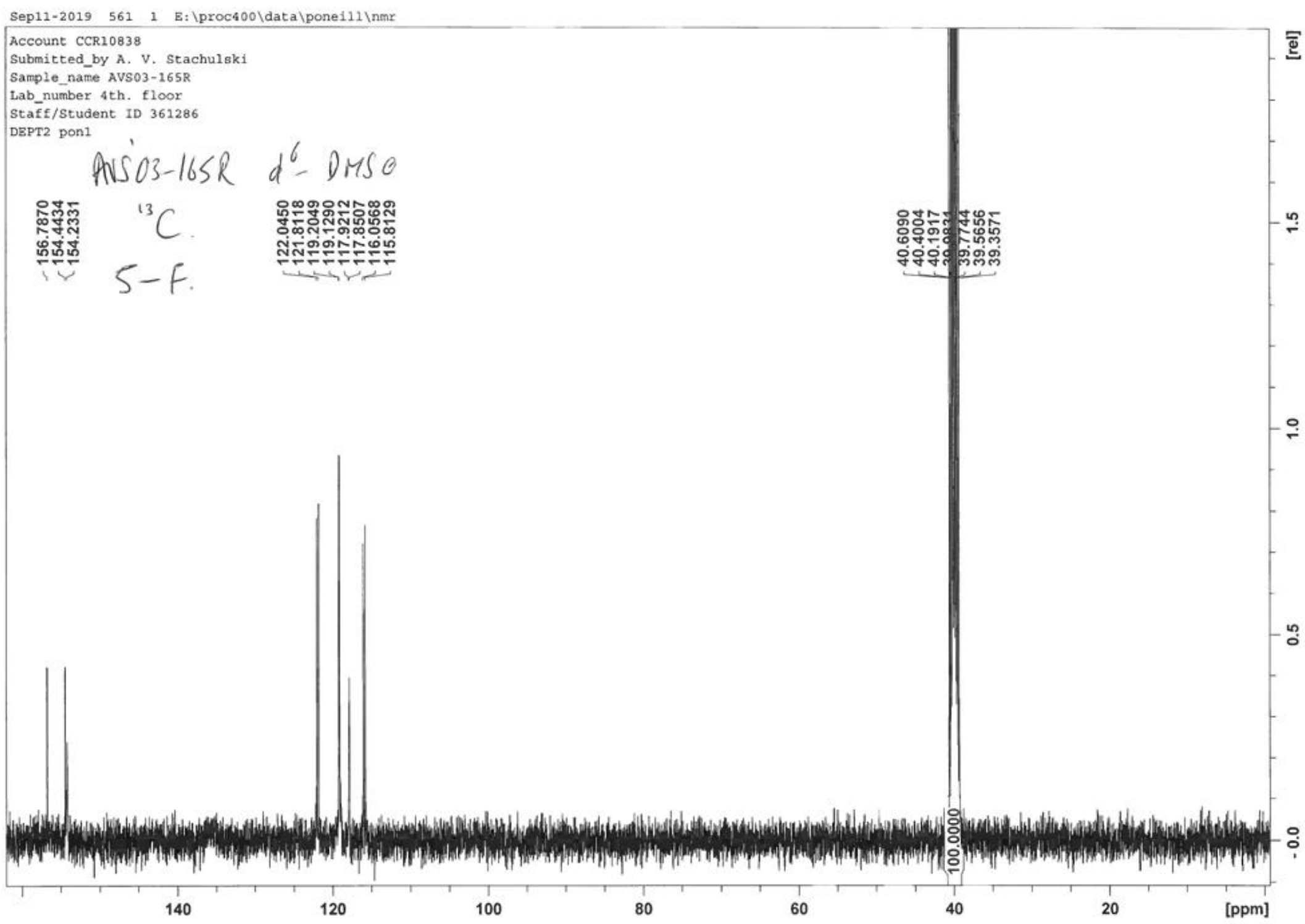

^{19}F NMR (376 MHz, $(CD_3)_2SO$)

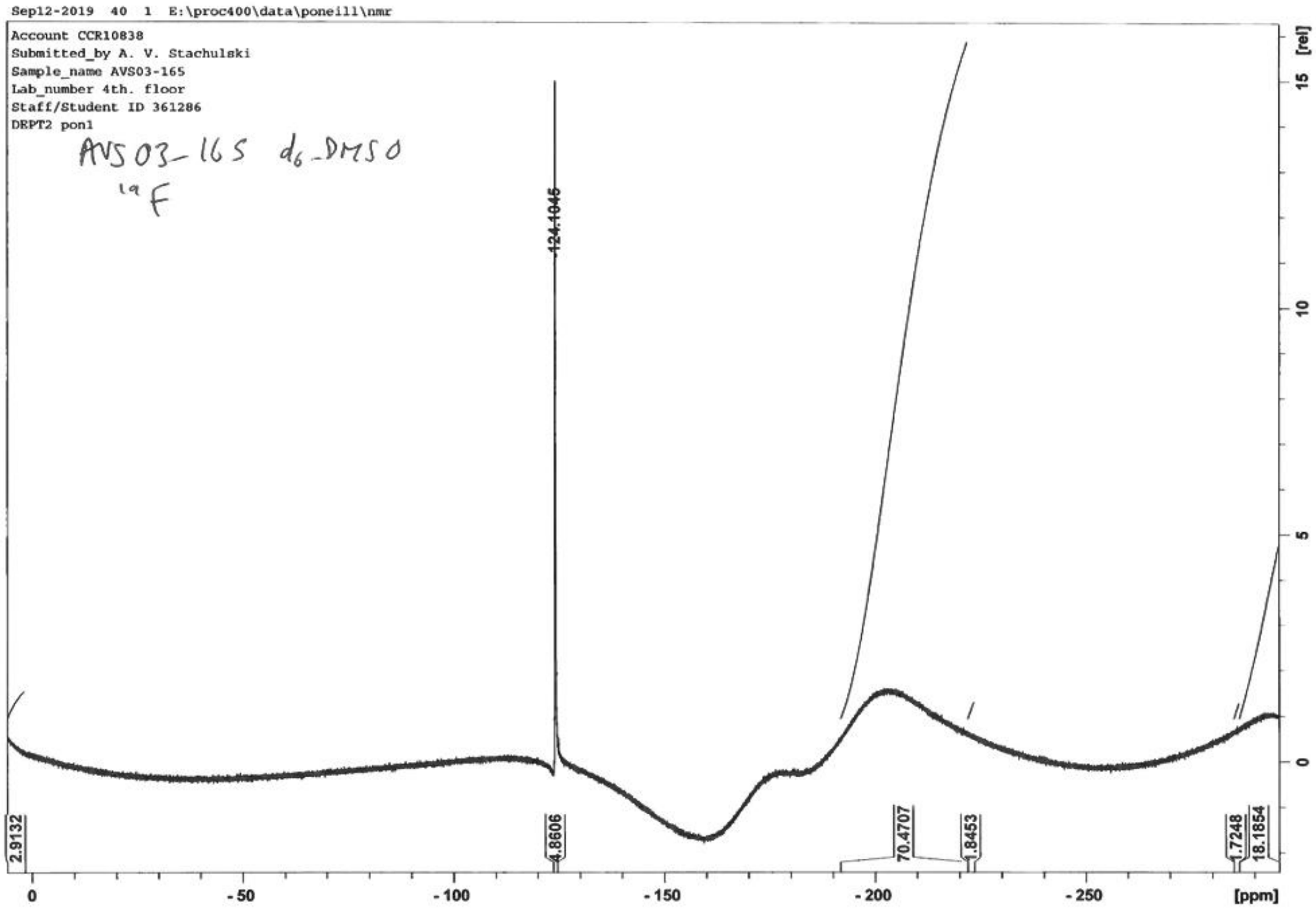

N-(5-Chlorothiazol-2-yl)-6-fluoro-2-hydroxybenzamide (10f)

^{1}H NMR (400 MHz, $(CD_3)_2SO$)

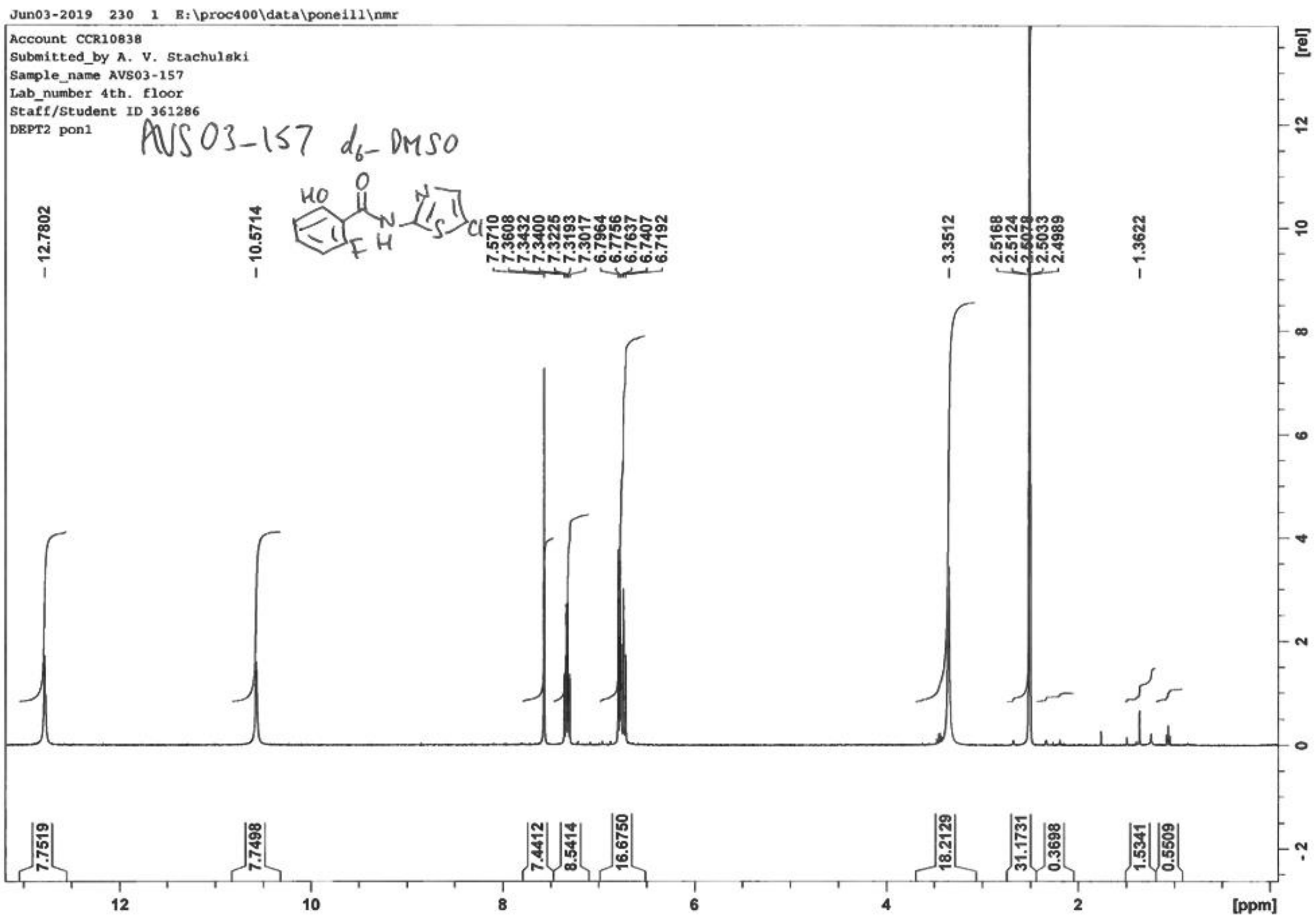

^{13}C NMR (101 MHz, $(CD_3)_2SO$)

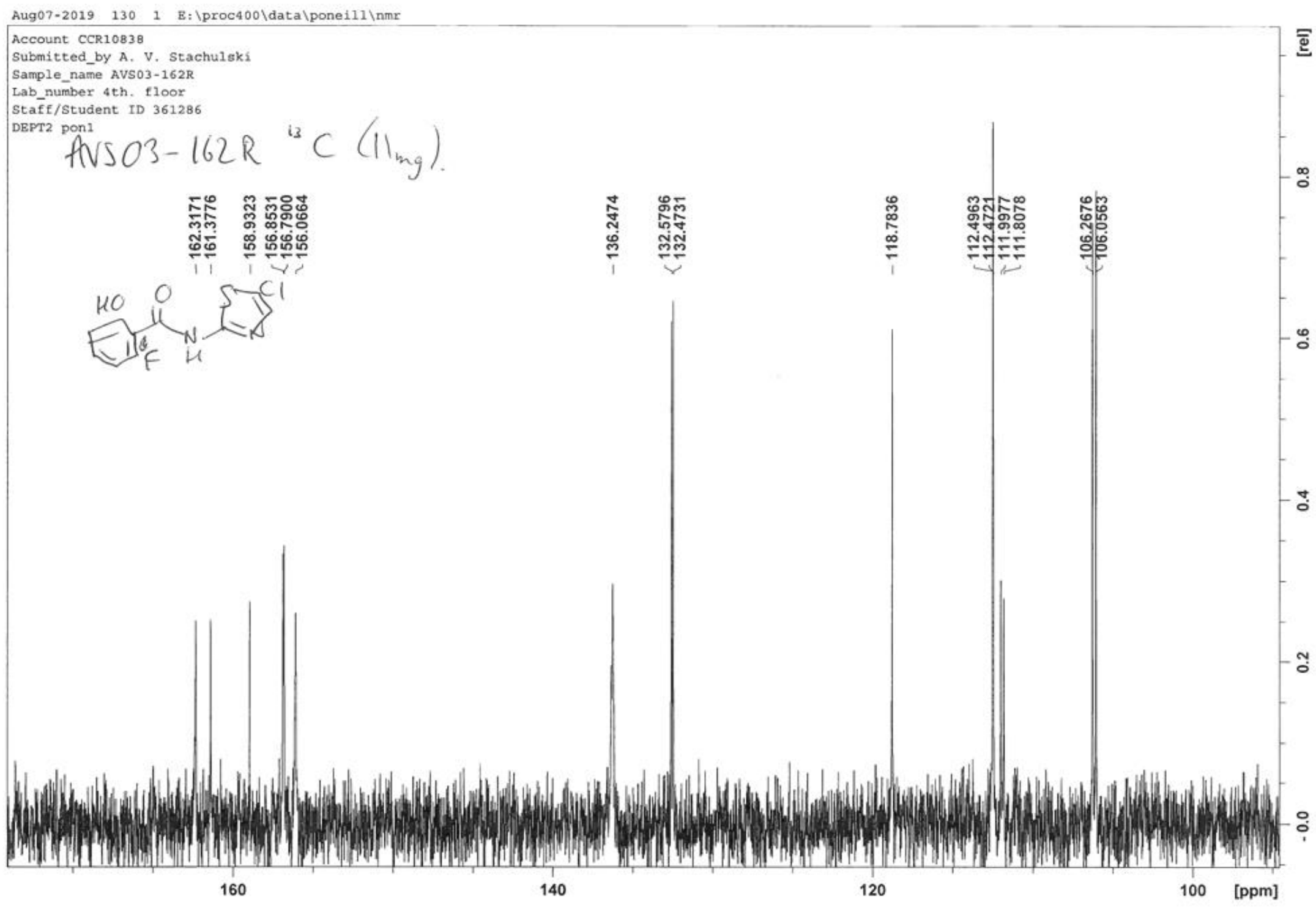

^{19}F{^{1}H} NMR (376 MHz, $(CD_3)_2SO$)

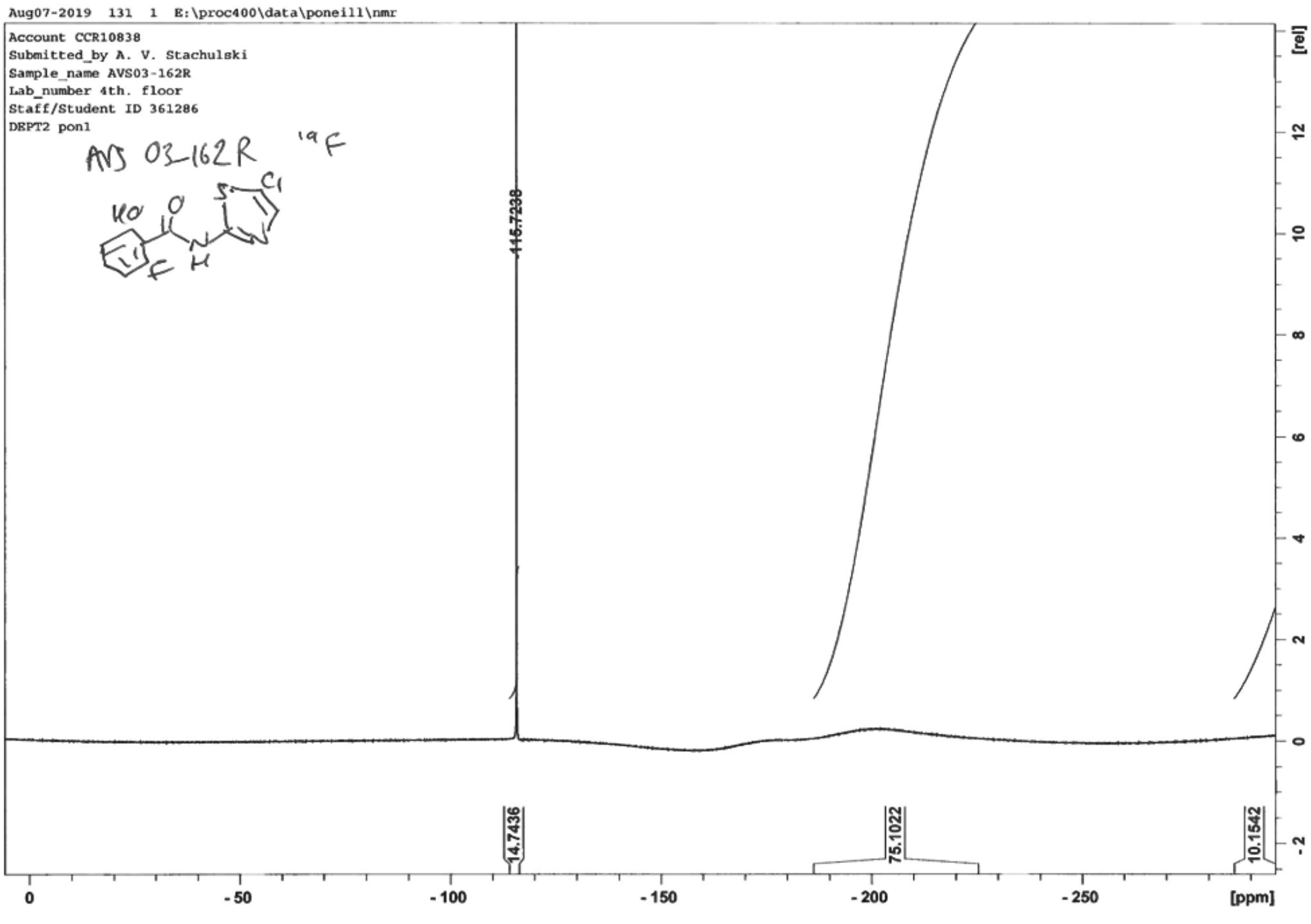

3-Chloro-*N*-(5-chlorothiazol-2-yl)-2-hydroxybenzamide (10g)

^{1}H NMR (400 MHz, $(CD_3)_2SO$)

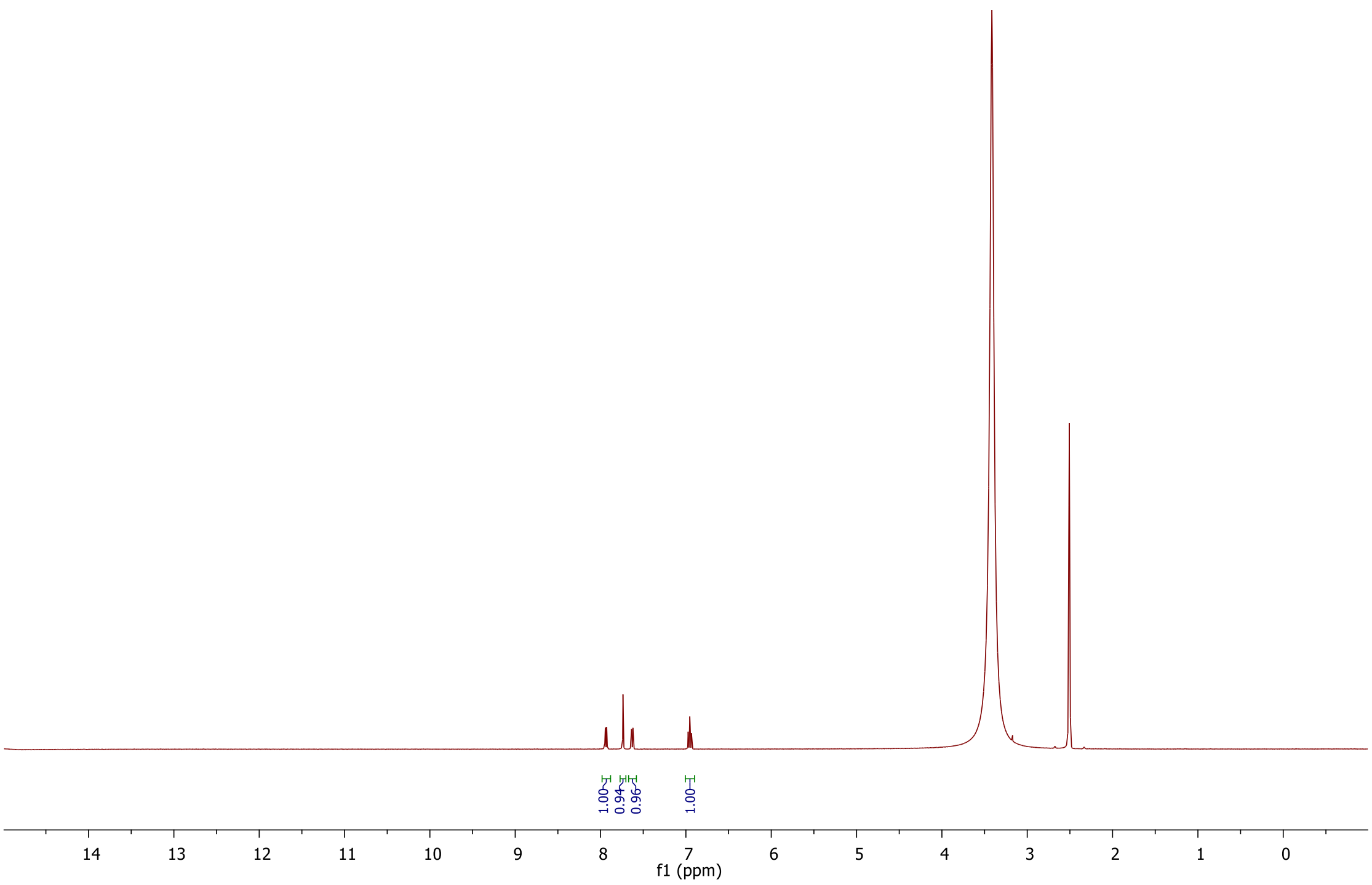

^{13}C NMR (101 MHz, $(CD_3)_2SO$)

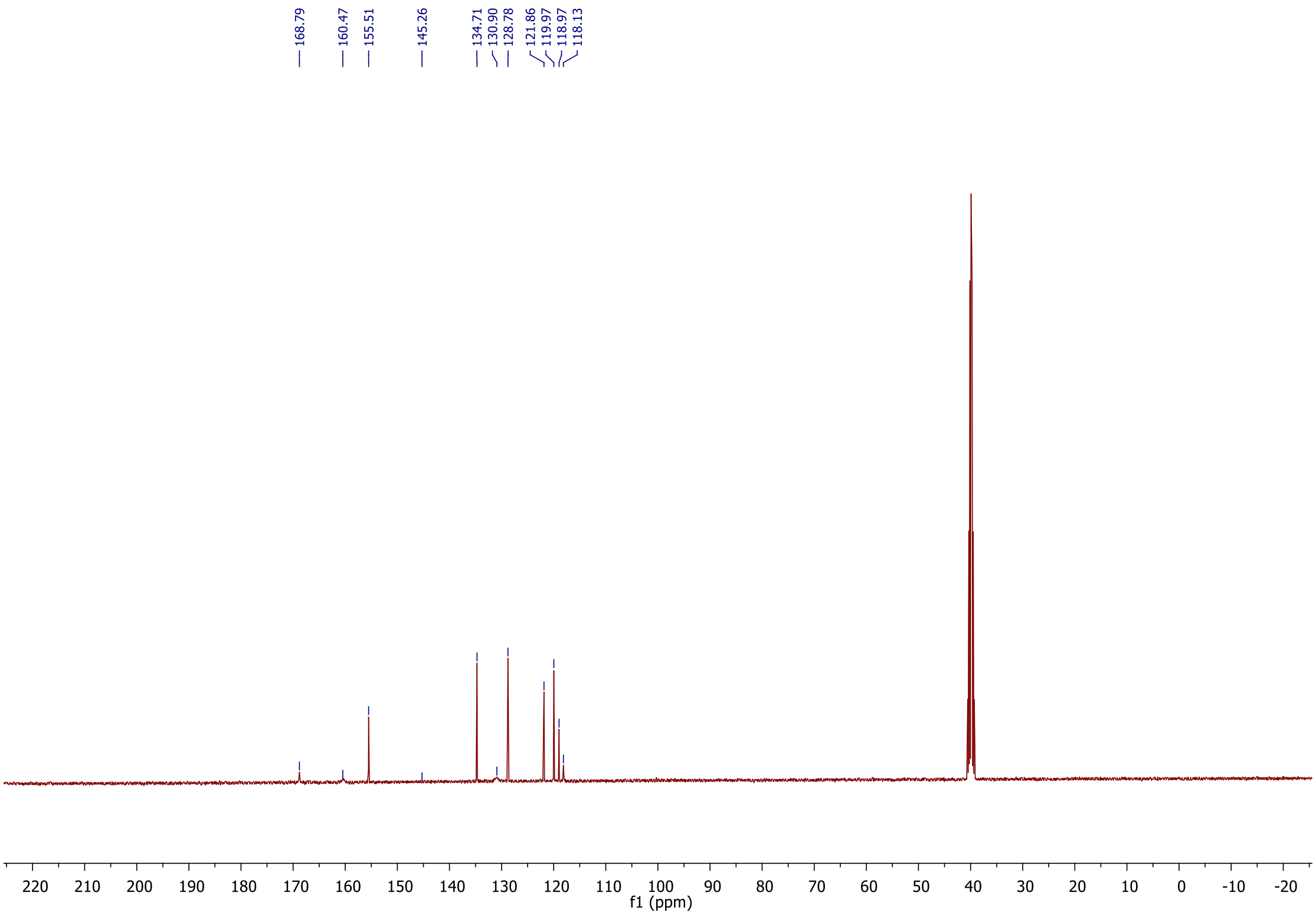

4-Chloro-*N*-(5-chlorothiazol-2-yl)-2-hydroxybenzamide (10h)

^{1}H NMR (400 MHz, $(CD_3)_2SO$)

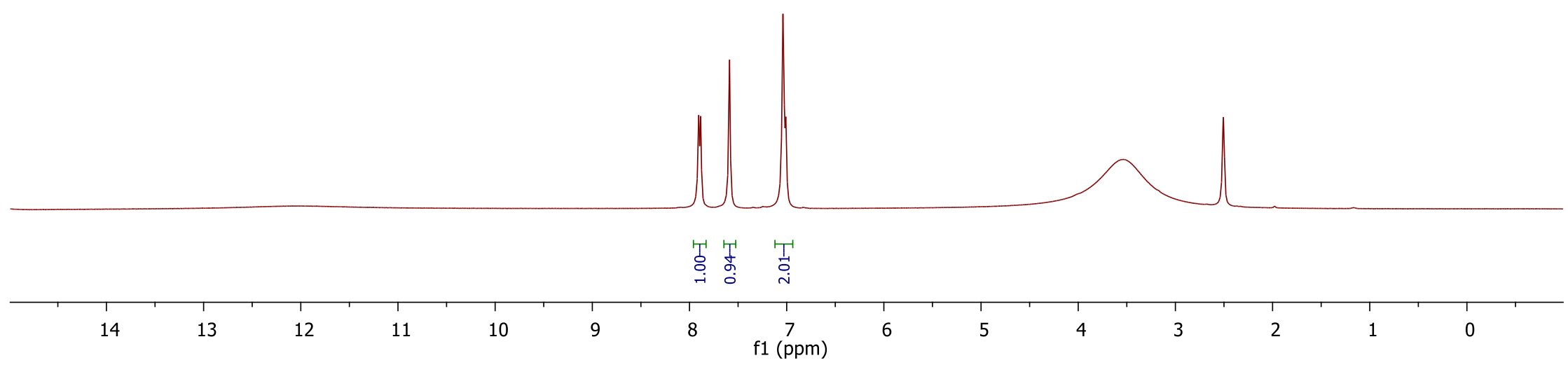

^{13}C NMR (101 MHz, $(CD_3)_2SO$)

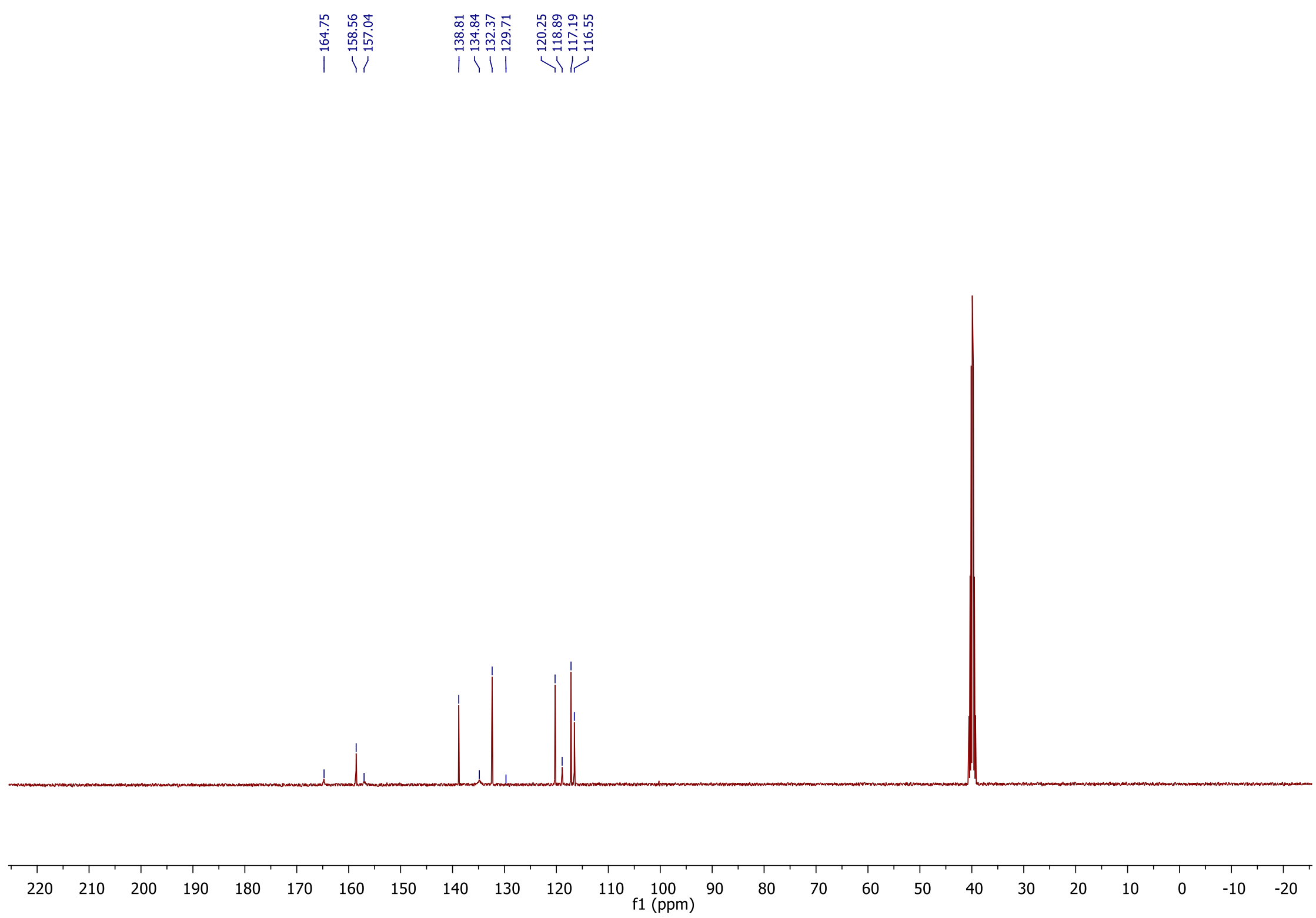

5-Chloro-*N*-(5-chlorothiazol-2-yl)-2-hydroxybenzamide (10i)

^{1}H NMR (400 MHz, $(CD_3)_2SO$)

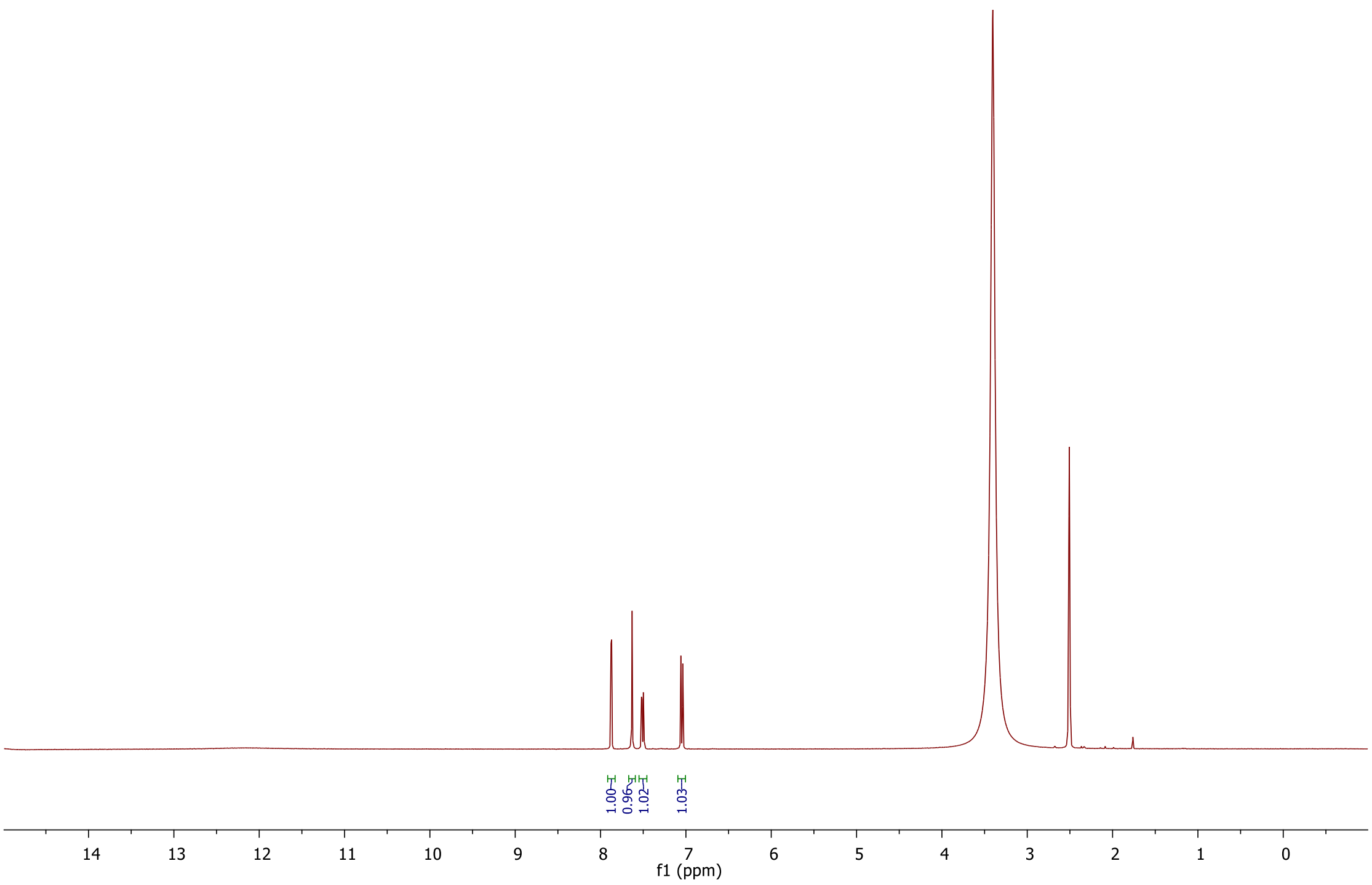

^{13}C NMR (101 MHz, $(CD_3)_2SO$)

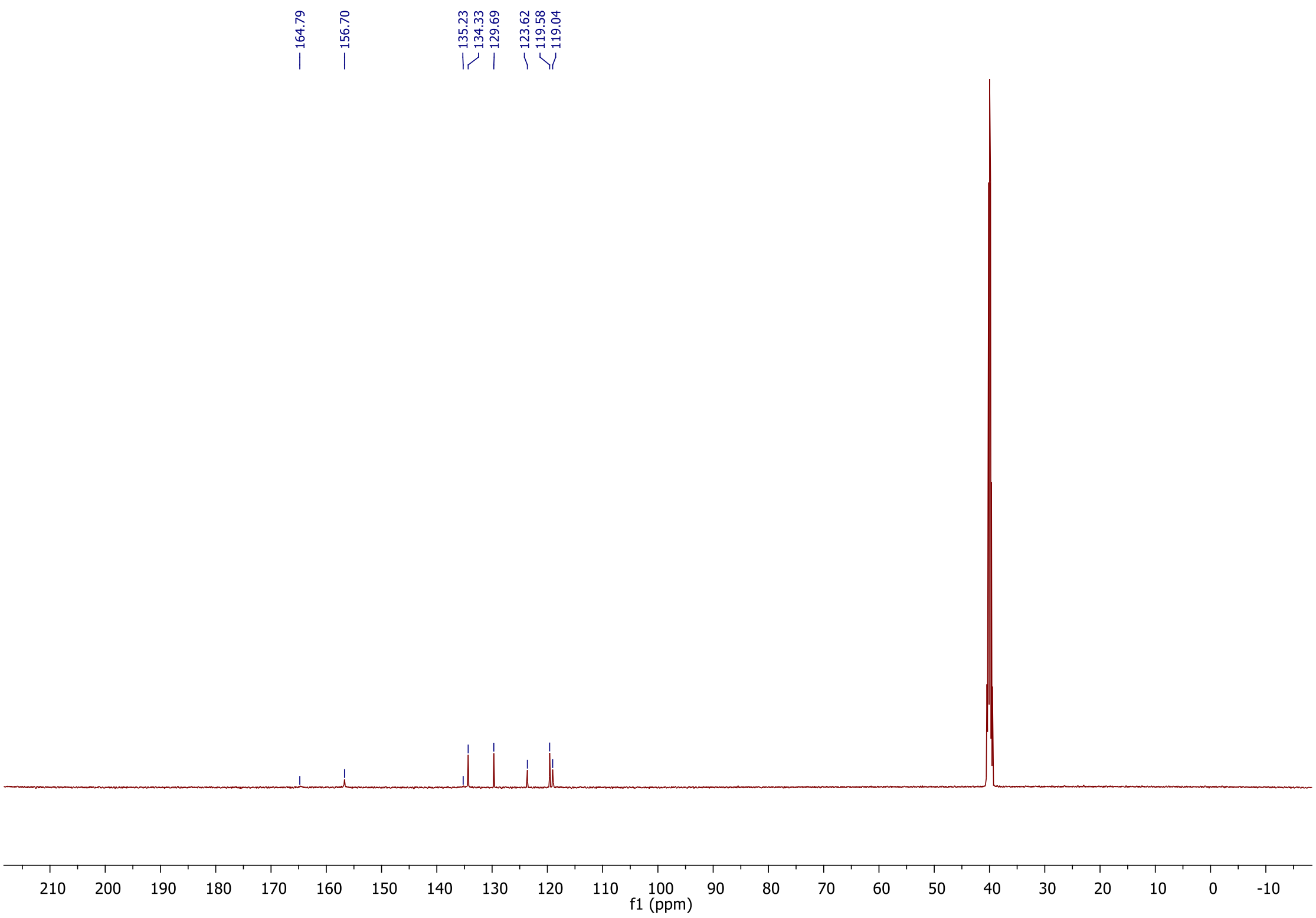

N-(5-Chlorothiazol-2-yl)-3,5-difluoro-2-hydroxybenzamide (10j)

^{1}H NMR (400 MHz, $(CD_3)_2SO$)

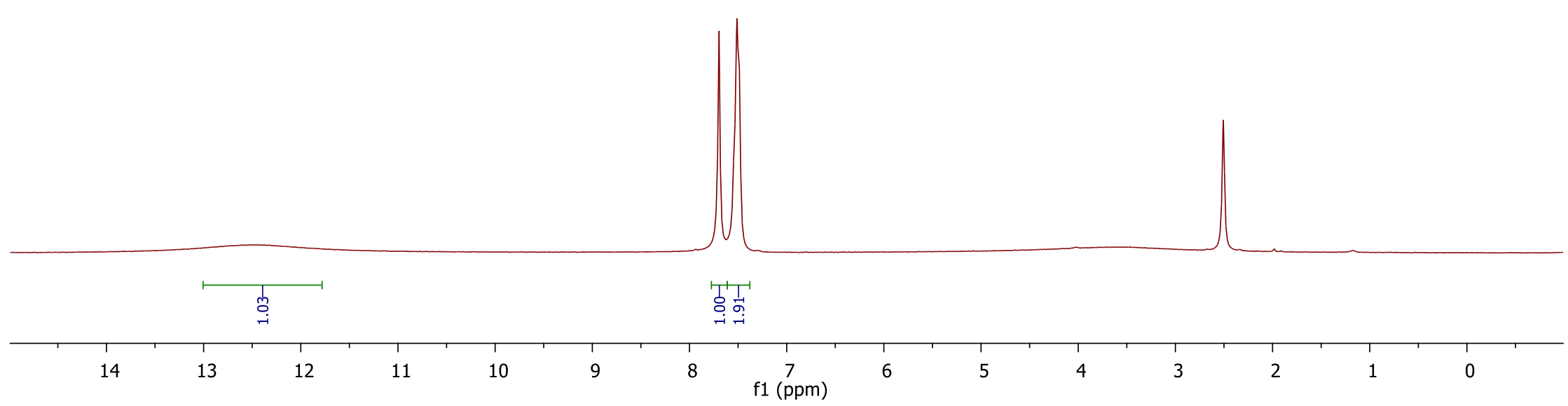

^{13}C NMR (101 MHz, $(CD_3)_2SO$)

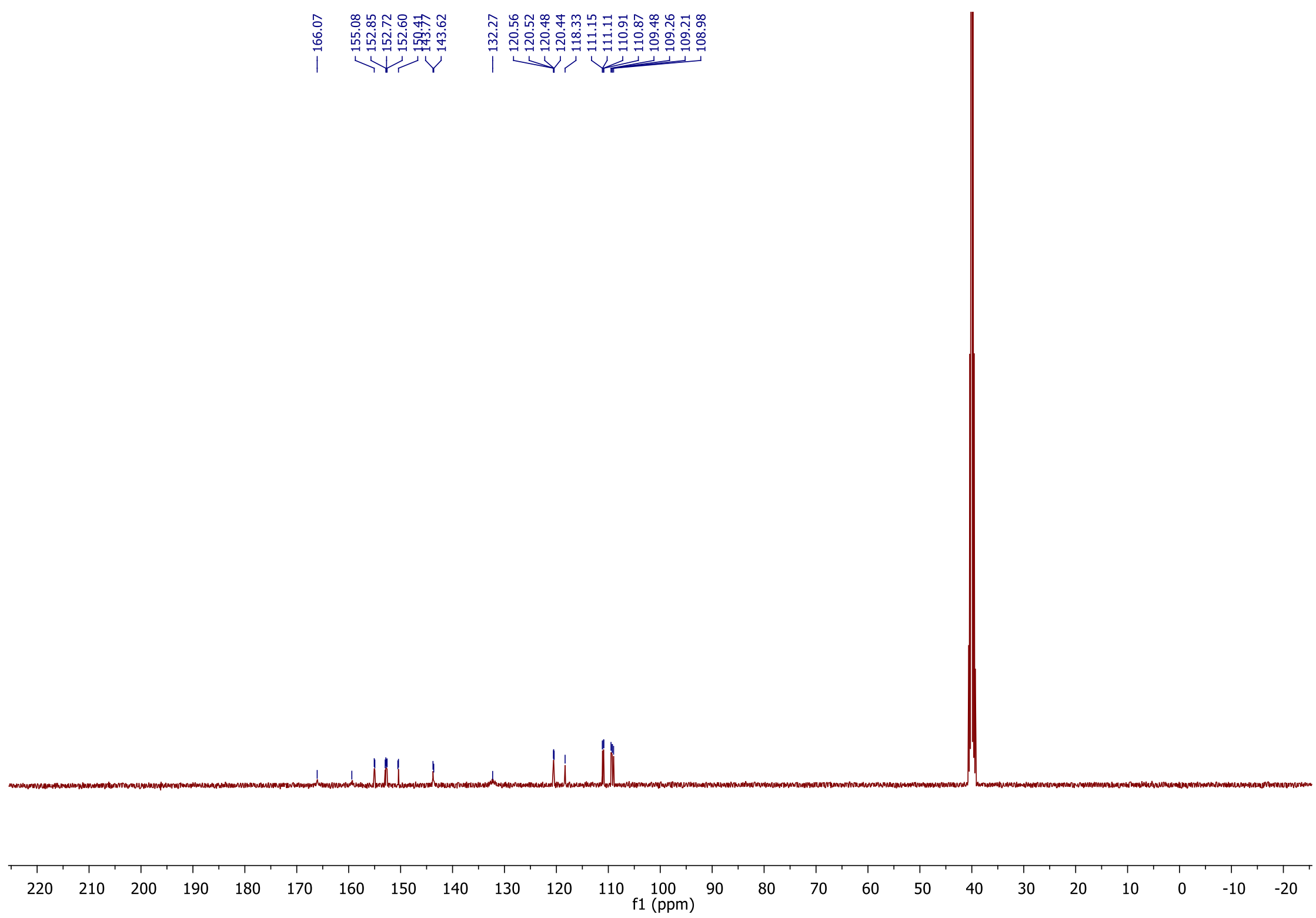

$^{19}F\{^{1}H\}$ NMR (376 MHz, $(CD_3)_2SO$)

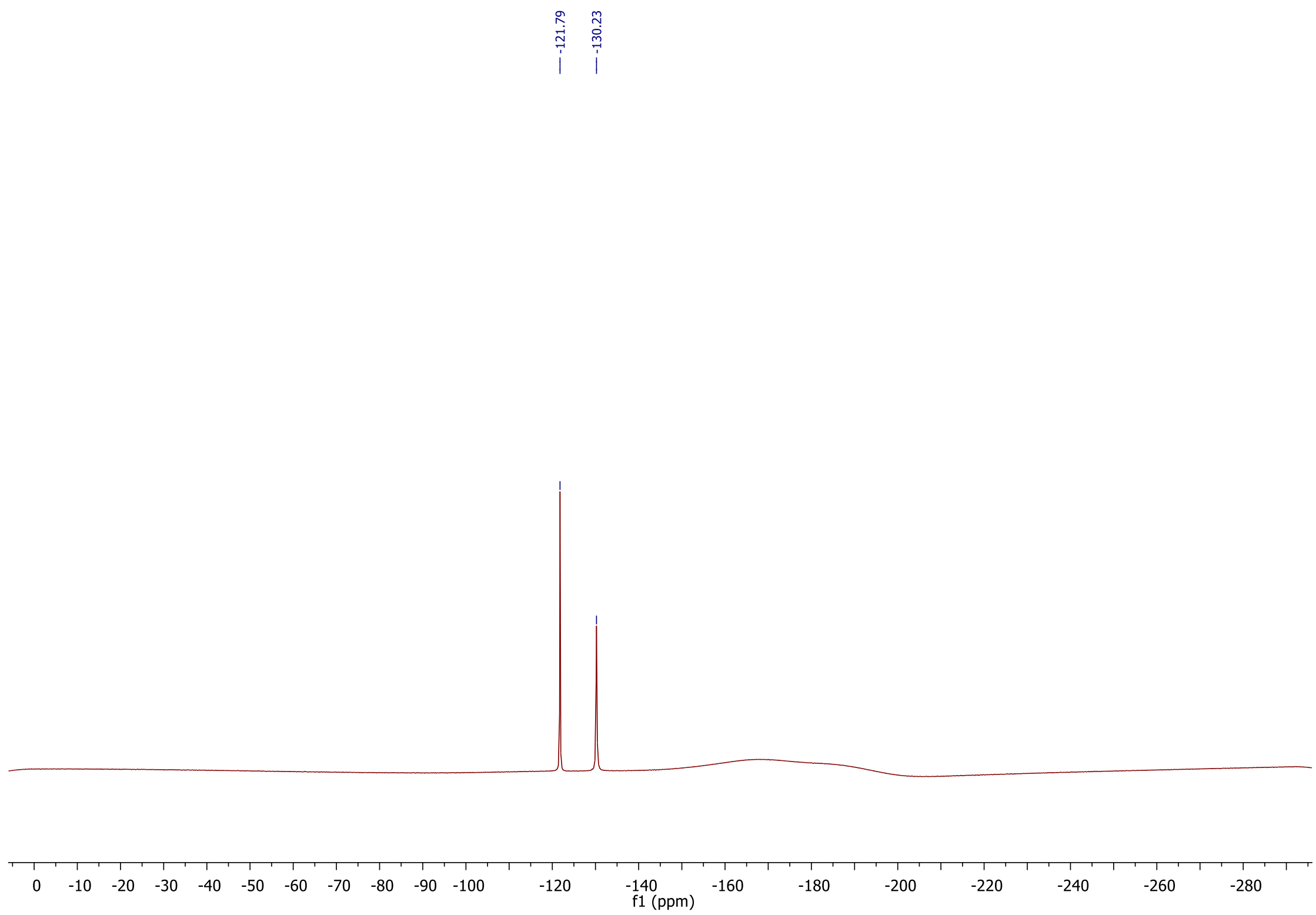

5-Chloro-*N*-(5-chlorothiazol-2-yl)-3-fluoro-2-hydroxybenzamide (10k)

^{1}H NMR (400 MHz, $(CD_3)_2SO$)

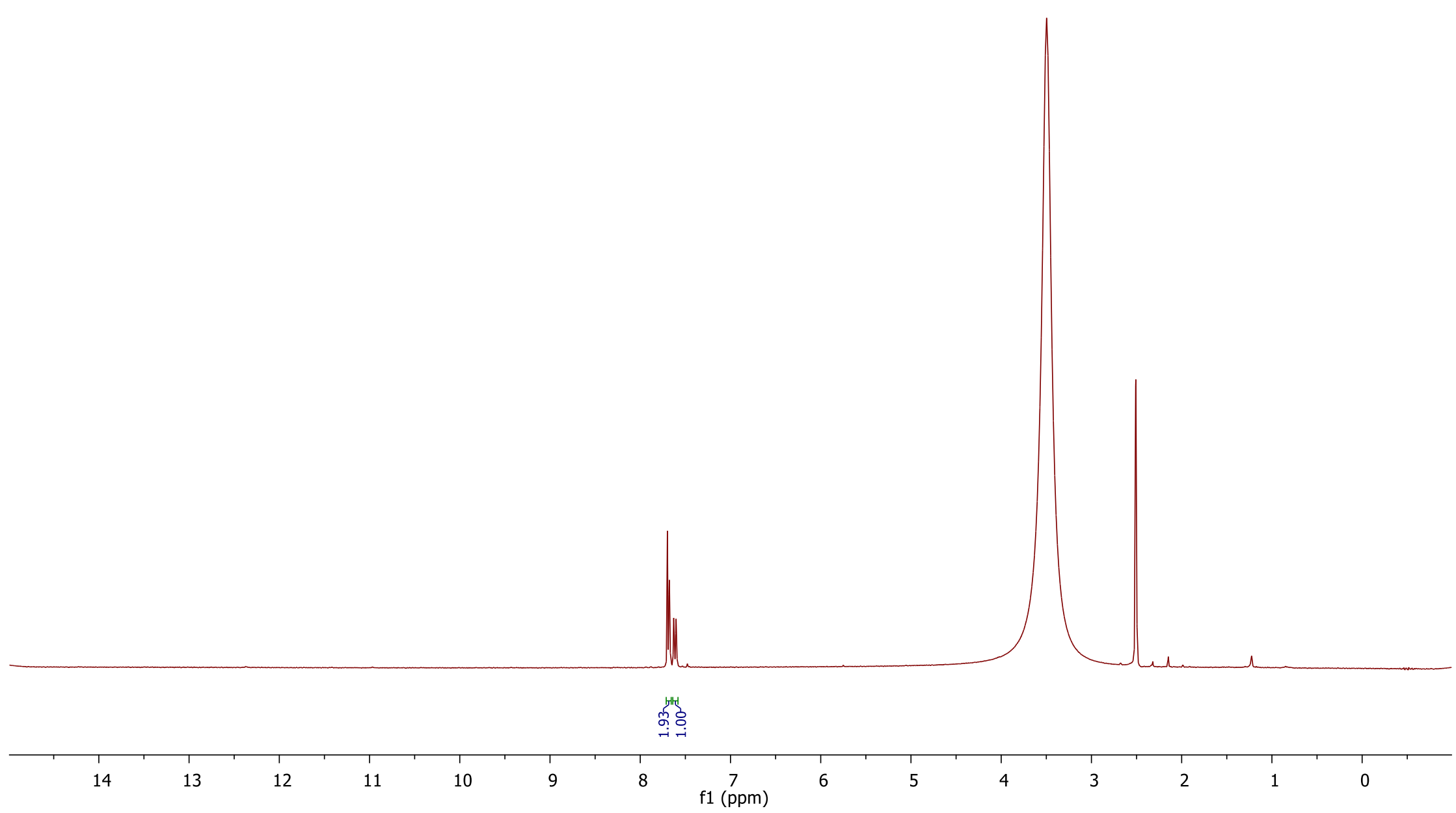

^{13}C NMR (101 MHz, $(CD_3)_2SO$)

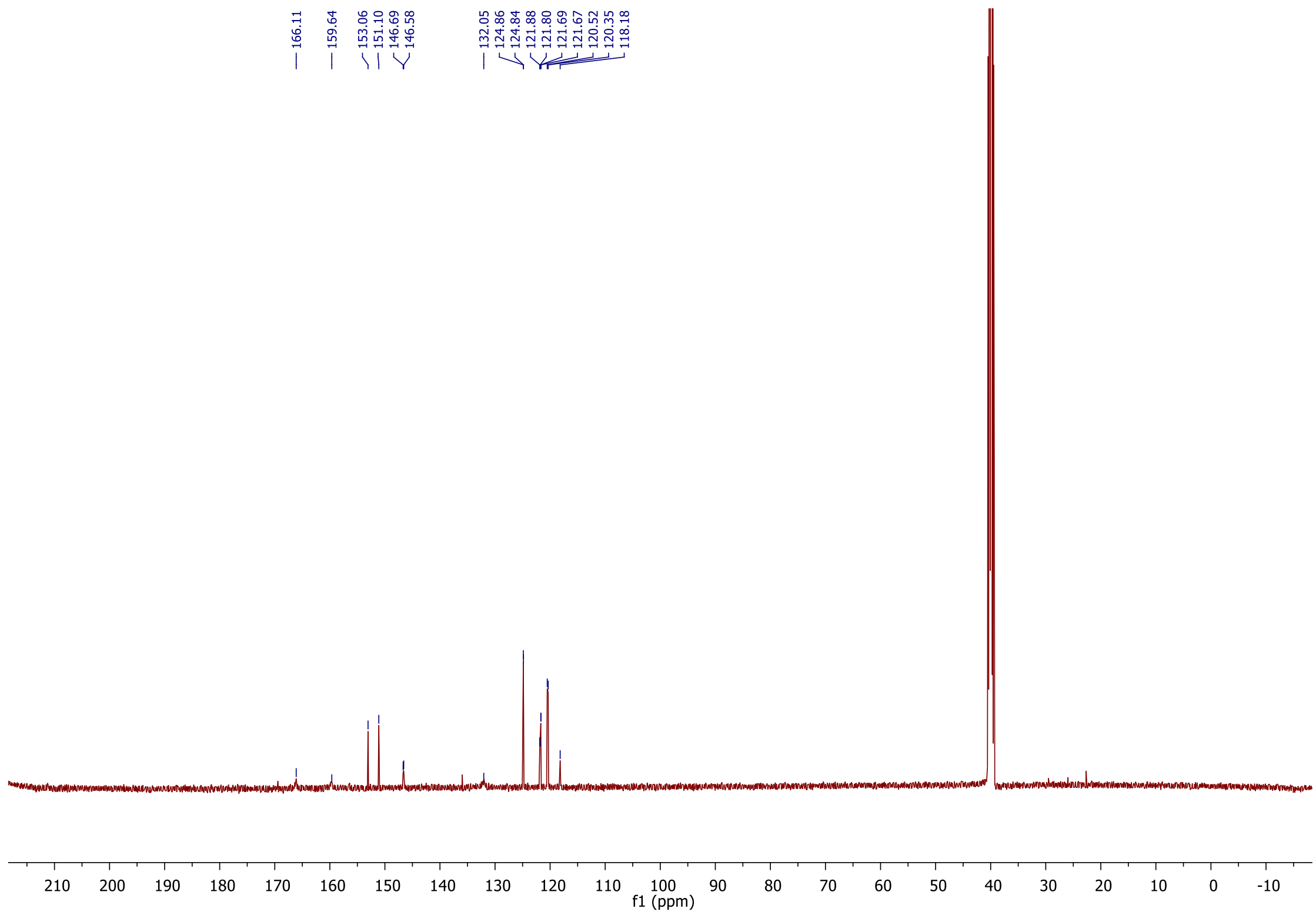

$^{19}F\{^1H\}$ NMR (376 MHz, $(CD_3)_2SO$)

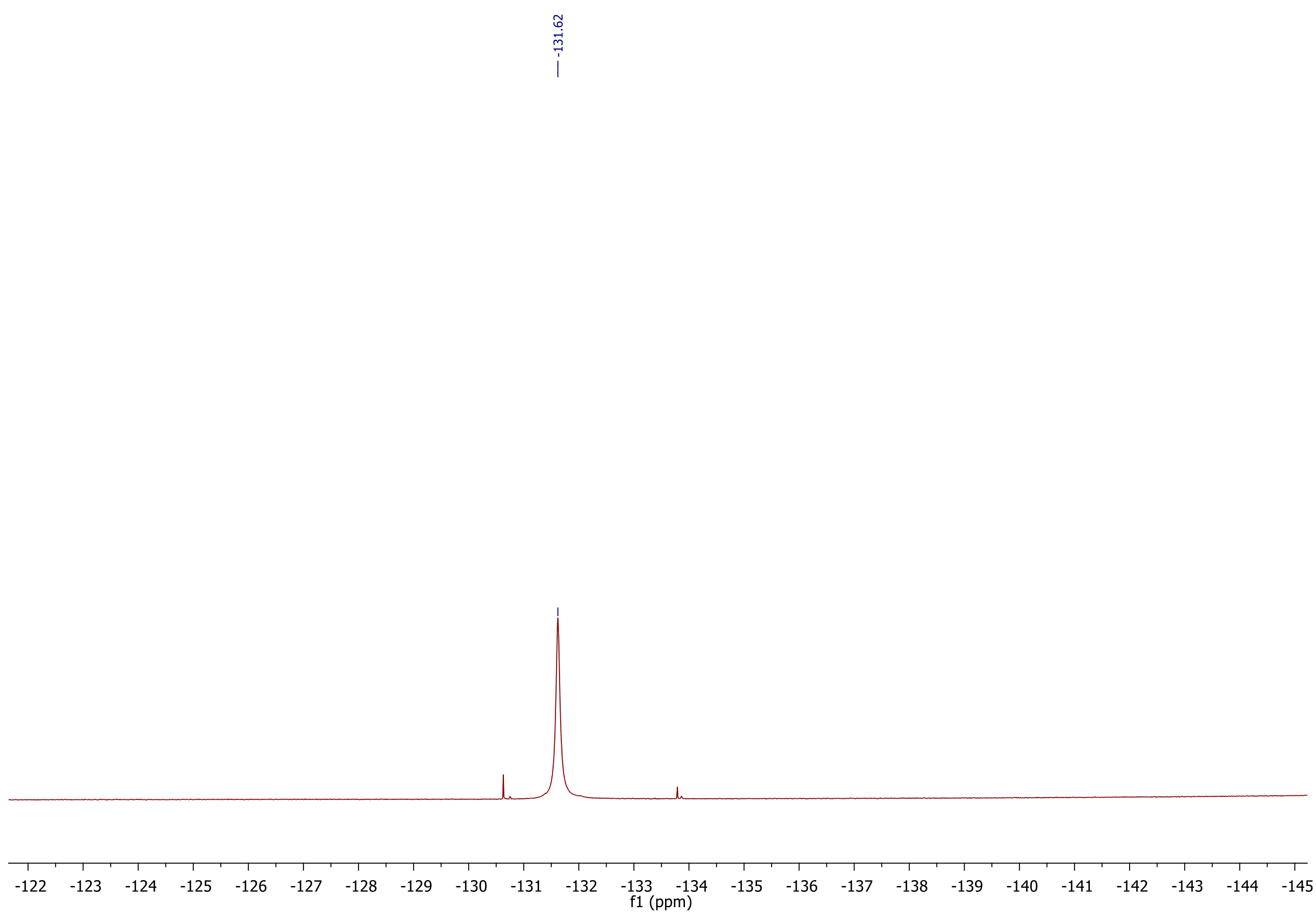

3-Chloro-*N*-(5-chlorothiazol-2-yl)-5-fluoro-2-hydroxybenzamide (10l)

^{1}H NMR (500 MHz, $(CD_3)_2SO$)

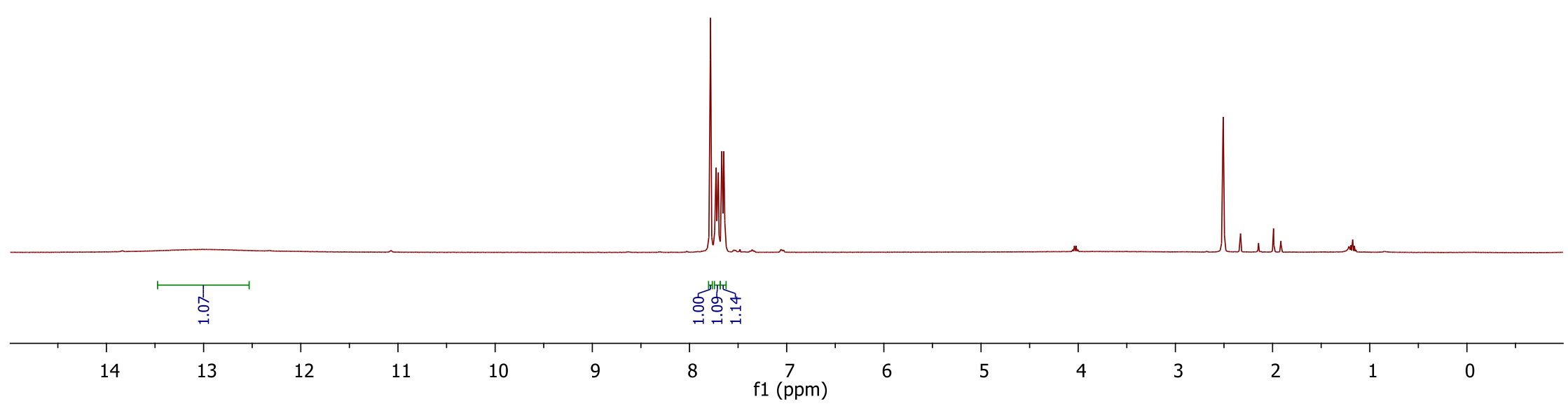

^{13}C NMR (126 MHz, $(CD_3)_2SO$)

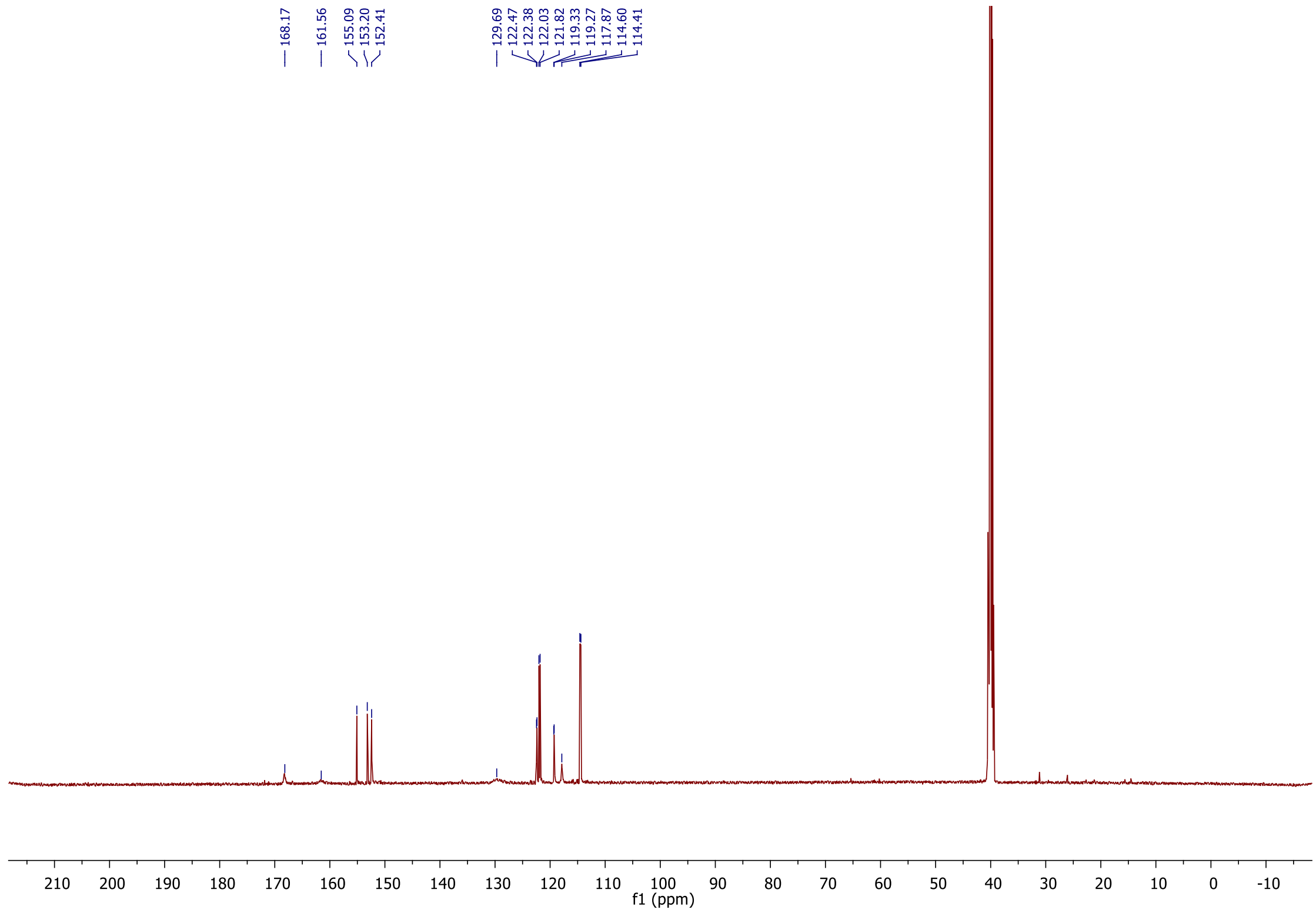

$^{19}F\{^1H\}$ NMR (376 MHz, $(CD_3)_2SO$)

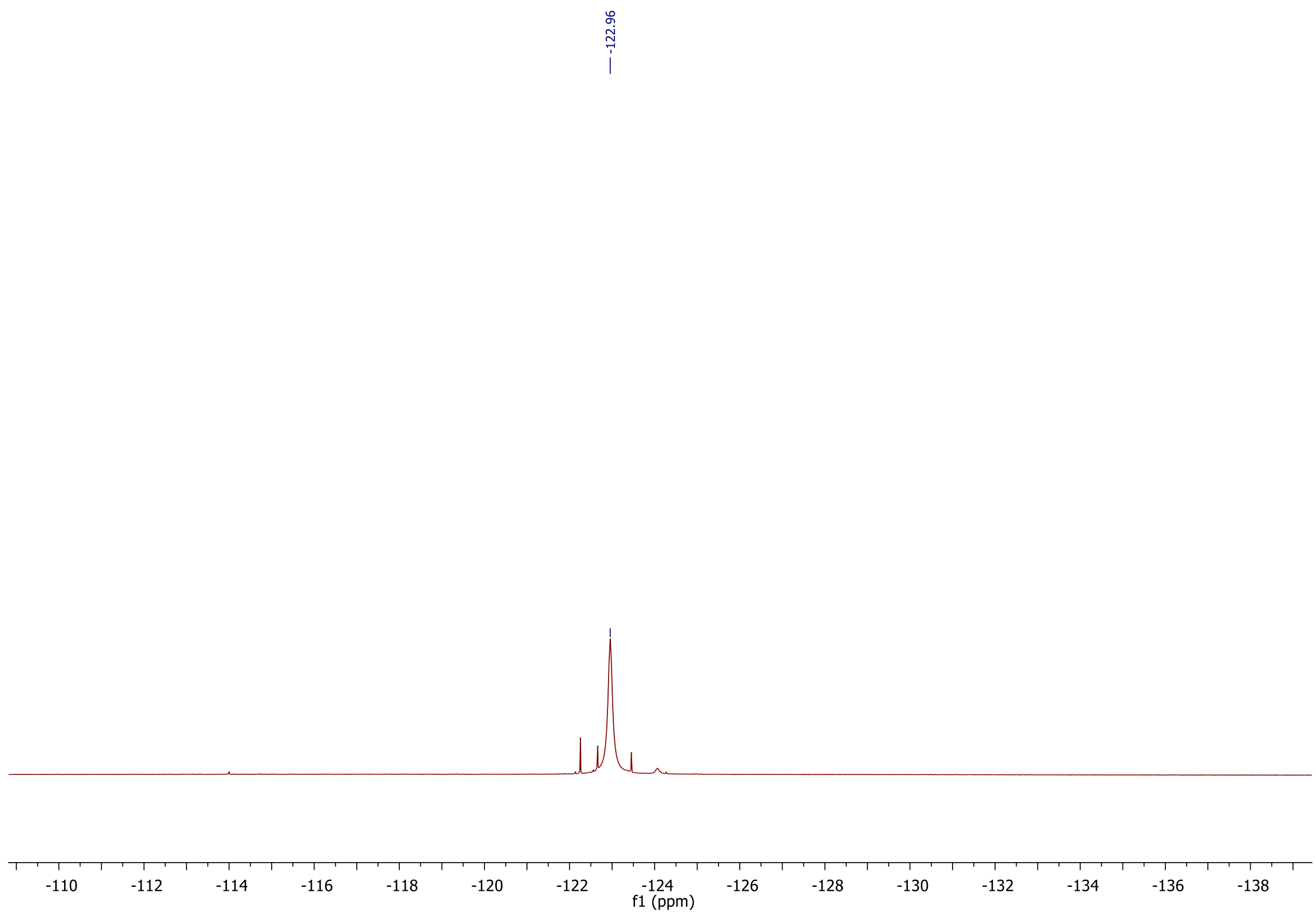

3,5-Dichloro-*N*-(5-chlorothiazol-2-yl)-2-hydroxybenzamide (10m)

^1H NMR (400 MHz, $(CD_3)_2SO$)

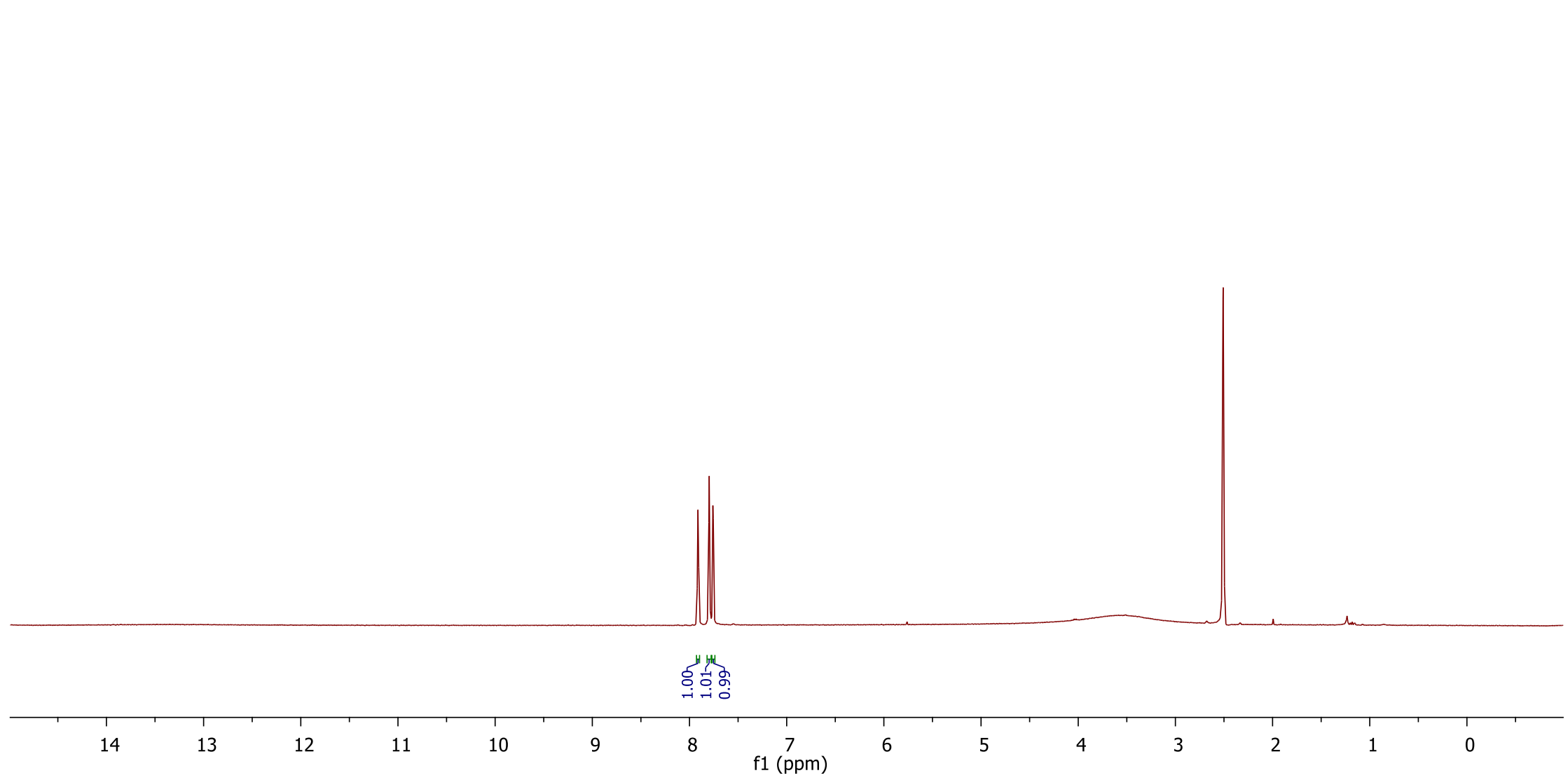

^{13}C NMR (126 MHz, $(CD_3)_2SO$)

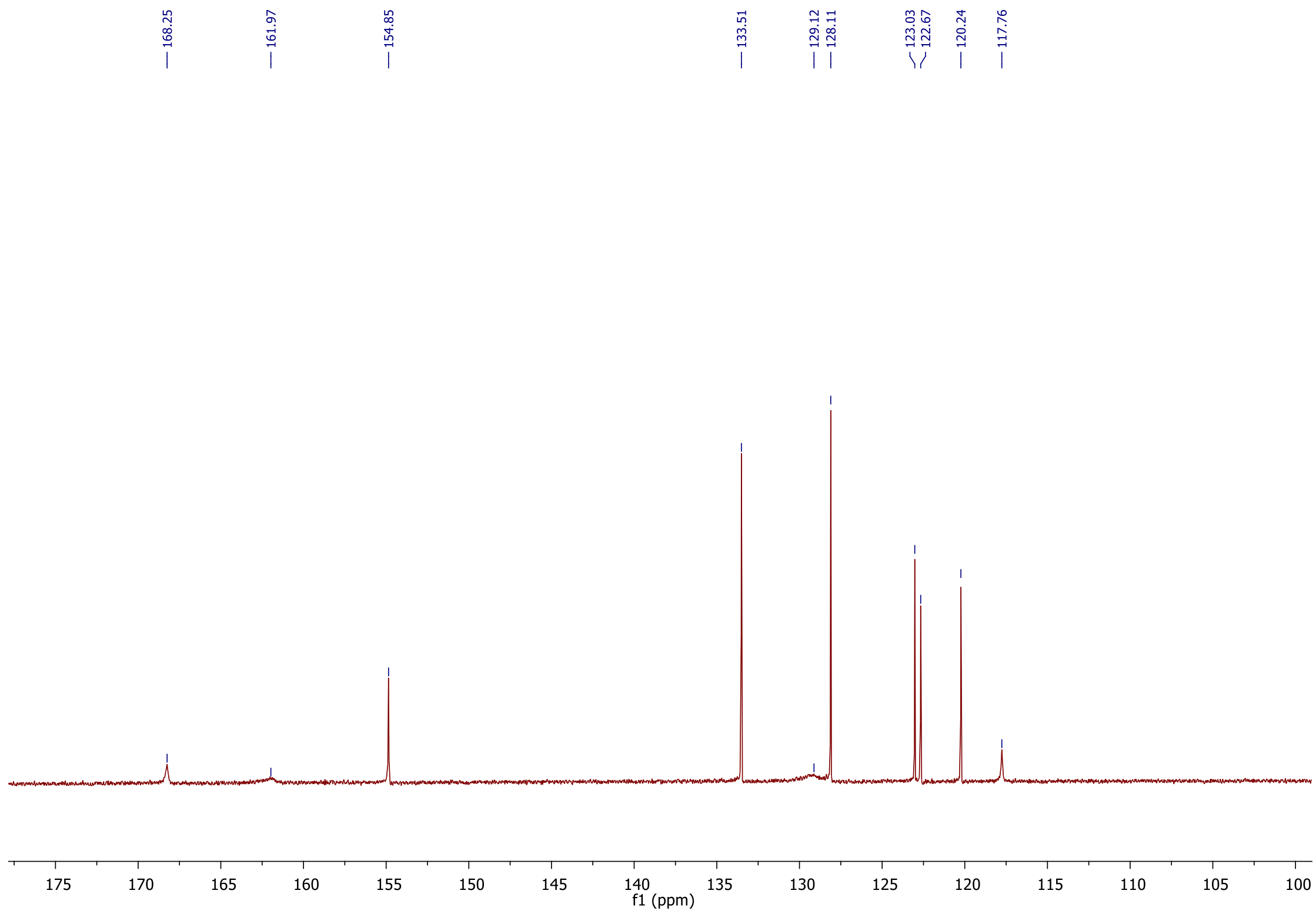

4-Fluoro-2-hydroxy-*N*-(5-nitrothiazol-2-yl)benzamide (11a)

^{1}H NMR (400 MHz, $(CD_3)_2SO$)

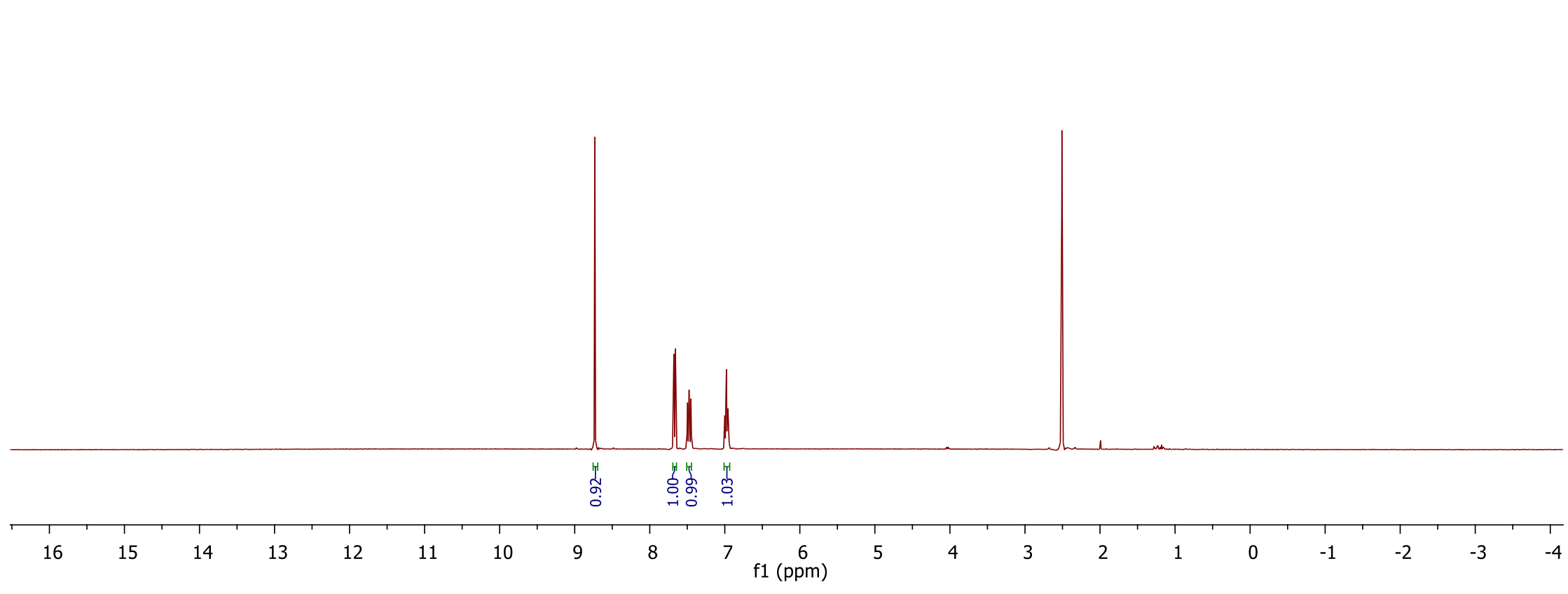

^{13}C NMR (126 MHz, $(CD_3)_2SO$)

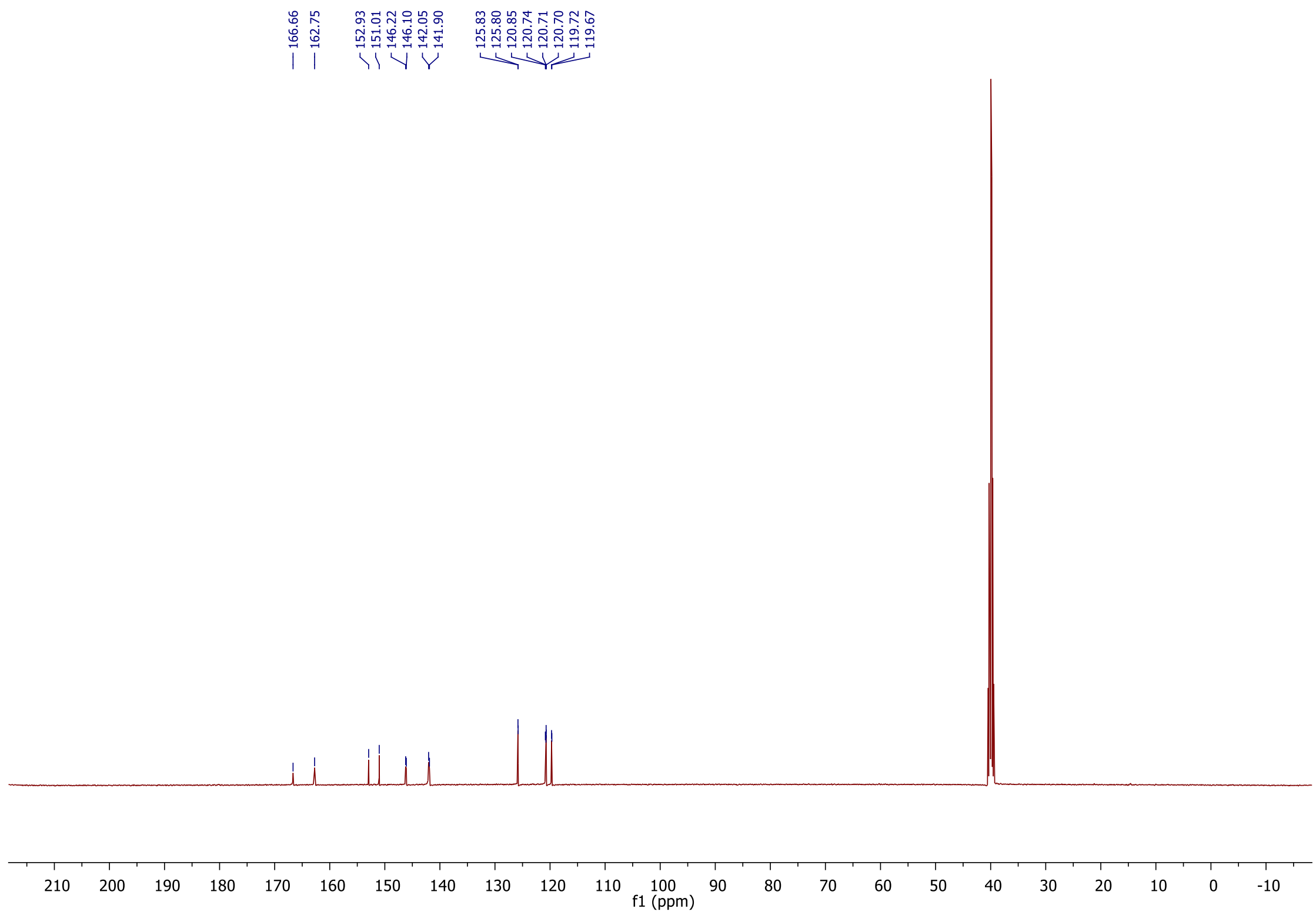

$^{19}F\{^1H\}$ NMR (376 MHz, $(CD_3)_2SO$)

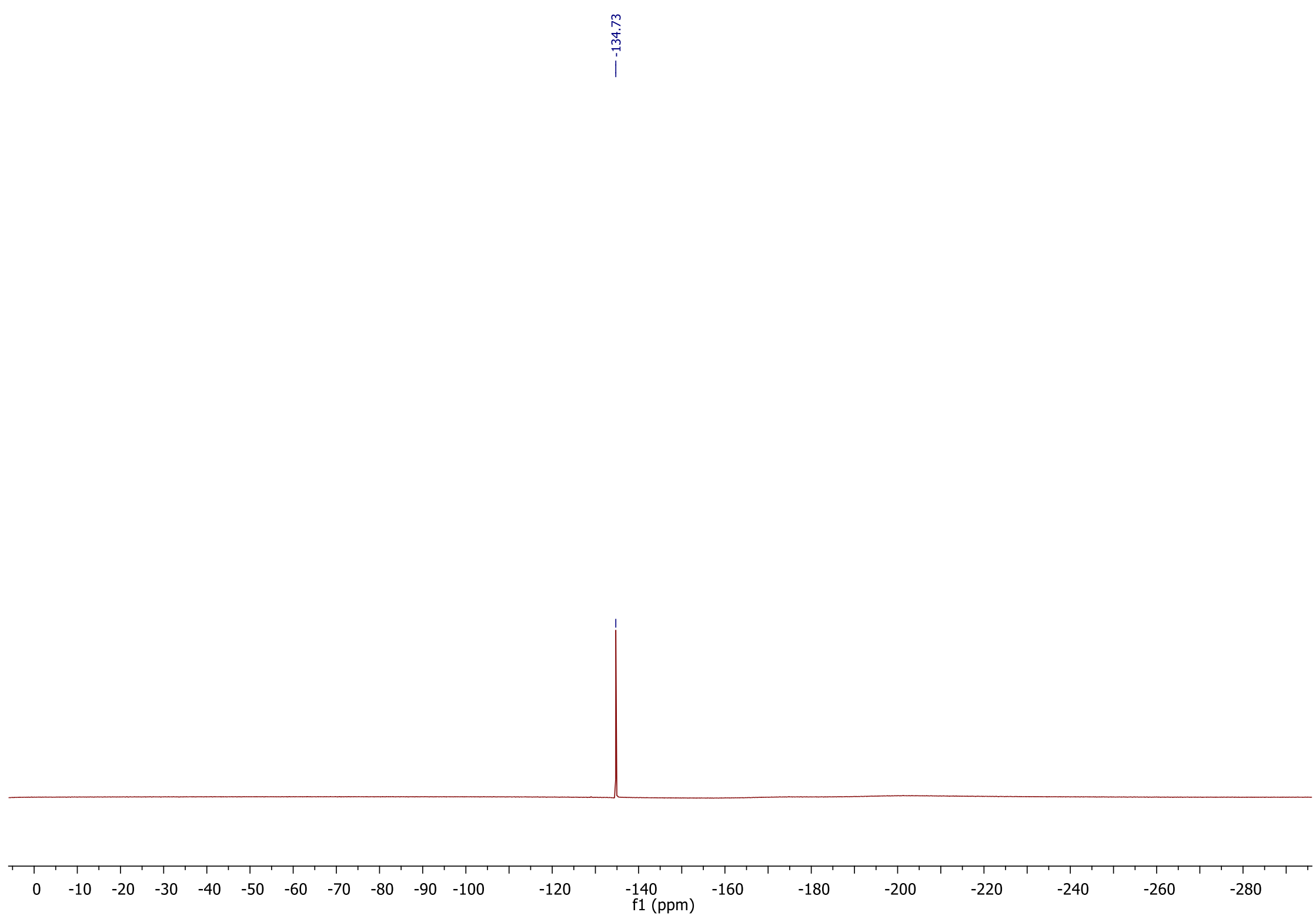

4-Fluoro-2-hydroxy-*N*-(5-nitrothiazol-2-yl)benzamide (11b)

^{1}H NMR (400 MHz, $(CD_3)_2SO$)

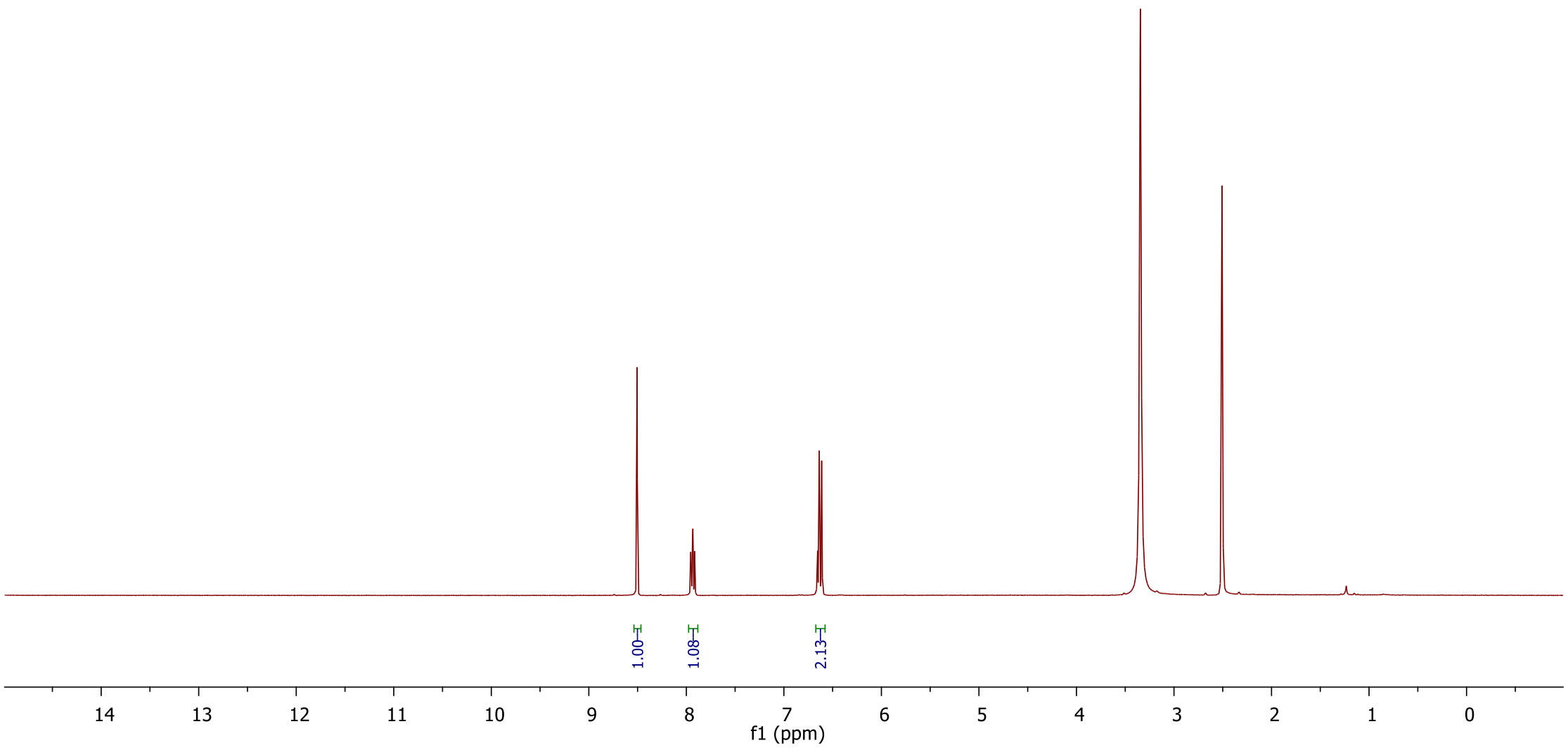

^{13}C NMR (126 MHz, $(CD_3)_2SO$)

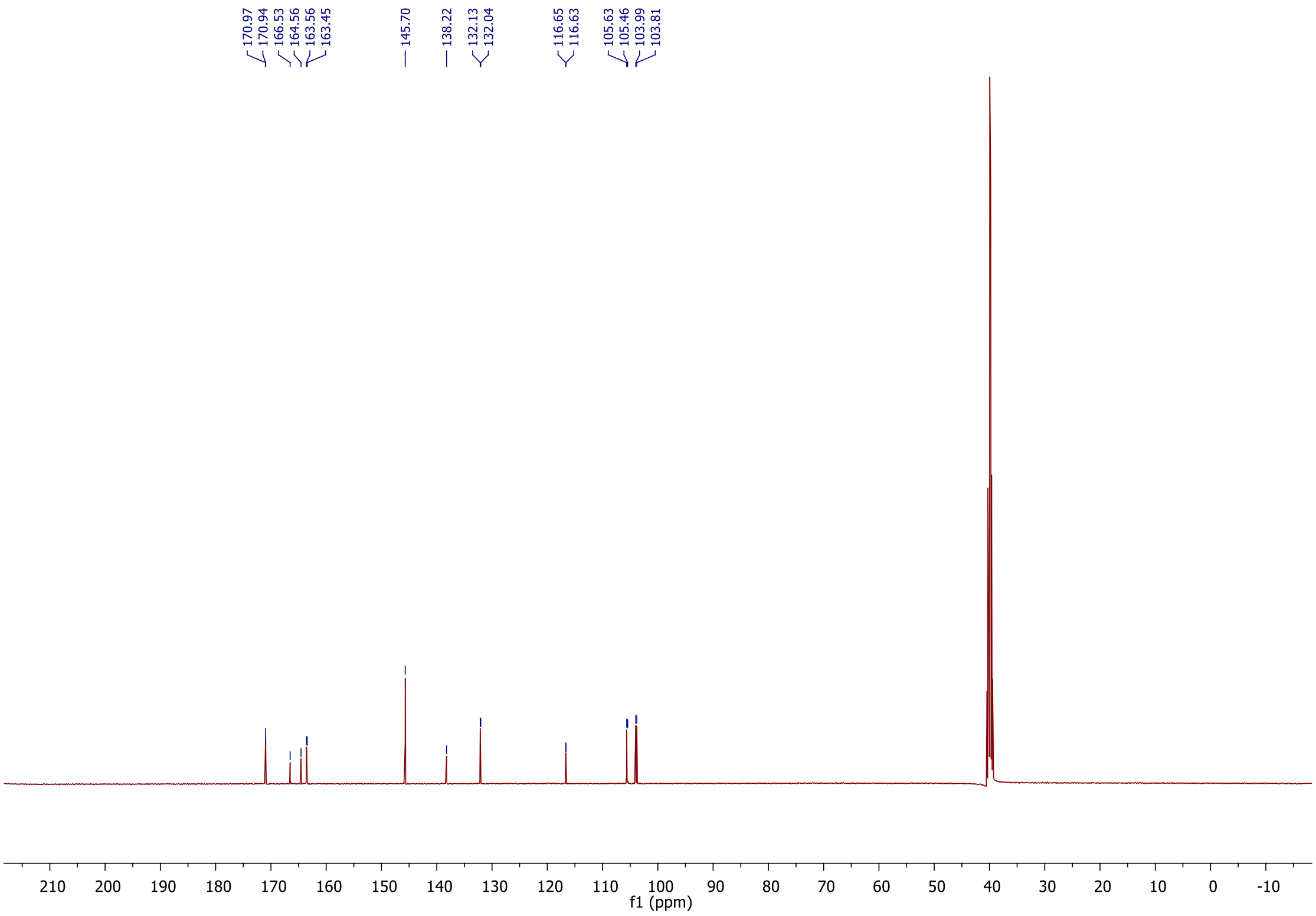

$^{19}F\{^{1}H\}$ NMR (376 MHz, $(CD_3)_2SO$)

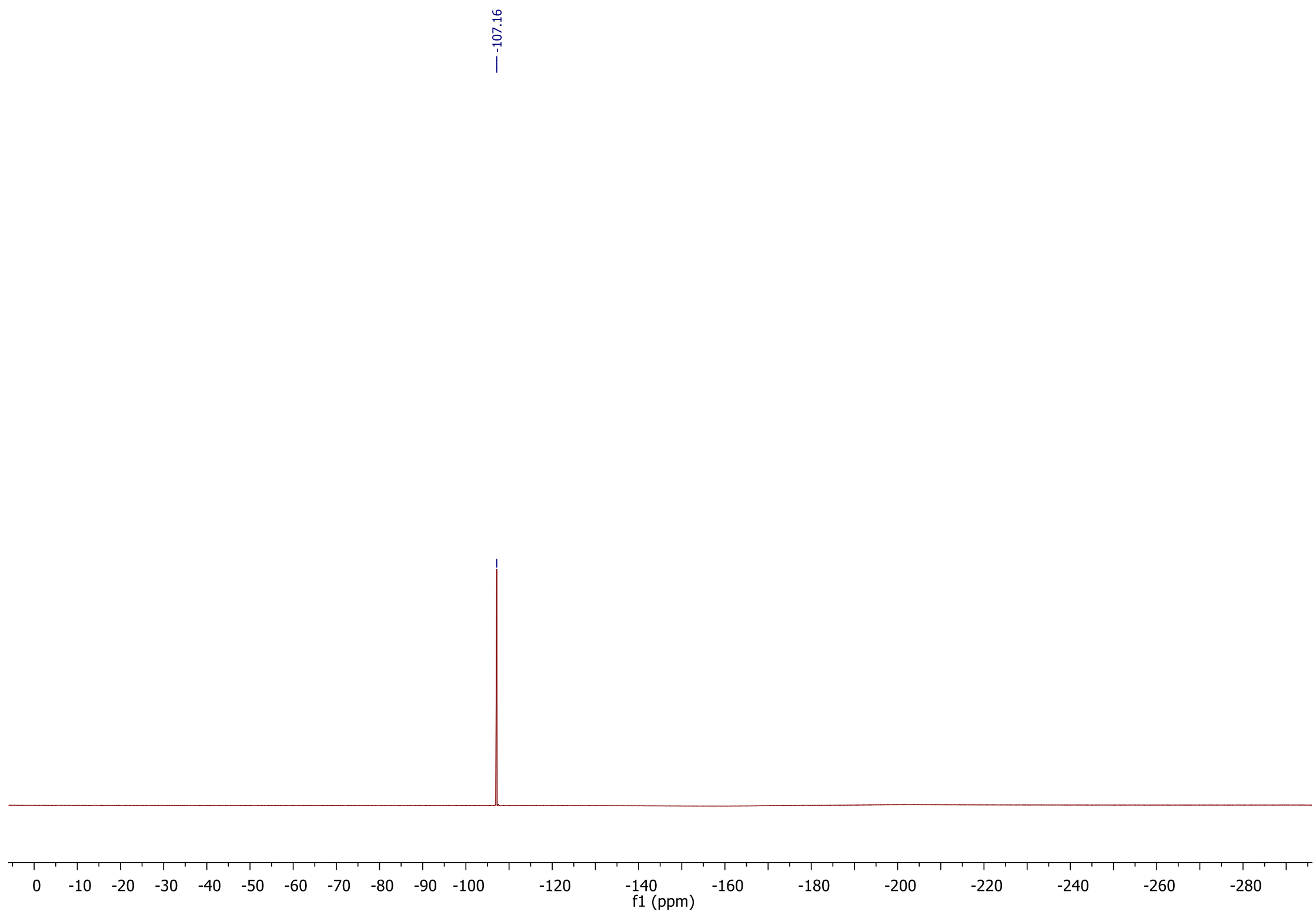

5-Fluoro-2-hydroxy-*N*-(5-nitrothiazol-2-yl)benzamide (11c)

^{1}H NMR (400 MHz, $(CD_3)_2SO$)

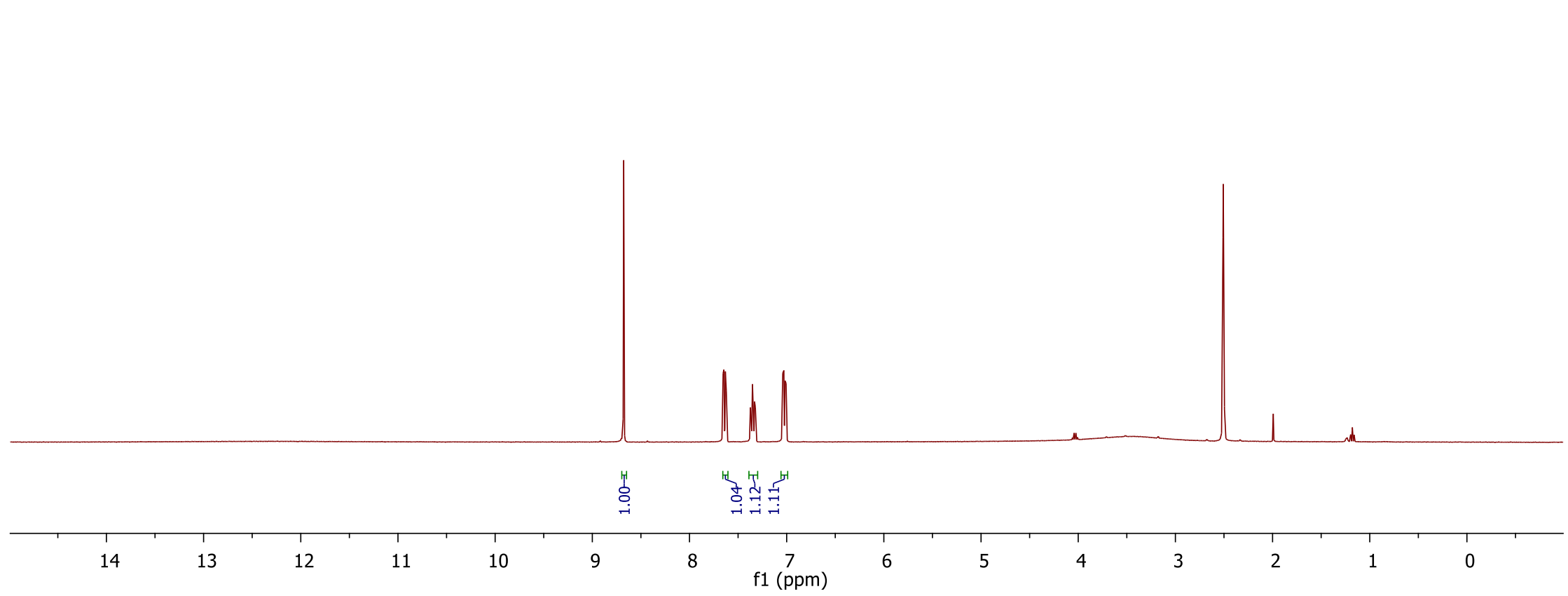

^{13}C NMR (126 MHz, $(CD_3)_2SO$)

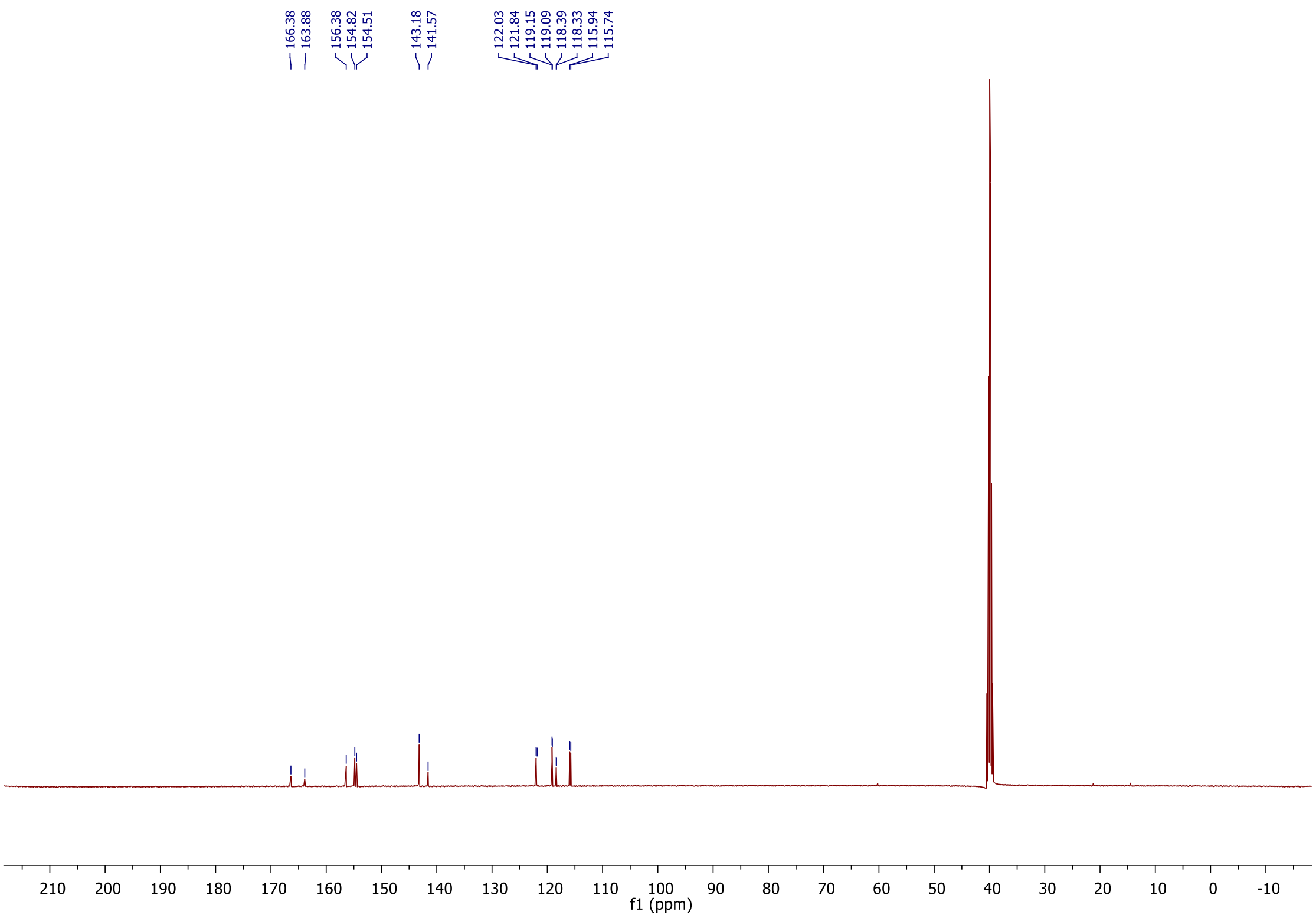

$^{19}F\{^1H\}$ NMR (376 MHz, $(CD_3)_2SO$)

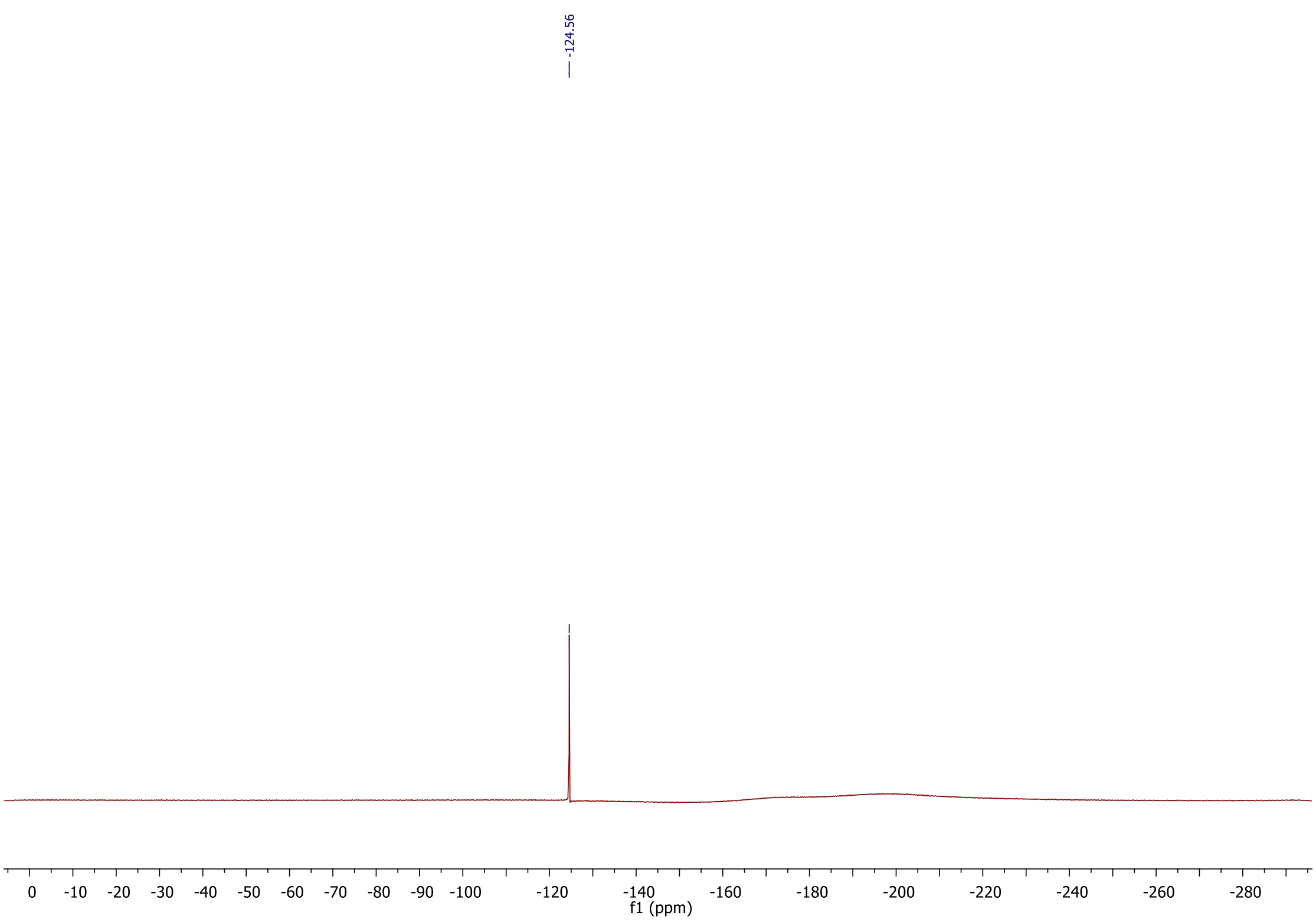

2-Fluoro-6-hydroxy-*N*-(5-nitrothiazol-2-yl)benzamide (11d)

^{1}H NMR (400 MHz, $(CD_3)_2SO$)

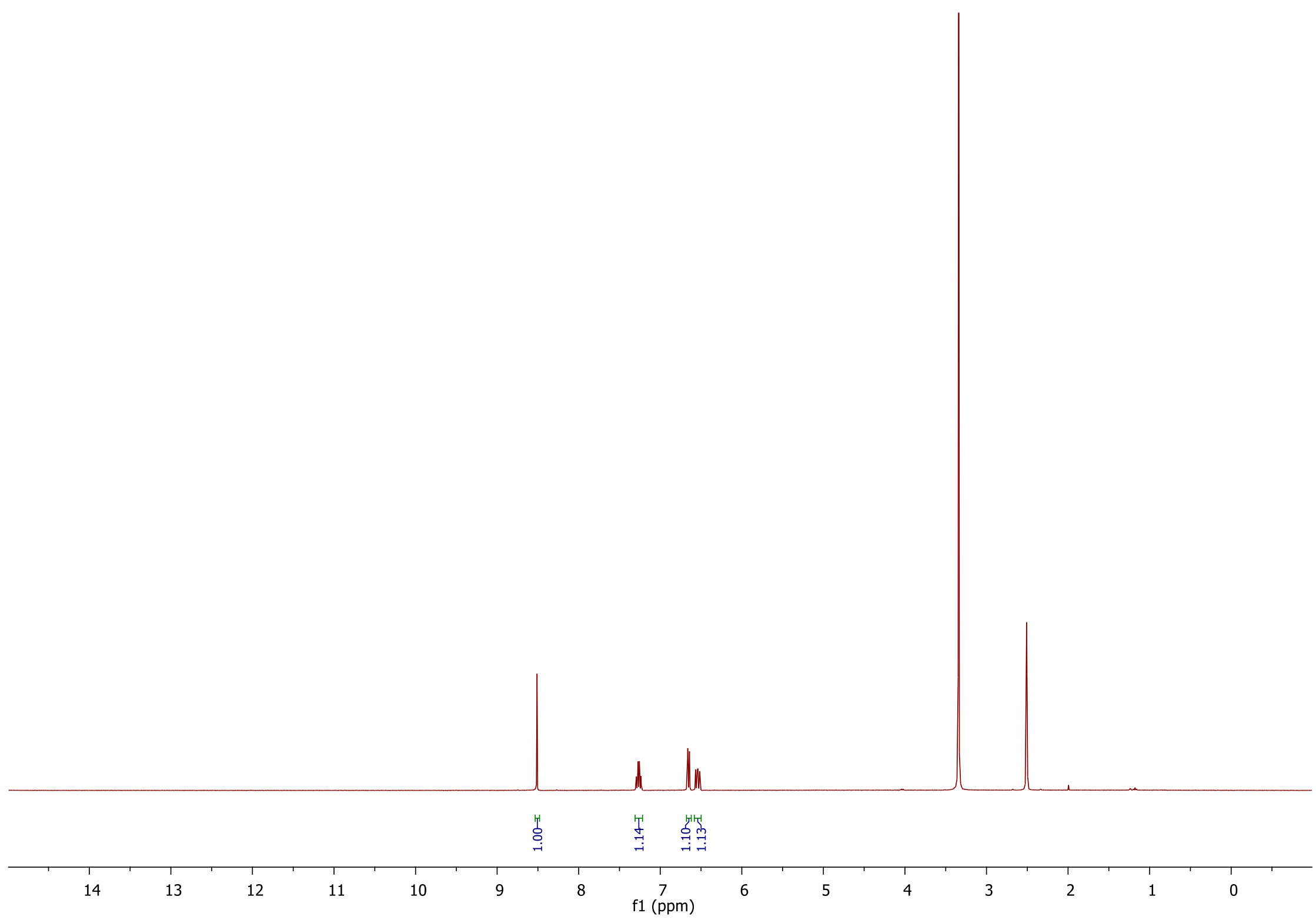

^{13}C NMR (126 MHz, $(CD_3)_2SO$)

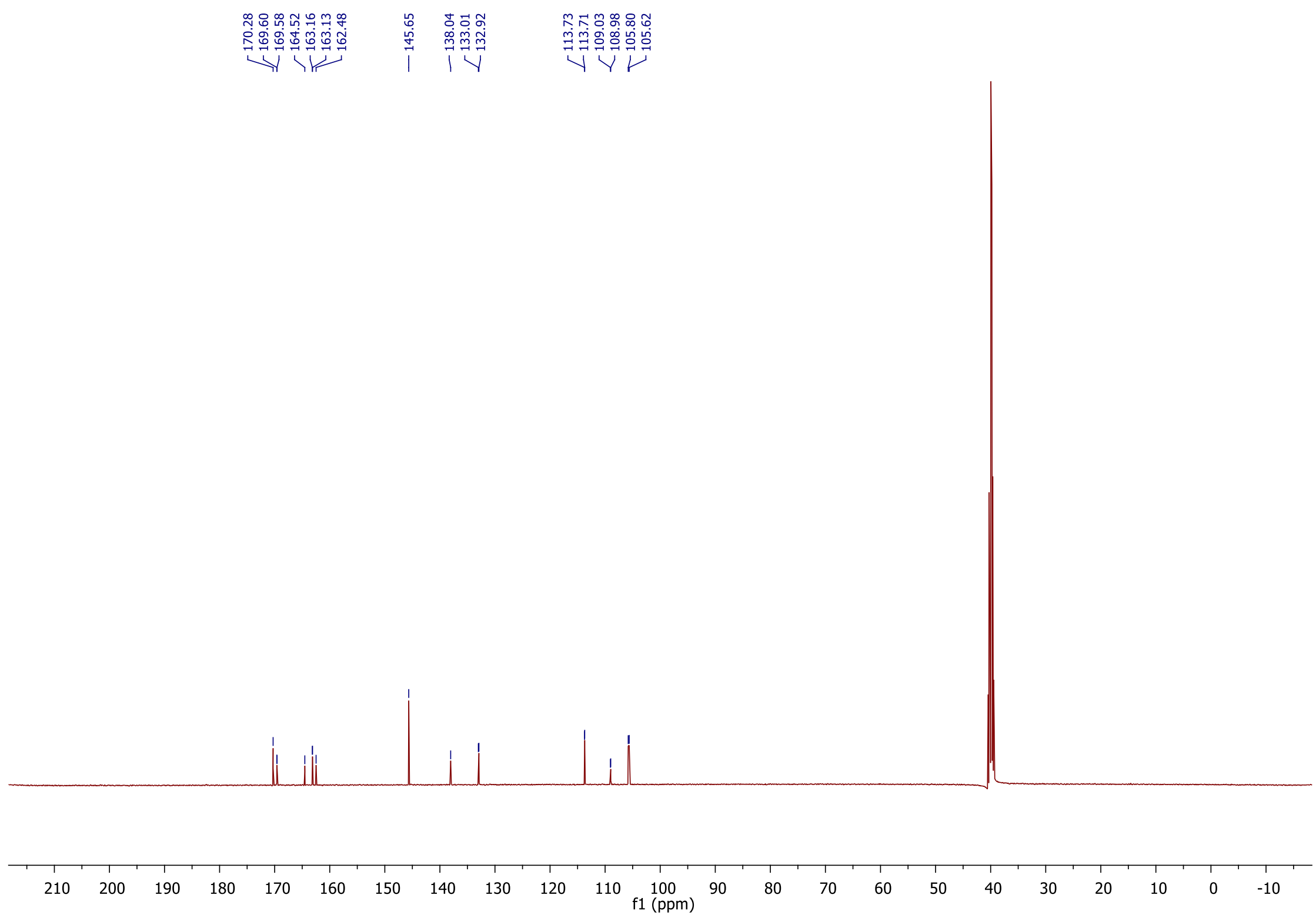

$^{19}F\{^1H\}$ NMR (376 MHz, $(CD_3)_2SO$)

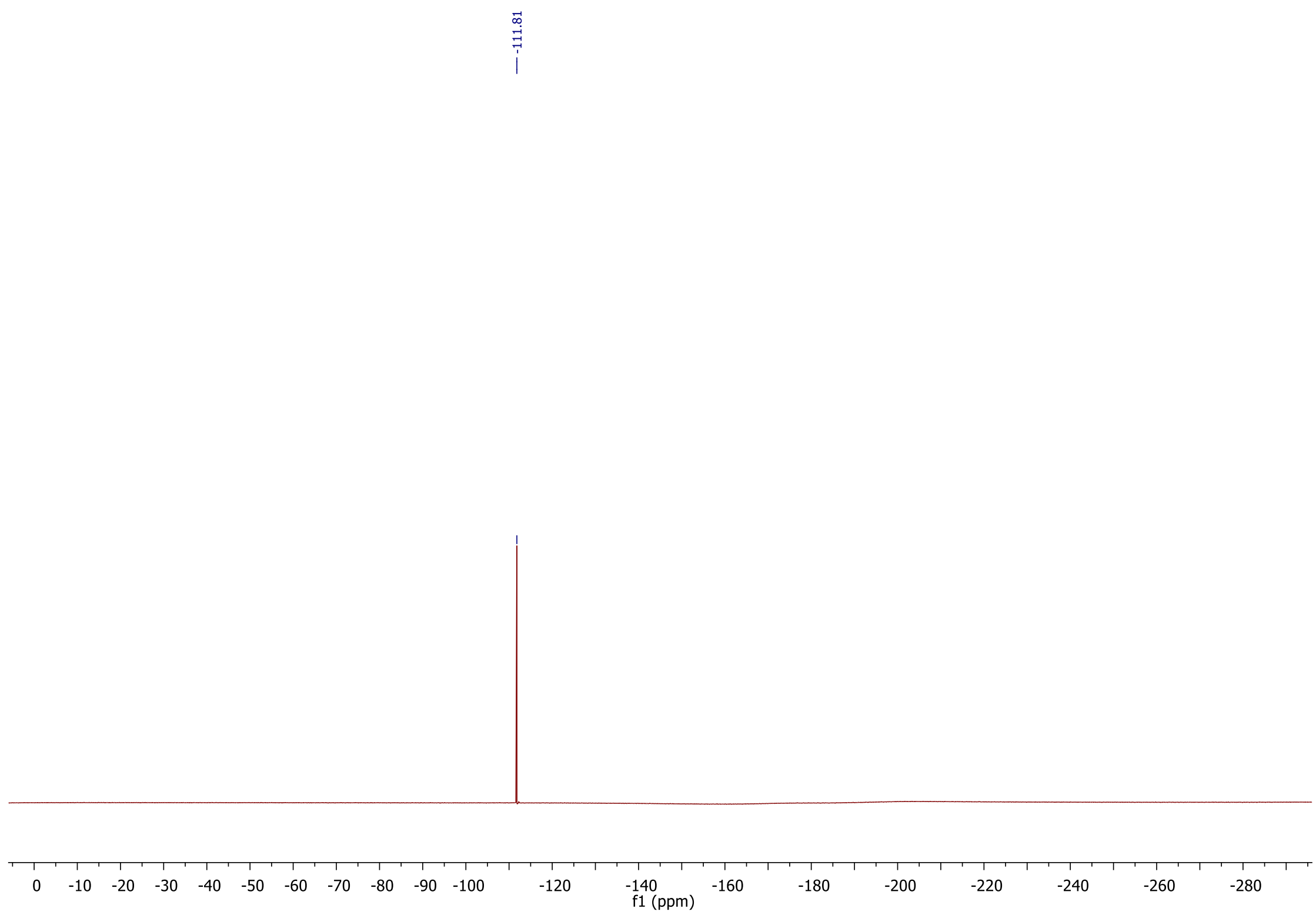

4-Chloro-2-hydroxy-*N*-(5-nitrothiazol-2-yl)benzamide (11e)

^1H NMR (500 MHz, $(CD_3)_2SO$)

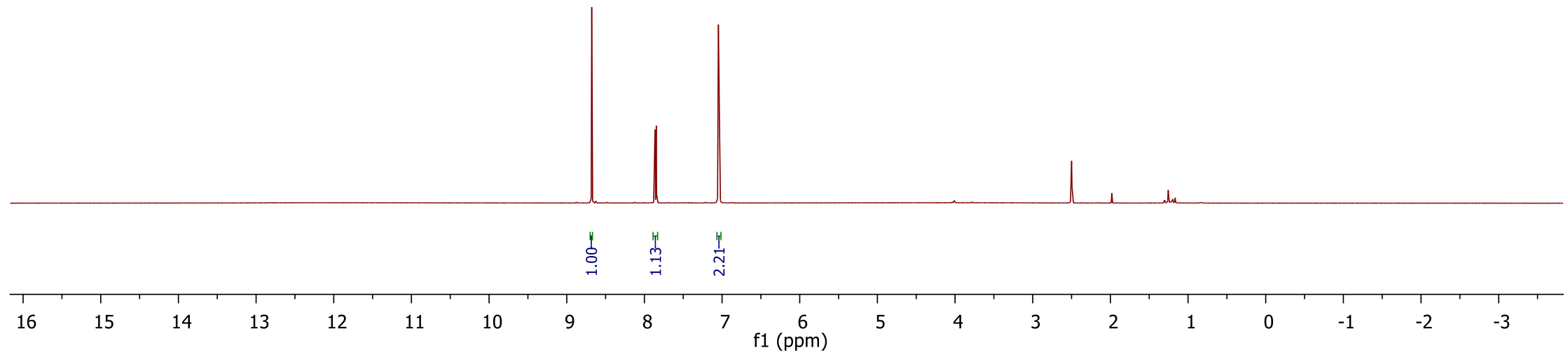

^{13}C NMR (126 MHz, $(CD_3)_2SO$)

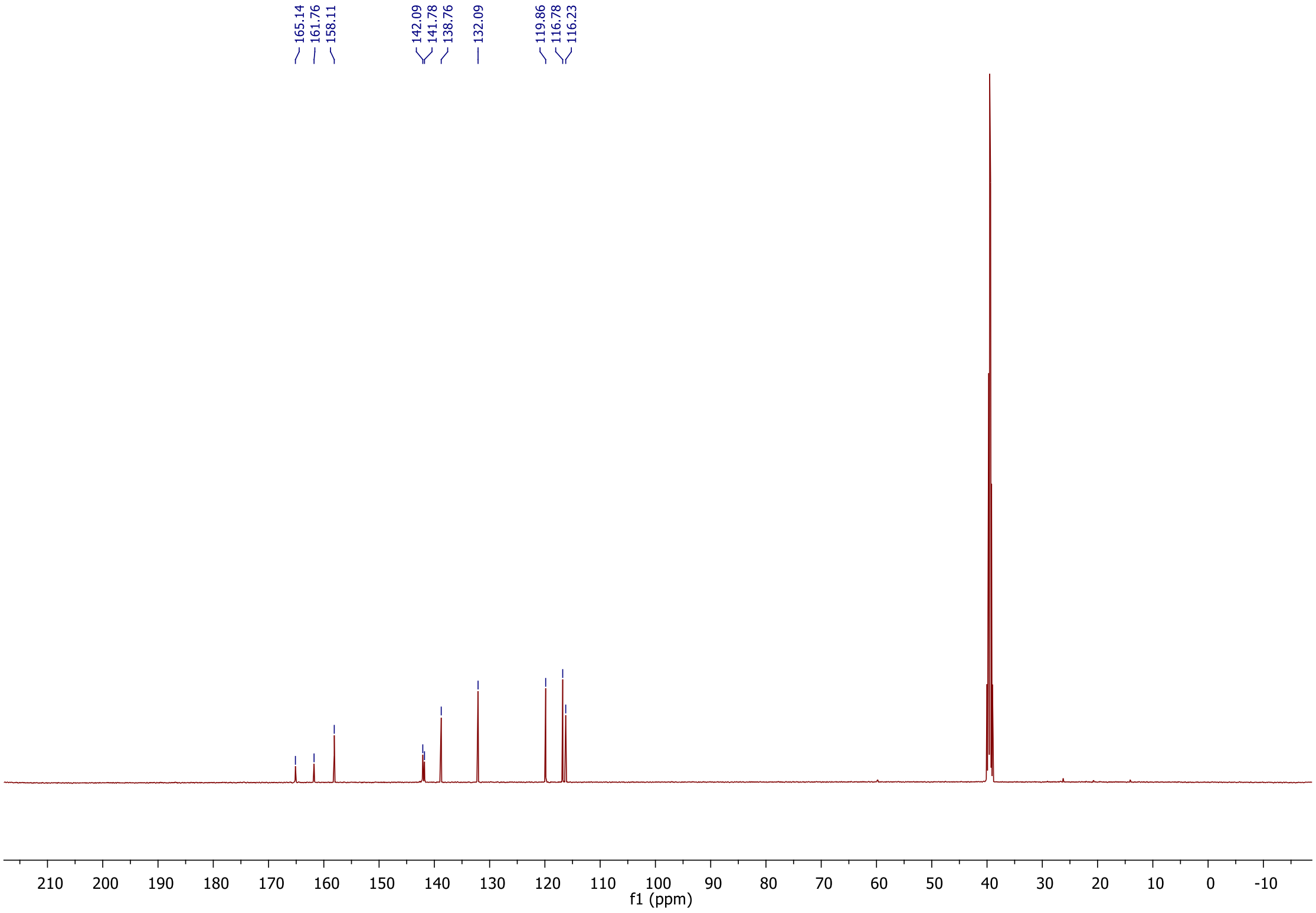

5-Chloro-2-hydroxy-*N*-(5-nitrothiazol-2-yl)benzamide (11f)

^{1}H NMR (500 MHz, $(CD_3)_2SO$)

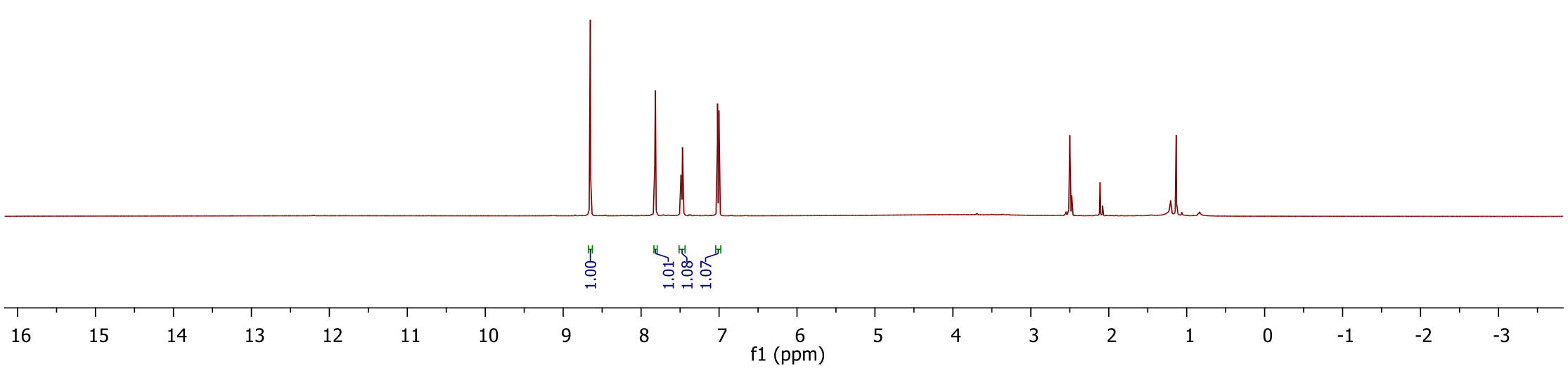

^{13}C NMR (126 MHz, $(CD_3)_2SO$)

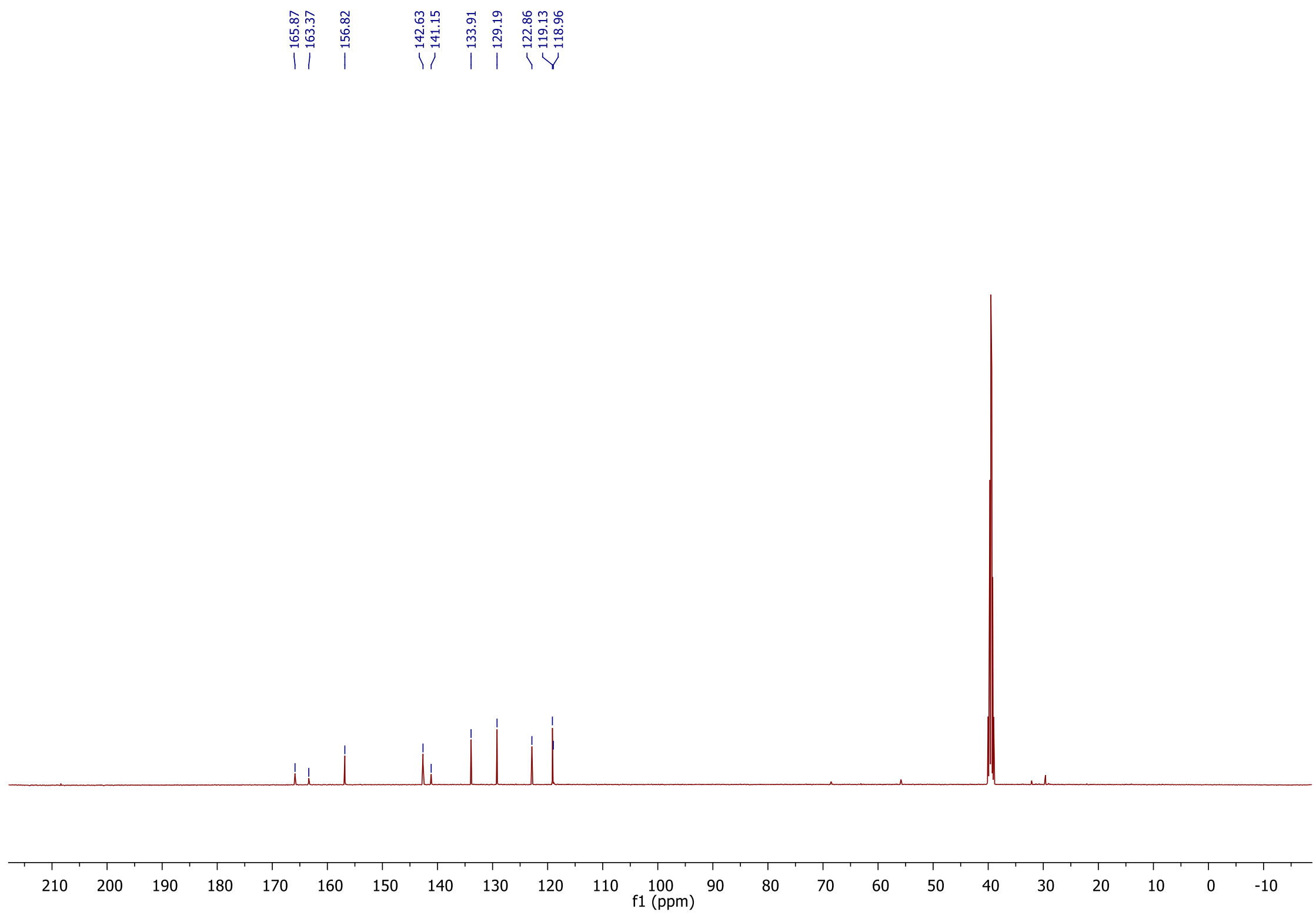

3,5-Difluoro-2-hydroxy-*N*-(5-nitrothiazol-2-yl)benzamide (11g)

^{1}H NMR (500 MHz, $(CD_3)_2SO$)

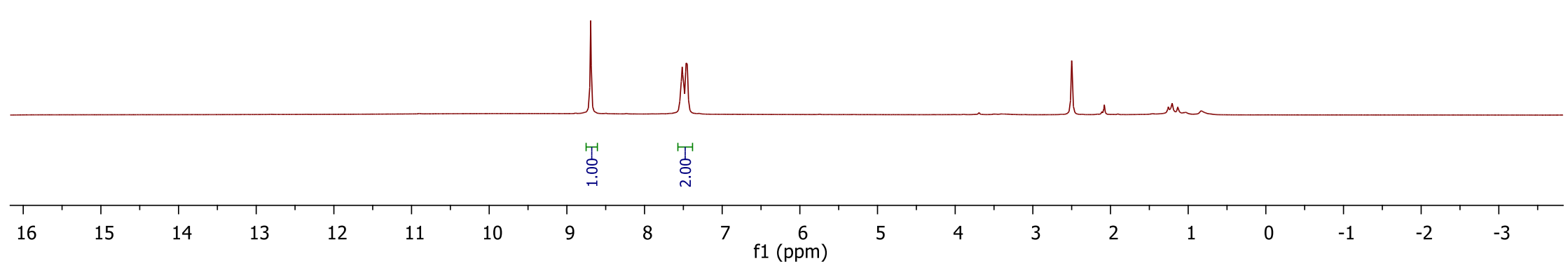

^{13}C NMR (126 MHz, $(CD_3)_2SO$)

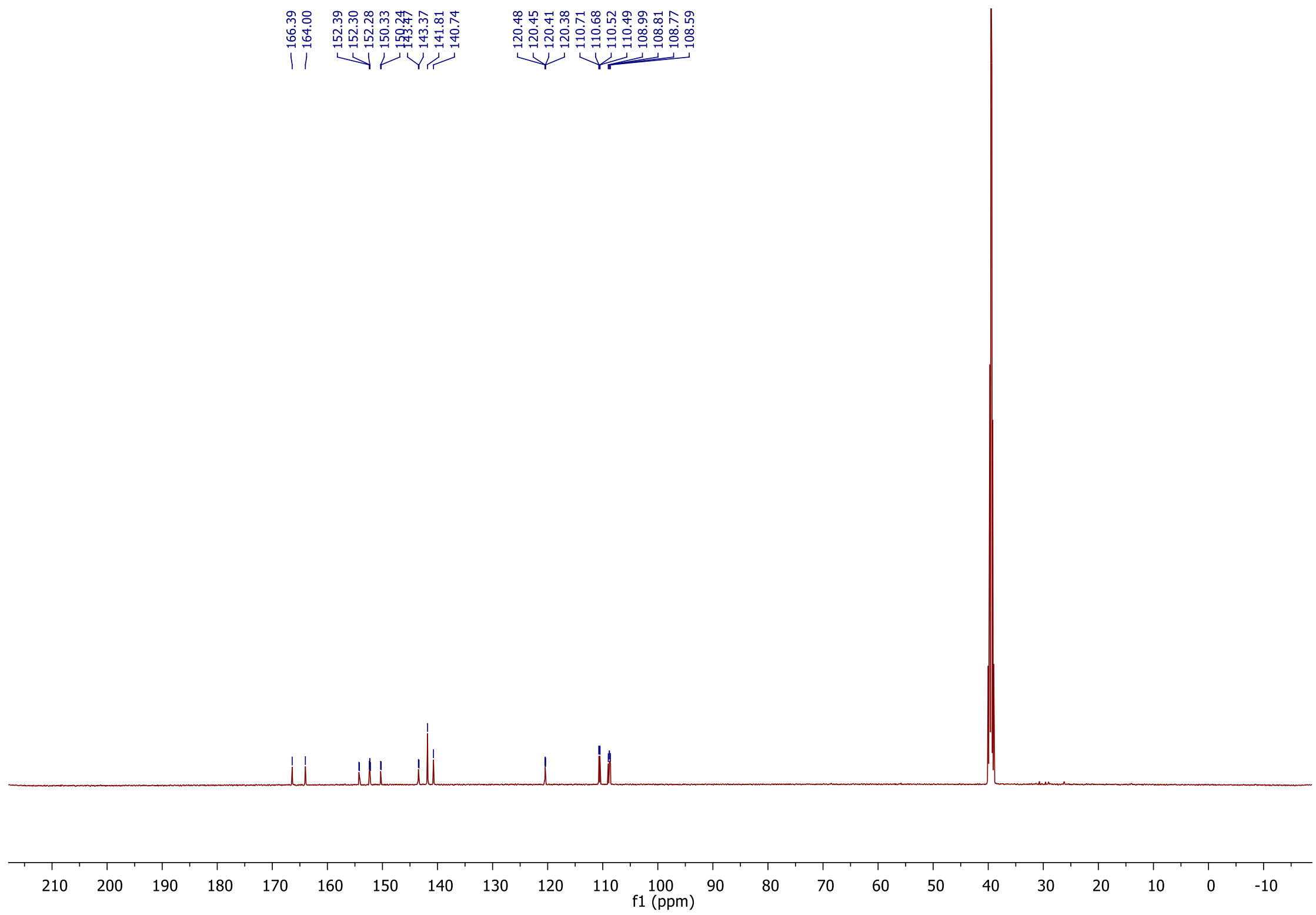

$^{19}F\{^1H\}$ NMR (376 MHz, $(CD_3)_2SO$)

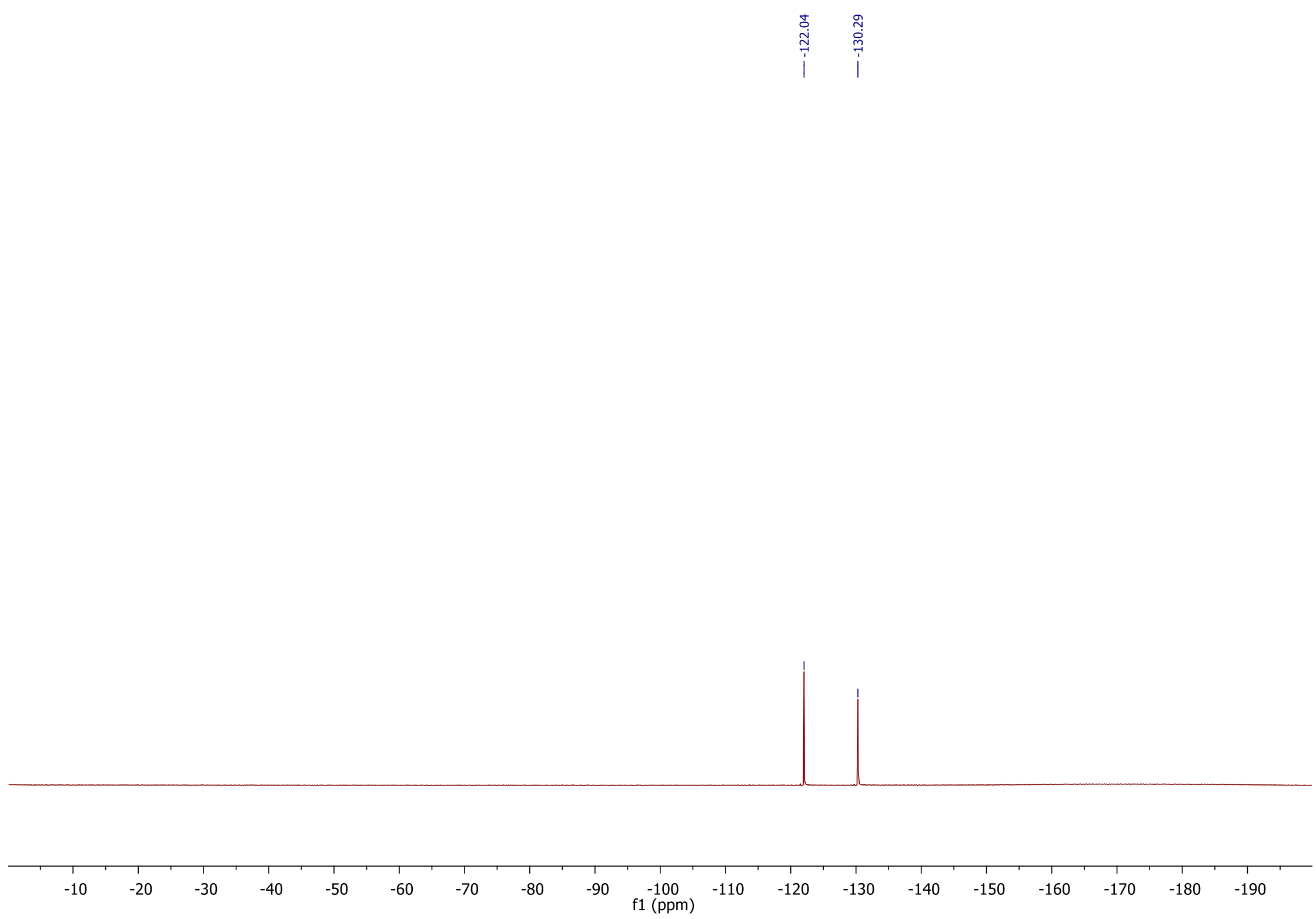

3,5-Dichloro-2-hydroxy-*N*-(5-nitrothiazol-2-yl)benzamide (11h)

^{1}H NMR (400 MHz, $(CD_3)_2SO$)

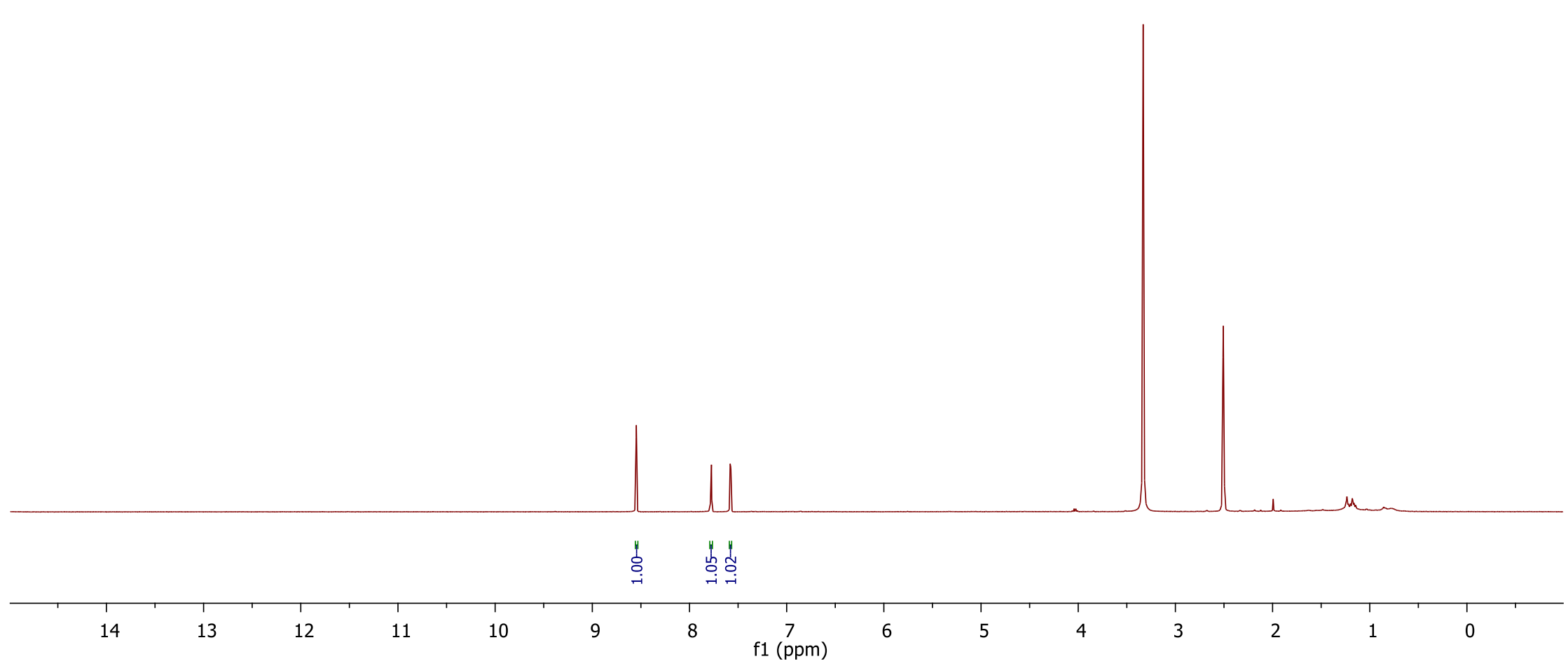

^{13}C NMR (126 MHz, $(CD_3)_2SO$)

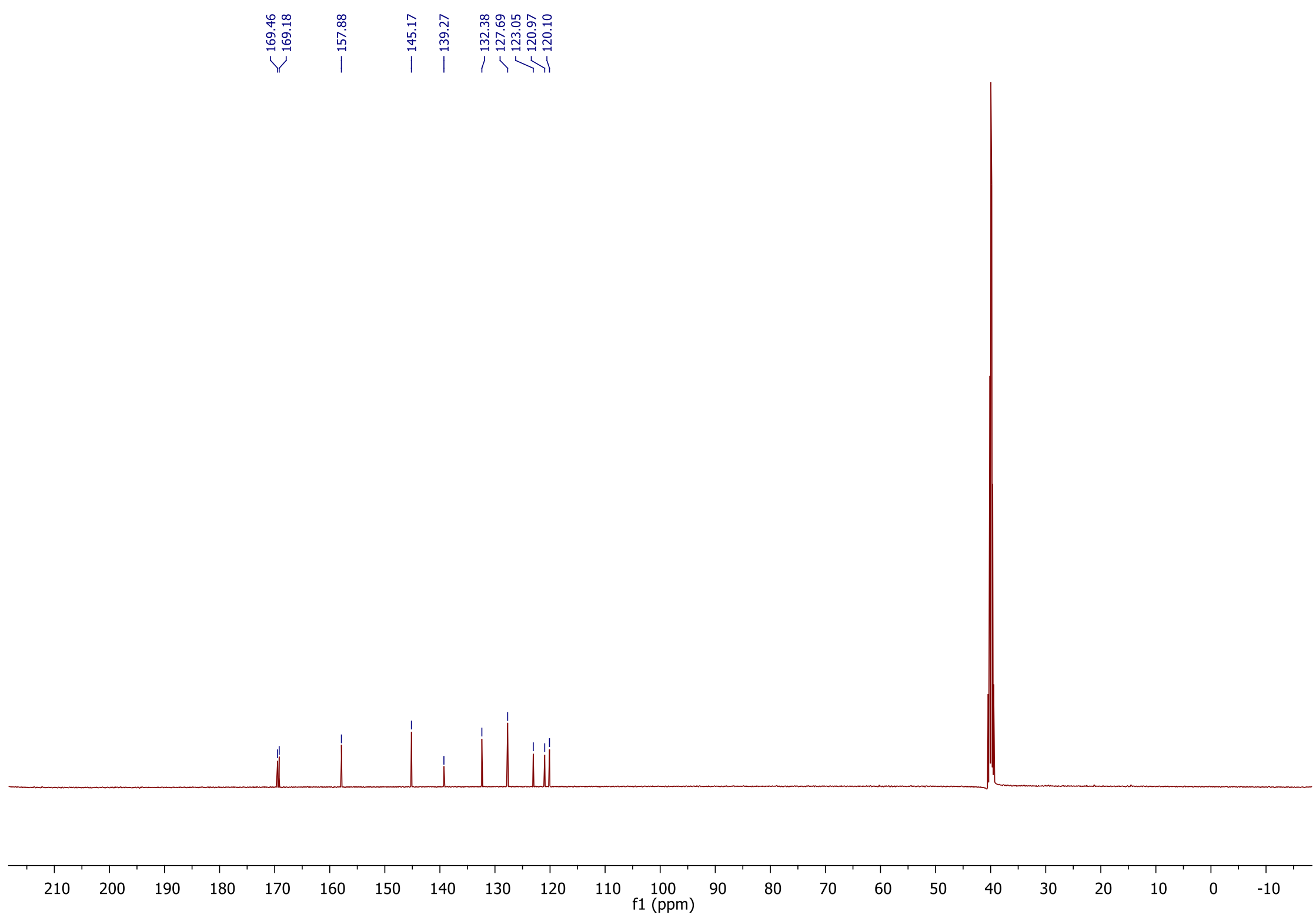

5-Chloro-3-fluoro-2-hydroxy-*N*-(5-nitrothiazol-2-yl)benzamide (11i)

^{1}H NMR (500 MHz, $(CD_3)_2SO$)

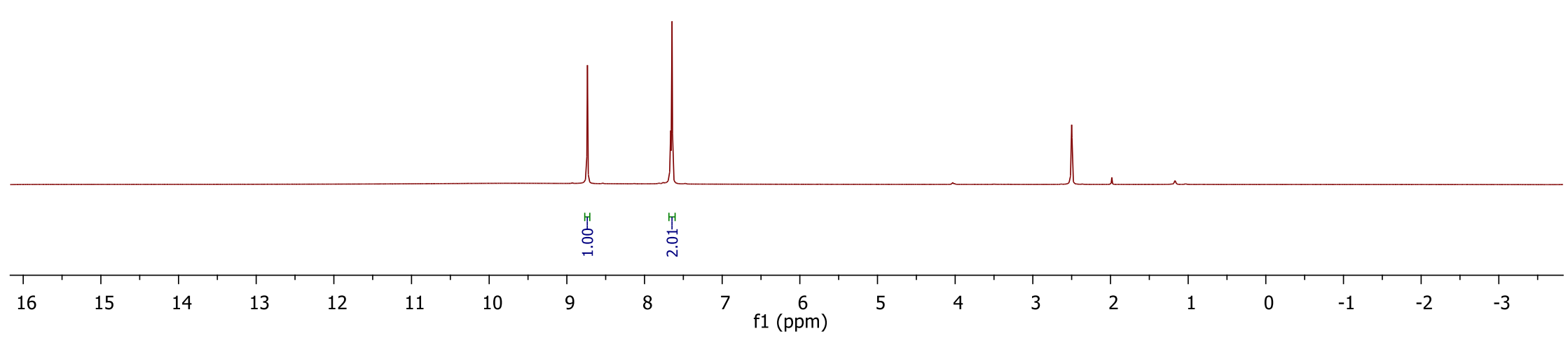

^{13}C NMR (126 MHz, $(CD_3)_2SO$)

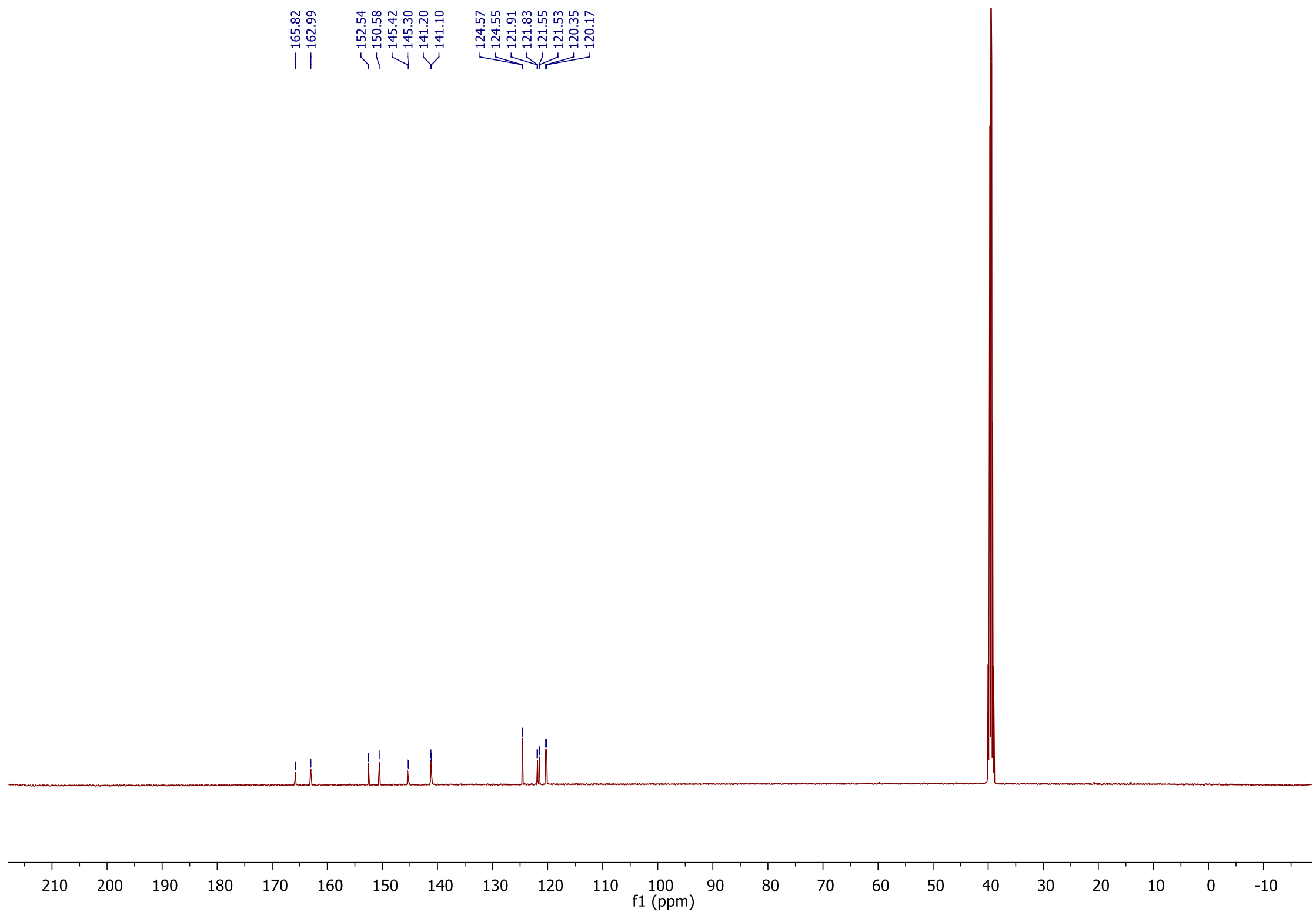

$^{19}F\{^{1}H\}$ NMR (376 MHz, $(CD_3)_2SO$)

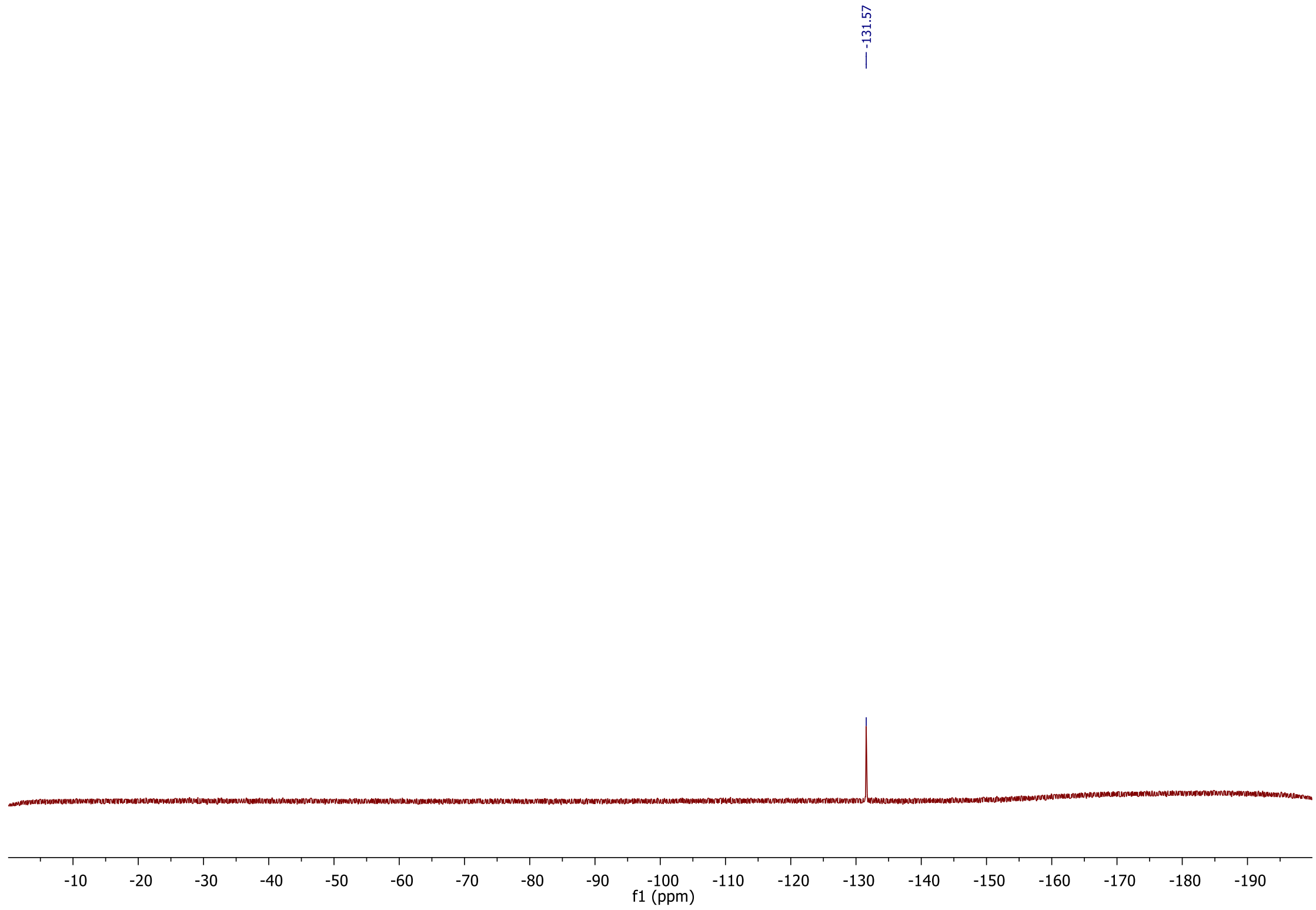

3-Chloro-2-hydroxy-*N*-(5-nitrothiazol-2-yl)benzamide (11j)

^{1}H NMR (500 MHz, $(CD_3)_2SO$)

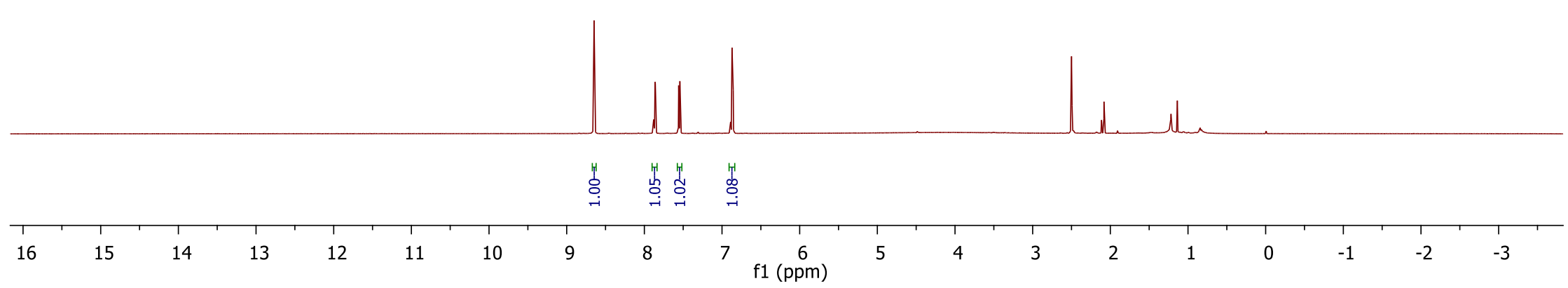

^{13}C NMR (126 MHz, $(CD_3)_2SO$)

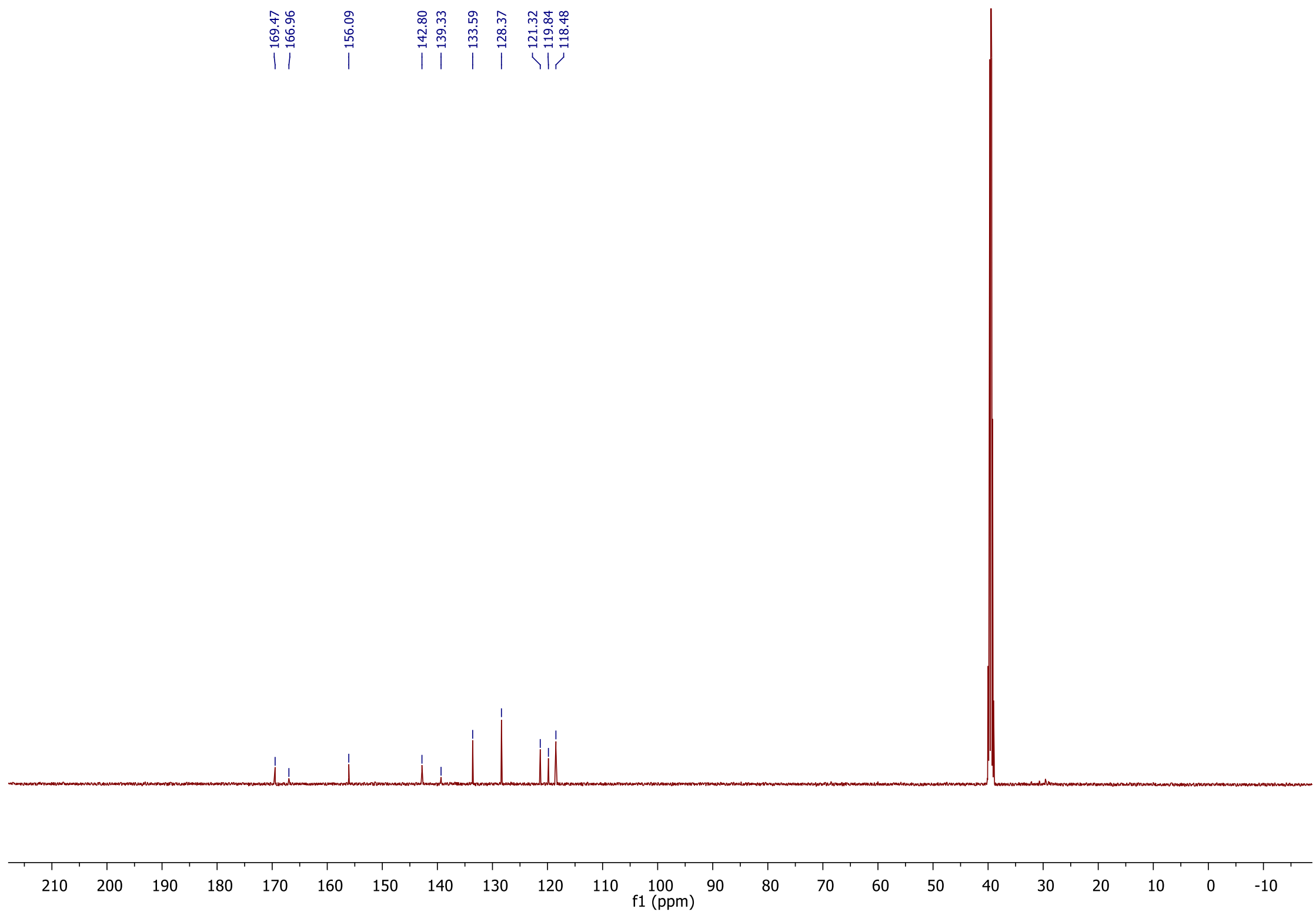

3-Chloro-5-fluoro-2-hydroxy-*N*-(5-nitrothiazol-2-yl)benzamide (11k)

^{1}H NMR (500 MHz, $(CD_3)_2SO$)

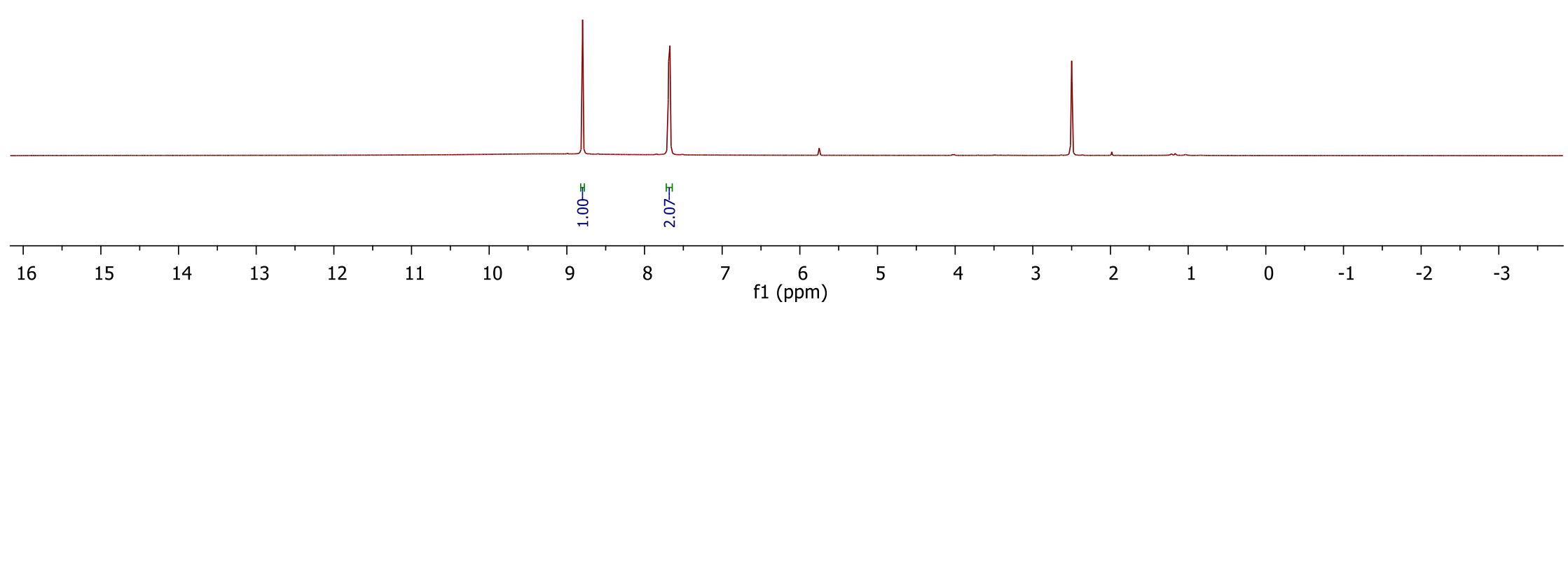

^{13}C NMR (126 MHz, $(CD_3)_2SO$)

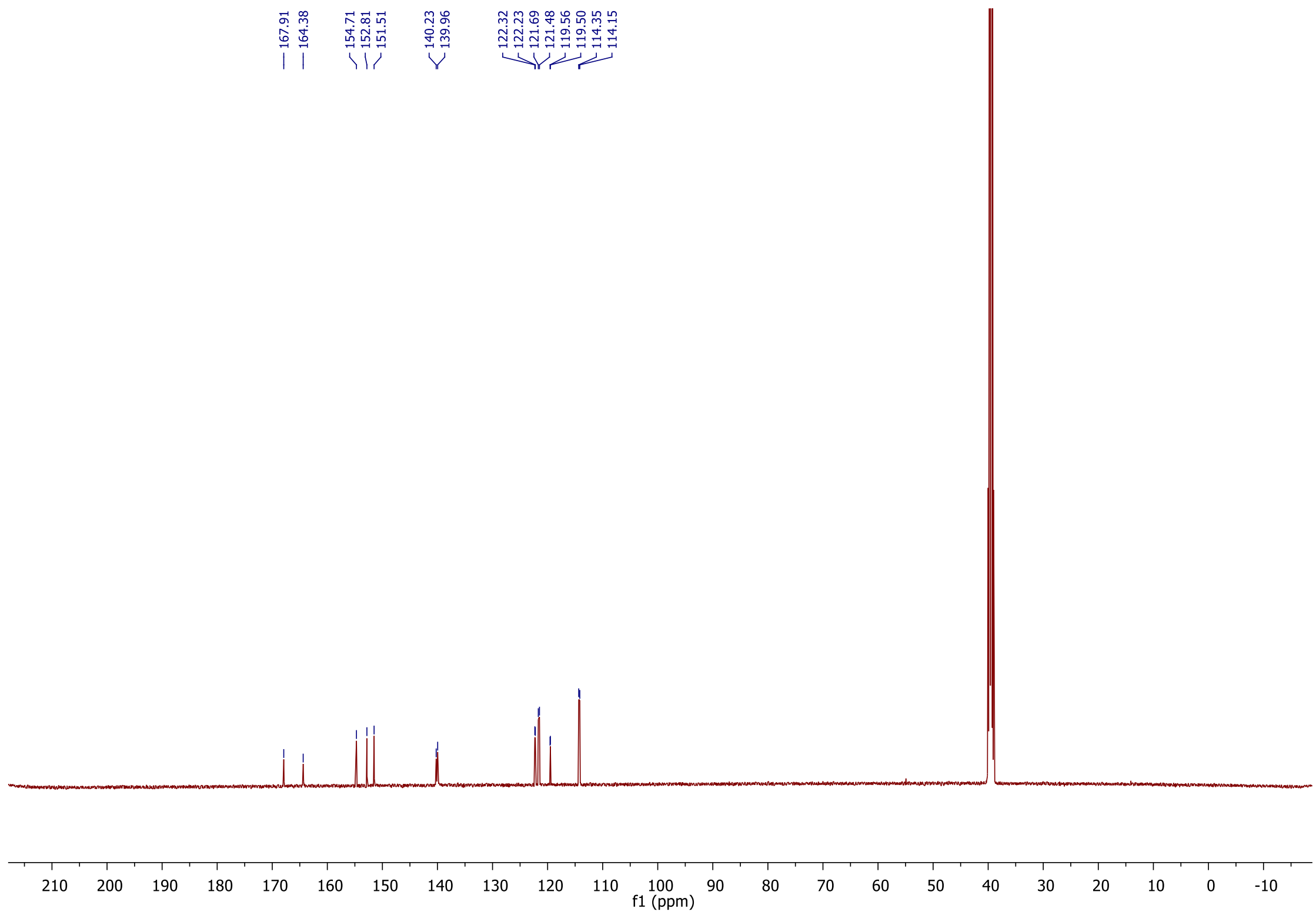

$^{19}F\{^1H\}$ NMR (376 MHz, $(CD_3)_2SO$)

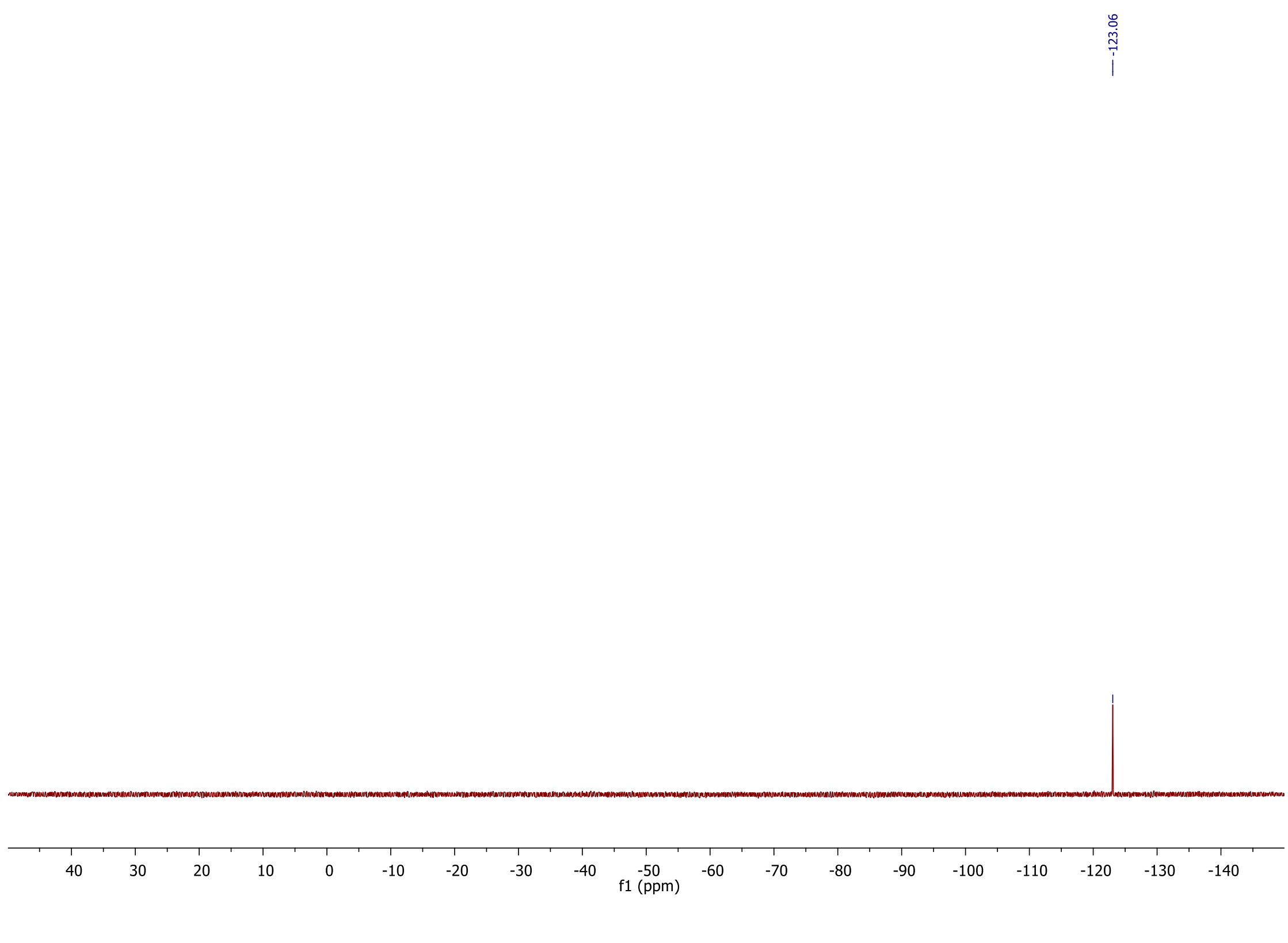

_N_-(4-(Ethylsulfonyl)thiazol-2-yl)-2-hydroxybenzamide (15a)

^{1}H NMR (500 MHz, $(CD_3)_2SO$)

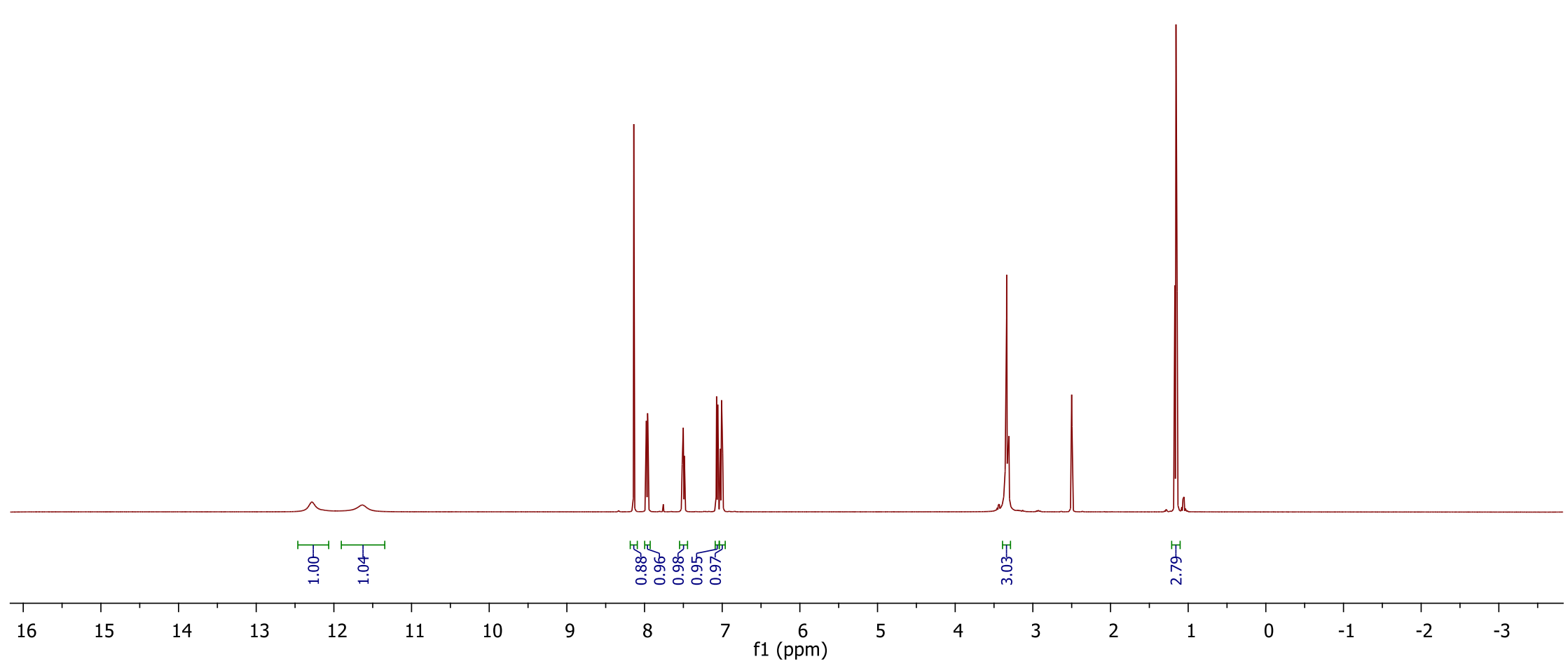

^{13}C NMR (126 MHz, $(CD_3)_2SO$)

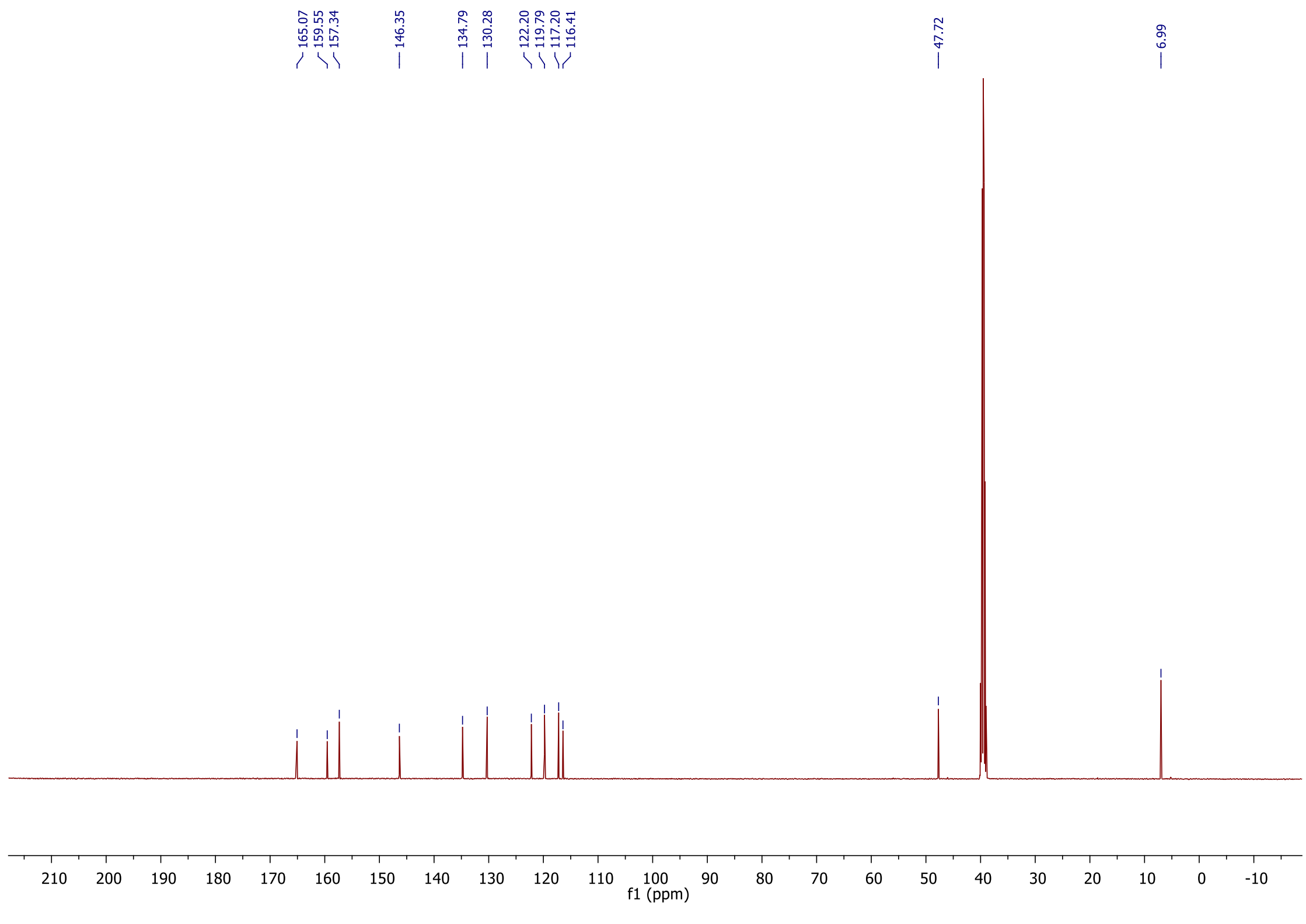

2-Hydroxy-*N*-(4-(*n*-propylsulfonyl)thiazol-2-yl)benzamide (15b)

^{1}H NMR (500 MHz, $(CD_3)_2SO$)

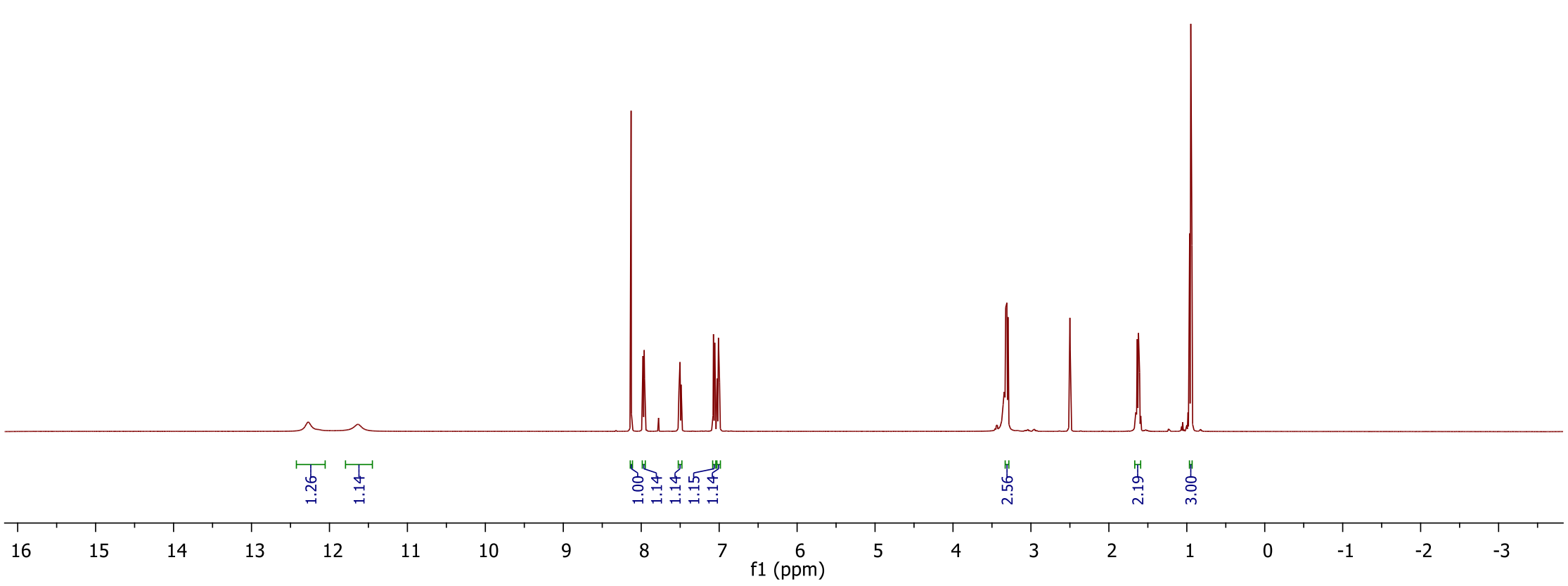

^{13}C NMR (126 MHz, $(CD_3)_2SO$)

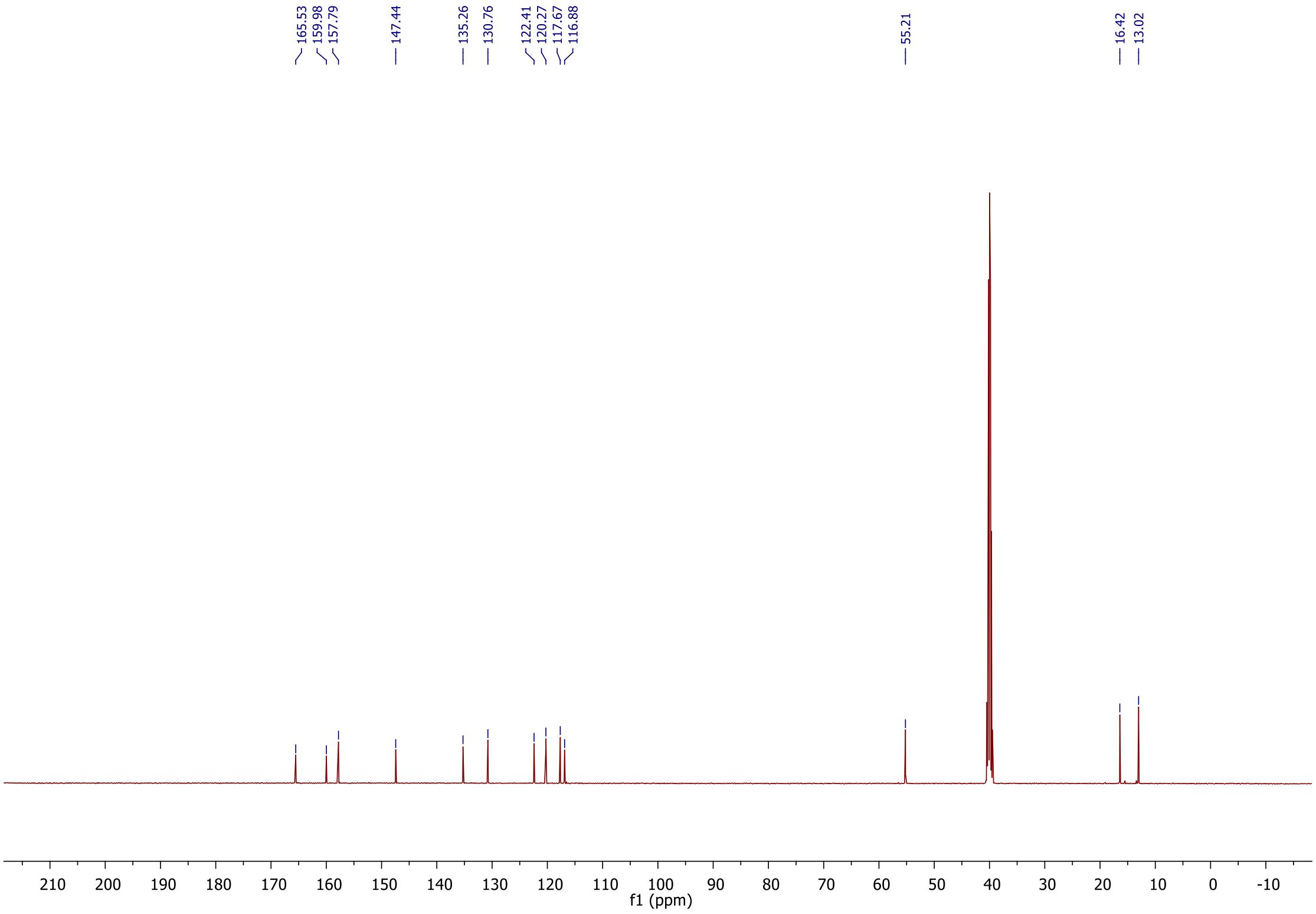

N-(4-(n-Butylsulfonyl)thiazol-2-yl)-2-hydroxybenzamide (15c)

^{1}H NMR (500 MHz, $(CD_3)_2SO$)

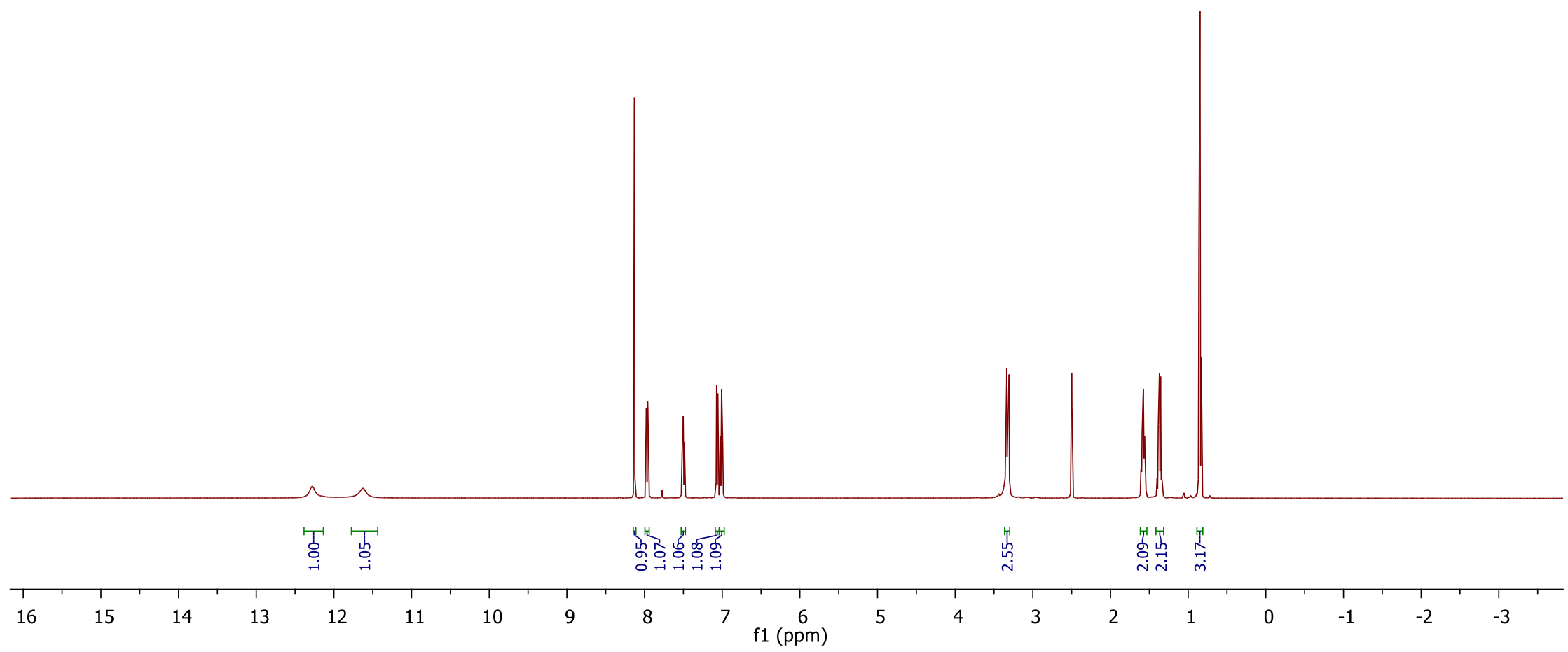

^{13}C NMR (126 MHz, $(CD_3)_2SO$)

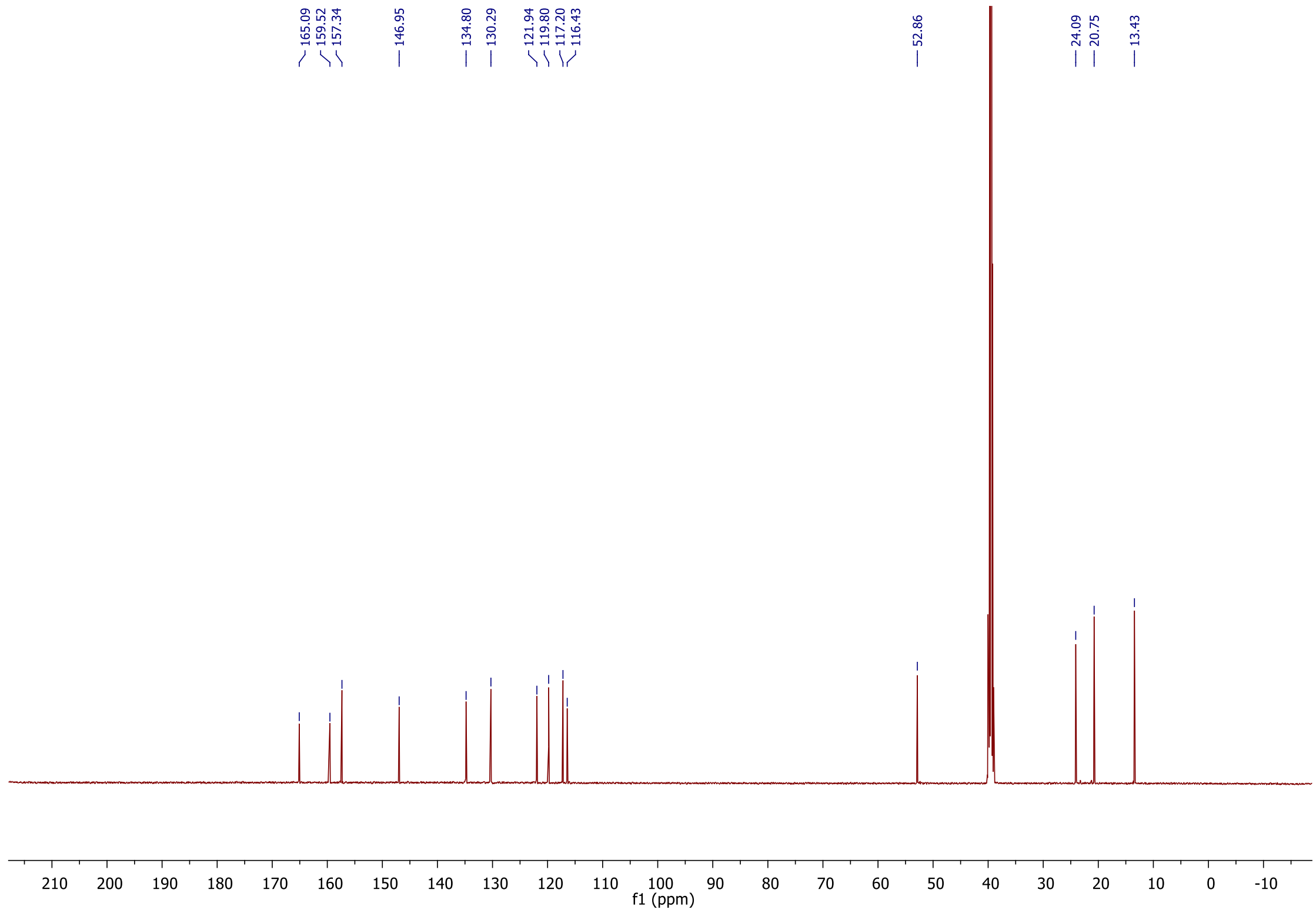

2-Hydroxy-*N*-(4-(isopropylsulfonyl)thiazol-2-yl)benzamide (15d)

^{1}H NMR (500 MHz, $(CD_3)_2SO$)

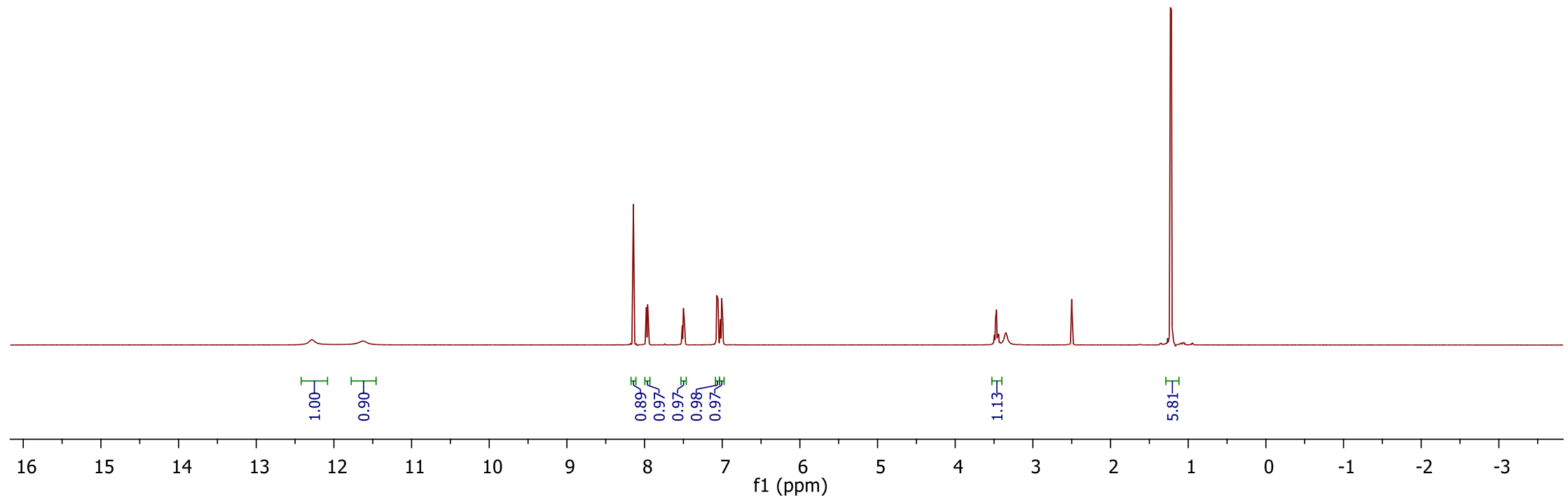

^{13}C NMR (126 MHz, $(CD_3)_2SO$)

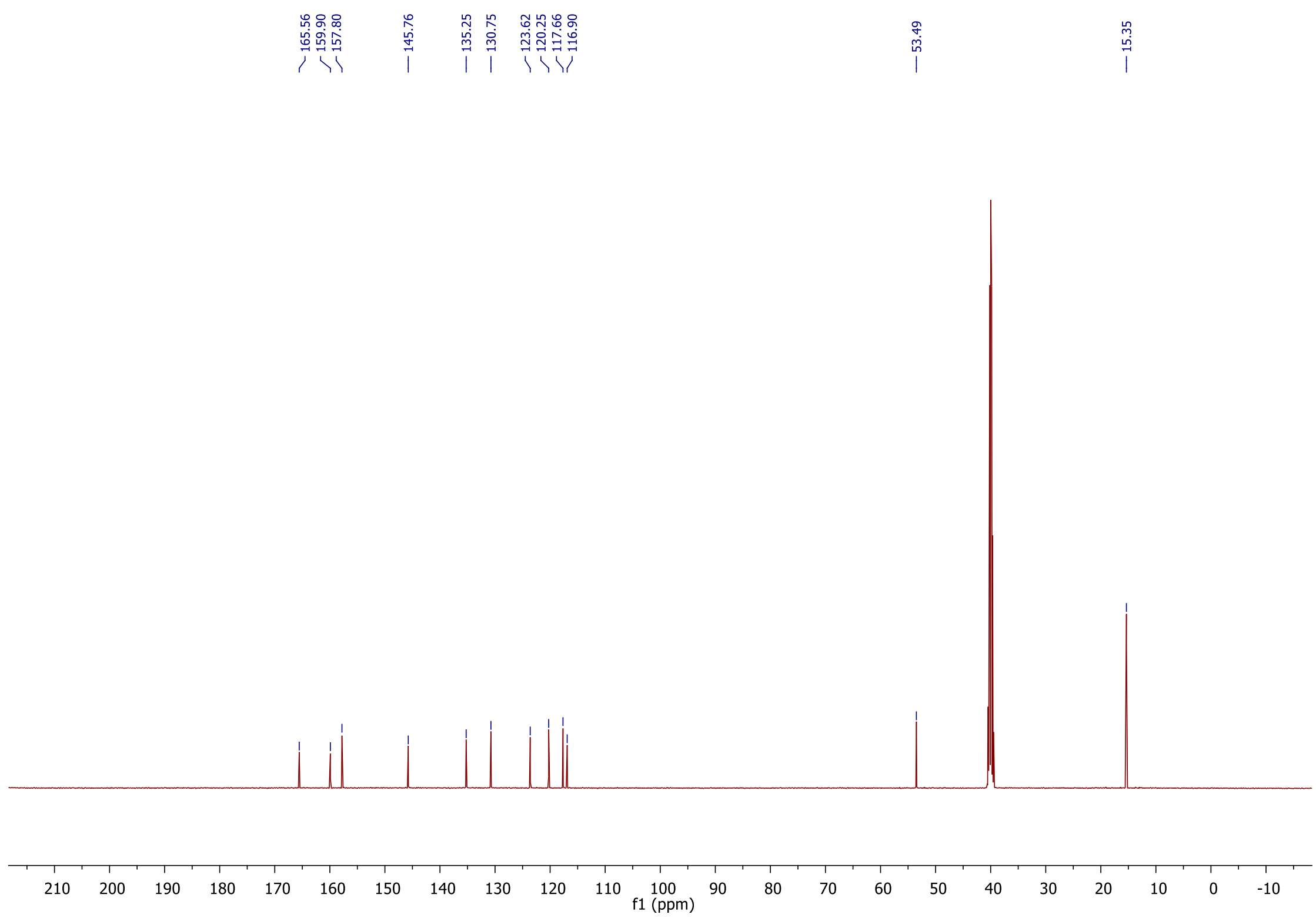

_N_-(4-(_tert_-Butylsulfonyl)thiazol-2-yl)-2-hydroxybenzamide (15e)

^{1}H NMR (500 MHz, $(CD_3)_2SO$)

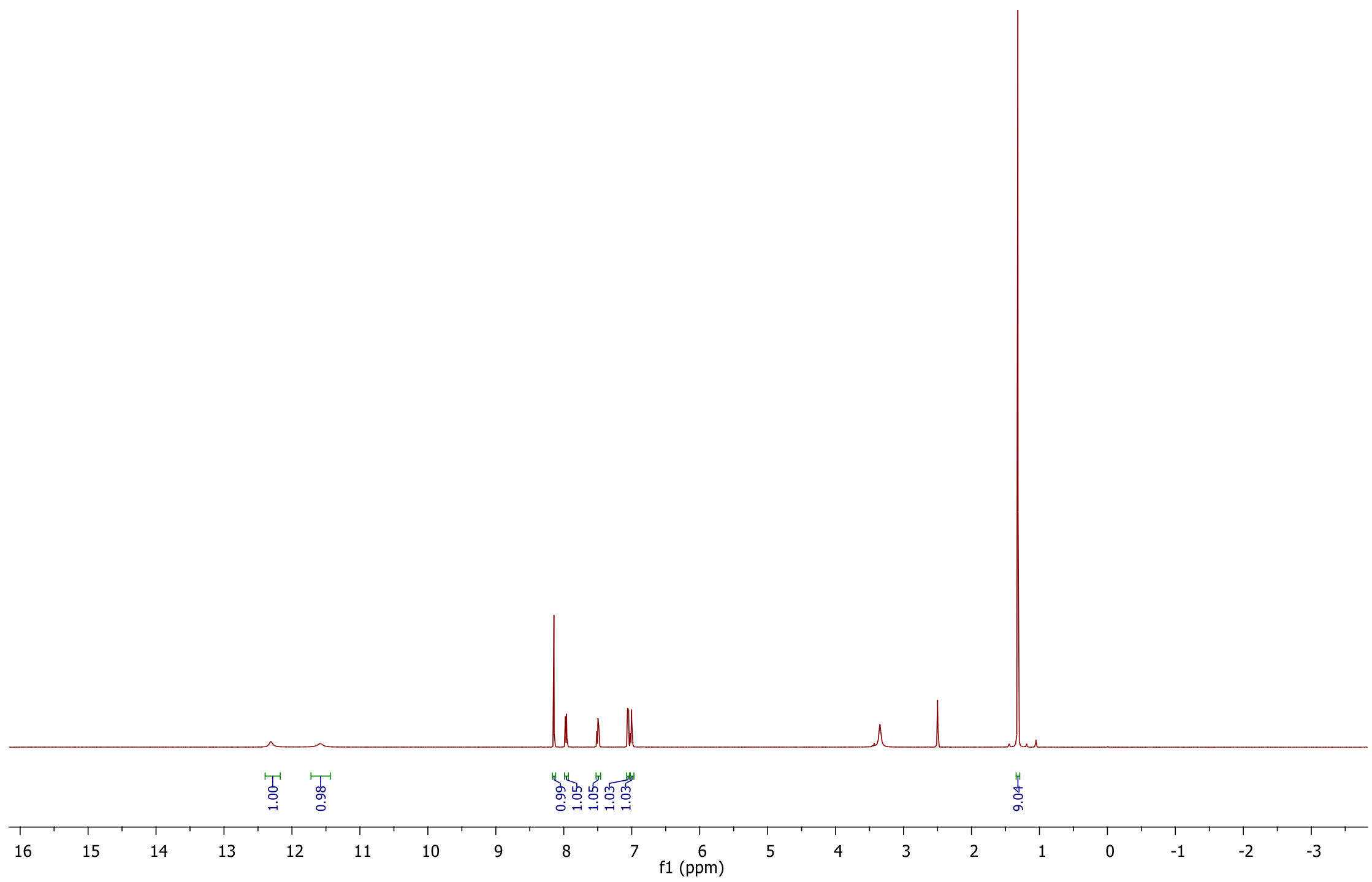

^{13}C NMR (126 MHz, $(CD_3)_2SO$)

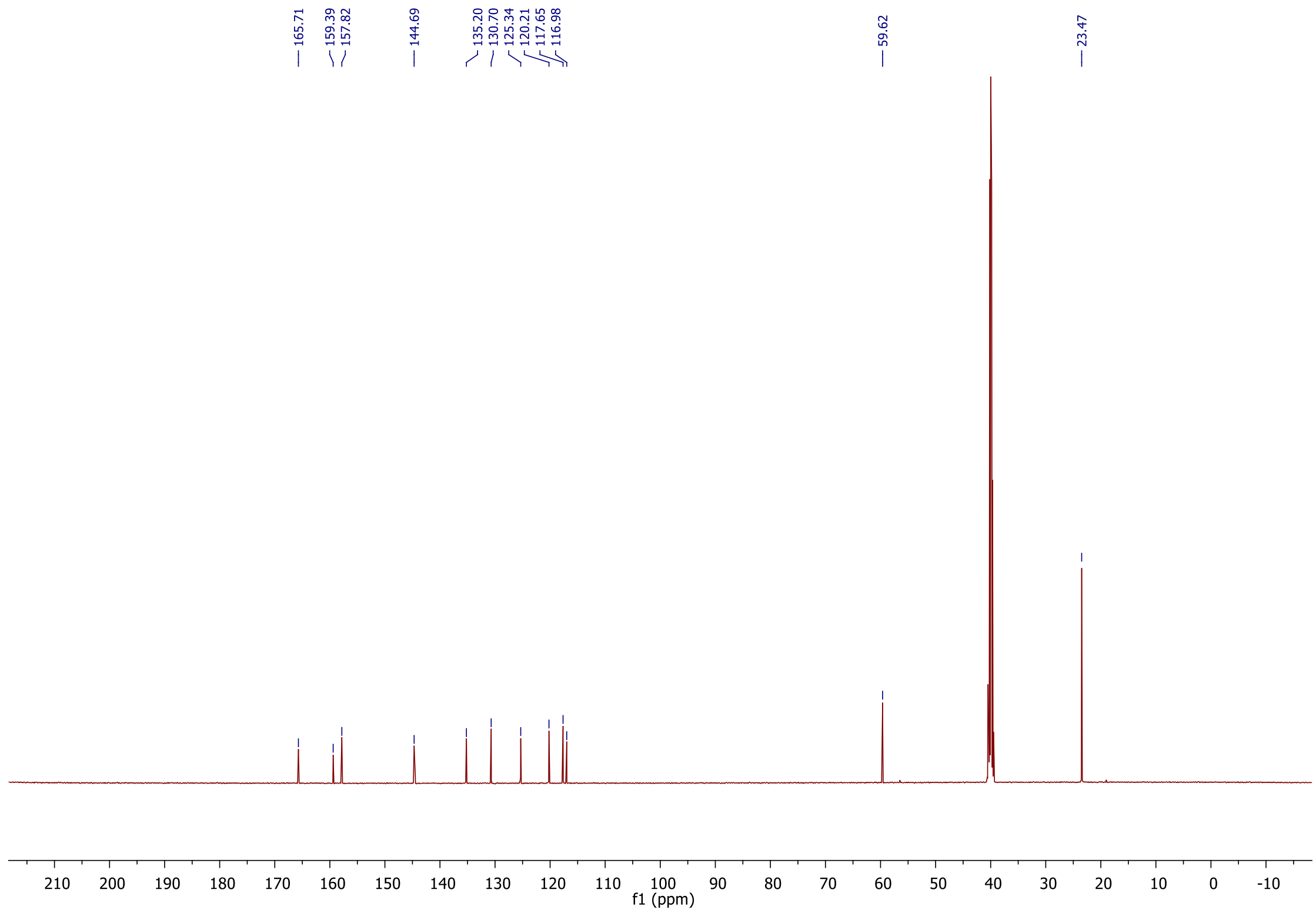

***N*-(4-(Cyclopentylsulfonyl)thiazol-2-yl)-2-hydroxybenzamide (15f)**

^{1}H NMR (500 MHz, $(CD_3)_2SO$)

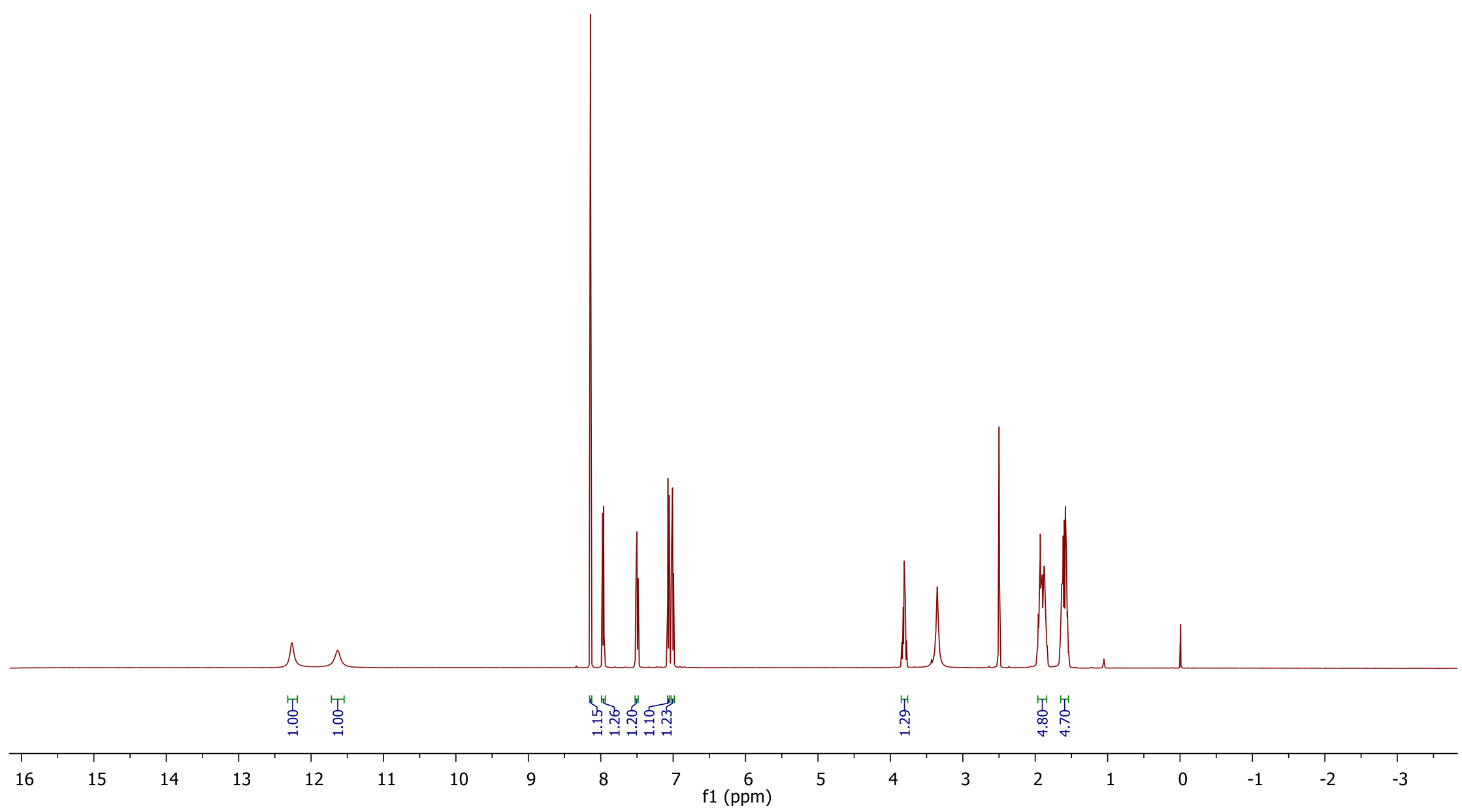

^{13}C NMR (126 MHz, $(CD_3)_2SO$)

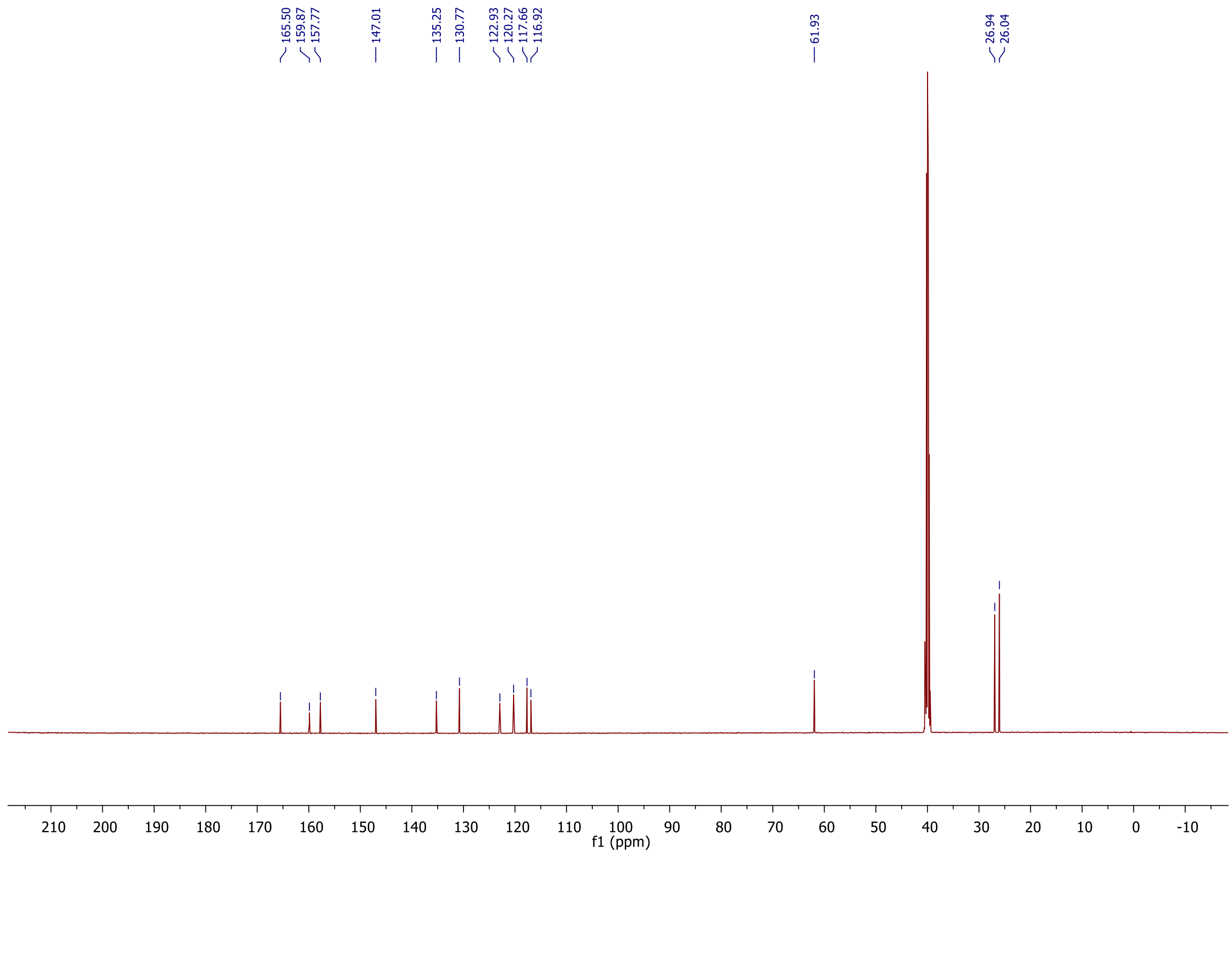

N-(4-((Cyclopropylmethyl)sulfonyl)thiazol-2-yl)-2-hydroxybenzamide (15g)

^{1}H NMR (500 MHz, $(CD_3)_2SO$)

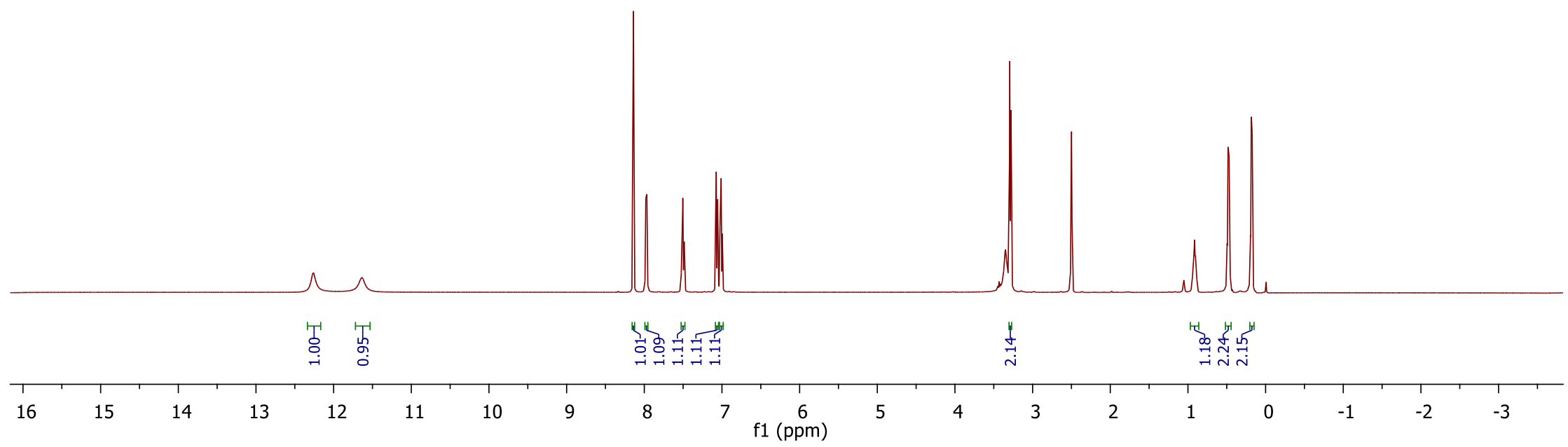

^{13}C NMR (126 MHz, $(CD_3)_2SO$)

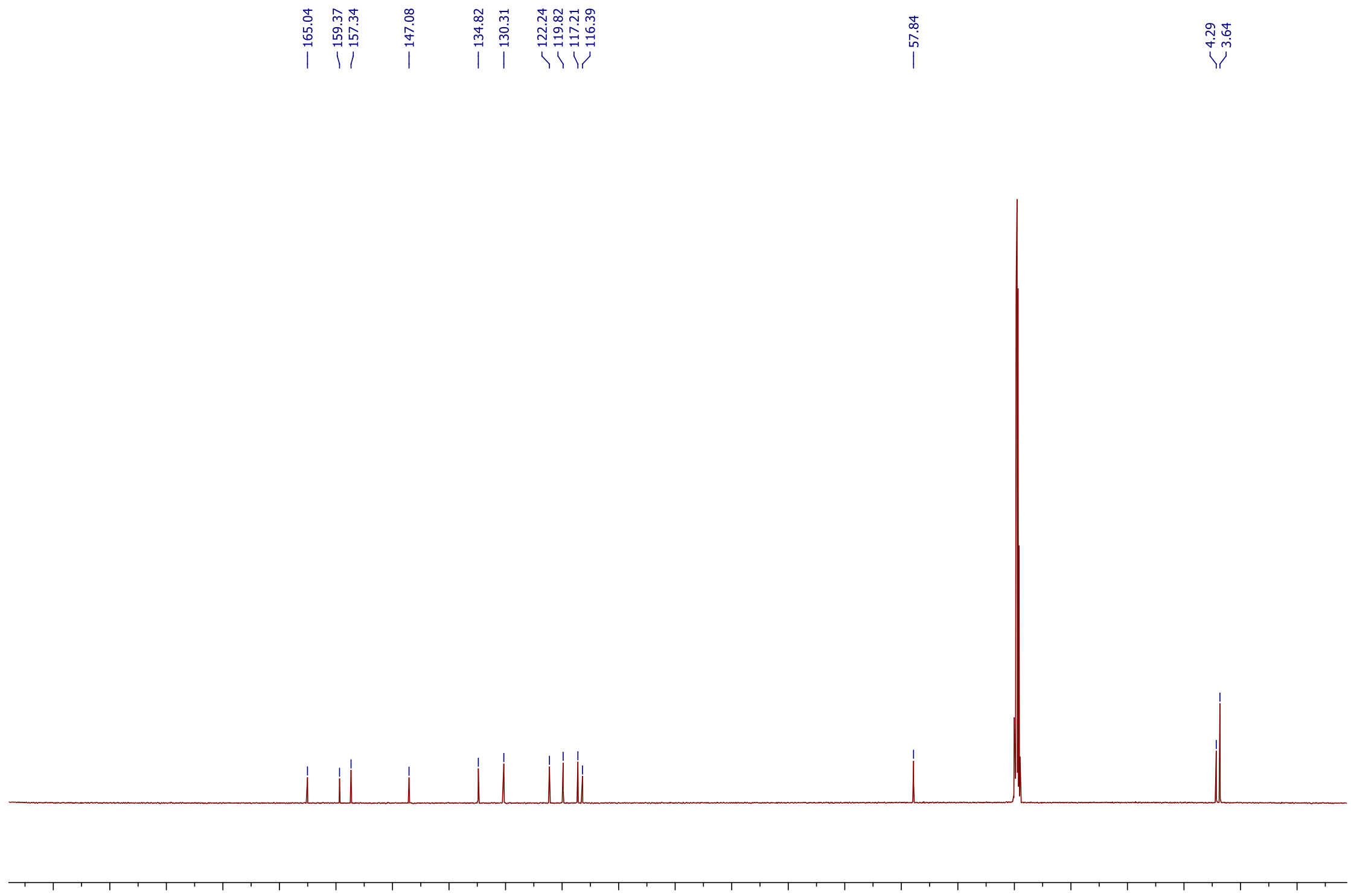

N-(4-((Cyclobutylmethyl)sulfonyl)thiazol-2-yl)-2-hydroxybenzamide (15h)

^{1}H NMR (500 MHz, $(CD_3)_2SO$)

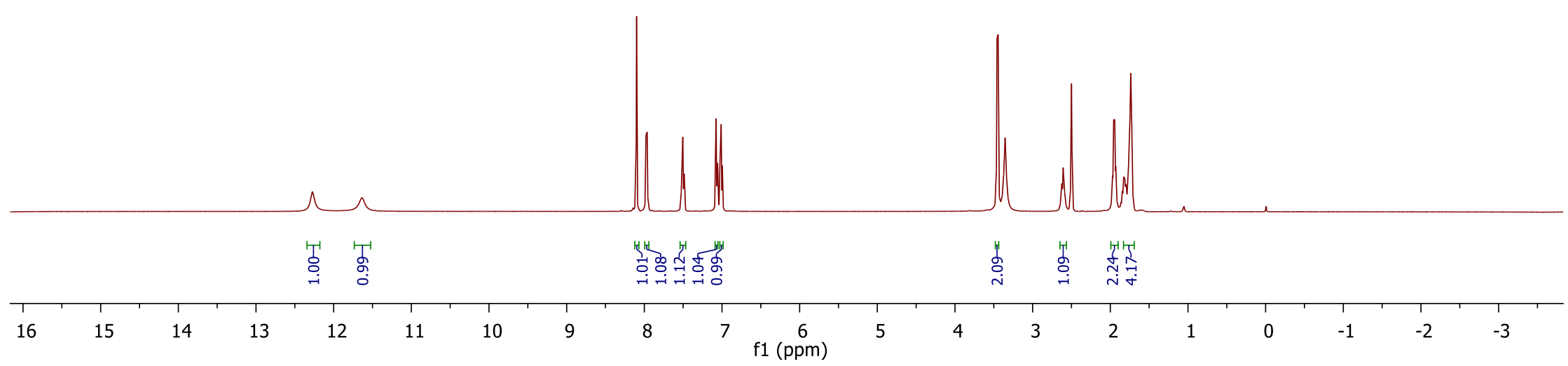

^{13}C NMR (126 MHz, $(CD_3)_2SO$)

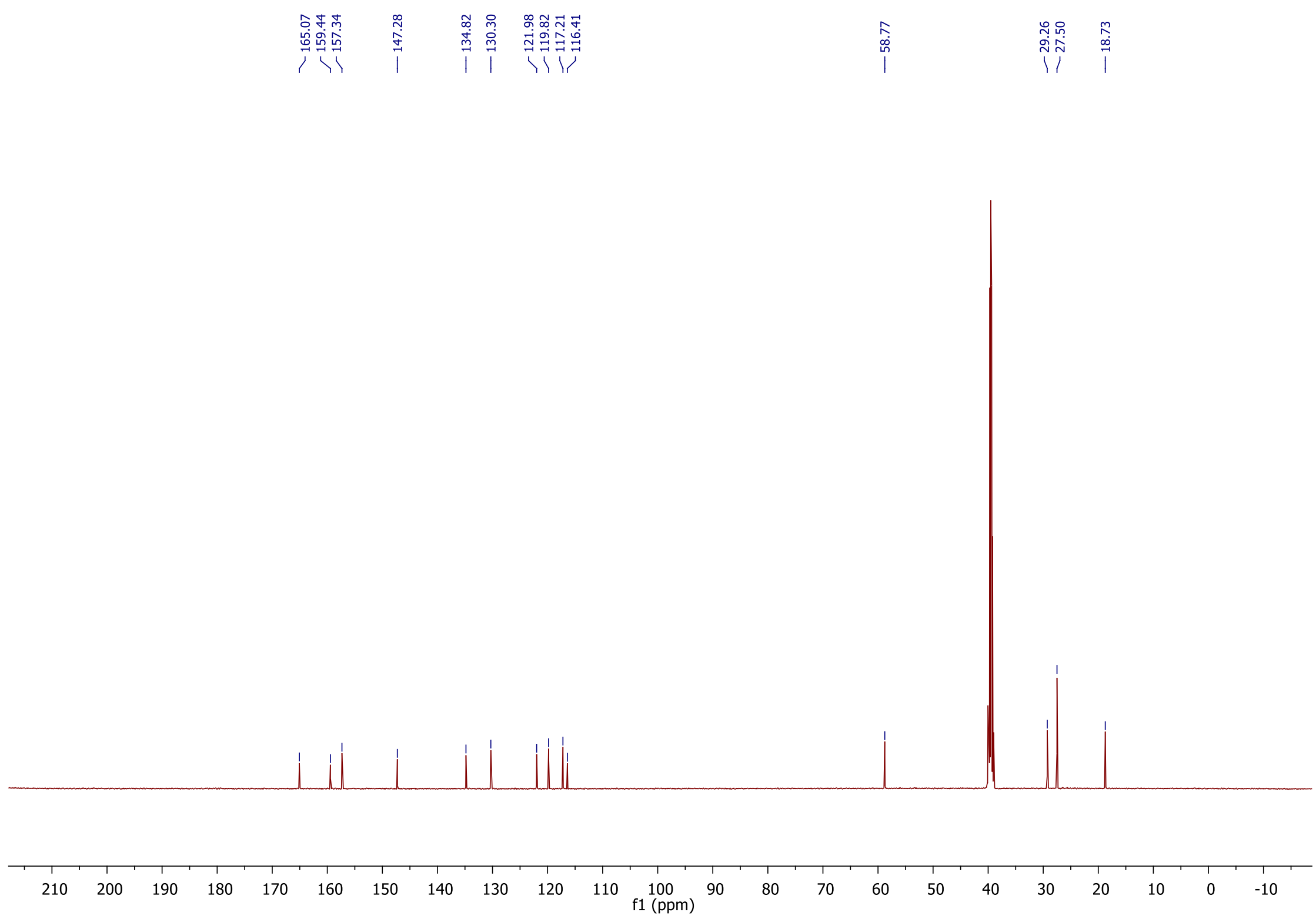

4. References

(1) Silverman, F. P.; Petracek, P. D.; Heiman, D. F.; Fledderman, C. M.; Warrior, P. Salicylate Activity. 3. Structure Relationship to Systemic Acquired Resistance. *J. Agric. Food Chem.* **2005**, *53* (25), 9775–9780. https://doi.org/10.1021/jf051383t.

(2) Dean, M. A.; Hitchcock, S. R. Synthesis and Application of Oxadiazines as Chiral Ligands for the Enantioselective Addition of Diethylzinc to Aldehydes. *Tetrahedron: Asymmetry* **2010**, *21* (20), 2471–2478. https://doi.org/10.1016/j.tetasy.2010.09.010.

(3) Tobe, M.; Isobe, Y.; Goto, Y.; Obara, F.; Tsuchiya, M.; Matsui, J.; Hirota, K.; Hayashi, H. Synthesis and Biological Evaluation of CX-659S and Its Related Compounds for Their Inhibitory Effects on the Delayed-Type Hypersensitivity Reaction. *Bioorganic & Medicinal Chemistry* **2000**, *8* (8), 2037–2047. https://doi.org/10.1016/S0968-0896(00)00126-7.

(4) Baecker, D.; Obermoser, V.; Kirchner, E. A.; Hupfauf, A.; Kircher, B.; Gust, R. Fluorination as Tool to Improve Bioanalytical Sensitivity and COX-2-Selective Antitumor Activity of Cobalt Alkyne Complexes. *Dalton Trans.* **2019**, *48* (42), 15856–15868. https://doi.org/10.1039/C9DT03330K.

(5) Sharma, H.; Patil, S.; Sanchez, T. W.; Neamati, N.; Schinazi, R. F.; Buolamwini, J. K. Synthesis, Biological Evaluation and 3D-QSAR Studies of 3-Keto Salicylic Acid Chalcones and Related Amides as Novel HIV-1 Integrase Inhibitors. *Bioorganic & Medicinal Chemistry* **2011**, *19* (6), 2030–2045. https://doi.org/10.1016/j.bmc.2011.01.047.

(6) Obermoser, V.; Baecker, D.; Schuster, C.; Braun, V.; Kircher, B.; Gust, R. Chlorinated Cobalt Alkyne Complexes Derived from Acetylsalicylic Acid as New Specific Antitumor Agents. *Dalton Trans.* **2018**, *47* (12), 4341–4351. https://doi.org/10.1039/C7DT04790H.

(7) Stachulski, A. V.; Pidathala, C.; Row, E. C.; Sharma, R.; Berry, N. G.; Lawrenson, A. S.; Moores, S. L.; Iqbal, M.; Bentley, J.; Allman, S. A.; Edwards, G.; Helm, A.; Hellier, J.; Korba, B. E.; Semple, J. E.; Rossignol, J.-F. Thiazolides as Novel Antiviral Agents. 2. Inhibition of Hepatitis C Virus Replication. *J. Med. Chem.* **2011**, *54* (24), 8670–8680. https://doi.org/10.1021/jm201264t.

(8) Stachulski, A. V.; Pidathala, C.; Row, E. C.; Sharma, R.; Berry, N. G.; Iqbal, M.; Bentley, J.; Allman, S. A.; Edwards, G.; Helm, A.; Hellier, J.; Korba, B. E.; Semple, J. E.; Rossignol, J.-F. Thiazolides as Novel Antiviral Agents. 1. Inhibition of Hepatitis B Virus Replication. *J. Med. Chem.* **2011**, *54* (12), 4119–4132. https://doi.org/10.1021/jm200153p.

(9) Jeankumar, V. U.; Chandran, M.; Samala, G.; Alvala, M.; Koushik, P. V.; Yogeeswari, P.; Salina, E. G.; Sriram, D. Development of 5-Nitrothiazole Derivatives: Identification of Leads against Both Replicative and Latent Mycobacterium Tuberculosis. *Bioorganic & Medicinal Chemistry Letters* **2012**, *22* (24), 7414–7417. https://doi.org/10.1016/j.bmcl.2012.10.060.

(10) Praveen Rao, P. N.; Amini, M.; Li, H.; Habeeb, A. G.; Knaus, E. E. Design, Synthesis, and Biological Evaluation of 6-Substituted-3-(4-Methanesulfonylphenyl)-4-Phenylpyran-2-Ones: A Novel Class of Diarylheterocyclic Selective Cyclooxygenase-2 Inhibitors. *J. Med. Chem.* **2003**, *46* (23), 4872–4882. https://doi.org/10.1021/jm0302391.

(11) Dagli, D. J.; Yu, P.-S.; Wemple, J. Darzens Synthesis of Glycidic Thiol Esters. Formation of a .Beta.-Lactone by-Product. *J. Org. Chem.* **1975**, *40* (22), 3173–3178. https://doi.org/10.1021/jo00910a003.

(12) Gennari, C.; Vulpetti, A.; Pain, G. Highly Enantio- and Diastereoselective Boron Aldol Reactions of α-Heterosubstituted Thioacetates with Aldehydes and Silyl Imines. *Tetrahedron* **1997**, *53* (16), 5909–5924. https://doi.org/10.1016/S0040-4020(97)00251-2.

(13) Rossignol, J.-F.; Semple, J. E. Compounds and Methods for Treating Influenza. CA3038405C, February 1, 2022.

(14) Iyori, Y.; Ueno, R.; Morishige, A.; Chatani, N. Nickel-Catalyzed C–O/N–H, C–S/N–H, and C–CN/N–H Annulation of Aromatic Amides with Alkynes: C–O, C–S, and C–CN Activation. *Chem. Sci.* **2021**, *12* (5), 1772–1777. https://doi.org/10.1039/D0SC06056A.

(15) Piacentini, S.; Riccio, A.; Santopolo, S.; Pauciullo, S.; La Frazia, S.; Rossi, A.; Rossignol, J.-F.; Santoro, M. G. The FDA-approved drug nitazoxanide is a potent inhibitor of human seasonal coronaviruses

acting at postentry level: effect on the viral spike glycoprotein. *Frontiers in Microbiology* **2023**, *14*, 1-13. https://doi.org/10.3389/fmicb.2023.1206951.
(16) La Frazia, S.; Piacentini, S.; Riccio, A.; Rossignol, J.-F.; Santoro, M. G. The Second-Generation Thiazolide Haloxanide Is a Potent Inhibitor of Avian Influenza Virus Replication. *Antiviral Research* **2018**, *157*, 159–168. https://doi.org/10.1016/j.antiviral.2018.06.008.
(17) Santoro, M.G.; Amici, C.; Elia, G.; Benedetto, A.; Garaci, E. Inhibition of virus protein glycosylation as the mechanism of the antiviral action of prostaglandin A in Sendai virus-infected cells. Journal of General Virology 1989, 70, 789-800. https://doi.org/10.1099/0022-1317-70-4-789.
(18) K. Doyle, H. Lönn, H. Käck, A. Van de Poël, S. Swallow, P. Gardiner, S. Connolly, J. Root, C. Wikell, G. Dahl, K. Stenvall, P. Johannesson, *J. Med. Chem.* **2016**, *59*, 9457–9472.
